# Effects of Maternal Nutritional Supplements and Dietary Interventions on Placental Complications: An Umbrella Review, Meta-Analysis and Evidence Map

**DOI:** 10.3390/nu13020472

**Published:** 2021-01-30

**Authors:** Mai-Lei Woo Kinshella, Shazmeen Omar, Kerri Scherbinsky, Marianne Vidler, Laura A. Magee, Peter von Dadelszen, Sophie E. Moore, Rajavel Elango, The PRECISE Conceptual Framework Working Group

**Affiliations:** 1 Department of Obstetrics and Gynaecology, BC Children’s and Women’s Hospital and University of British Columbia, Vancouver, BC V6Z 2K8, Canada; maggie.kinshella@cw.bc.ca (M.-L.W.K.); shazmeen.omar@cw.bc.ca (S.O.); Kerri.Scherbinsky@bcchr.ca (K.S.); Marianne.Vidler@cw.bc.ca (M.V.); laura.a.magee@kcl.ac.uk (L.A.M.); PVD@kcl.ac.uk (P.v.D.); 2 Department of Pediatrics, University of British Columbia, Vancouver, BC V6H 0B3, Canada; 3 Department of Women & Children’s Health, King’s College London, London WC2R 2LS, UK; sophie.moore@kcl.ac.uk; 4 Medical Research Council Unit, The Gambia at the London School of Hygiene and Tropical Medicine, Fajara, P.O. Box 273 Banjul, The Gambia; 5 School of Population and Public Health, University of British Columbia, Vancouver, BC V6T 1Z3, Canada; 6 Division of Neonatology, BC Women’s Hospital and Health Centre, Vancouver, BC V6H 3N1, Canada

**Keywords:** maternal dietary interventions, nutritional supplements, pre-eclampsia, small for gestational age, low birthweight, stillbirths, maternal mortality, umbrella review

## Abstract

The placenta is a vital, multi-functional organ that acts as an interface between maternal and fetal circulation during pregnancy. Nutritional deficiencies during pregnancy alter placental development and function, leading to adverse pregnancy outcomes, such as pre-eclampsia, infants with small for gestational age and low birthweight, preterm birth, stillbirths and maternal mortality. Maternal nutritional supplementation may help to mitigate the risks, but the evidence base is difficult to navigate. The primary purpose of this umbrella review is to map the evidence on the effects of maternal nutritional supplements and dietary interventions on pregnancy outcomes related to placental disorders and maternal mortality. A systematic search was performed on seven electronic databases, the PROSPERO register and references lists of identified papers. The results were screened in a three-stage process based on title, abstract and full-text by two independent reviewers. Randomized controlled trial meta-analyses on the efficacy of maternal nutritional supplements or dietary interventions were included. There were 91 meta-analyses included, covering 23 types of supplements and three types of dietary interventions. We found evidence that supports supplementary vitamin D and/or calcium, omega-3, multiple micronutrients, lipid-based nutrients, and balanced protein energy in reducing the risks of adverse maternal and fetal health outcomes. However, these findings are limited by poor quality of evidence. Nutrient combinations show promise and support a paradigm shift to maternal dietary balance, rather than single micronutrient deficiencies, to improve maternal and fetal health. The review is registered at PROSPERO (CRD42020160887).

## 1. Introduction

Sustainable Development Goal 2 aims to eradicate world hunger by 2030; however, the 2020 State of Food Security and Nutrition in the World report indicated that 8.9% of people in the world are undernourished, which has impacts on both maternal and fetal well-being [1]. Maternal undernutrition is known to have important impacts on fetal development and early infancy as the sole source of nutrients for a growing infant from conception through exclusive breastfeeding [2]. Undernourished mothers are more likely to have low birthweight (LBW) infants; preconception folate deficiencies are associated with neural tube defects and there is increasing recent understanding of the impact of early nutrition on chronic diseases in later life, through the “developmental origins of health and disease” (DOHaD) research focus [3].

While adverse effects of maternal undernutrition have traditionally focused on reduced maternal nutrient supply to the fetus, a review by Belkacemi et al. [4] highlighted the crucial mediating role of the placenta. The placenta is a multi-functional organ that acts as an interface between the maternal and fetal circulations [4]. Maternal undernutrition alters placental development and function, leading to fetal growth restriction [4]. Studies of the impact of severe maternal undernutrition during the Dutch Famine during the winter of 1944–1945 found associations between placental weight and rates of LBW, preterm births (PTB), stillbirths and neonatal death [5,6]. Additionally, the failure of the proper development of the placenta is associated with pre-eclampsia (PE), the most serious of the hypertensive disorders of pregnancy (HDP) and the second leading direct cause of maternal mortality worldwide [7,8,9]. HDPs are associated with an estimated 46,000 maternal deaths, 416,000 stillbirths and 1.5–2 million neonatal deaths annually [7]. The development of PE involves inadequate placentation, maternal inflammatory response and generalized endothelial dysfunction [8,9]. Nutrition plays an important role in placentation in part due to the clinical antioxidant and anti-inflammatory properties of certain micronutrients [10].

Nutritional supplementation is considered an important part of policies to protect vulnerable populations, such as pregnant and lactating mothers and their infants, from health risks associated with undernutrition [1]. Previous umbrella reviews report that calcium supplementation is promising for reducing the risk of PE, while there was a lack of evidence for supplementary vitamin C, E or D [10,11]. Additionally, zinc or vitamin D supplementation on their own may reduce the risk of PTB [12], and balanced protein energy and multiple micronutrients (MMN) may reduce the risk of small for gestational age (SGA) and vitamin A, calcium, MMN and antenatal nutritional counselling may protect against LBW [13,14]. However, since these umbrella reviews were published, new systematic reviews, as well as updated versions of Cochrane systematic reviews on nutritional supplements, have been published. In addition, while PE, SGA, LBW, PTB, stillbirths and maternal mortality may be interconnected through maternal nutritional influences on placental disorders, evidence of the efficacy of nutritional supplementation and dietary intervention trials has not yet been mapped across outcomes and a gap exists, particularly for stillbirth and maternal mortality. This umbrella review included a systematic review of existing meta-analyses of randomized controlled trials (RCTs) and generated an evidence map for the efficacy of nutritional supplements and dietary interventions for adverse outcomes related to placental disorders and maternal mortality.

## 2. Materials and Methods

This review was been developed in accordance with the Preferred Reporting Items for Systematic Reviews and Meta-Analysis (PRISMA) checklist (Table S1) [15]. The protocol was registered to PROSPERO (CRD42020160887) prior to conducting the review.

### 2.1. Objective

The primary objective of the umbrella review was to evaluate the effects of nutritional supplements and dietary interventions on pregnancy outcomes related to placental disorders or maternal mortality. The primary outcomes were PE (as defined by individual study authors), PTB (<37 weeks or as defined by study authors), SGA (<10th centile or SGA/intrauterine growth restriction as defined by study authors), LBW (<2500 g or as defined by study authors), stillbirth and maternal death (42 days postpartum or as defined by study authors). Secondary outcomes were severe PE, gestational hypertension, eclampsia, and HELLP syndrome (hemolysis, elevated liver enzymes and low platelet count; all as defined by study authors). The secondary objective of this review was to break down contributing studies by those conducted in high-income countries (HICs) and low- and middle-income countries (LMICs) and explore whether findings shifted when analyses were restricted to trials conducted in LMICs.

### 2.2. Study Inclusion and Exclusion

All reviews reporting meta-analyses of RCTs assessing the efficacy of nutritional supplements or dietary interventions on primary outcomes of interest among pregnant women or those planning on becoming pregnant were included. Reviews of observational studies and those that incorporate theoretical studies or published opinion as their primary source of evidence were excluded [16]. Existing umbrella reviews were excluded, but were reviewed for any meta-analyses not captured in searches. Reviews not written in English were excluded due to limited capacity of the review team to comprehensively search non-English databases. There were no restrictions on the date of publication.

### 2.3. Search Strategy

Searches were conducted on Medline Ovid, EBM Reviews (The Cochrane Database of Systematic Reviews, ACP Journal Club, Clinical Evidence, Evidence-Based Mental Health, Evidence-Based Nursing, Evidence Report/Technology Assessment), JBI Database of Systematic Reviews and Implementation Reports, Web of Science, Cumulative Index to Nursing and Allied Health (CINAHL) and Embase. Additionally, searches were supplemented by reviewing the Database of Abstracts of Reviews of Effects, the PROSPERO register and scanning reference lists. Searches were conducted from database inception to November 2019. Search terms are included in Table S2.

Results were screened in a three-stage process based on title, abstract and then full-text review in duplicate by two independent reviewers (MWK, SO) at each stage. Study selections were compared and discrepancies were resolved by discussion with a third reviewer (K.S.). Duplicates and studies that did not meet the selection criteria were removed at each round. Search results were uploaded in Mendeley (Elsevier, London, UK) to remove duplicates, then the reference list was uploaded to Excel (Microsoft, Redmond, Washington, USA) for study selection.

### 2.4. Data Extraction

Data were extracted in a two-stage process. From reviews, details were extracted regarding the intervention, outcome, number of RCT studies, number of participants, variation between studies and pooled results with 95% confidence intervals (CIs) on a piloted table in Word (Microsoft, Redmond, Washington, USA). Where there were multiple reviews for an intervention, individual RCTs were extracted from reviews, their full text located, and details extracted on their study design, country, participants, interventions, comparison, co-interventions, and outcomes relevant to the current overview review. Two reviewers independently extracted data from a sample of eligible studies until full agreement was achieved on the details pulled (five studies), with the remainder extracted by one reviewer (M.W.K.).

### 2.5. Quality and Risk of Bias Assessment

We assessed the quality of reviews using AMSTAR 2, a widely used and validated critical appraisal tool to evaluate systematic reviews of healthcare interventions [16,17,18,19]. The risk of bias (RoB) of individual RCTs was based on their assessment in the most recent, highest quality review, according to AMSTAR 2, and evaluated on random sequence generation, allocation concealment, blinding of participants, personnel and outcome assessment, incomplete outcome data, selective reporting, and any other bias [20]. If there was no existing review with an AMSTAR 2 rating of moderate or above, then RoB was assessed by independent review by two reviewers.

### 2.6. Data Analysis

Included reviews were narratively synthesized according to included interventions, key findings and quality assessment. Evidence from studies of similar design, data collection methodology, sample and outcomes reported were pooled using Review Manager (RevMan 5) and reported as risk ratios (RRs) with 95% CI. To overcome the challenge that meta-analyses of systematic reviews could repeat individual studies and consequently give too much statistic power and result in a misleading estimate, each of the included reviews were unpicked when there were multiple reviews on a given intervention and the results of the individual included studies combined [17]. Evidence was pooled according to Mantel–Haenszel random-effects model for analyses with substantial heterogeneity (I^2^ > 50%) and sensitivity analyses were planned a priori on primary outcomes, (i) excluding RCTs with high RoB and (ii) excluding studies conducted in HICs. Estimates of publication bias were considered using funnel plots if there were more than 10 included studies. Certainty of evidence of primary outcomes were mapped using GRADE (Grading of Recommendations Assessment, Development and Evaluation) and classified as high, moderate, low or very low [20]. Strength of association was evaluated according to the Harvard Cancer Index.

## 3. Results

### 3.1. Search Results

**Figure 1:**
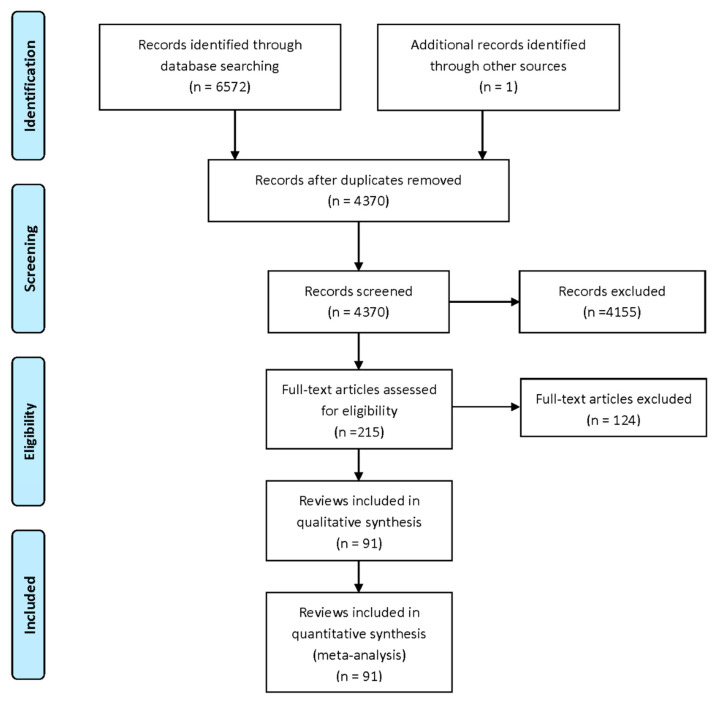
PRISMA flow diagram.

We identified 6572 records from database searches (2310 from Medline, 1268 from EBM reviews, 210 from JBI, 1468 from Web of Science, 665 from CINAHL and 651 from Embase). There were 4370 unique records after the removal of duplicates. After title and abstract screening, 215 records remained for full-text review (Figure 1). In total, 124 records were excluded at full-text review, including a mismatch in populations in a single study (i.e., supplementing infants), three in intervention (dietary interventions not reported separately), three in comparator (compared dosages, thus supplement given to both intervention and control), 12, which did not report outcomes of interest (i.e., infant mortality), 19 in study design (i.e., observational studies, commentary) and 48 duplicates and older versions of Cochrane reviews (Table S3). Nineteen umbrella reviews [10,11,12,13,14,21,22,23,24,25,26,27,28,29,30,31,32,33,34] were excluded and 19 reviews that did not report meta-analyses of RCTs (narrative syntheses only, meta-analysis of observational studies or combined observational and RCTs) (vitamin A [35,36,37], vitamin B6 and/or 12 [35,38], vitamin C and/or E [35,38,39], vitamin D and/or calcium [35,37,40,41,42], iron and/or folic acid [35,37,41,43,44], magnesium [35,37], zinc [45,46,47], MMN [35,37,41,48,49,50], balanced protein energy supplementation [51,52], antenatal dietary counselling [53]) were also excluded. The umbrella reviews found in searches were reviewed for unique meta-analyses; one was considered but was ultimately not included because, while a meta-analysis was planned, their searches found no studies for inclusion [49] (Table S4).

There were 91 meta-analyses in the umbrella review, including 23 Cochrane systematic reviews (latest versions) (Table S5). The interventions evaluated in the meta-analyses included 23 types of supplements (vitamins A, B6, C and/or E, D and/or calcium, iodine, iron and/or folic acid, magnesium, zinc, antioxidants, garlic, L-arginine, MMN, polyunsaturated omega-3 fatty acids, balanced protein-energy, high protein, calf blood extract, glucose, galactose, lipid-based nutrient supplements (LNS), food and fortified food products) and three types of dietary interventions (salt restriction, caffeine restriction, antenatal dietary counselling). L-arginine [54,55], antioxidants [56,57,58] and food and fortified food products [59] were not reported separately because they covered trials reported in other categories. For example, a study on the impact of L-arginine supplementation also included other vitamins and minerals and was included with multiple micronutrient supplementation [60].

### 3.2. Quality Assessment

**Figure 2:**
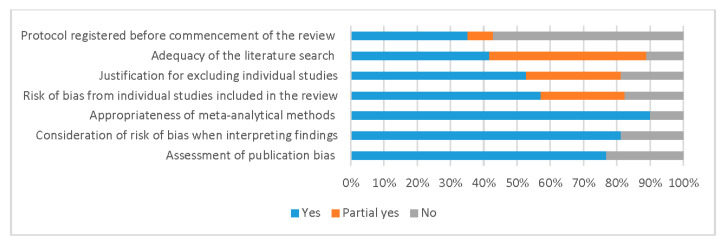
Quality assessment of reviews with meta-analyses.

Figure 2 reports the quality assessment, according to the AMSTAR 2 critical domains. Quality assessments by review are included in Table S6. Of the 91 included meta-analyses, 47% registered a review protocol prior to commencing, though 8% lacked detail on a meta-analysis plan and planned sensitivity analyses, and 89% had a fair to comprehensive literature search strategy. Excluded studies were reported in 82%, though 29% lacked referencing and/or justification details. Over half (57%) considered random allocation sequence, unconcealed allocation, blinding of patients and assessors and selective reporting in individual studies, though 82% included some form of RoB assessment. Ninety percent of included meta-analyses had appropriate methods, 81% considered RoB when interpreting the results of the review and 77% described the assessment or planned assessment of publication bias.

### 3.3. Effects of Nutrient Supplementation and Dietary Interventions

A summary of outcomes by review is reported in Table S7. The study characteristics of individual trials covered by reviews are included in Table S8, their individual trial outcomes in Table S9 and RoB in Table S10. Forest plots and funnel plots are included in Figure S1 and sensitivity analyses in Figure S2.

#### 3.3.1. Vitamin A

There were three reviews reporting meta-analyses on SGA, LBW, PTB, stillbirths and maternal deaths, consistently finding no effect of vitamin A and/or beta-carotene (vitamin A precursor) supplementation [61,62,63]. A Cochrane review also found no effect of vitamin A on maternal mortality in areas of high vitamin A deficiency [62]. The reviews covered ten trials published between 1999 and 2011 [64,65,66,67,68,69,70,71,72,73], with a secondary analysis published in 2013 [74]. Pooled outcomes confirmed no significant effect of vitamin A and/or beta-carotene supplementation across outcomes of interest. None of the studies reported PE outcomes, though one study found no significant effect on gestational hypertension [67] or rates of eclampsia [73]. Sensitivity analyses were not applicable as all studies had low or unclear RoB and all were conducted in LMICs.

#### 3.3.2. Vitamin C and/or E

Eight reviews found no significant effects of vitamin C and/or E supplementation [75,76,77,78,79,80,81,82]. The only significant finding was a weak adverse effect on LBW rates among women at risk of PE in the oldest review [79]. Among women with low baseline intake, two Cochrane reviews did not find a significant effect of vitamin C and/or E supplementation [75,82]. Reviews covered 19 trials examining vitamin C and/or E [83,84,85,86,87,88,89,90,91,92,93,94,95,96,97,98,99], including one secondary analysis of smokers [100]. Three trials were published in 2014 or later [84,92,100]. Pooled outcomes confirmed no significant effect of vitamin C and/or E supplementation across all outcomes of interest. Almost half of the trials were conducted in HICs [85,86,89,91,92,94,95,99,100]. Sensitivity analyses restricted to LMIC studies found a marginally significant protective effect on PE (RR 0.86, 95% CI: 0.76–0.99, I^2^ 40%, 11 studies, *n* = 6883); there were no changes in the direction or significance in other primary outcomes. Sensitivity analyses that excluded four studies considering high RoB [97,98,101,102] also found no changes in the direction or significance in primary outcomes. Vitamin C and/or E supplementation was not significantly associated with any of the secondary outcomes.

#### 3.3.3. Vitamin D

There were 13 reviews that reported meta-analyses for vitamin D supplementation [81,103,104,105,106,107,108,109,110,111,112,113,114]. Approximately half of reviews reported that vitamin D supplementation had a significant protective effect on PE incidence [81,107,109,110,113], SGA [103,104,111], LBW [105,111,113]. There was little evidence that vitamin D supplementation had an effect on PTB or stillbirth. A Cochrane review examined PE, LBW and PTB for studies conducted North of the Tropic of Cancer or South of the Tropic of Capricorn, where there is less sunlight exposure for the synthesis of vitamin D_3_. All studies reporting on PE included in the Cochrane review were conducted outside of the tropics, a non-significant effect on PTB did not change and there was a potentially stronger effect on LBW (all: RR 0.55, 95% CI: 0.35–0.87; outside tropics: RR 0.39, 95% CI: 0.24–0.65) though CIs overlap [113]. The reviews covered 25 trials [115,116,117,118,119,120,121,122,123,124,125,126,127,128,129,130,131,132,133,134,135,136,137,138,139], of which a majority were published in 2014 or later [117,118,119,121,122,123,125,126,127,128,129,130,132,133,137,138,139]. There were ten studies with high RoB [116,120,125,128,129,131,133,135,137,138] and ten were conducted in HICs [116,119,127,130,131,132,133,134,135,136]. Pooled outcomes found a 38% reduced risk of developing PE among pregnant women who received vitamin D supplementation in comparison to those who did not without heterogeneity between studies (RR 0.62 95% CI: 0.43–0.91, I^2^ 0%, 12 studies, *n* = 1353), which did not change in direction or significance with sensitivity analyses. Pooled outcomes also found that vitamin D supplementation reduced the risk of SGA by 41% (RR 0.59, 95% CI: 0.39–0.88, I^2^ 0%, five studies, *n* = 726), but the association was not significant after studies with a high RoB were excluded (RR 0.78, 95%CI: 0.42–1.44, I^2^ 0%, two studies, *n* = 305). Associations with LBW, PTB and stillbirth were non-significant, and no studies reported maternal mortality outcomes. For PTB, the 95% CI reached 1.00 in the fixed effects model but not in the random effects model; evidence for a non-significant association was strengthened in sensitivity analyses, excluding studies with a high RoB. Sensitivity analyses restricted to studies conducted in LMICs found a significantly reduced risk of PTB with vitamin D supplementation (RR 0.54 95% CI: 0.38–0.77, I^2^ 0%, 11 studies, *n* = 1154). Vitamin D supplementation was not significantly associated with any of the secondary outcomes.

#### 3.3.4. Vitamin D and Calcium

Four moderate-to-high quality reviews reporting meta-analyses on the effect of vitamin D and calcium co-supplementation [110,112,113,140] found a consistently significant effect on reduced rates of PE [110,112,113,140] but higher rates of PTB [113,140]. Results by baseline maternal dietary status were not reported by the two Cochrane reviews [113,141]. The reviews covered eight trials [120,121,142,143,144,145,146,147], of which two were published in 2014 or later [143,146], four had high RoB [120,144,145,147] and all were conducted in LMICs. Pooled outcomes found that vitamin D and calcium co-supplementation was associated with 51% reduced risk of PE [RR 0.49 (0.31–0.77, I^2^ 0%, three studies, *n* = 1120) and a 53% increased risk of PTB (RR 1.53 (1.02–2.30, I^2^ 0%, six studies, *n* = 988), though the adverse effect on PTB was not significant after studies with high RoB were excluded. There was no significant effect on SGA or LBW nor severe or gestational hypertension among secondary outcomes. No studies reported on stillbirth or maternal mortality outcomes and sensitivity analyses excluding studies from HICs were not applicable.

#### 3.3.5. Calcium

Calcium supplementation was evaluated in 19 meta-analyses [47,110,140,148,149,150,151,152,153,154,155,156,157,158,159,160,161,162,163], in which a majority were found to be protective against PE incidence, with the few reviews that reported no effects largely among subgroups, such as those with adequate baseline calcium intake [153,156] or featuring periconceptual supplementation [148]. A majority of reviews also reported significant beneficial effects of calcium supplementation on rates of PTB [47,140,149,150,160,161] but largely no effect on LBW or SGA. When outcomes were considered by baseline dietary calcium in a Cochrane review, only low calcium diet was significantly associated with lower rates of PE [140]. Results for LBW, SGA, PTB and stillbirths were non-significant for both those with low or adequate calcium baseline intake [140]. The reviews covered 23 trials [164,165,166,167,168,169,170,171,172,173,174,175,176,177,178,179,180,181,182,183,184,185,186], of which ten were considered studies with high RoB [164,165,167,170,173,176,179,182,184,186] and two studies were published in 2014 or later [164,185]. The majority of studies were conducted in LMICs with only six that were conducted in HICs [166,174,175,177,181,183]. Pooled outcomes found a 48% reduction in the risk of PE (RR 0.52, 95% CI: 0.41–0.65, I^2^ 67%, 24 studies, *n* = 27,442) with significant heterogeneity and publication bias suspected. Sensitivity analyses restricted to studies with low or unclear RoB and studies conducted in LMICs reduced the heterogeneity slightly, but this was still substantial (I^2^ 63–69%). Calcium supplementation was associated with a 16% risk reduction in LBW rates (RR 0.84, 95% CI: 0.73–0.96, I^2^ 43%, 11 studies, *n* = 7800). The effect was strengthened in sensitivity analyses limited to studies conducted in LMICs only (RR 0.58, 95% CI: 0.37–0.90, I^2^ 0%, five studies, *n* = 1110). Calcium supplementation was associated with a 47% reduced risk of PTB (RR 0.53, 95% CI: 0.33–0.86, I^2^ 95%, 18 studies, *n* = 14,078) but there was significant heterogeneity and publication bias suspected. Significant heterogeneity remained in sensitivity analyses. Calcium supplementation was not associated with SGA, stillbirth, or maternal mortality. Among secondary outcomes, there was a significant reduction in the risk of developing severe PE (RR 0.78, 95% CI: 0.78–0.93, I^2^ 0%, six studies, *n* = 14,099) and gestational hypertension (RR 0.75, 95% CI: 0.61–0.93, I^2^ 58%, 10 studies, *n* = 11,143) but not for eclampsia or HELLP syndrome.

#### 3.3.6. Iron and/or Folic Acid

There were nine reviews that reported meta-analyses for iron and/or folic acid supplementation [47,152,187,188,189,190,191,192,193], which largely found no effect on outcomes of interest. A network meta-analysis found that iron was significantly attributed to lower rates of PTB [47] and another review found that iron with or without folic acid supplementation had a weak effect on reducing rates of LBW [188]. A Cochrane review examined iron alone and iron with folic acid supplementation by baseline anemia status and found that studies were largely conducted with women who were non-anemic at the start of supplementation when reported [189]. The reviews covered 21 trials evaluating iron and/or folic acid supplementation [194,195,196,197,198,199,200,201,202,203,204,205,206,207,208,209,210,211,212,213,214], including nine trials with high RoB [195,197,200,201,203,206,207,213,214] and ten conducted in HICs [195,198,199,201,203,206,209,211,213,214]. None were published in 2014 or later. Pooled outcomes found that iron and/or folic acid significantly reduced rates of LBW by 13% (RR 0.87, 95% CI: 0.77–0.98, I^2^ 27%, 13 studies, *n* = 22,946), though the association was not significant after studies with high RoB were excluded. There was no evidence of a significant effect on PE, SGA, PTB, stillbirth, and maternal mortality, which were unchanged in the direction of effect and significance with sensitivity analyses. Sensitivity analyses were not applicable for maternal mortality. Among the secondary outcomes, only eclampsia was reported by one study, which found no effect [196].

#### 3.3.7. Zinc

There were four reviews that reported meta-analyses for zinc supplementation [42,45,46,47], which found evidence of a beneficial effect on PTB [45,46,47], except among adolescent pregnancies [42]. A review covering a single trial found a significant effect on LBW among adolescent pregnancies [42], though two other reviews did not find a significant effect on LBW or SGA [45,46]. A Cochrane review found that significant effects on PTB was only found among those with low baseline zinc, no effect remained for LBW and SGA, whether low or adequate baseline and maternal baseline zinc status were not reported for PE [46]. The reviews covered 15 trials [66,196,215,216,217,218,219,220,221,222,223,224,225,226,227,228,229], including five with high RoB [215,218,222,225,229], almost half of which were conducted in HICs [216,218,220,224,226,228,229] and none were published in 2014 or later. Pooled outcomes found a significant 14% reduction in the risk of PTB with zinc supplementation (RR 0.86, 95% CI: 0.76–0.97, I^2^ 17%, 16 studies, *n* = 7563), though the association was not significant after studies with high RoB were excluded. The association also lost its significance when restricted to studies in LMICs, although notably this excluded the six oldest studies conducted between 1984–1996. There was no significant effect on other primary outcomes and of the secondary outcomes, only one study reported on gestational hypertension, and the findings were not found to be significant [215]. In sensitivity analyses that excluded studies with high RoB, zinc was adversely associated with risk of PE (RR 3.62, 95% CI: 1.02–12.82, I^2^ N/A, 1 study, *n* = 479), though this was based on a single study from the late 1980s. Sensitivity analyses found no changes in the direction or significance in the associations with SGA and LBW and stillbirth and maternal mortality associations were uninterpretable due to the small number of included studies in the original analyses. 

#### 3.3.8. Multiple Micronutrients, with or without Lipid-Based Nutrient Supplementation

Eight reviews reporting meta-analyses evaluating the effect of MMN supplements on their own [47,81,230,231,232,233,234,235,236] found beneficial effects on LBW and SGA and mixed findings on PTB and stillbirth. Evidence of a beneficial effect on PE is limited by the report from only a single, poor quality review [81]. A Cochrane review found that the significant effect of MMN supplementation on PTB was only found in women with BMI ≤ 20 [233]. MMN supplementation was not significant for SGA regardless of BMI status [233]. The reviews on MMN supplementation covered 25 trials [60,67,196,198,204,210,237,238,239,240,241,242,243,244,245,246,247,248,249,250,251,252,253,254,255], which included three secondary analyses [256,257,258]. Of these, six were considered studies with a high RoB [60,241,242,243,244,248] and five studies were published in 2014 or later [241,249,252,253,255]. The majority of studies were conducted in LMIC settings, with one study conducted in a HIC [255]. Nine studies used the UNIMMAP multiple micronutrients formulation in their recommended dosages [242,243,245,247,248,249,250,251,254]. Pooled outcomes found a 60% reduced risk of PE (RR 0.40, 95% CI: 0.27–0.59, I^2^ 0%, two studies, *n* = 510], though the association was not significant after excluding one study with a high RoB in sensitivity analyses. Additionally, a significant reduction in the risks of SGA (RR 0.88, 95% CI: 0.82–0.95, I^2^ 59%, 16 studies, *n* = 39,696) and LBW (RR 0.87, 95% CI: 0.85–0.90, I^2^ 22%, 20 studies, *n* = 70,293) were also found with maternal MMN supplementation, with no change in direction or significance in sensitivity analyses. There was a high level of heterogeneity observed for SGA, which increased slightly in sensitivity analyses (I^2^ 62–67%). There was no evidence of a significant effect for PTB, stillbirth, maternal mortality nor severe PE, eclampsia or gestational hypertension among secondary outcomes.

There were two reviews reporting meta-analyses evaluating the effect of LNS [259,260], with one review reporting protective effects on SGA and LBW [260]. The reviews covered five trials [241,252,253,261,262], of which two were considered studies with a high RoB [241,261]. All were conducted in LMICs and four were published in 2014 or later [252,253,262,263]. Pooled outcomes found a significantly reduced risk of SGA (RR 0.93, 95% CI: 0.88–0.98, I^2^ 0%, four studies, *n* = 4828) and LBW (RR 0.89, 95% CI: 0.82–0.98, I^2^ 0%, five studies, *n* = 5851) among pregnant women receiving MMN with LNS, with less heterogeneity than MMN supplementation only. There was no evidence of a significant effect for PTB, stillbirth, or maternal mortality. No changes in direction or significance with the exclusion of studies with high RoB for all analyses (not applicable for maternal mortality). None reported on PE or secondary outcomes.

#### 3.3.9. Polyunsaturated Omega-3 Fatty Acids

There were 14 reviews with meta-analyses that evaluated the effect of polyunsaturated omega-3 fatty acid supplementation [264,265,266,267,268,269,270,271,272,273,274,275,276,277], with no largely significant effects, except some mixed findings on PTB. Maternal nutritional status at baseline was not reported by a Cochrane review, though when examining the underlying risk for outcomes, omega-3 supplementation had a significant effect for pre-eclampsia, only among women with high risk [274]. The reviews covered 35 trials [123,278,279,280,281,282,283,284,285,286,287,288,289,290,291,292,293,294,295,296,297,298,299,300,301,302,303,304,305,306,307,308,309,310], including two secondary analyses [311,312]. Nine studies were published in the year 2014 or later [281,283,288,289,290,291,296,306,310]. Twenty-three studies were conducted in HICs [279,280,281,285,286,288,289,290,291,292,293,294,295,298,299,300,302,304,305,307,308,309,310]. There were 14 studies considered to be at high RoB [279,280,281,286,287,290,291,295,298,301,302,304,306,308,310]. Three studies examined omega-3 and omega-6 co-supplementation [302,303,304]. Pooled outcomes found a 13% reduction in LBW (RR 0.87, 95% CI: 0.78–0.96, I^2^ 28%, 16 studies, *n* = 7940) and SGA (RR 0.85, 95% CI: 0.76–0.95, I^2^ 13%, 28 studies, *n* = 10,524) with omega-3 supplementation, which remained significant in sensitivity analyses that removed studies with high RoB, but not when studies were restricted to those conducted in LMICs. Additionally, while there was no significant effect on PE, SGA and stillbirths, sensitivity analyses restricting to studies in LMICs only found a significant effect on PE (RR 0.40, 95% CI: 0.21–0.77, I^2^ 0%, six studies, *n* = 1343). None reported maternal mortality outcomes. There was no significant effect for severe PE, eclampsia or gestational hypertension.

#### 3.3.10. Dietary Interventions

Based on a single Cochrane review for each exposure, there is no current evidence that dietary salt [313] or caffeine restriction [314] reduces rates of PE, SGA or PTB. LBW, stillbirth and maternal mortality were not explored in these reviews. From seven reviews that reported meta-analyses on the effect of antenatal diet and nutritional counselling [59,264,315,316,317,318,319], there is some evidence for a reduced risk of developing PE among normal weight women [264,318] but not among overweight or obese women [317]. Reviews reported mixed results on effects for LBW and PTB, with potentially significant effects for nutritional counselling to increase protein energy intake in particular [316], and consistently no effect across reviews on SGA. The reviews covered 21 trials with antenatal dietary counselling [310,320,321,322,323,324,325,326,327,328,329,330,331,332,333,334,335,336,337,338,339]; all but one [334] was conducted in a HIC. There were eight reviews at high RoB [310,320,323,327,329,331,332,334] and six published in 2014 or later [310,327,328,329,330,337]. Eight studies included diet and nutrition counseling, alongside physical activity promotion [322,323,324,325,326,327,328,329,330]. Pooled outcomes found a 28% reduction in PTB with antenatal dietary counselling (RR 0.72, 95% CI: 0.61–0.86, I^2^ 21%, 14 studies, *n* = 7612) and sensitivity analyses, excluding studies with high risk, did not change the direction of effect or significance and marginally lowered heterogeneity. Other primary outcomes were not significant and there was no evidence of a significant effect for severe PE or gestational hypertension among secondary outcomes. Almost all studies were conducted in HICs; therefore, sensitivity analyses restricted to studies conducted in LMICs were not applicable for primary outcomes, except in LBW, where antenatal dietary counselling was found to be significantly protective in one study with a high RoB [334].

#### 3.3.11. Other Nutrient Factors

One Cochrane review each reported on balanced protein energy supplementation [316], high protein supplementation [316], calf blood extract supplementation [340], glucose supplementation [340] and galactose supplementation [340]. Balanced protein energy supplementation was found to significantly reduce the risk of SGA (RR 0.79, 95% CI: 0.69–0.90, I^2^ 16%, seven studies, *n* = 4408) and stillbirth (RR 0.60, 95% CI 0.39–0.94, I^2^ 10%, five studies, *n* = 3408), while high protein supplementation may be associated with adverse effects on SGA (RR 1.58, 95% CI: 1.03–2.41, I^2^ N/A, one study, *n* = 505) [316]. Calf blood, glucose supplementation or galactose supplementation were not significantly associated with SGA [340]. Other primary outcomes were not reported. Additionally, a single Cochrane review each reported on vitamin B6 [341], iodine [342], magnesium [343], or garlic [344]. No significant associations were found (see Table 1).

### 3.4. Evidence Map

Table 1 is an evidence map summarizing the findings for included interventions. Details of the GRADE assessment for each intervention are included in Table S11. The map shows low to moderate certainty of the evidence for almost all interventions and outcomes; however, there was a high certainty of evidence for both vitamin D and combined vitamin D and calcium supplementation on the risk of PE and LNS on SGA. There was a high certainty of evidence for MMN, LNS and omega-3 supplementation on LBW rates. The map shows high certainty of evidence for omega-3 supplementation and antenatal dietary counselling on PTB rates. Evidence was poor in evaluating the effects of vitamin B6, iodine, garlic, balanced protein energy, high protein, calf blood extract, glucose and galactose supplementation, as well as salt and caffeine restriction dietary interventions. The availability of data was especially sparse for the effect of interventions on maternal mortality.

## 4. Discussion

The purpose of the umbrella review was to evaluate the current evidence on the effect of nutritional supplements and dietary interventions on PE, SGA, LBW, PTB, stillbirth and maternal death. There were few significant effects of interventions, though there were areas where evidence was sparse. Where there were significant associations, effect sizes were largely weak (RR 0.70–0.89) or not discernible (RR 0.90–0.99). Associations with moderate strength included a protective effect of vitamin D supplementation on PE and SGA, combined vitamin D and calcium supplementation on PE, calcium on PE and PTB, MMN on PE and balanced protein energy supplementation on risk of stillbirth. Balanced protein energy supplementation was the only intervention with evidence to reduce stillbirth rates. None were shown to reduce maternal mortality rates.

Considering all significant effects, our findings were largely similar to previous umbrella reviews and the most recent Cochrane reviews. We were able to include more trials in our updated umbrella review and this contributed to the few differences found in comparison with previous reviews. Similar to previous umbrella reviews, calcium supplementation was associated with a reduced risk of PE [10]. In addition, there was evidence to support vitamin D supplementation for PE prevention, which is in line with a network meta-analysis demonstrating that vitamin D is the most promising intervention in comparison to vitamin D and calcium co-supplementation or calcium on its own [110]. MMN supplementation may have been a promising intervention for PE prevention when individual trials were reviewed, which was previously not reported in reviews [10,233]. The findings showed that balanced protein energy supplementation and MMN supplementation was protective against SGA, which is in line with previous reviews [13,14]. Additionally, LNS may be protective against SGA when compared with iron–folic acid supplementation. Similar to a Cochrane review [345], there was a significant association of vitamin D with SGA, but there is some uncertainty as the association was not significant after excluding studies with high RoB. Findings showed that calcium, iron and/or folic acid, MMN, LNS and omega-3 supplementation was protective against LBW; this result builds on previous umbrella reviews where effectiveness was limited to MMN and calcium [13,14]. In contrast to previous umbrella reviews [13,14], but in line with a recent Cochrane review [62], there was no significant effect of vitamin A supplementation on reduced rates of LBW. This review expanded on findings of a previous overview review that zinc or vitamin D supplementation may help to lower rates of PTB [12] and also to suggest that calcium, omega-3 and antenatal dietary counselling may be beneficial. Similar to a previous umbrella review [11] and Cochrane review [113], it was also found that vitamin D and calcium co-supplementation may increase risk of PTB [12,113]. This review suggests uncertainty in the conclusion as the association was not significant after excluding studies with high RoB. This umbrella review adds to the literature by considering multiple interventions on multiple outcomes associated with placental conditions. No single intervention was effective in reducing risk across the six adverse pregnancy outcomes, highlighting that, while all may, in part, be associated with placental disorders, there is heterogeneity in the conditions and different pathways of influence. Interventions associated with protective effects on multiple outcomes included vitamin D (PE, SGA), calcium (PE, LBW, PTB), MMN (PE, SGA, LBW), LNS (SGA, LBW), omega-3 (LBW, PTB) and balanced protein energy supplementation (SGA, stillbirths).

Understanding the pathway to disease and the putative protective role each nutrient may help to explain the heterogeneity of the associations observed. Calcium supplementation may reduce risk of PE through reducing maternal hypertension [10]. Calcium is an important blood pressure regulator; low serum calcium levels increase the secretion of the parathyroid hormone, which increases intracellular calcium in vascular smooth muscle and leads to vasoconstriction [162]. Calcium supplementation decreases parathyroid hormone release and reduces smooth muscle contractility to lower blood pressure as well as potentially preventing preterm labor by reducing the uterine smooth muscle contractility [162]. Vitamin D supports calcium homeostasis by increasing calcium absorption in the intestine, but also helps to regulate the placental immune system and inflammation during placental development [346], which may help explain its effect on SGA, whereas its relationship with calcium may be more apparent in later pregnancy. Omega-3 fatty acids reduce oxidative stress and inflammation associated with PE development, as well as PTB risk factors [274,347,348]. The promising effect of balanced protein energy supplementation on fetal growth and stillbirth supports a shift from single micronutrient deficiencies, which has been much of the focus of the included studies to date, to a broader conceptualization of balanced nutrition and a combination of nutrients. This is also supported by the promising findings of MMN, LNS and vitamin D–calcium co-supplementation, though more research is needed on vitamin D–calcium co-supplementation to understand mechanisms of potential adverse effects on PTB.

A shift to considering diet quality and promoting healthy eating practices is highlighted in the latest WHO guidelines on antenatal care, which recommends counselling on healthy eating and keeping physically active during pregnancy [349]. Daily oral iron and folic acid supplementation, increasing daily energy and protein intake through education or supplementation in undernourished populations, calcium supplementation among women with low baseline intake, vitamin A supplementation among populations with severe deficiency and caffeine restriction among women with high intake were also recommended [349]. While our review did not find direct effects on our outcomes of interest, iron–folic acid supplementation is important in reducing maternal anemia [189], and there is a current gap in the literature on anemia as a potential mediator on the association between supplementation and pregnancy outcomes. Context-specific recommendations also underline the need to understand local malnutrition prevalence and maternal nutritional statuses. While inconsistently reported in trials, recent Cochrane reviews suggest the influence of baseline maternal nutritional status. Calcium supplementation was significantly protective against PE only among women with low calcium diets [140], zinc supplementation was protective against PTB only among those with low baseline zinc [46] and a significant effect of MMN supplementation on PTB was only found in women with BMI ≤ 20 [233]. We found protective effects of omega-3 and vitamin C/E supplementation on PE and vitamin D supplementation on PTB only among studies in LMICs. A significant effect of vitamin C/E supplementation on PE was particularly of note because antioxidant supplementation (i.e., vitamin A, C, E, garlic, etc.) was not associated with significant effects across outcomes, which is in line with existing reviews [9,82,347]. This suggests a need to further look at relationships between supplementation and underlying maternal nutritional deficiencies, which has been reported as common in LMICs [350]. However, the findings with vitamin C/E supplementation in LMICs must be taken with caution as effects were largely driven by two studies with unclear and high RoB. Lastly, the WHO recommendation on counselling on healthy eating during pregnancy is based largely on studies in HICs; whether these recommendations apply in LMIC settings, where food insecurity is common, needs to be explored in detail.

Although this umbrella review covers an impressive body of global research spanning decades, much remains inconclusive. First, some evidence for nutritional interventions are based on old and low-quality studies with small sample sizes, such as with iron and/or folic acid. Secondly, there are gaps in data; earlier studies did not always examine outcomes such as pregnancy hypertension, fetal growth restriction or stillbirths, and there is often a focus on infant outcomes even with recent studies. More studies measure LBW and PTB in almost all interventions and there are less that examine PE, stillbirth and maternal mortality. Pregnant and lactating mothers are targeted for interventions to improve infant health and growth, which represents a missed opportunity to also understand how nutritional interventions can help prevent maternal morbidities and mortality [351]. Third, nutritional interventions can be challenging to evaluate due to insufficient follow-up time in RCTs to detect dietary behavioral change, adequately accounting for underlying dietary deficiencies and routine prenatal supplementation and that RCTs may not always be ethically feasible when existing evidence supports a particular intervention or when a nutritional deficiency is known to be associated with adverse outcomes [352]. Consequently, while conventional systematic reviews value RCTs as the gold standard of study design and undervalue weak strength of associations, well-implemented observational cohort studies may also provide high quality evidence and small effect sizes may have a large impact at a population level [352]. Additionally, effects on outcomes such as stillbirth and maternal death may be confounded by access to quality maternity care [353,354]. The WHO antenatal care guidelines do not recommend MMN supplementation due to potential adverse effects on perinatal mortality [349], which some researchers highlighted that trials reporting adverse effects were conducted in poor rural settings where most mothers had no education and poor access to quality care [232]. They suggest that supplements need to be delivered, together with improving obstetric and postnatal care [232]. This highlights the necessity of nutrition-specific interventions, including nutritional supplementation, alongside nutrition-sensitive interventions, such as those around poverty, water and sanitation, food security, access to care and women’s empowerment working in tandem [355]. However, this also makes for complex interventions and, therefore, interpretation of results.

While this umbrella review comprehensively covered existing reviews to broadly map the evidence, it was limited in capturing the nuances of individual trials. Though most studies had common definitions, such as delivery <37 weeks gestation for PTB, outcome definitions sometimes varied. Maternal nutritional status at baseline and dosages or gestational age of supplementation were not explored, which could influence outcomes. Additionally, the review was limited by the availability of systematic reviews. While most intervention domains had a recent review with moderate to high quality rating, according to the AMSTAR 2 assessment, this may not have captured the most recent individual trials reported or interventions without coverage in a systematic review. For example, the analyses did not cover the FACT trial, a large international, multi-center RCT evaluating folic acid supplementation on pre-eclampsia, published in 2018, because it was not covered in existing systematic reviews [356].

## 5. Conclusions

In summary, this overview of the efficacy of nutritional supplements and dietary intervention on maternal mortality and placental disorders found evidence that supports vitamin D and/or calcium, omega-3, MMN, LNS and balanced protein energy supplementation to reduce the risks of multiple outcomes. However, more research is needed, particularly around potential adverse effects of vitamin D and calcium co-supplementation and high-protein maternal diets. Overall, these findings are limited by poor quality of evidence but show promise in considering combinations of nutrient factors and a strengthening of policy guidelines that focus on healthy eating and promote dietary balance rather than a focus on trials of single micronutrients.

## Figures and Tables

**Table 1:** Evidence map of direction of effect, strength of association and certainty of the evidence.

	PE			SGA		LBW		PTB		Stillbirth			Maternal Mortality
Nutritional supplements													
Vitamin A	N/A			RR 1.00 (0.98–1.03, I^2^ = 0%, 3 studies, *n* = 14,694) ●●●		RR 0.92 (0.75–1.13, I^2^ = 51%, 6 studies, *n* = 16,214) ●●		RR 0.98 (0.94–1.01, I^2^ = 20%, 6 studies, *n* = 40,788) ●●●		RR 0.97 (0.92–1.03, I^2^ = 0%, 4 studies, *n* = 140,145) ●●●			RR 0.82 (0.56–1.19, I^2^ = 62%, 5 studies, *n* = 161,474) ●●
Vitamin B6	RR 1.71 (0.85–3.45, I^2^ N/A, 2 studies, *n* = 1197) ●●			N/A		N/A		N/A		N/A			N/A
Vitamin C and/or E	RR 0.96 (0.89–1.04, I^2^ 33%, 19 studies, *n* = 24,819) ●●			RR 0.96 (0.89–1.03, I^2^ 24%, 13 studies, *n* = 21,964) ●●●		RR 0.93 (0.73–1.19, I^2^ 86%, 7 studies, *n* = 14,356) ●●		RR 1.00 (0.90–1.11, I^2^ 53%,17 studies, *n* = 23,687) ●●		RR 1.21 (0.92–1.58, I^2^ 0%, 9 studies, *n* = 19,908) ●●●			RR 0.60 (0.14–2.52, I^2^ 0%, 6 studies, *n* = 17,574) ●●●
Vitamin D	RR 0.62 (0.43–0.91, I^2^ 0%, 12 studies, *n* = 1353) ●●●●			RR 0.59 (0.39–0.88, I^2^ 0%, 5 studies, *n* = 726) ●●		RR 0.76 (0.54–1.06, I^2^ 64%, 6 studies, *n* = 792) ●●		RR 0.70 (0.49–1.00, I^2^ 39%, 17 studies, *n* = 4019) ●●●		RR 0.62 (0.19–2.00, I^2^ 0%, 5 studies, *n* = 860) ●●●			N/A
Vitamin D and calcium	RR 0.49 (0.31–0.77, I^2^ 0%, 3 studies, *n* = 1120) ●●●●			RR 0.90 (0.58–1.38, I^2^ NA, 1 study, *n* = 660) ●●		RR 0.63 (0.12–3.24, I^2^ 16%, 2 studies, *n* = 107) ●●		RR 1.53 (1.02–2.30, I^2^ 0%, 6 studies, *n* = 988) ●●●		N/A			N/A
Calcium	RR 0.52 (0.41–0.65, I^2^ 67%, 24 studies, *n* = 27,442) ●●			RR 1.01 (0.83–1.23, I^2^ 20%, 9 studies, *n* = 6407) ●●●		RR 0.84 (0.73–0.96, I^2^ 43%, 11 studies, n =7800) ●●●		RR 0.53 (0.33–0.86, I^2^ 95%, 18 studies, *n* = 14,078) ●●		RR 0.55 (0.24–1.23, I^2^ 75%, 7 studies, *n* = 10,687) ●●			RR 0.59 (0.18–1.92, I^2^ 40%, 5 studies, *n* = 10,057) ●●●
Iodine	N/A			RR 1.26 (0.77–2.05, I^2^ 0%, 2 studies, *n* = 377) ●●		RR 0.56 (0.26–1.20, I^2^ 0%, 2 studies, *n* = 377) ●●		RR 0.71 (0.30–1.66, I^2^ 32%, 2 studies, *n* = 376) ●●		N/A			N/A
Iron and/or folic acid	RR 0.99 (0.67–1.47, I^2^ 0%, 6 studies, *n* = 4454) ●●			RR 0.92 (0.80–1.06, I^2^ 67%, 7 studies, *n* = 8507 ●●		RR 0.87 (0.77–0.98, I^2^ 27%, 13 studies, *n* = 22,946) ●●●		RR 0.97 (0.89–1.06, I^2^ 0%, 15 studies, *n* = 24,420) ●●●		RR 0.88 (0.66–1.17, I^2^ 0%, 7 studies, *n* = 16,891) ●●●			N/A
Magnesium	RR 0.87 (0.58–1.32, I^2^ 0%, 3 studies, *n* = 1042) ●●●			RR 0.76 (0.54–1.07, I^2^ 7%, 3 studies, *n* = 1291) ●●●		RR 0.95 (0.83–1.09, I^2^ 22%, 5 studies, *n* = 5577) ●●●		RR 0.89 (0.69–1.14, I^2^ 37%, 7 studies, *n* = 5981) ●●●		RR 0.73 (0.43–1.25, I^2^ 0%, 4 studies, *n* = 5526) ●●			N/A
Zinc	RR 1.31 (0.82–2.10, I^2^ 39%, 5 studies, *n* = 2518) ●●			RR 1.03 (0.95–1.11, I^2^ 28%, 8 studies, n =4334) ●●●		RR 1.05 (0.94–1.17, I^2^ 26%, 12 studies, *n* = 5545) ●●		RR 0.86 (0.76–0.97, I^2^ 17%, 16 studies, *n* = 7563) ●●●		RR 0.20 (0.01–4.12, I^2^ NA, 2 studies, *n* = 376) ●●			RR 0.31 (0.01–7.43, I^2^ NA, 1 study, *n* = 85) ●●
Garlic	RR 0.78 (0.31–1.93, I^2^ N/A, 1 study, *n* = 100) ●●			N/A		N/A		N/A		N/A			N/A
Multiple micronutrients	RR 0.40 (0.27–0.59, I^2^ 0%, 2 studies, *n* = 510) ●●			RR 0.88 (0.82–0.95, I^2^ 59%, 16 studies, *n* = 39,696) ●●●		RR 0.87 (0.85–0.90, I^2^ 22%, 20 studies, *n* = 70,293) ●●●●		RR 0.93 (0.85–1.02, I^2^ 74%, 17 studies, *n* = 87,731) ●●		RR 1.09 (0.89–1.34, I^2^ 75%, 20 studies, *n* = 11,4385) ●●			RR 1.17 (0.82–1.67, I^2^ 0%, 8 studies, *n* = 84,081) ●●●
Lipid-based nutrients	N/A			RR 0.93 (0.88–0.98, I^2^ 0%, 4 studies, *n* = 4828) ●●●●		RR 0.89 (0.82–0.98, I^2^ 0%, 5 studies, *n* = 5851) ●●●●		RR 0.99 (0.86–1.14, I^2^ 0%, 5 studies, *n* = 6072) ●●●		RR 1.06 (0.60–1.87, I^2^ 55%, 4 studies, *n* = 6778) ●●			RR 0.52 (0.12–2.28, I^2^ 0%, 3 studies, *n* = 5628) ●●●
Balanced protein-energy	RR 1.48 (0.82–2.66, I^2^ N/A, 2 study, *n* = 463) ●●			RR 0.79 (0.69–0.90, I^2^ 16%, 7 studies, *n* = 4408) ●●●		N/A		RR 0.96 (0.80–1.16, I^2^ 0%, 5 studies, *n* = 3374) ●●		RR 0.60 (0.39–0.94, I^2^ 10%, 5 studies, *n* = 3408) ●●●			N/A
High protein	N/A			RR 1.58 (1.03–2.41, I^2^ N/A, 1 study, *n* = 505) ●●		N/A		N/A		RR 0.81 (0.31–2.15, I^2^ NA, 1 study, *n* = 529) ●●			N/A
Calf blood extract	N/A			RR 0.54 (0.20–1.47, I^2^ N/A, 1 study, *n* = 31)		N/A		N/A		N/A			N/A
Glucose	N/A			RR 1.11 (0.64–1.92, I^2^ N/A, 1 study, *n* = 30) ●		N/A		N/A		N/A			N/A
Galactose	N/A			RR 0.78 (0.39–1.54, I^2^ N/A, 1 study, *n* = 30) ●		N/A		N/A		N/A			N/A
Omega-3	RR 0.87 (0.71–1.07, I^2^ 9%, 18 studies, *n* = 7166) ●●●			RR 1.01 (0.90–1.13, I^2^ 0%, 8 studies, *n* = 6907) ●●●		RR 0.87 (0.79–0.96, I^2^ 28%, 16 studies, *n* = 7940) ●●●●		RR 0.85 (0.76–0.95, I^2^ 13%, 28 studies, *n* = 10,524) ●●●●		RR 0.89 (0.58–1.37, I^2^ 32%, 13 studies, *n* = 7547) ●●			N/A
Dietary interventions													
Salt restriction	RR 1.11 (0.46–2.66, I^2^ 0%, 2 studies, *n* = 603) ●●			RR 1.50 (0.73–3.07, I^2^ NA, 1 study, *n* = 242) ●●		N/A		RR 1.08 (0.46–2.56, I^2^ NA, 1 study, *n* = 242) ●●		N/A			N/A
Caffeine restriction	N/A			RR 0.97 (0.57–1.64, I^2^ NA, 1 study, *n* = 1150) ●●		N/A		RR 0.81 (0.48–1.37, I^2^ NA, 1 study, *n* = 1153) ●●		N/A			N/A
Antenatal dietary counselling	RR 0.97 (0.84–1.14, I^2^ 6%, 15 studies, *n* = 8087) ●●●			RR 1.15 (0.94–1.41, I^2^ 0%, 10 studies, *n* = 5529) ●●●		RR 0.54 (0.17–1.71, I^2^ 84%, 4 studies, *n* = 2271) ●●		RR 0.72 (0.61–0.86 I^2^ 21%, 14 studies, *n* = 7612) ●●●●		RR 0.63 (0.28–1.40, I^2^ 0%, 6 studies, *n* = 5631) ●●●			RR 1.08 (0.07–17.32, I^2^ NA, 1 study, *n* = 2122) ●●
**LEGEND** **Direction of effect and strength of association**													
Benefit—Strong effect (RR < 0.40)		Benefit—Moderate effect (RR 0.40–0.69)			Benefit—Weak effect (RR 0.70–0.89)				Benefit—Not discernible (RR 0.90–0.99)			No significant effect (95% CI crosses 1.00)	
Harm—Not discernable (RR 1.01–1.09)		Harm—Weak effect (RR 1.10–1.49)			Harm—Moderate effect (RR 1.50–2.99)				Harm—Strong effect (RR ≥ 3.00)			Not available	
**Certainty of the evidence**													
High ●●●●			Moderate ●●●				Low ●●				Very low ●		

PE—pre-eclampsia; SGA—small-for-gestational age; LBW—low birth weight; PTB—preterm birth.

## Data Availability

The data presented in this study are available in supplementary materials.

## Supplementary Materials

The following are available online at https://www.mdpi.com/2072-6643/13/2/472/s1, Table S1: PRISMA checklist, Table S2: Search terms, Table S3: Excluded studies, Table S4: Summary of overview reviews, Table S5: Included reviews by dietary factor, Table S6: Quality assessment for included reviews, Table S7: Summary of outcomes by reviews, Table S8: Study characteristics of individual trials covered in reviews, Table S9: Individual trials’ outcomes, Table S10: Risk of bias assessment, Table S11: GRADE, Figure S1: Primary analyses and funnel plots, Figure S2: Sensitivity analyses.
