# Supplementary material for: Effects of Maternal Nutritional Supplements and Dietary Interventions on Placental Complications: An Umbrella Review, Meta-Analysis and Evidence Map
Source: Nutrients. 2021 Jan 30;13(2):472. doi: 10.3390/nu13020472 (PMC7912620; doi:10.3390/nu13020472)
Supplement: Supplementary file 1 [file nutrients-13-00472-s001.zip › Supplementary files/Figures S1 - Analyses and funnel plots.docx]

**Figures S1–Primary analyses and funnel plots**

Contents

[1. Vitamin A 4](#_Toc55292971)

[1.1 Gestational hypertension 4](#_Toc55292972)

[1.2 Eclampsia 4](#_Toc55292973)

[1.3 Small for gestational age 4](#_Toc55292974)

[1.4 Low birthweight 5](#_Toc55292975)

[1.5 Preterm delivery 5](#_Toc55292976)

[1.6 Stillbirths 5](#_Toc55292977)

[1.7 Maternal mortality 6](#_Toc55292978)

[2. Vitamin C and/or E 6](#_Toc55292979)

[2.1 Pre-eclampsia 6](#_Toc55292980)

[2.2 Severe pre-eclampsia 7](#_Toc55292981)

[2.3 Gestational hypertension 8](#_Toc55292982)

[2.4 Eclampsia 8](#_Toc55292983)

[2.5 HELLP Syndrome 9](#_Toc55292984)

[2.6 Small for gestational age 9](#_Toc55292985)

[2.7 Low birthweight 10](#_Toc55292986)

[2.8 Preterm birth 11](#_Toc55292987)

[2.9 Stillbirth 12](#_Toc55292988)

[2.10 Maternal mortality 12](#_Toc55292989)

[3. Vitamin D and/or calcium 13](#_Toc55292990)

[3.1 Pre-eclampsia 13](#_Toc55292991)

[3.2 Severe pre-eclampsia 14](#_Toc55292992)

[3.3 Gestational hypertension 15](#_Toc55292993)

[3.4 Eclampsia 16](#_Toc55292994)

[3.5 HELLP Syndrome 16](#_Toc55292995)

[3.6 Small for gestational age 17](#_Toc55292996)

[3.7 Low birthweight 18](#_Toc55292997)

[3.8 Preterm birth 19](#_Toc55292998)

[3.9 Stillbirth 20](#_Toc55292999)

[3.10 Maternal mortality 21](#_Toc55293000)

[4. Iron and/or folic acid 21](#_Toc55293001)

[4.1 Pre-eclampsia 21](#_Toc55293002)

[4.2 Eclampsia 22](#_Toc55293003)

[4.3 Small for gestational age 22](#_Toc55293004)

[4.4 Low birthweight 23](#_Toc55293005)

[4.5 Preterm birth 24](#_Toc55293006)

[4.6 Stillbirth 25](#_Toc55293007)

[4.7 Maternal mortality 25](#_Toc55293008)

[5. Zinc 26](#_Toc55293009)

[5.1 Pre-eclampsia 26](#_Toc55293010)

[5.2 Gestational hypertension 26](#_Toc55293011)

[5.3 Small for gestational age 26](#_Toc55293012)

[5.4 Low birthweight 27](#_Toc55293013)

[5.5 Preterm birth 27](#_Toc55293014)

[5.6 Stillbirth 28](#_Toc55293015)

[5.7 Maternal mortality 28](#_Toc55293016)

[6. Multiple micronutrients 28](#_Toc55293017)

[6.1 Pre-eclampsia 28](#_Toc55293018)

[6.2 Severe pre-eclampsia 29](#_Toc55293019)

[6.3 Gestational hypertension 29](#_Toc55293020)

[6.4 Eclampsia 29](#_Toc55293021)

[6.5 Small for gestational age 30](#_Toc55293022)

[6.6 Low birthweight 31](#_Toc55293023)

[6.7 Preterm birth 32](#_Toc55293024)

[6.8 Stillbirth 33](#_Toc55293025)

[6.9 Maternal mortality 33](#_Toc55293026)

[7. Lipid-based nutrients 34](#_Toc55293027)

[7.1 Small for gestational age 34](#_Toc55293028)

[7.2 Low birthweight 34](#_Toc55293029)

[7.3 Preterm birth 34](#_Toc55293030)

[7.4 Stillbirth 35](#_Toc55293031)

[7.5 Maternal mortality 35](#_Toc55293032)

[8. Polyunsaturated omega-3 fatty acid 36](#_Toc55293033)

[8.1 Pre-eclampsia 36](#_Toc55293034)

[8.2 Severe pre-eclampsia 37](#_Toc55293035)

[8.3 Gestational hypertension 37](#_Toc55293036)

[8.4 Eclampsia 37](#_Toc55293037)

[8.5 Small for gestational age 38](#_Toc55293038)

[8.6 Low birthweight 39](#_Toc55293039)

[8.7 Preterm birth 40](#_Toc55293040)

[8.8 Stillbirth 41](#_Toc55293041)

[8.9 Maternal mortality 42](#_Toc55293042)

[9. Antenatal dietary counselling with or without physical activity promotion 43](#_Toc55293043)

[9.1 Pre-eclampsia 43](#_Toc55293044)

[9.2 Severe pre-eclampsia 44](#_Toc55293045)

[9.3 Gestational hypertension 44](#_Toc55293046)

[9.4 Small for gestational age 45](#_Toc55293047)

[9.5 Low birthweight 46](#_Toc55293048)

[9.6 Preterm birth 46](#_Toc55293049)

[9.7 Stillbirth 47](#_Toc55293050)

[9.8 Maternal mortality 48](#_Toc55293051)

[10. Sensitivity analyses 48](#_Toc55293052)

[10.1 Vitamin A 48](#_Toc55293053)

[10.1.1 Studies with low/unclear risk of bias only 48](#_Toc55293054)

[10.1.2 LMIC studies only 48](#_Toc55293055)

[10.2 Vitamin C and/or E 49](#_Toc55293056)

[10.2.1 Studies with low/unclear risk of bias only 49](#_Toc55293057)

[10.2.2 LMIC studies only 52](#_Toc55293058)

[10.3 Vitamin D and/or calcium 56](#_Toc55293059)

[10.3.1 Studies with low/unclear risk of bias only 56](#_Toc55293060)

[10.3.2 LMIC studies only 61](#_Toc55293061)

[10.4 Iron and/or folic acid 65](#_Toc55293062)

[10.4.1 Studies with low/unclear risk of bias only 65](#_Toc55293063)

[10.4.2 LMIC studies only 69](#_Toc55293064)

[10.5 Zinc 72](#_Toc55293065)

[10.5.1 Studies with low/unclear risk of bias only 72](#_Toc55293066)

[10.5.2 LMIC studies only 74](#_Toc55293067)

# 1. Vitamin A

## 1.1 Gestational hypertension

*no funnel plot because number of included studies <10
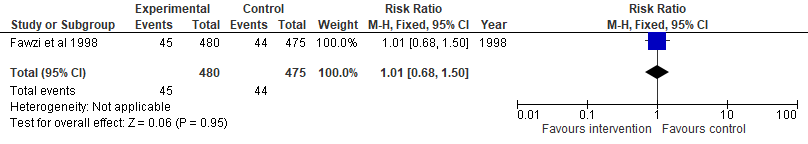


## 1.2 Eclampsia

*no funnel plot because number of included studies <10**
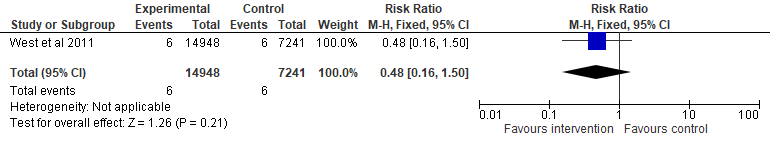
**

## 1.3 Small for gestational age

*no funnel plot because number of included studies <10
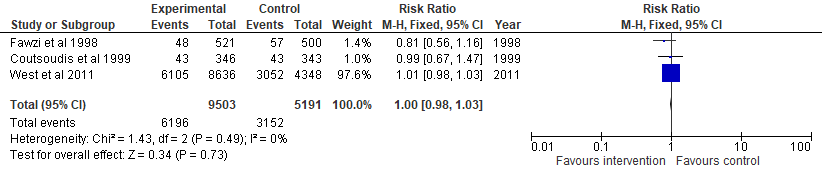


## 1.4 Low birthweight

*no funnel plot because number of included studies <10**
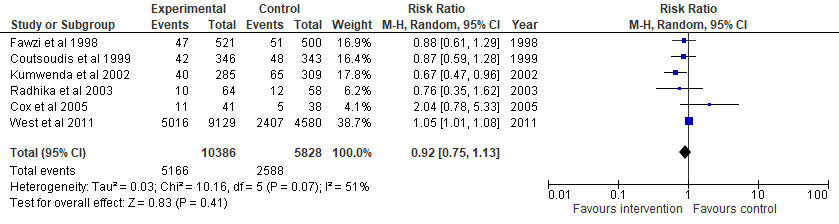
**

## 1.5 Preterm delivery

*no funnel plot because number of included studies <10**
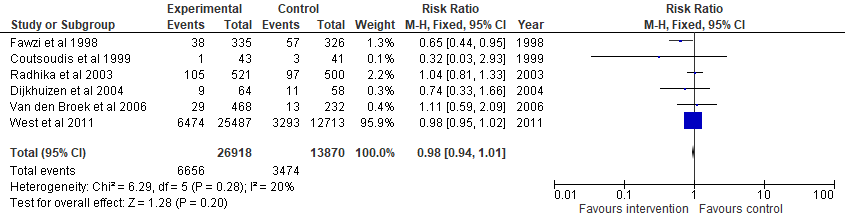
**

## 1.6 Stillbirths

*no funnel plot because number of included studies <10**
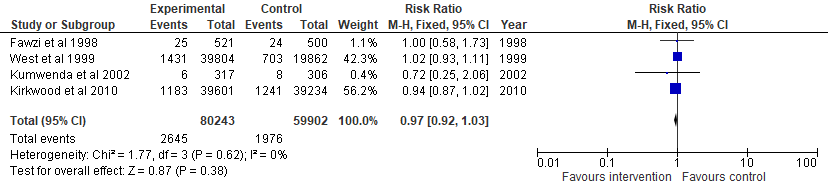
**

## 1.7 Maternal mortality

*no funnel plot because number of included studies <10**
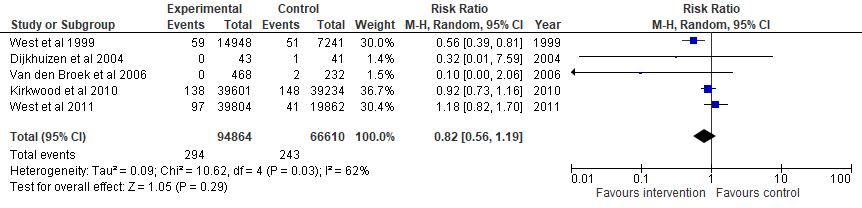
**

# 2. Vitamin C and/or E

## 2.1 Pre-eclampsia


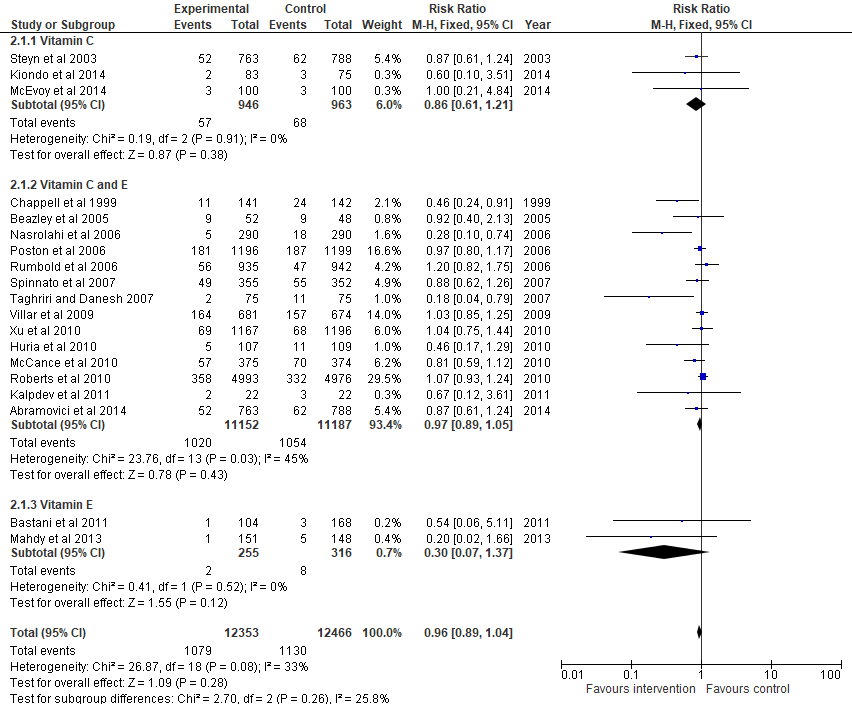


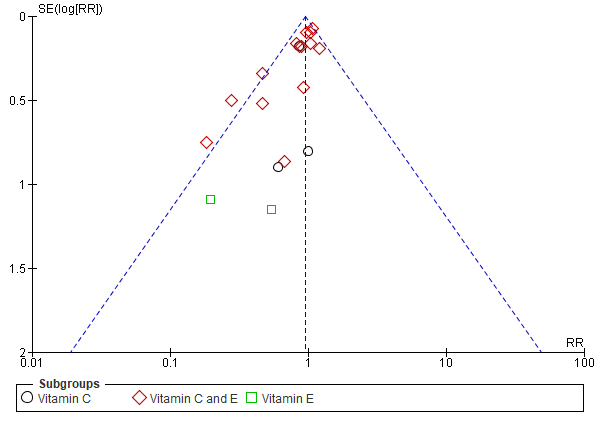


## 2.2 Severe pre-eclampsia

**
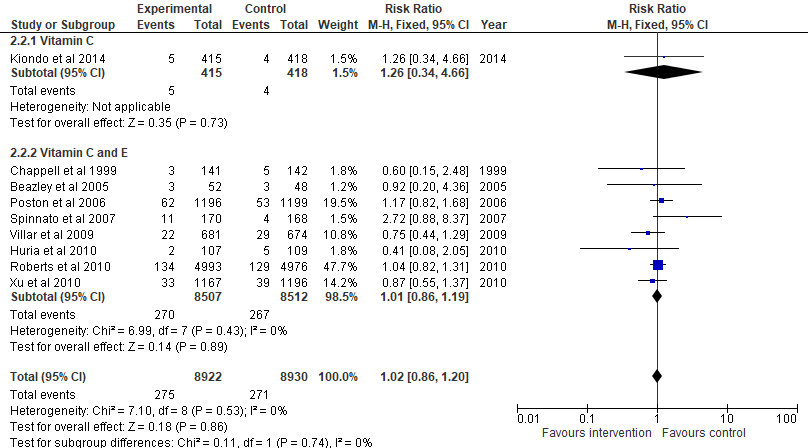
**

*no funnel plot because number of included studies <10

## 2.3 Gestational hypertension

**
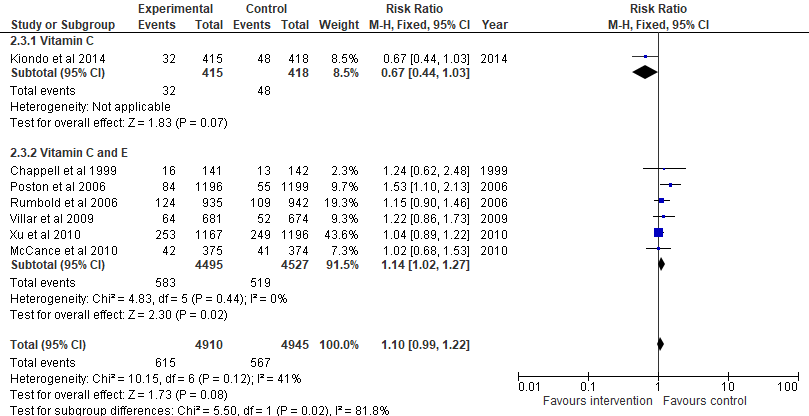
**

*no funnel plot because number of included studies <10

## 2.4 Eclampsia

**
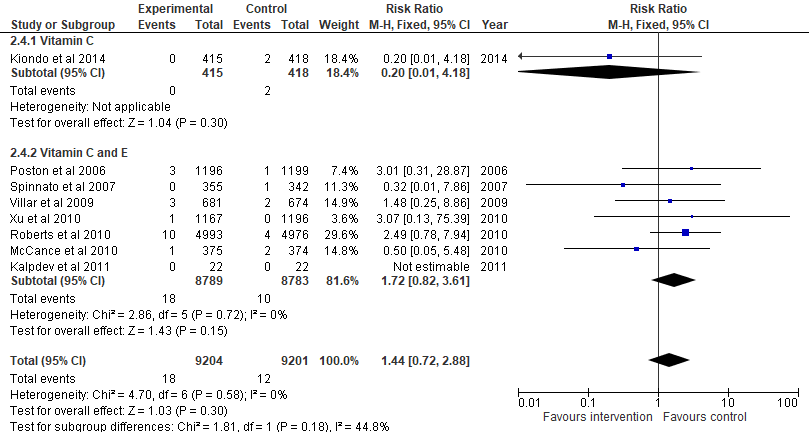
**

*no funnel plot because number of included studies <10

## 2.5 HELLP Syndrome

**
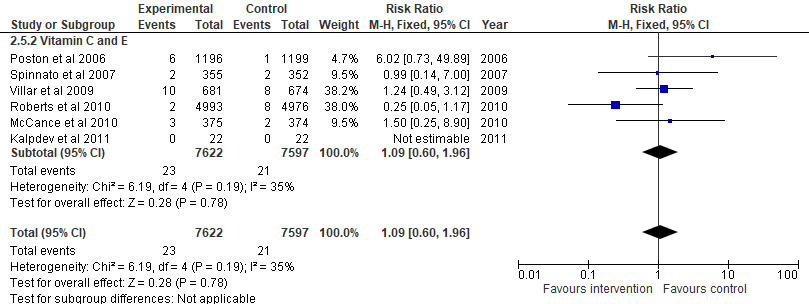
**

*no funnel plot because number of included studies <10

## 2.6 Small for gestational age

**
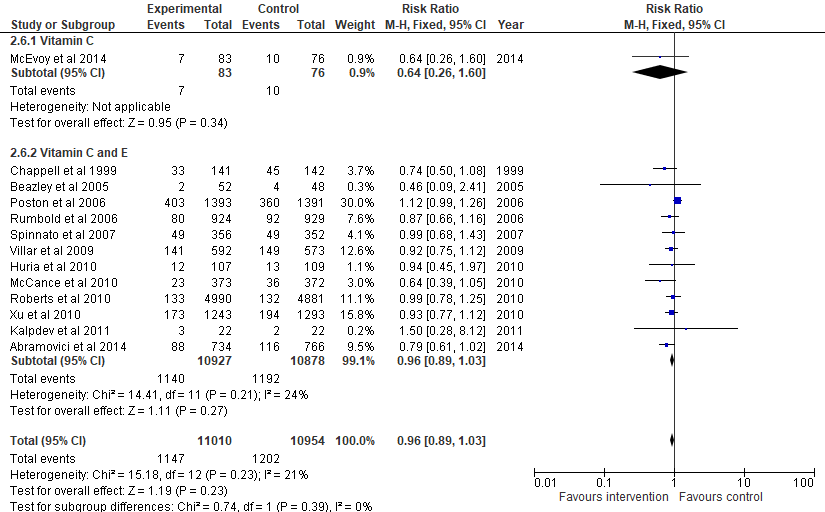
**

**
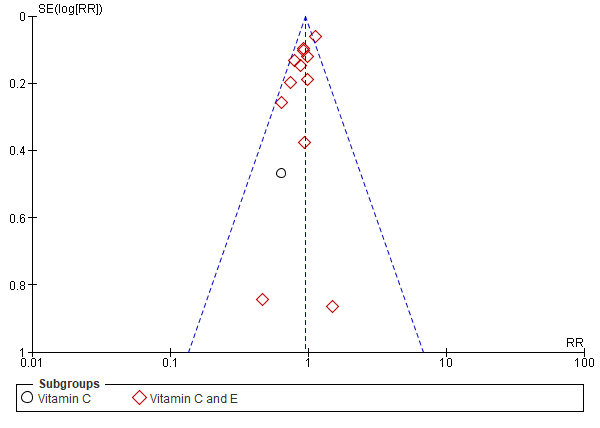
**

## 2.7 Low birthweight

**
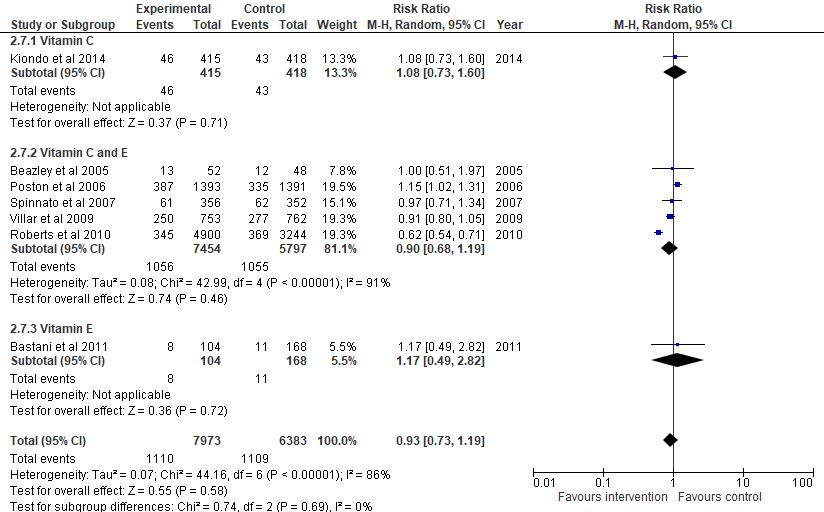
**

*no funnel plot because number of included studies <10

## 2.8 Preterm birth

**
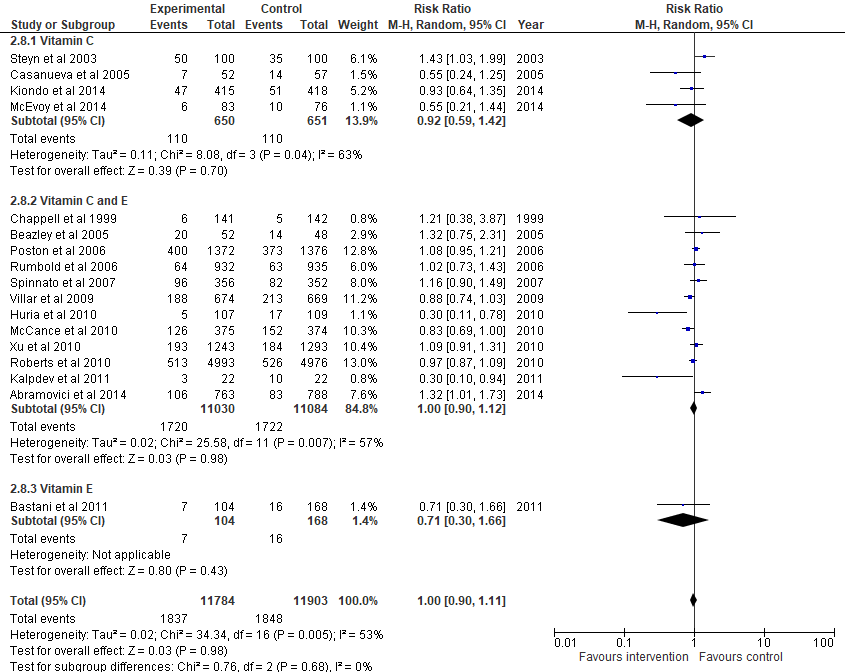
**

**
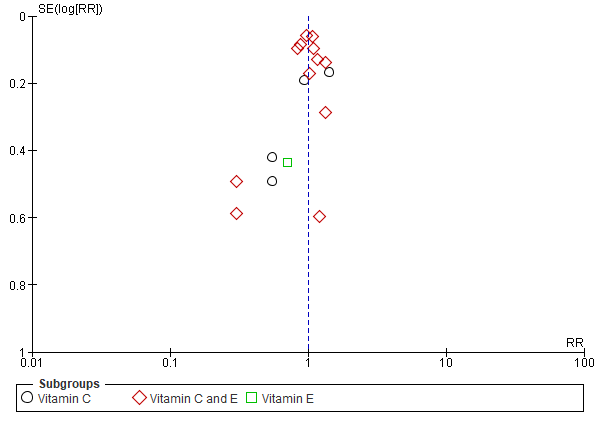
**

## 2.9 Stillbirth

**
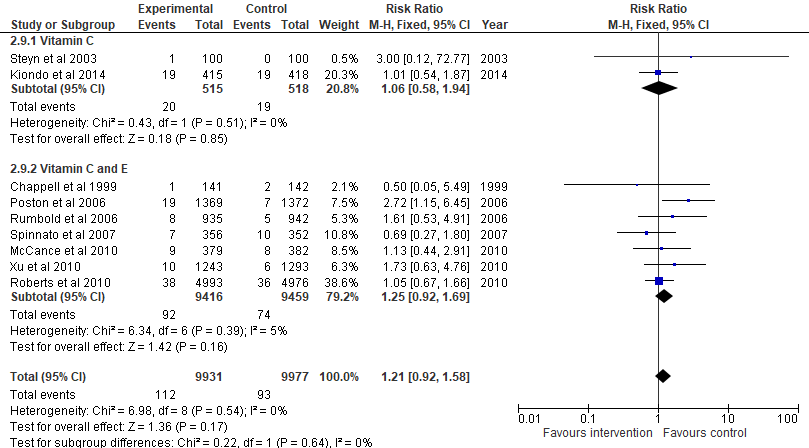
**

*no funnel plot because number of included studies <10

## 2.10 Maternal mortality

**
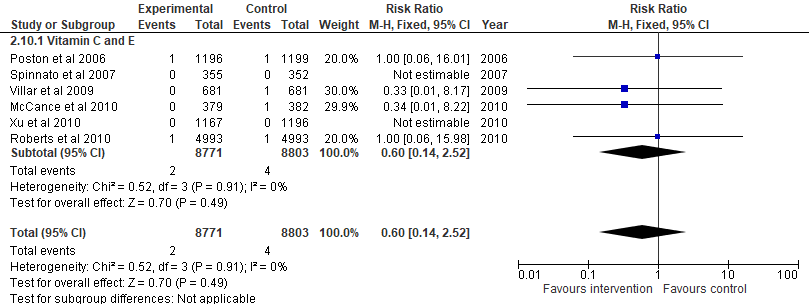
**

*no funnel plot because number of included studies <10

# 3. Vitamin D and/or calcium

## 3.1 Pre-eclampsia


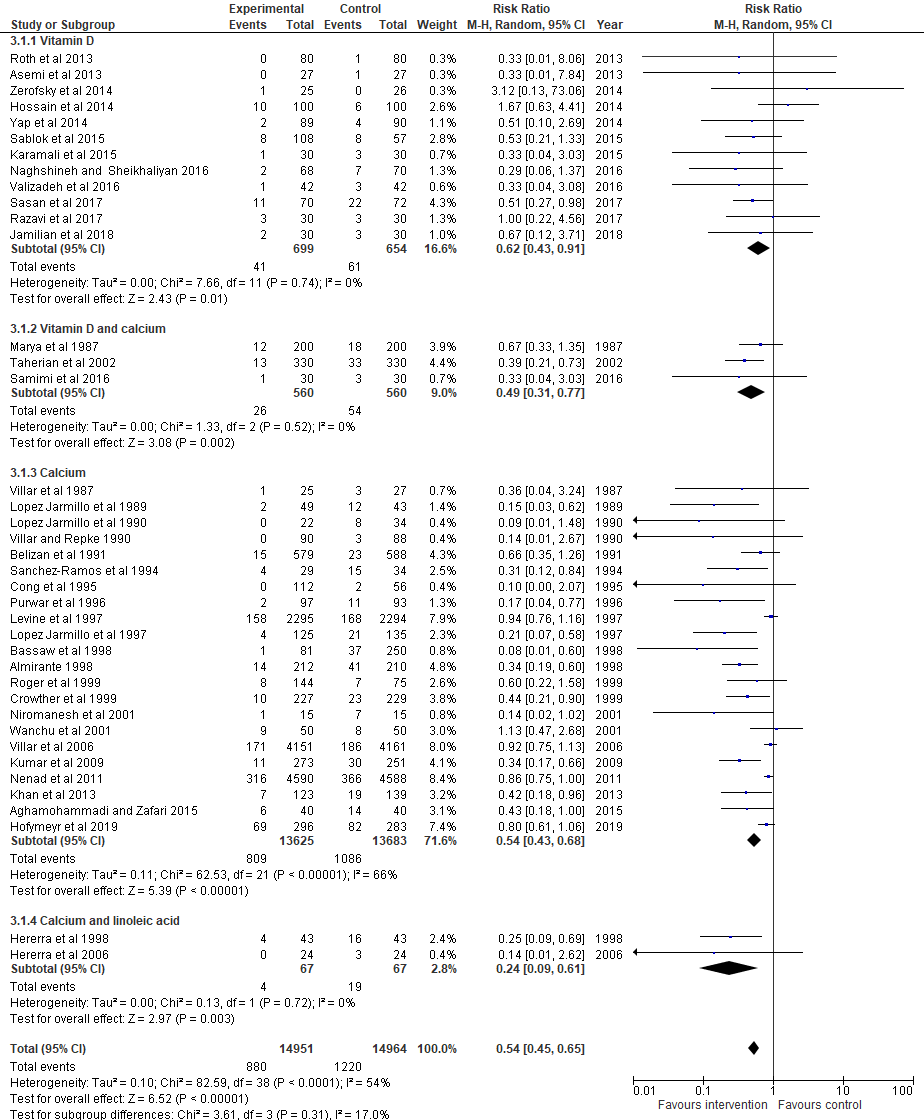


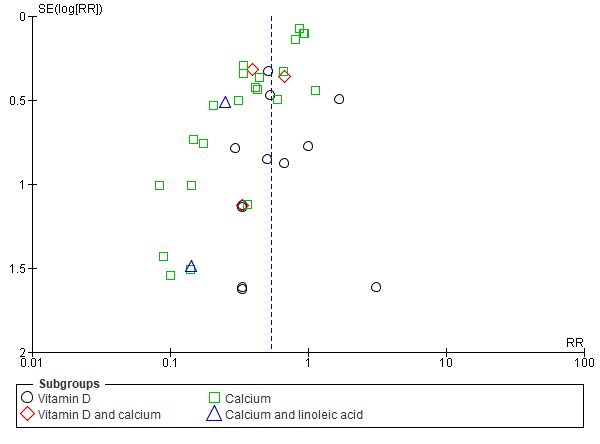


## 3.2 Severe pre-eclampsia

**
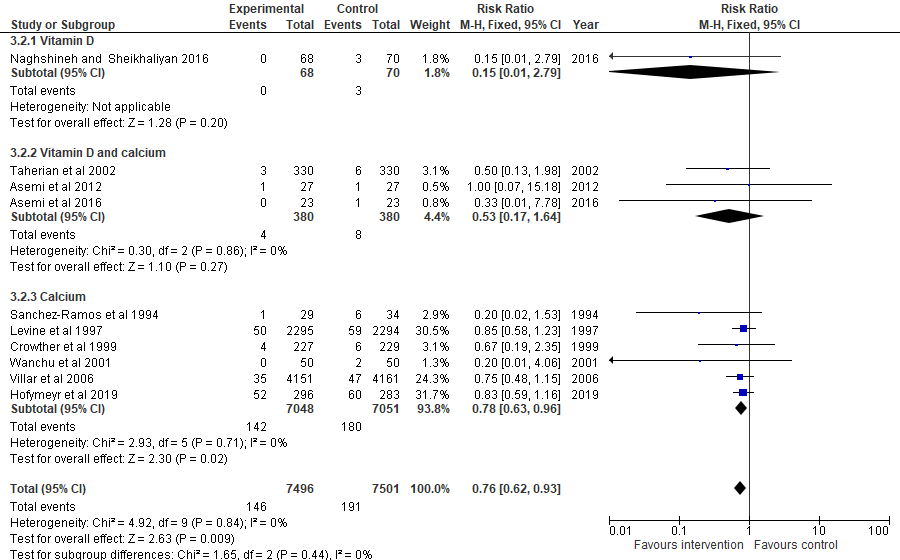
**

*no funnel plot because number of included studies for each subgroup reported (vitamin D, vitamin D and calcium, calcium) <10

## 3.3 Gestational hypertension

**
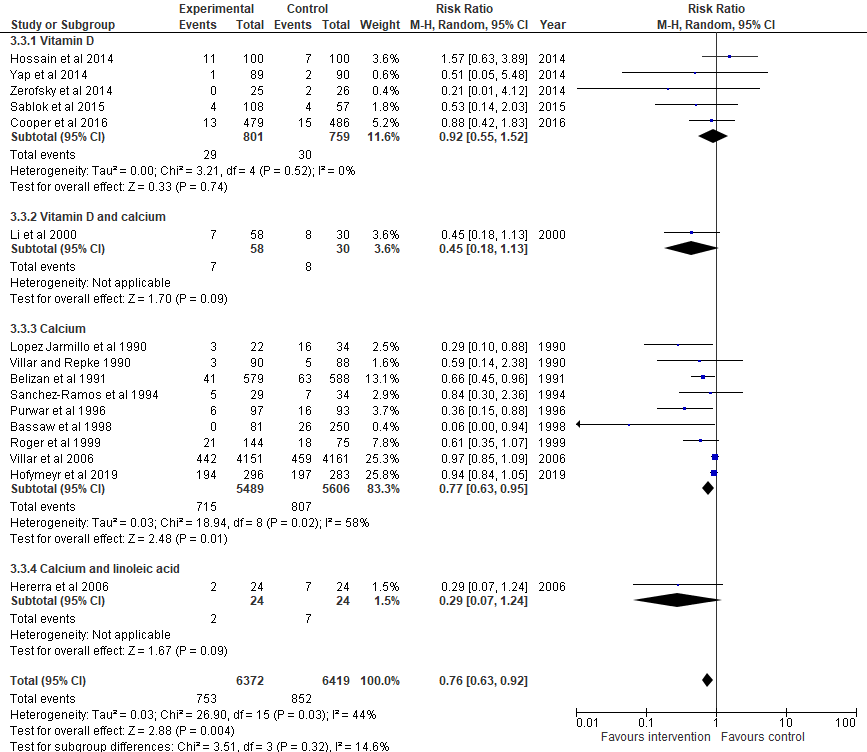
**

*no funnel plot because number of included studies for each subgroup reported (vitamin D, vitamin D and calcium, calcium) <10

## 3.4 Eclampsia

**
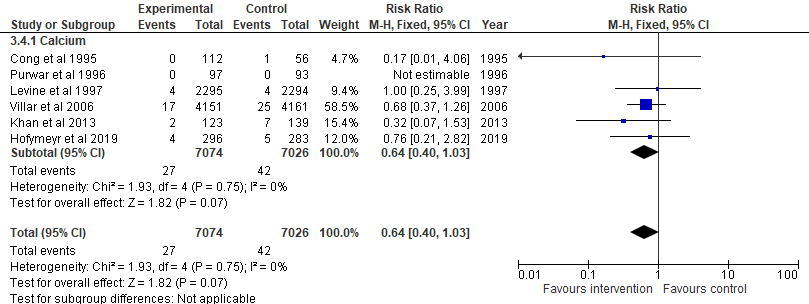
**

*no funnel plot because number of included studies for each subgroup reported (vitamin D, vitamin D and calcium, calcium) <10

## 3.5 HELLP Syndrome

**
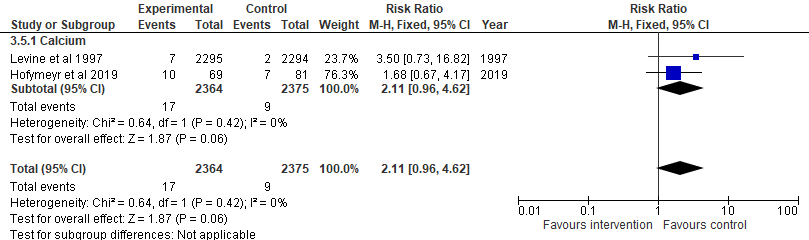
**

*no funnel plot because number of included studies for each subgroup reported (vitamin D, vitamin D and calcium, calcium) <10

## 3.6 Small for gestational age

**
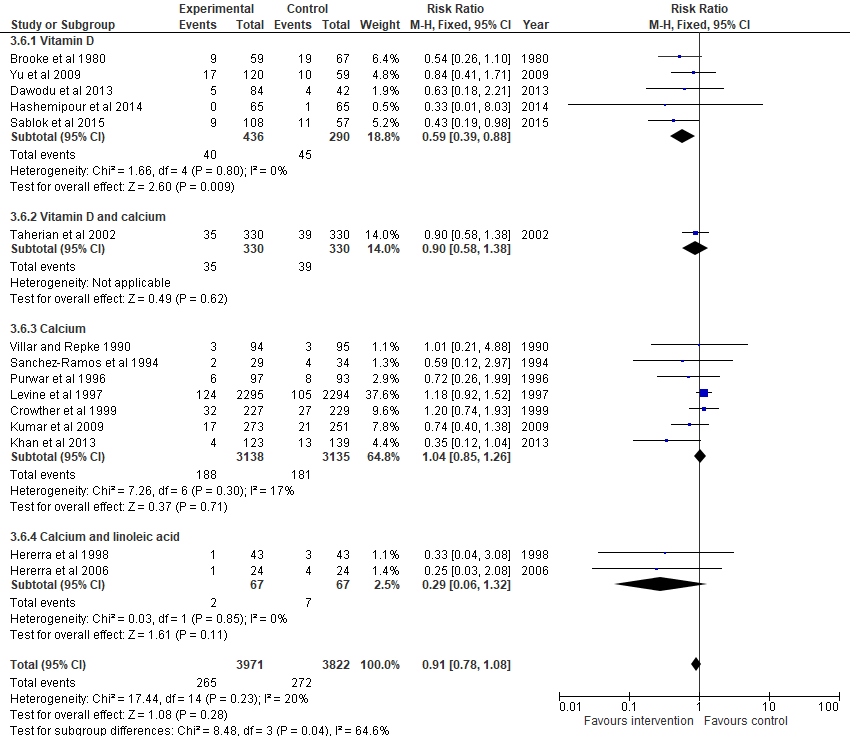
**

*no funnel plot because number of included studies for each subgroup reported (vitamin D, vitamin D and calcium, calcium) <10

## 3.7 Low birthweight

**
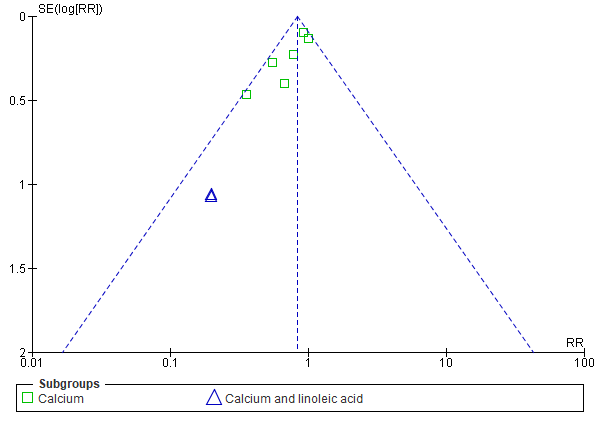

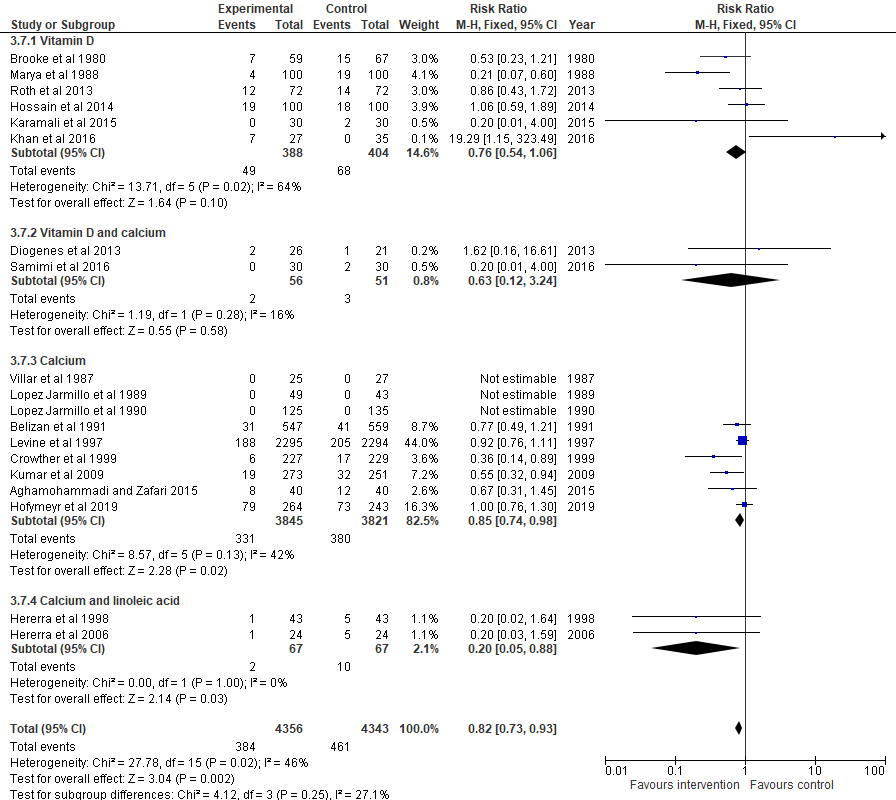
**

*no funnel plot for vitamin D and vitamin D because number of included studies for their subgroups <10

## 3.8 Preterm birth

**
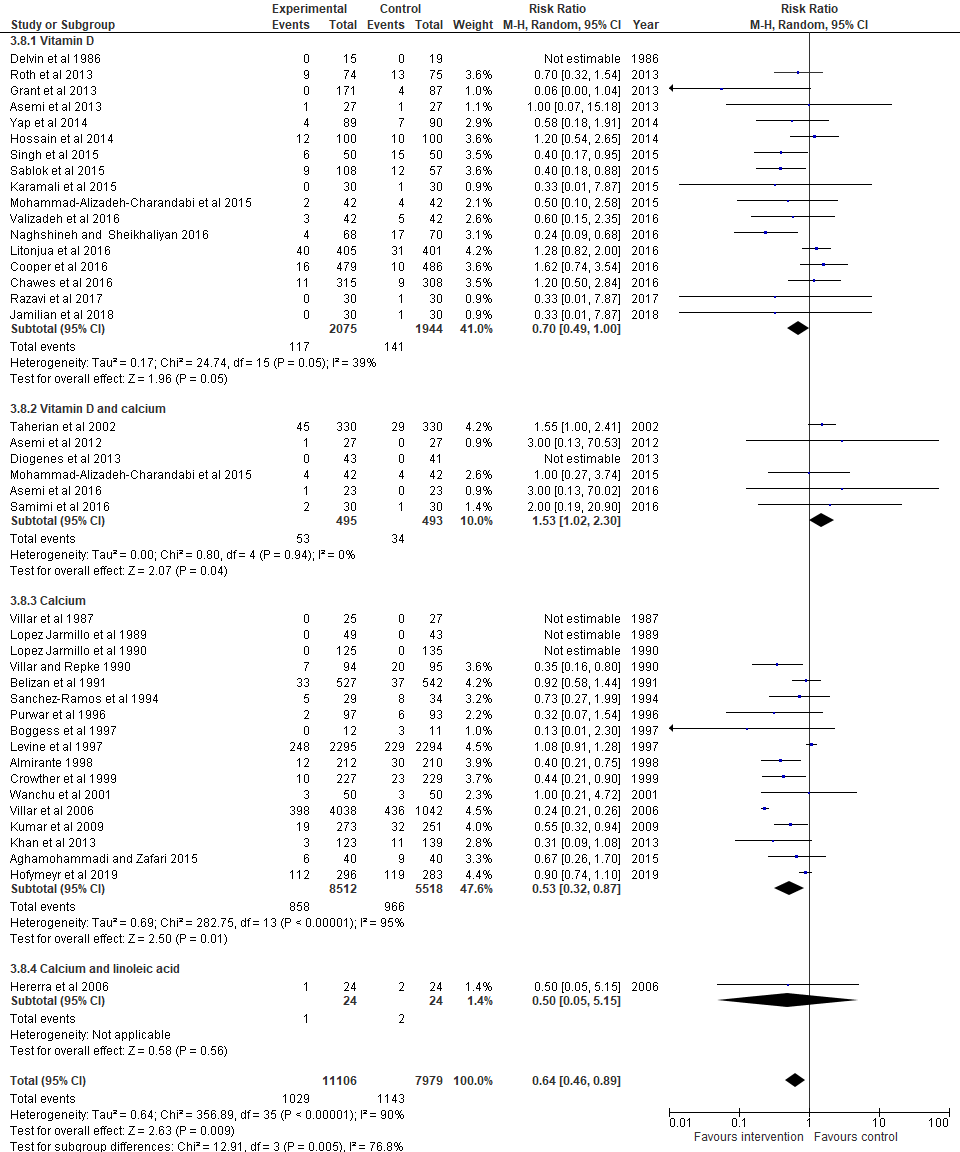
**

**
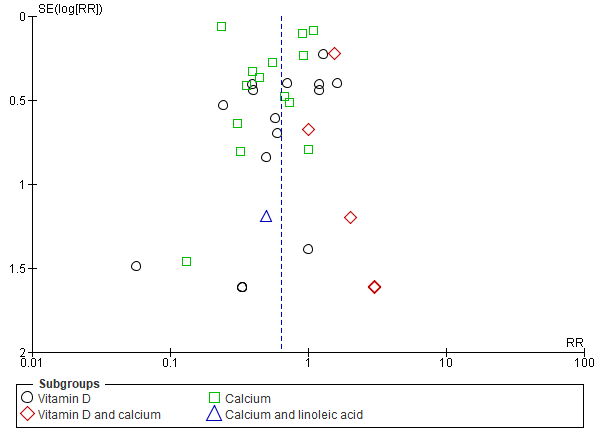
**

## 3.9 Stillbirth

**
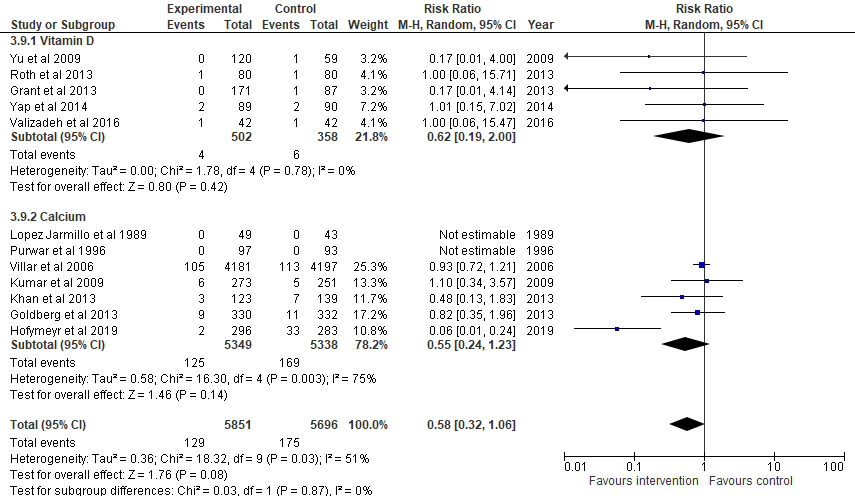
**

*no funnel plot because number of included studies for each subgroup reported (vitamin D, vitamin D and calcium, calcium) <10

## 3.10 Maternal mortality

**
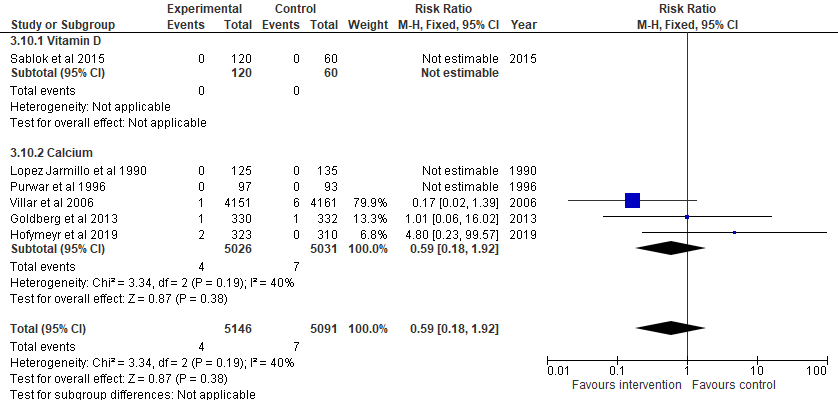
**

*no funnel plot because number of included studies for each subgroup reported (vitamin D, vitamin D and calcium, calcium) <10

# 4. Iron and/or folic acid

## 4.1 Pre-eclampsia


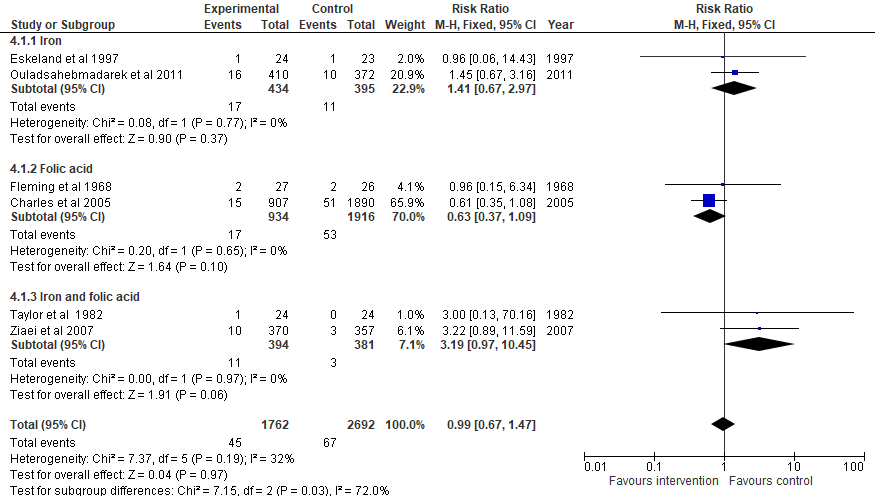


*no funnel plot because number of included studies <10

## 4.2 Eclampsia


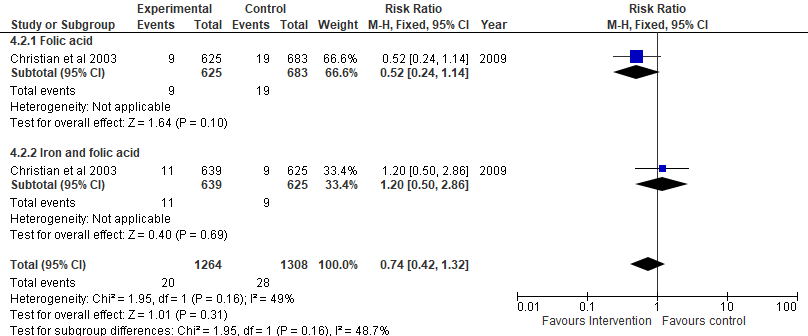


*no funnel plot because number of included studies <10

## 4.3 Small for gestational age


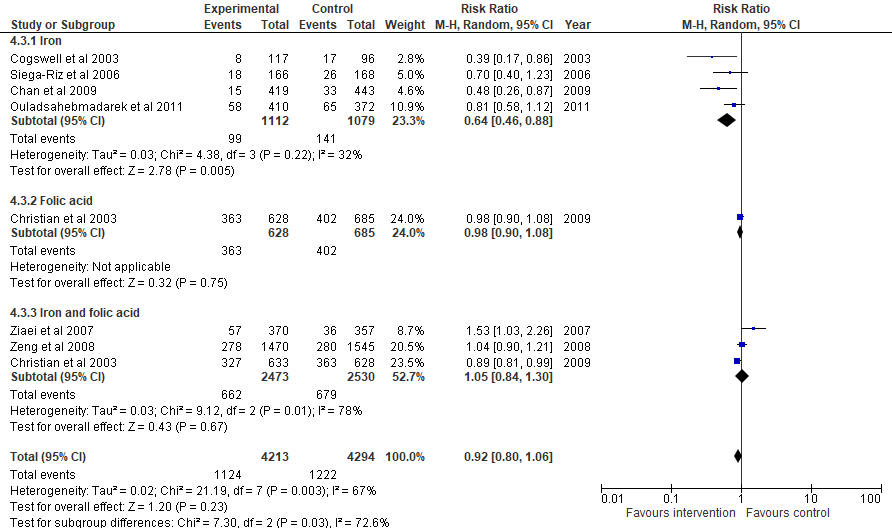


*no funnel plot because number of included studies <10

## 4.4 Low birthweight


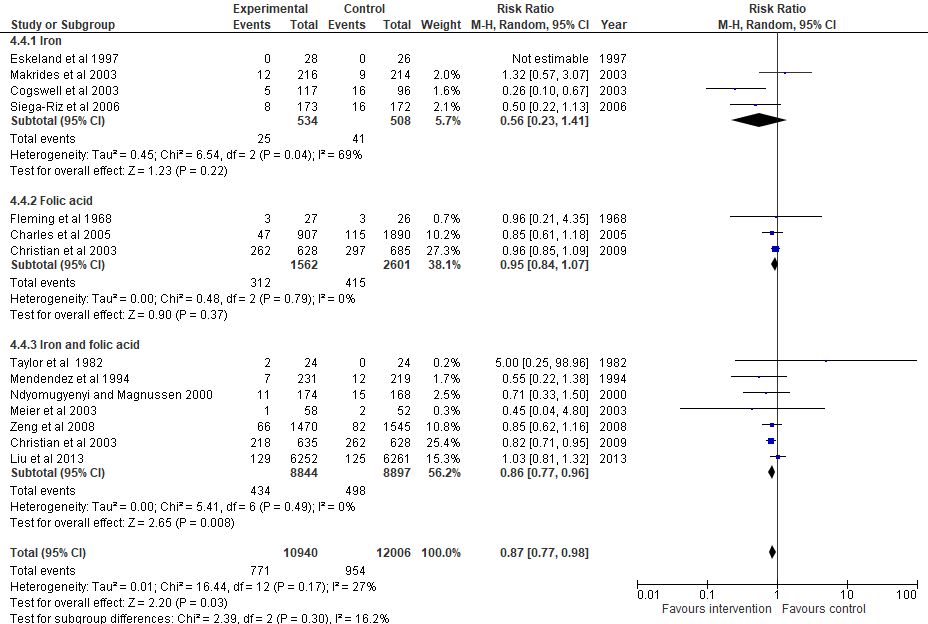


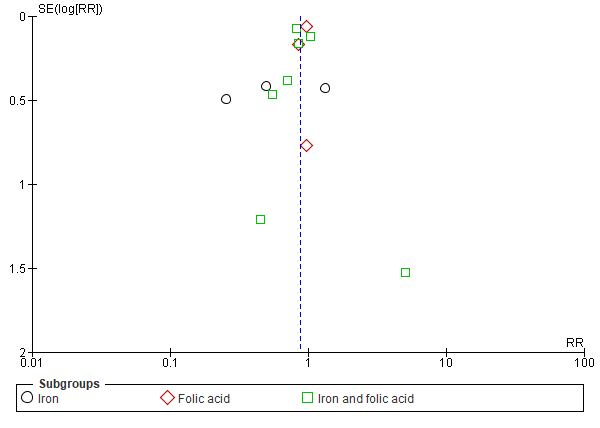


## 4.5 Preterm birth


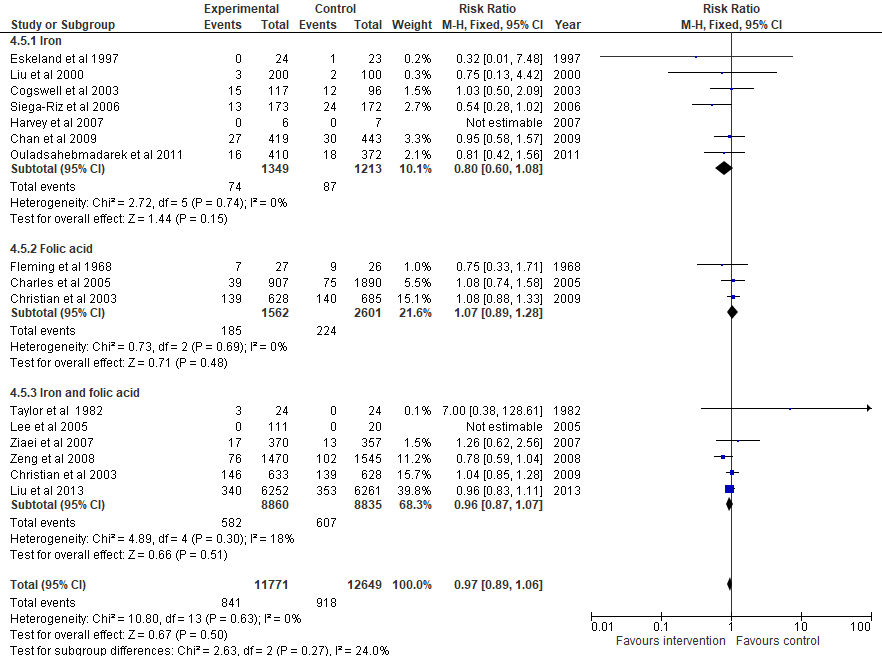


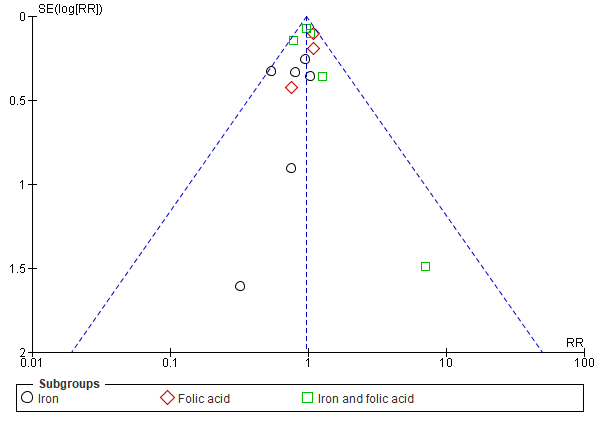


## 4.6 Stillbirth


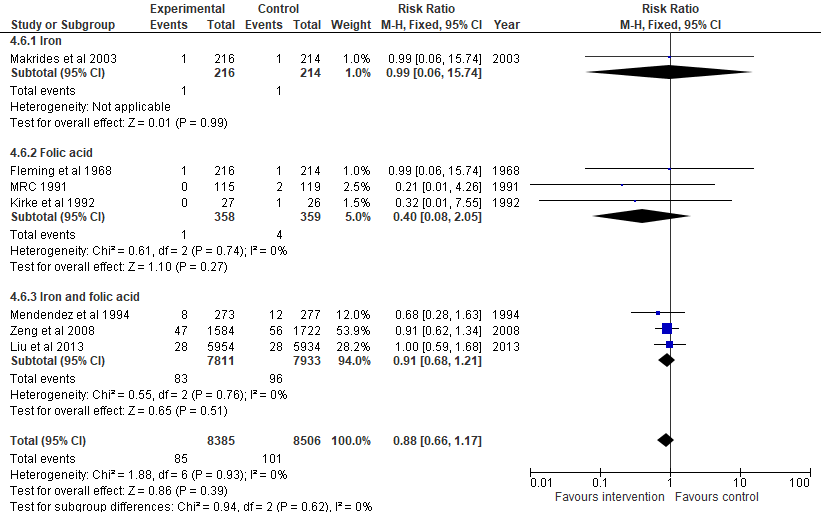


*no funnel plot because number of included studies <10

## 4.7 Maternal mortality


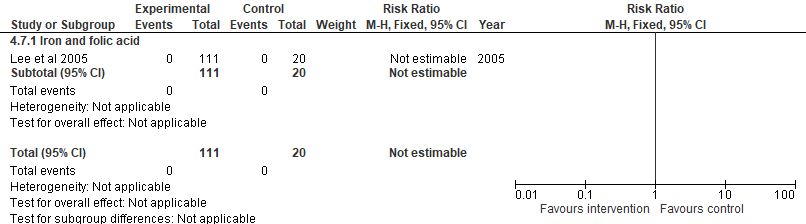


*no funnel plot because number of included studies <10

# 5. Zinc

## 5.1 Pre-eclampsia


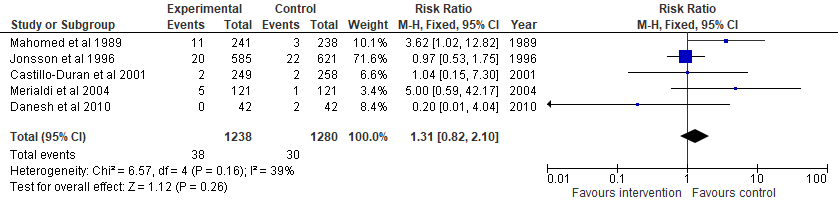
*no funnel plot because number of included studies <10

## 5.2 Gestational hypertension


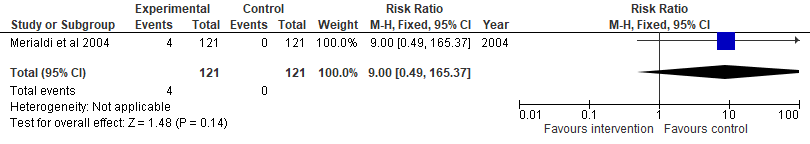
*no funnel plot because number of included studies <10

## 5.3 Small for gestational age


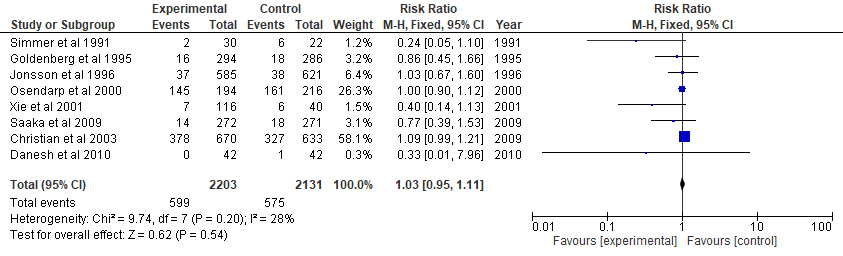
*no funnel plot because number of included studies <10

## 5.4 Low birthweight


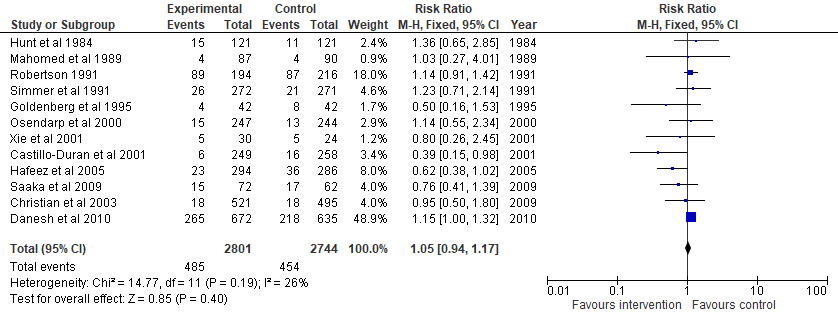


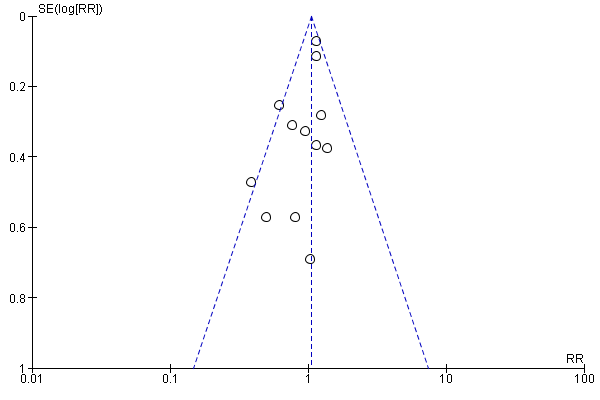


## 5.5 Preterm birth


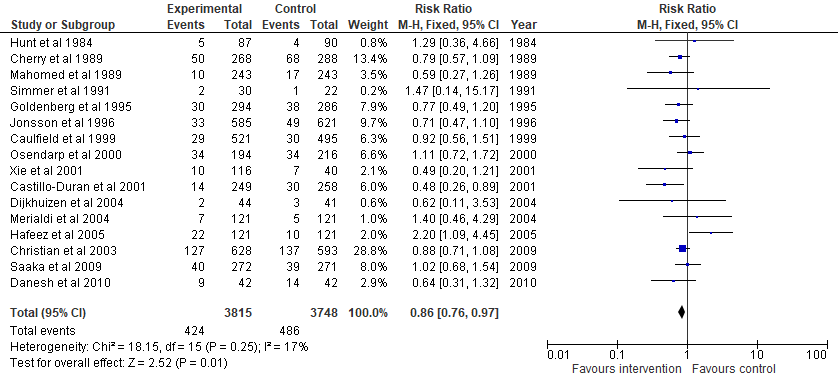


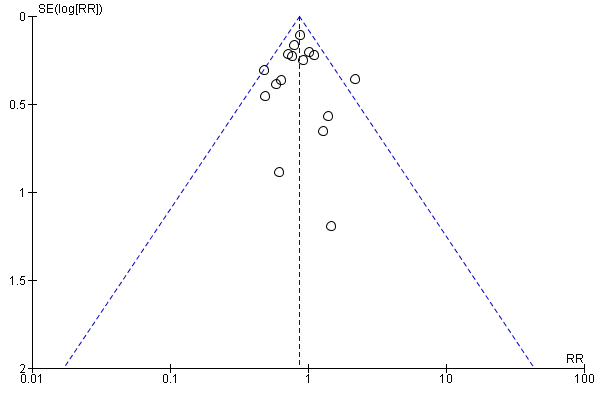


## 5.6 Stillbirth


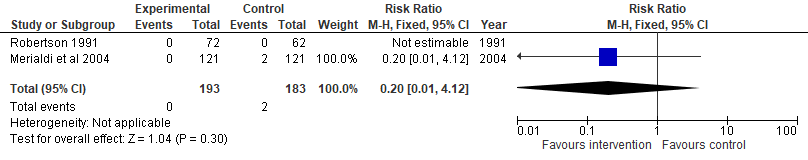
*no funnel plot because number of included studies <10

## 5.7 Maternal mortality


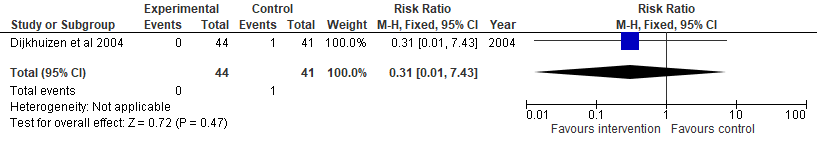
*no funnel plot because number of included studies <10

# 6. Multiple micronutrients

## 6.1 Pre-eclampsia


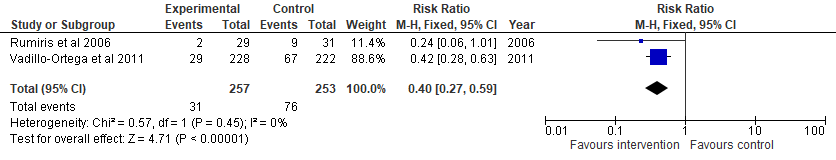
*no funnel plot because number of included studies <10

## 6.2 Severe pre-eclampsia

**
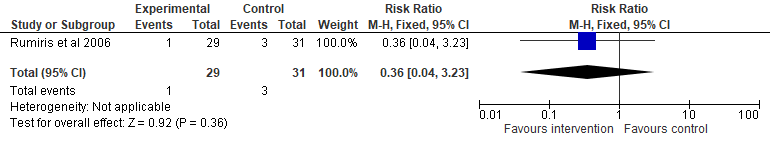
***no funnel plot because number of included studies <10

## 6.3 Gestational hypertension

**
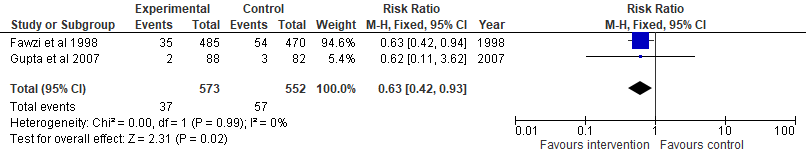
***no funnel plot because number of included studies <10

## 6.4 Eclampsia

**
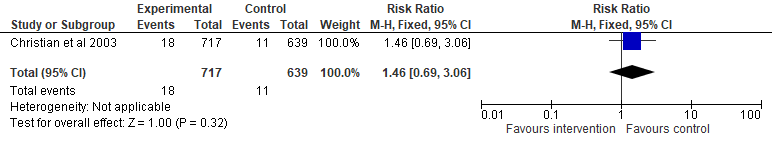
***no funnel plot because number of included studies <10

## 6.5 Small for gestational age


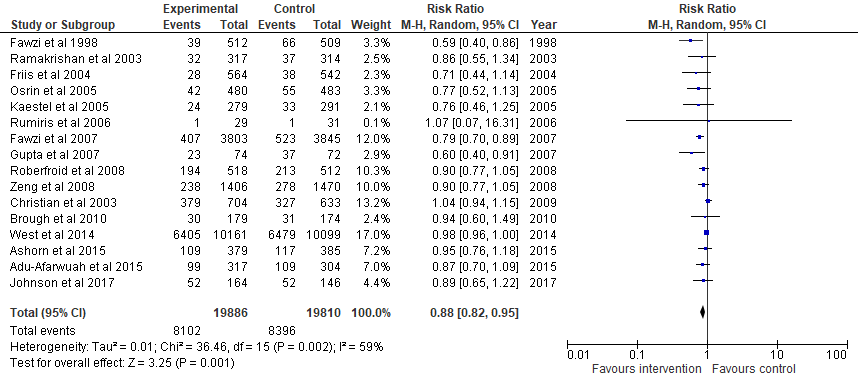


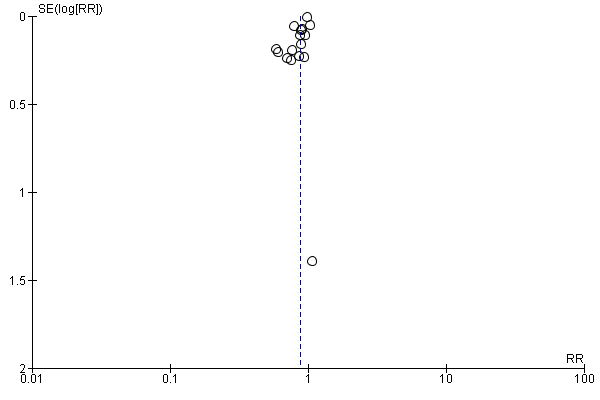


## 6.6 Low birthweight


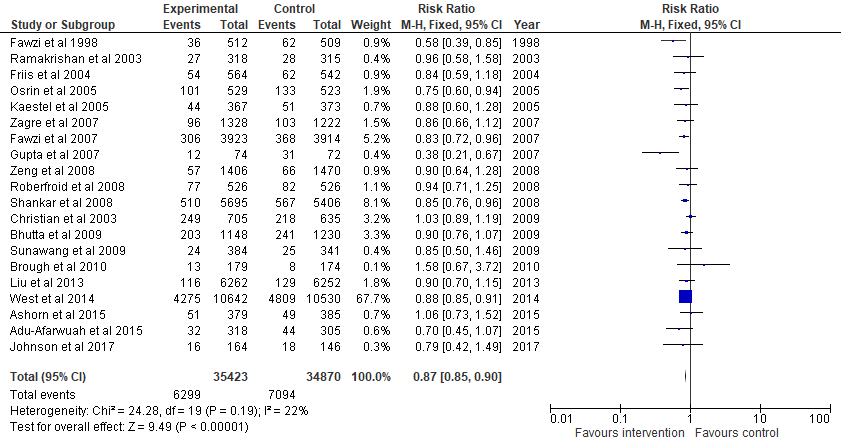


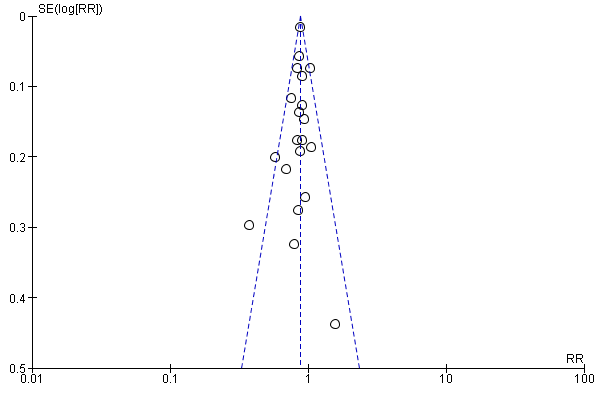


## 6.7 Preterm birth


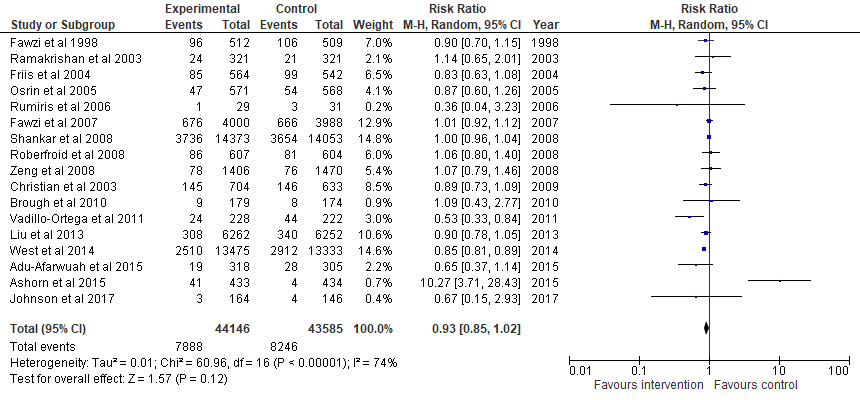


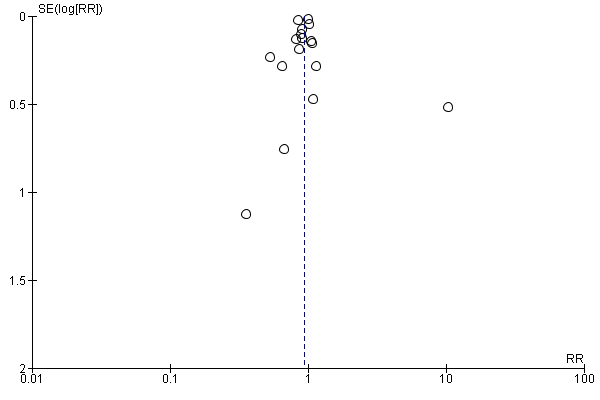


## 6.8 Stillbirth


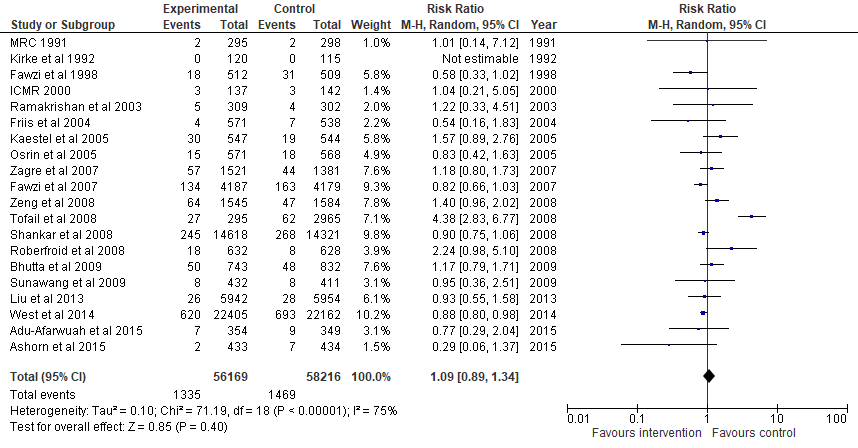


s
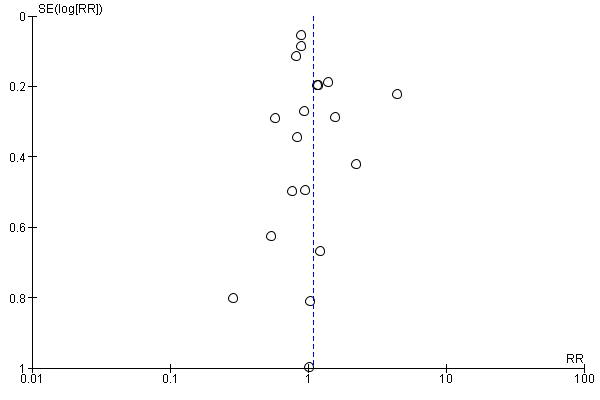


## 6.9 Maternal mortality


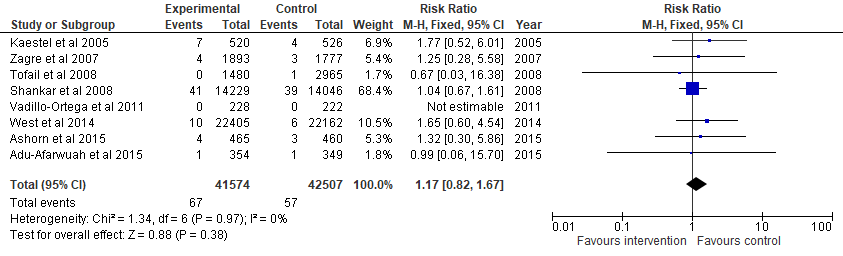
*no funnel plot because number of included studies <10

# 7. Lipid-based nutrients

## 7.1 Small for gestational age

**
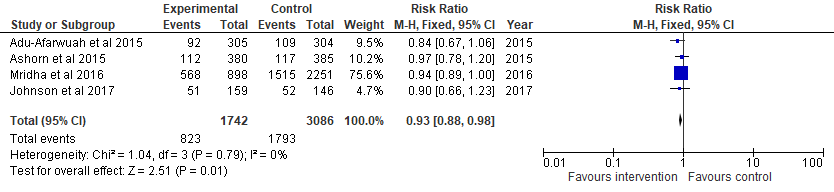
**

*no funnel plot because number of included studies <10

## 7.2 Low birthweight

**
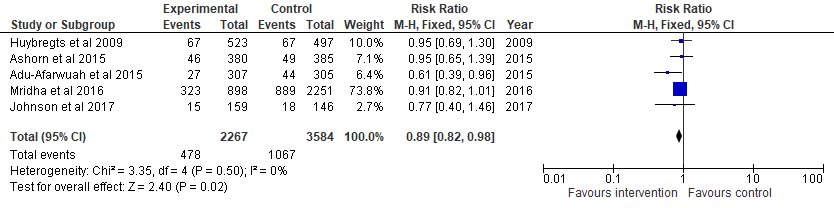
***no funnel plot because number of included studies <10

## 7.3 Preterm birth

**
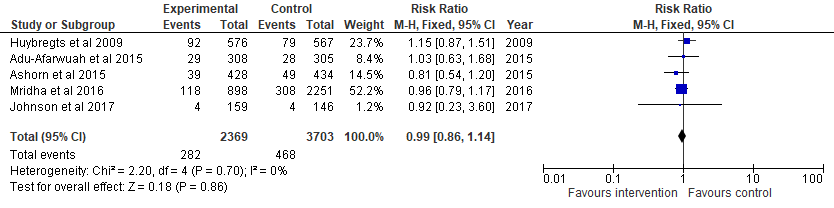
***no funnel plot because number of included studies <10

## 7.4 Stillbirth

**
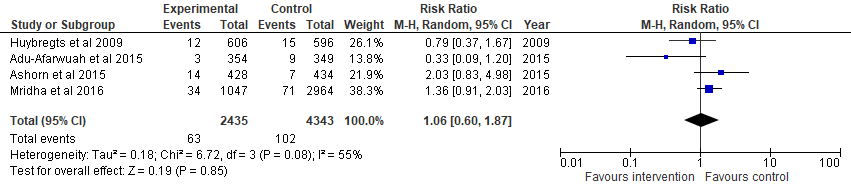
***no funnel plot because number of included studies <10

## 7.5 Maternal mortality


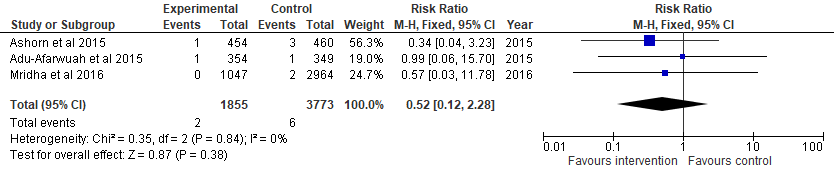
*no funnel plot because number of included studies <10

# 8. Polyunsaturated omega-3 fatty acid

## 8.1 Pre-eclampsia

**
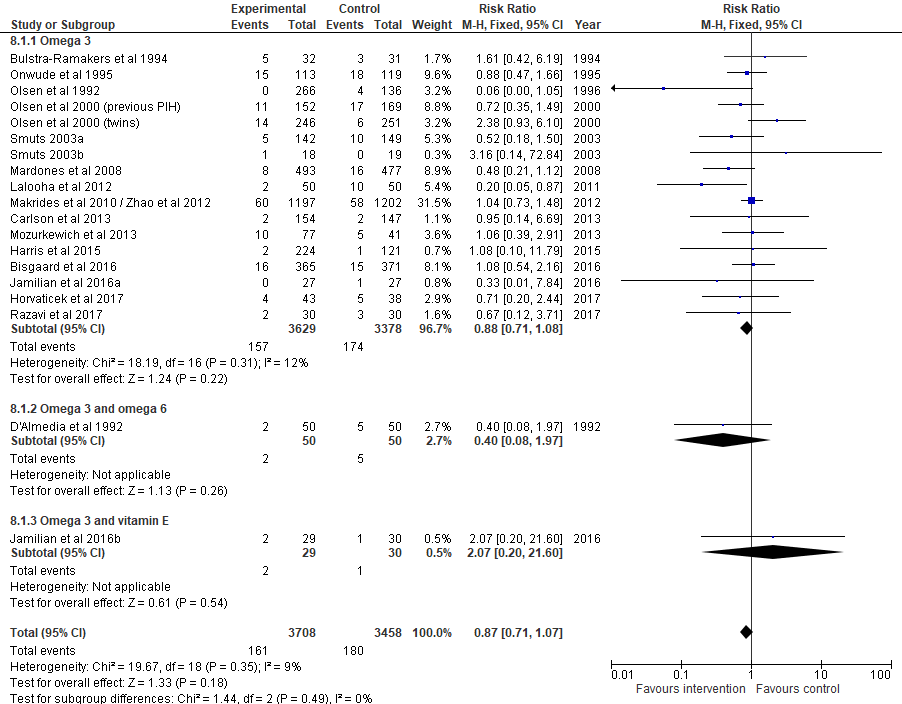
**

**
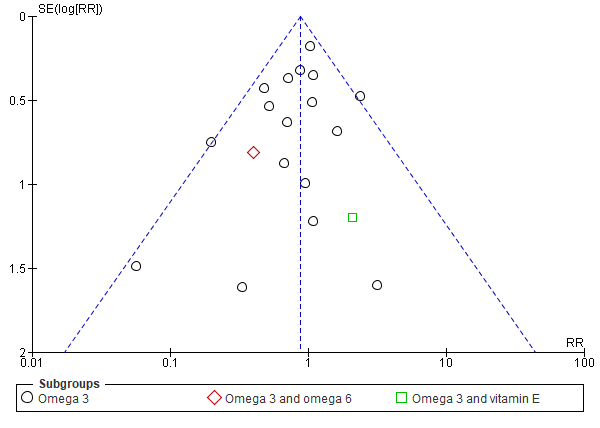
**

## 8.2 Severe pre-eclampsia

**
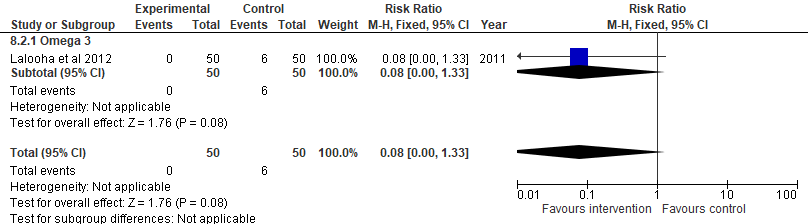
**

*no funnel plot because number of included studies <10

## 8.3 Gestational hypertension

**
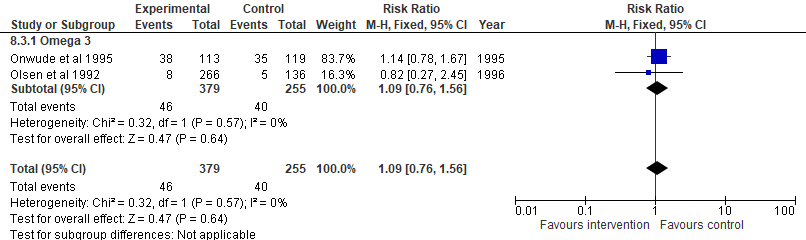
**

*no funnel plot because number of included studies <10

## 8.4 Eclampsia

**
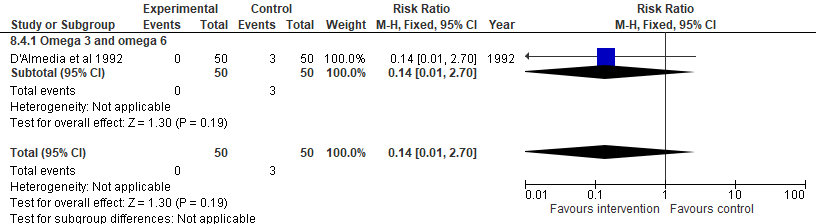
**

*no funnel plot because number of included studies <10

## 8.5 Small for gestational age

**
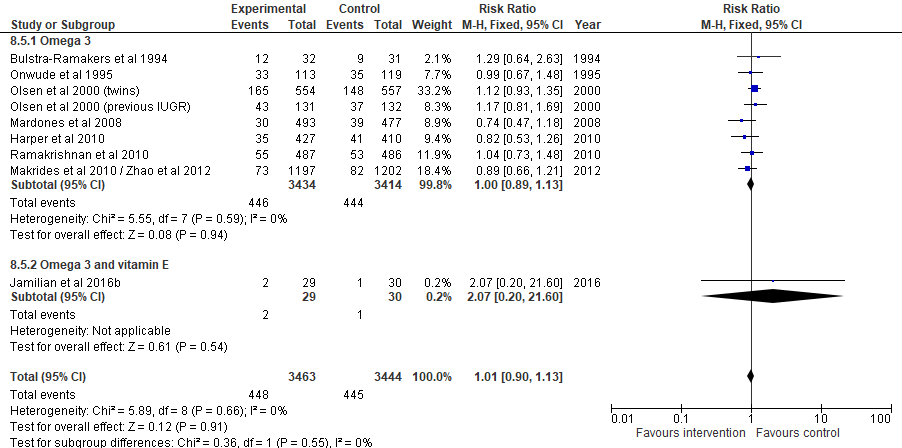
**

*no funnel plot because number of included studies <10

## 8.6 Low birthweight


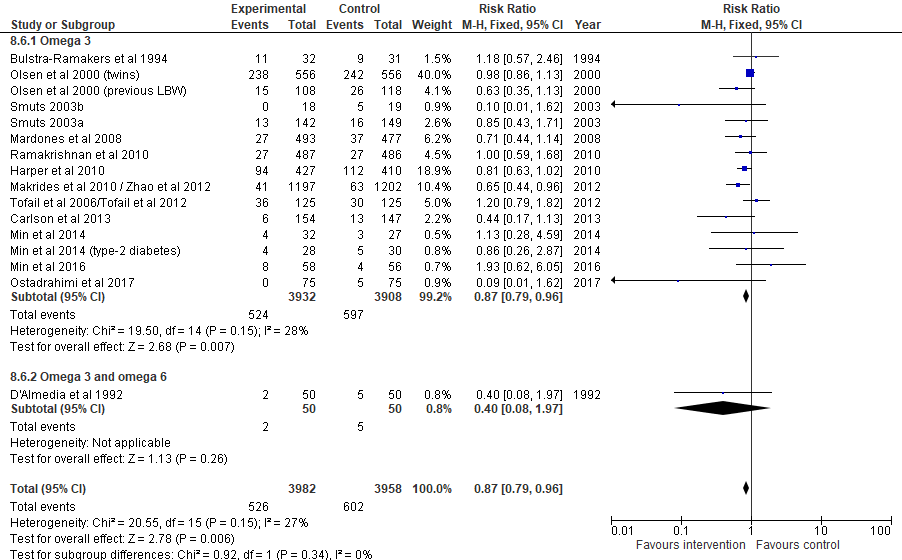


**
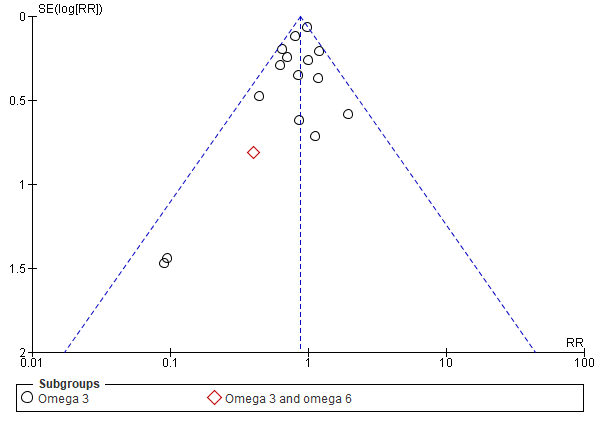
**

## 8.7 Preterm birth

**
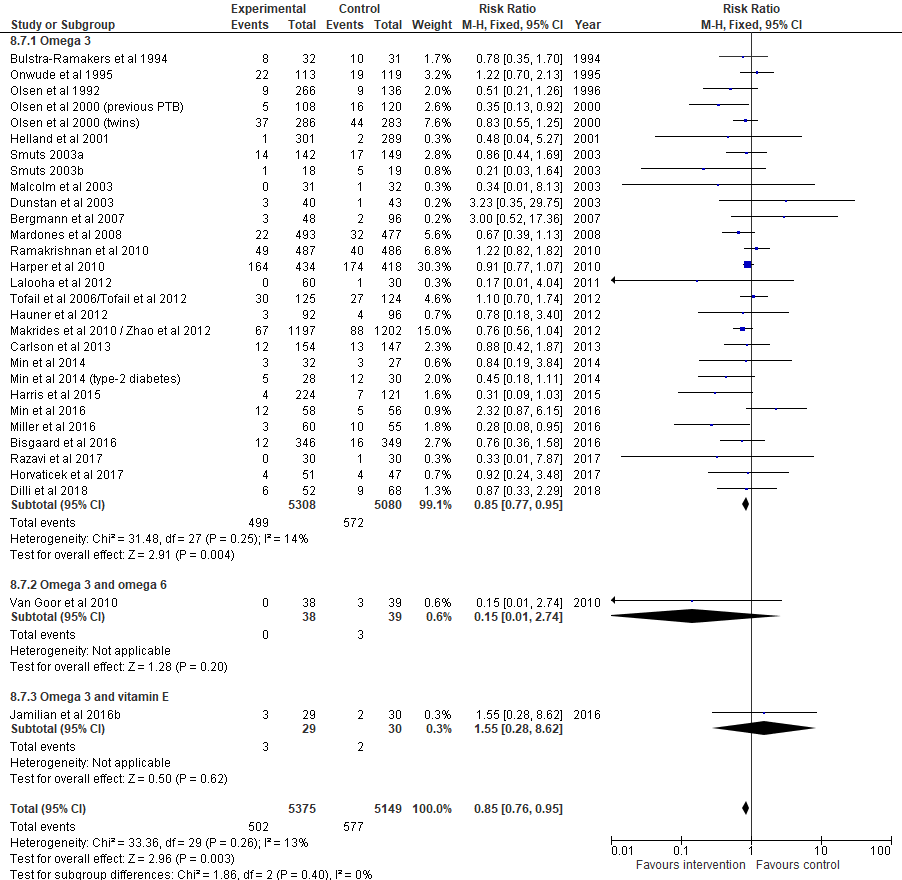
**

**
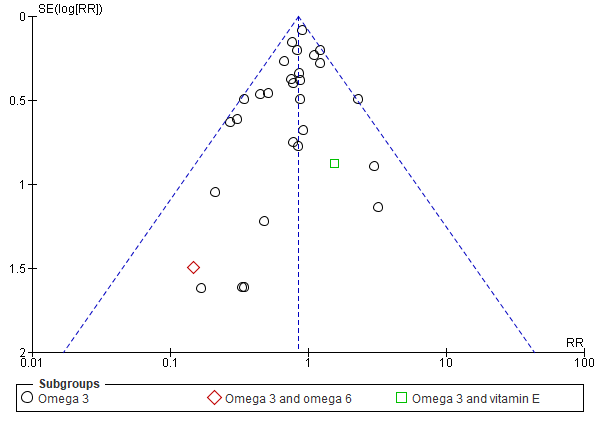
**

## 8.8 Stillbirth

**
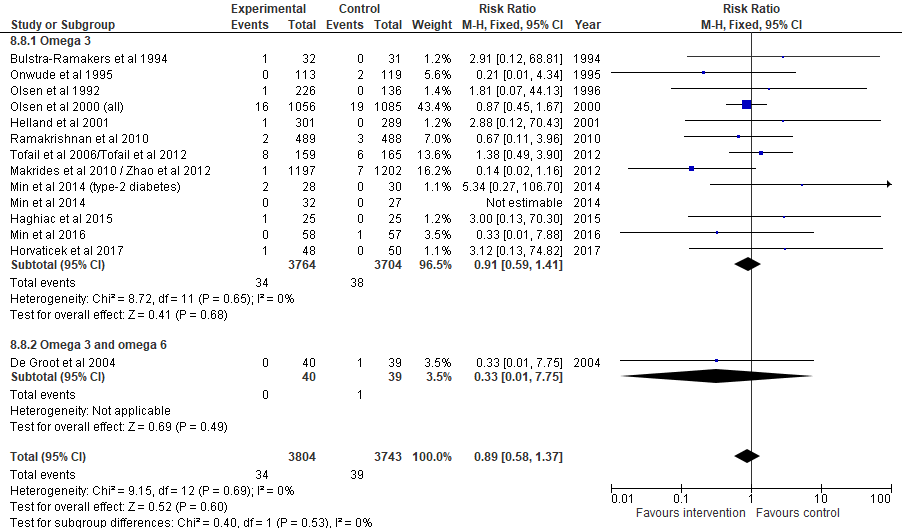
**

**
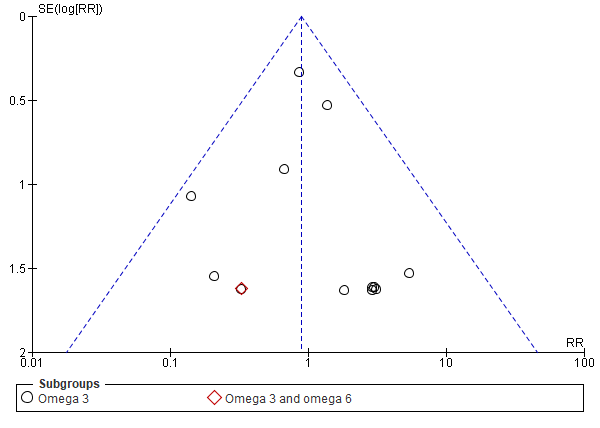
**

## 8.9 Maternal mortality


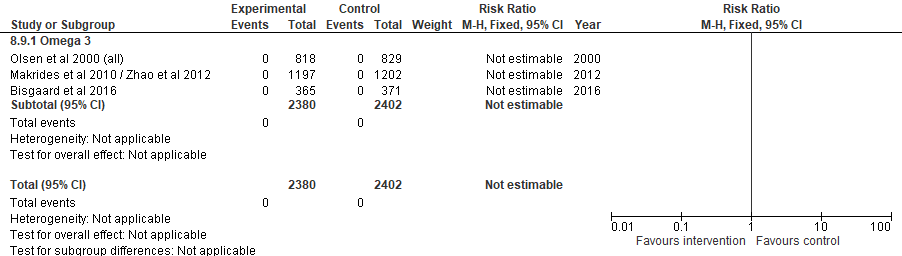


*no funnel plot because number of included studies <10

# 9. Antenatal dietary counselling with or without physical activity promotion

## 9.1 Pre-eclampsia


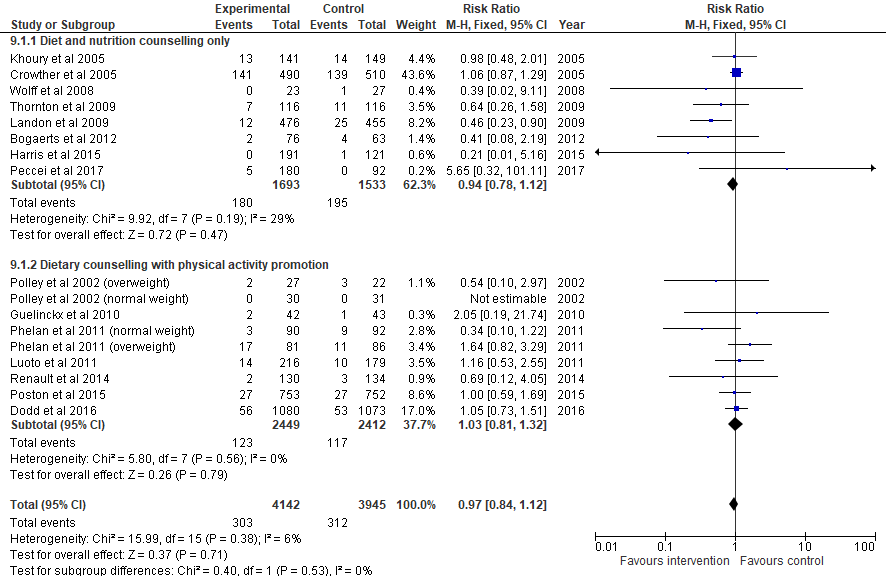


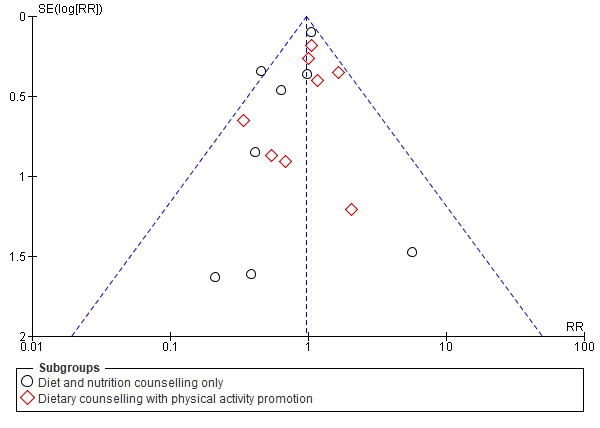


## 9.2 Severe pre-eclampsia


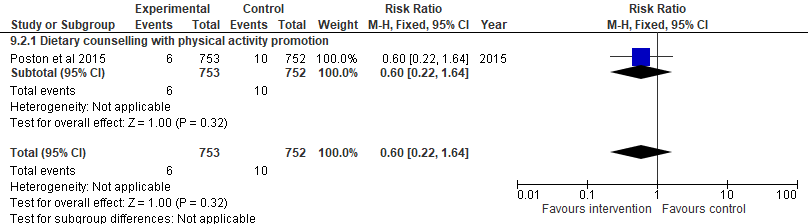
*no funnel plot because number of included studies <10

## 9.3 Gestational hypertension

**
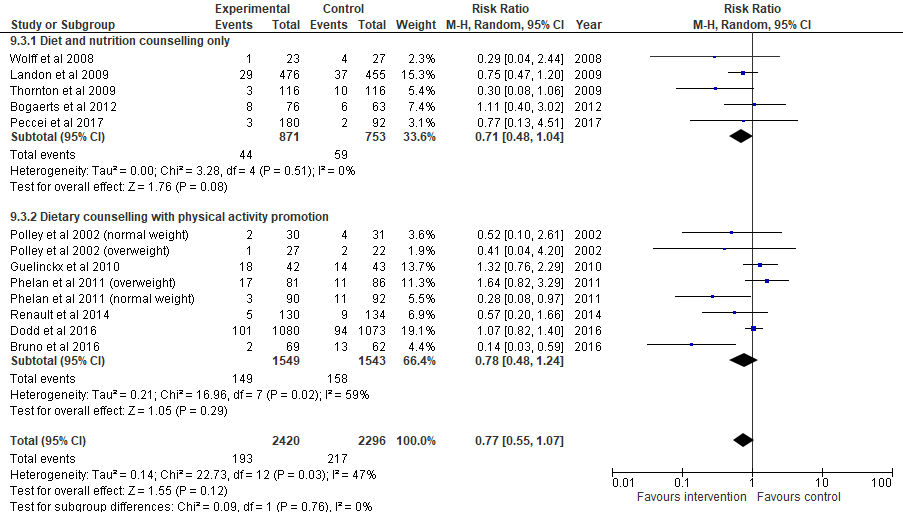
**

**
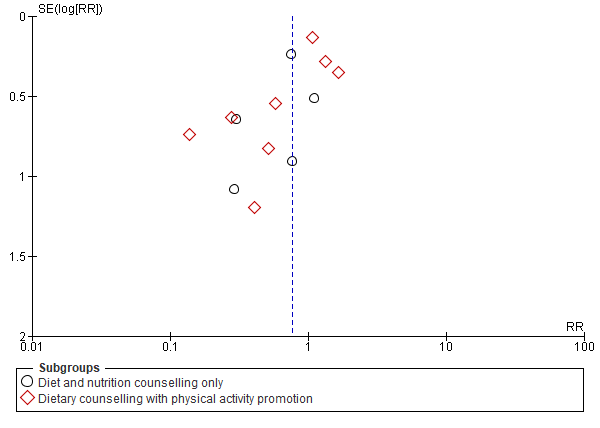
**

## 9.4 Small for gestational age

**
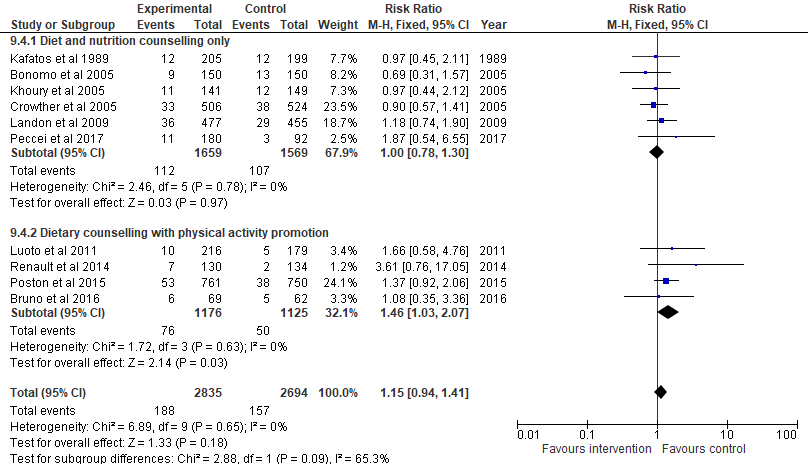
**

**
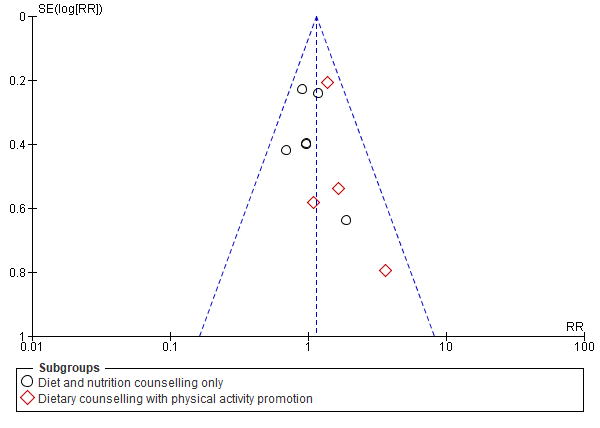
**

## 9.5 Low birthweight

**
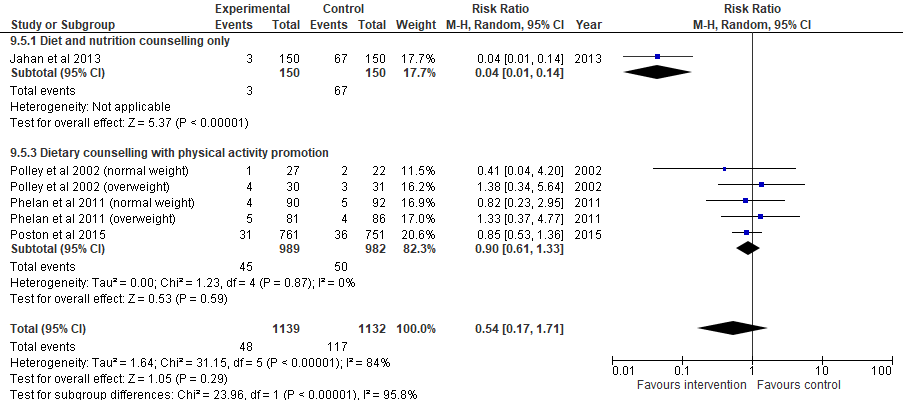
**

*no funnel plot because number of included studies <10

## 9.6 Preterm birth

**
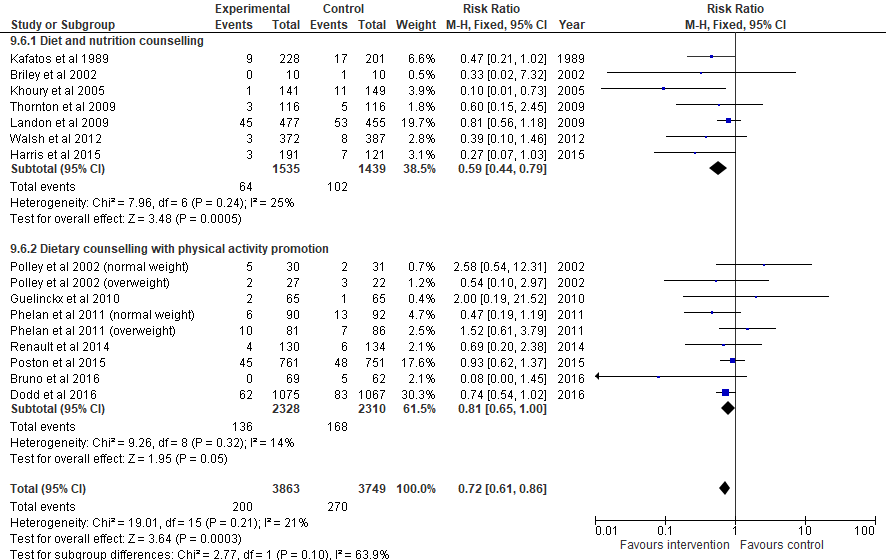
**

**
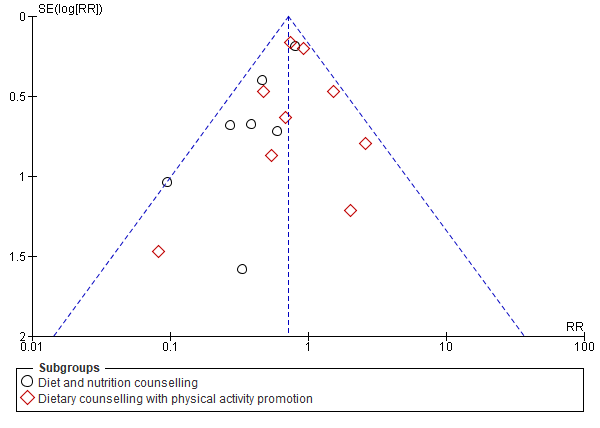
**

## 9.7 Stillbirth

**
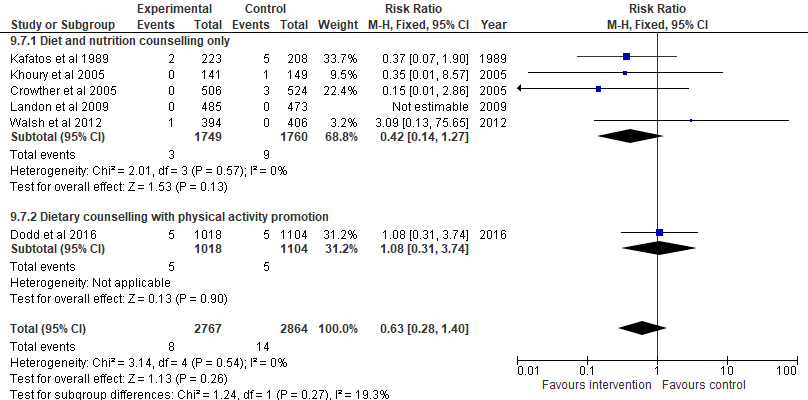
**

*no funnel plot because number of included studies <10

## 9.8 Maternal mortality

**
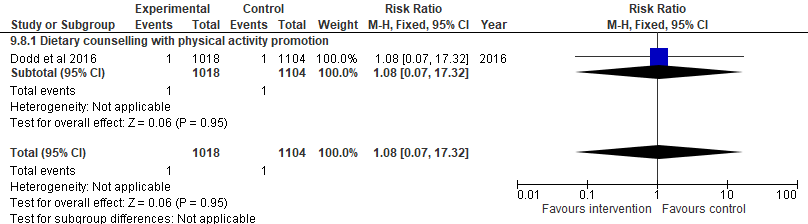
**

*no funnel plot because number of included studies <10
