# Supplementary material for: Effects of Maternal Nutritional Supplements and Dietary Interventions on Placental Complications: An Umbrella Review, Meta-Analysis and Evidence Map
Source: Nutrients. 2021 Jan 30;13(2):472. doi: 10.3390/nu13020472 (PMC7912620; doi:10.3390/nu13020472)
Supplement: Supplementary file 1 [file nutrients-13-00472-s001.zip › Supplementary files/Figures S2 - Sensitivity analyses.docx]

**Figures S2 - Sensitivity analyses**

Contents

[1. Vitamin A 5](#_Toc55379166)

[1.1 Studies with low/unclear risk of bias only 5](#_Toc55379167)

[10.1.2 LMIC studies only 5](#_Toc55379168)

[2. Vitamin C and/or E 5](#_Toc55379169)

[2.1 Studies with low/unclear risk of bias only 5](#_Toc55379170)

[2.1.1 Pre-eclampsia 5](#_Toc55379171)

[2.1.2 Small for gestational age 6](#_Toc55379172)

[2.1.3 Low birthweight 6](#_Toc55379173)

[2.1.4 Preterm birth 7](#_Toc55379174)

[2.1.5 Stillbirth 7](#_Toc55379175)

[2.1.6 Maternal mortality 7](#_Toc55379176)

[2.2 LMIC studies only 8](#_Toc55379177)

[2.2.1 Pre-eclampsia 8](#_Toc55379178)

[2.2.2 Small for gestational age 9](#_Toc55379179)

[2.2.3 Low birthweight 9](#_Toc55379180)

[2.2.4 Preterm birth 10](#_Toc55379181)

[2.2.5 Stillbirth 11](#_Toc55379182)

[2.2.6 Maternal mortality 11](#_Toc55379183)

[3. Vitamin D and/or calcium 12](#_Toc55379184)

[3.1 Studies with low/unclear risk of bias only 12](#_Toc55379185)

[3.1.1 Pre-eclampsia 12](#_Toc55379186)

[3.1.2 Small for gestational age 13](#_Toc55379187)

[3.1.3 Low birthweight 14](#_Toc55379188)

[3.1.4 Preterm birth 15](#_Toc55379189)

[3.1.5 Stillbirth 16](#_Toc55379190)

[3.1.6 Maternal mortality 16](#_Toc55379191)

[3.2 LMIC studies only 17](#_Toc55379192)

[3.2.1 Pre-eclampsia 17](#_Toc55379193)

[3.2.2 Small for gestational age 18](#_Toc55379194)

[3.2.3 Low birthweight 19](#_Toc55379195)

[3.2.4 Preterm birth 20](#_Toc55379196)

[3.2.5 Stillbirth 20](#_Toc55379197)

[3.2.6 Maternal mortality 21](#_Toc55379198)

[4. Iron and/or folic acid 21](#_Toc55379199)

[4.1 Studies with low/unclear risk of bias only 21](#_Toc55379200)

[4.1.1 Pre-eclampsia 21](#_Toc55379201)

[4.1.2 Small for gestational age 22](#_Toc55379202)

[4.1.3 Low birthweight 22](#_Toc55379203)

[4.1.4 Preterm birth 23](#_Toc55379204)

[4.1.5 Stillbirths 24](#_Toc55379205)

[4.1.6 Maternal mortality 24](#_Toc55379206)

[4.2 LMIC studies only 25](#_Toc55379207)

[4.2.1 Pre-eclampsia 25](#_Toc55379208)

[4.2.2 Small for gestational age 25](#_Toc55379209)

[4.2.3 Low birthweight 26](#_Toc55379210)

[4.2.4 Preterm birth 27](#_Toc55379211)

[4.2.5 Stillbirth 28](#_Toc55379212)

[4.2.6 Maternal mortality 28](#_Toc55379213)

[5. Zinc 28](#_Toc55379214)

[5.1 Studies with low/unclear risk of bias only 28](#_Toc55379215)

[5.1.1 Pre-eclampsia 28](#_Toc55379216)

[5.1.2 Small for gestational age 29](#_Toc55379217)

[5.1.3 Low birthweight 29](#_Toc55379218)

[5.1.4 Preterm birth 29](#_Toc55379219)

[5.1.5 Stillbirth 29](#_Toc55379220)

[5.1.6 Maternal mortality 30](#_Toc55379221)

[5.2 LMIC studies only 30](#_Toc55379222)

[5.2.1 Pre-eclampsia 30](#_Toc55379223)

[5.2.2 Small for gestational age 30](#_Toc55379224)

[5.2.3 Low birthweight 30](#_Toc55379225)

[5.2.4 Preterm birth 31](#_Toc55379226)

[5.2.5 Stillbirth 31](#_Toc55379227)

[5.2.6 Maternal mortality 31](#_Toc55379228)

[6. Multiple micronutrients 31](#_Toc55379229)

[6.1 Studies with low/unclear risk of bias only 31](#_Toc55379230)

[6.1.1 Pre-eclampsia 31](#_Toc55379231)

[6.1.2 Small for gestational age 32](#_Toc55379232)

[6.1.3 Low birthweight 32](#_Toc55379233)

[6.1.4 Preterm birth 33](#_Toc55379234)

[6.1.5 Stillbirth 33](#_Toc55379235)

[6.1.6 Maternal mortality 34](#_Toc55379236)

[6.2 LMIC studies only 34](#_Toc55379237)

[6.2.1 Pre-eclampsia 34](#_Toc55379238)

[6.2.2 Small for gestational age 34](#_Toc55379239)

[6.2.3 Low birthweight 35](#_Toc55379240)

[6.2.4 Preterm birth 35](#_Toc55379241)

[6.2.5 Stillbirth 35](#_Toc55379242)

[6.2.6 Maternal mortality 35](#_Toc55379243)

[7. Lipid-based nutrients 36](#_Toc55379244)

[7.1 Studies with low/unclear risk of bias only 36](#_Toc55379245)

[7.1.1 Small for gestational age 36](#_Toc55379246)

[7.1.2 Low birthweight 36](#_Toc55379247)

[7.1.3 Preterm birth 36](#_Toc55379248)

[7.1.4 Stillbirth 36](#_Toc55379249)

[7.1.5 Maternal mortality 36](#_Toc55379250)

[7.2 LMIC studies only 37](#_Toc55379251)

[9. Polyunsaturated omega-3 fatty acid 37](#_Toc55379252)

[9.1 Studies with low/unclear risk of bias only 37](#_Toc55379253)

[9.1.1 Pre-eclampsia 37](#_Toc55379254)

[9.1.2 Small for gestational age 38](#_Toc55379255)

[9.1.3 Low birthweight 38](#_Toc55379256)

[9.1.4 Preterm birth 39](#_Toc55379257)

[9.1.5 Stillbirth 40](#_Toc55379258)

[9.1.6 Maternal mortality 40](#_Toc55379259)

[9.2 LMIC studies only 41](#_Toc55379260)

[9.2.1 Pre-eclampsia 41](#_Toc55379261)

[9.2.2 Small for gestational age 42](#_Toc55379262)

[9.2.3 Low birthweight 42](#_Toc55379263)

[9.2.4 Preterm birth 43](#_Toc55379264)

[9.2.5 Stillbirth 44](#_Toc55379265)

[9.2.6 Maternal mortality 44](#_Toc55379266)

[10. Antenatal dietary counselling 45](#_Toc55379267)

[10.1 Studies with low/unclear risk of bias only 45](#_Toc55379268)

[10.1.1 Pre-eclampsia 45](#_Toc55379269)

[10.1.2 Small for gestational age 46](#_Toc55379270)

[10.1.3 Low birthweight 46](#_Toc55379271)

[10.1.4 Preterm birth 47](#_Toc55379272)

[10.1.5 Stillbirth 47](#_Toc55379273)

[10.1.6 Maternal mortality 47](#_Toc55379274)

[10.2 LMIC studies only 48](#_Toc55379275)

[10.2.1 Pre-eclampsia 48](#_Toc55379276)

[10.2.2 Small for gestational age 48](#_Toc55379277)

[10.2.3 Low birthweight 48](#_Toc55379278)

[10.2.4 Preterm birth 48](#_Toc55379279)

[10.2.5 Stillbirth 48](#_Toc55379280)

[10.2.6 Maternal mortality 48](#_Toc55379281)

# 1. Vitamin A

## 1.1 Studies with low/unclear risk of bias only

N/A - no studies with high risk of bias

## 10.1.2 LMIC studies only

N/A - no studies conducted in HICs

# 2. Vitamin C and/or E

## 2.1 Studies with low/unclear risk of bias only

### 2.1.1 Pre-eclampsia


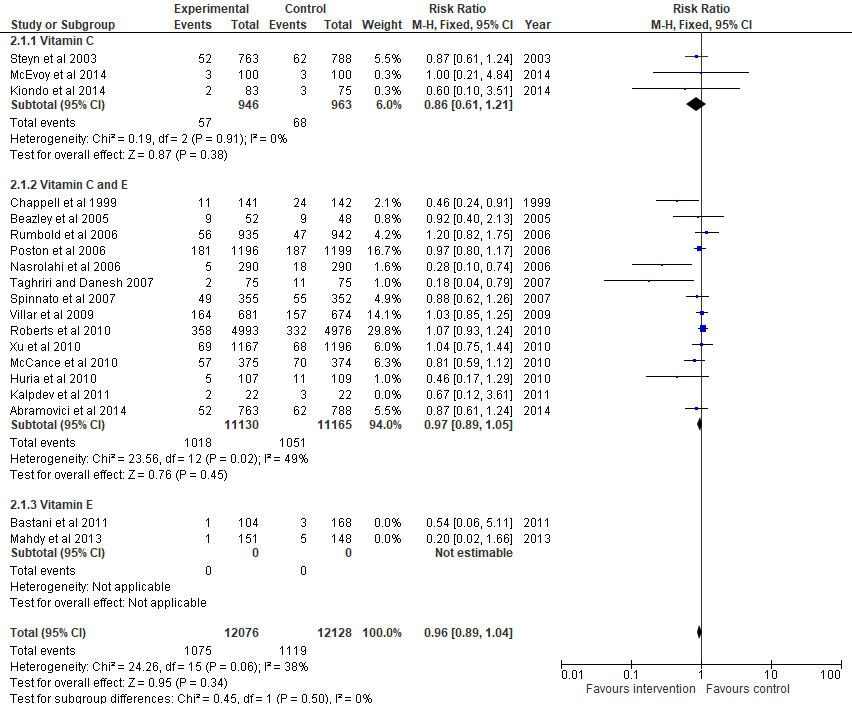


### 2.1.2 Small for gestational age


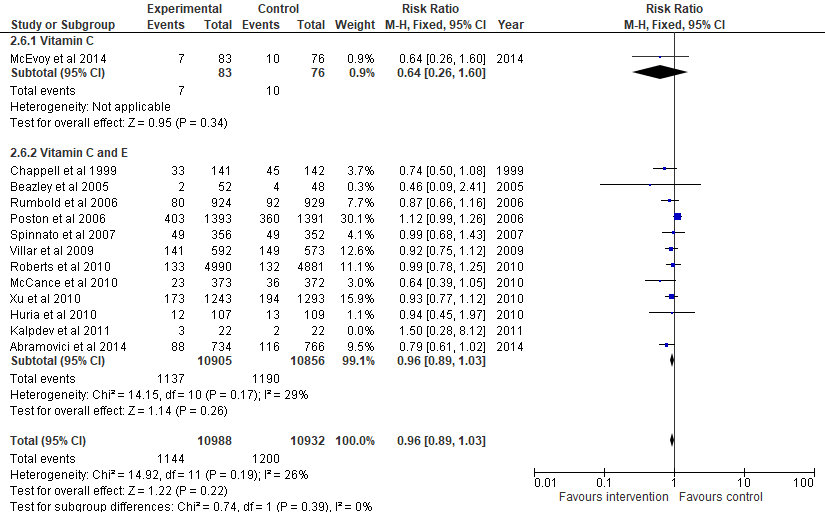


### 2.1.3 Low birthweight


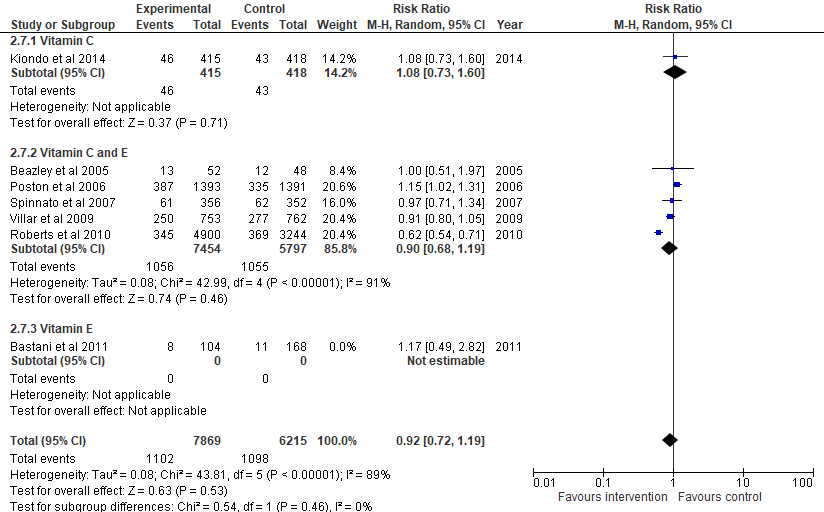


### 2.1.4 Preterm birth


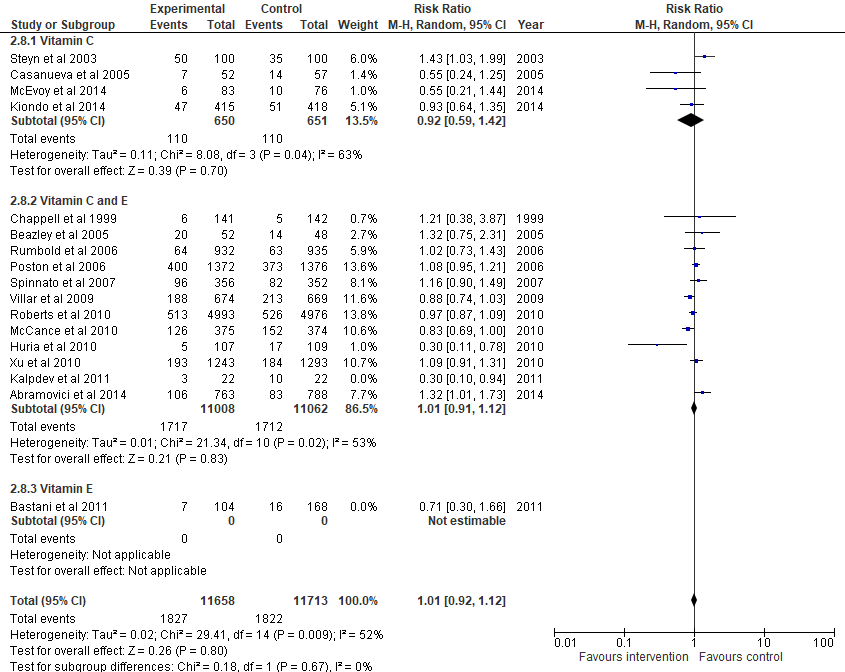


### 2.1.5 Stillbirth

N/A – none included were studies with high risk of bias

### 2.1.6 Maternal mortality

N/A – none included were studies with high risk of bias

## 2.2 LMIC studies only

### 2.2.1 Pre-eclampsia


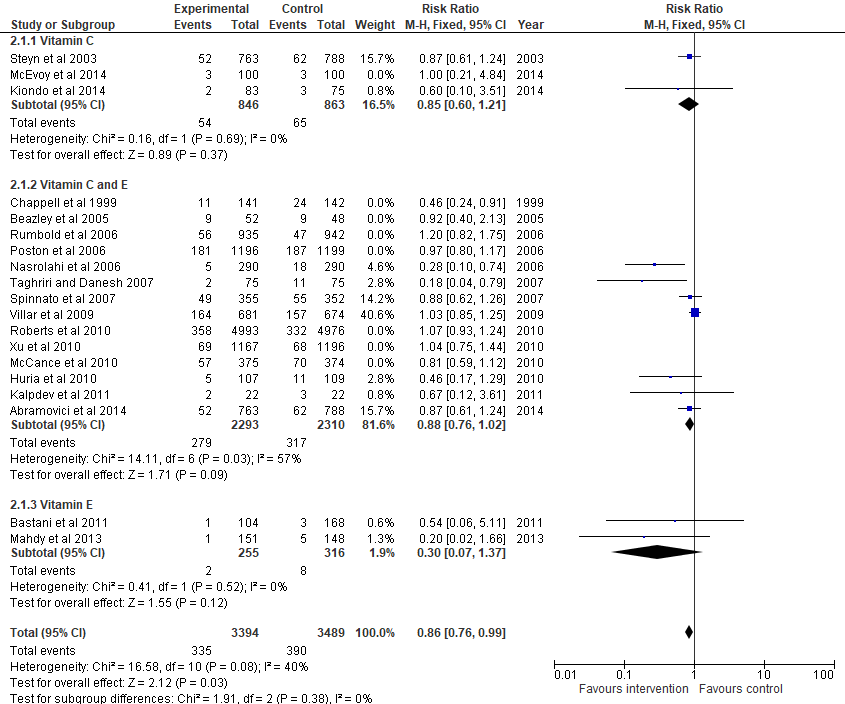


### 2.2.2 Small for gestational age


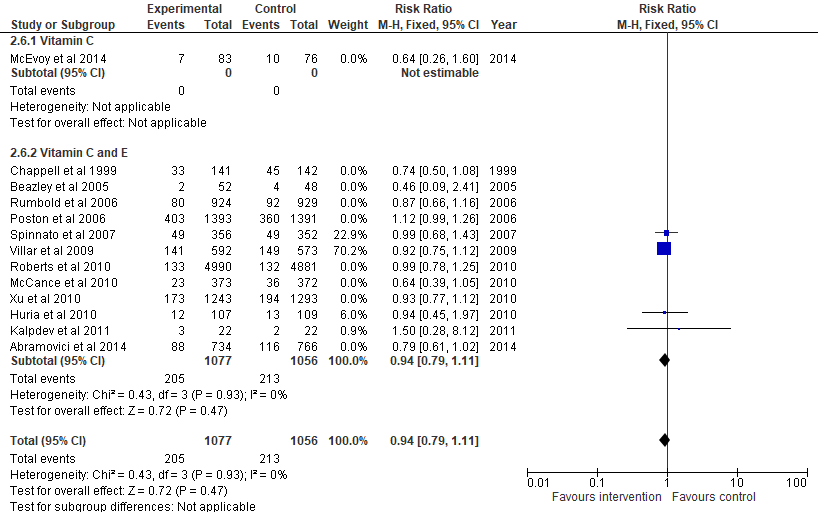


### 2.2.3 Low birthweight


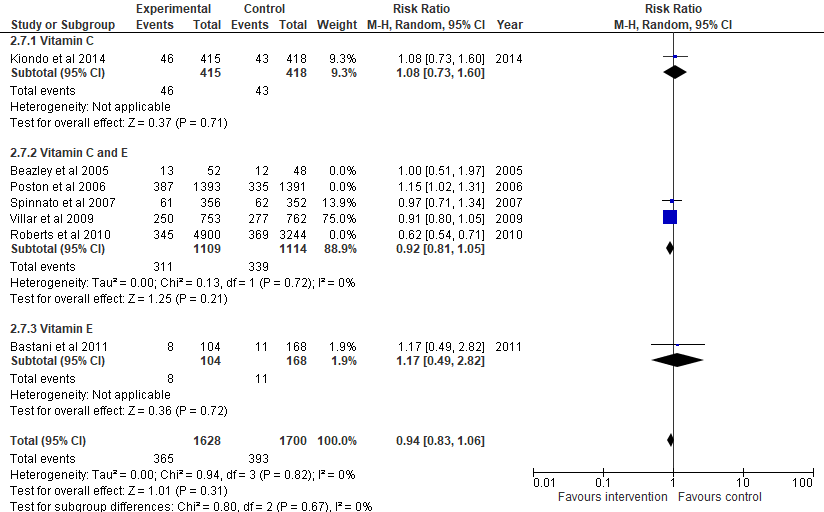


### 2.2.4 Preterm birth


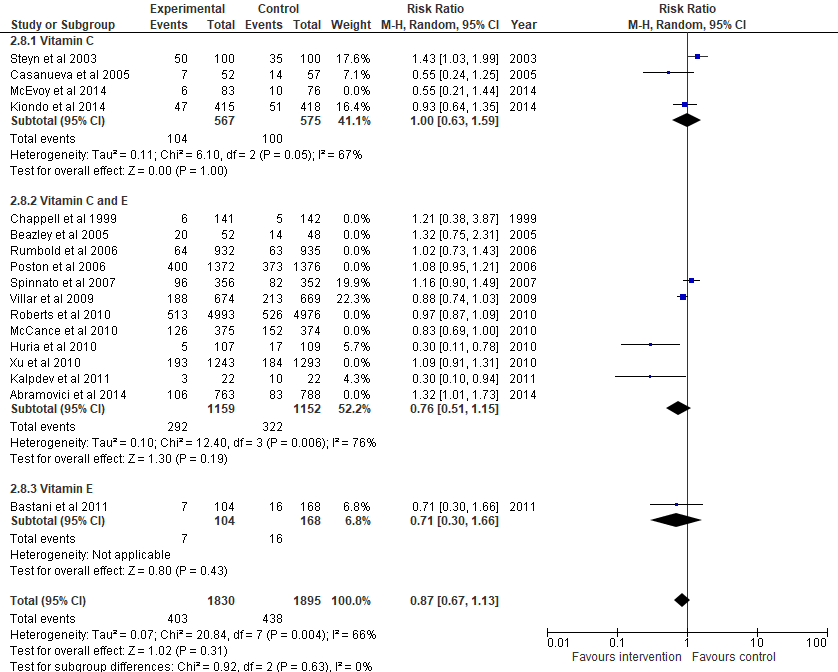


### 2.2.5 Stillbirth


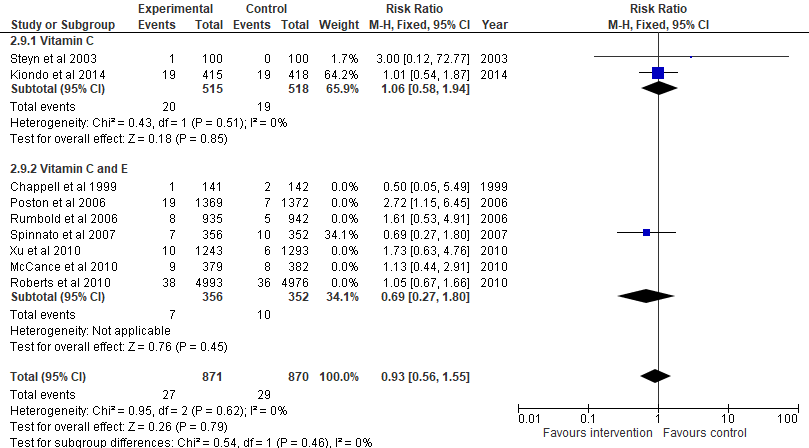


### 2.2.6 Maternal mortality


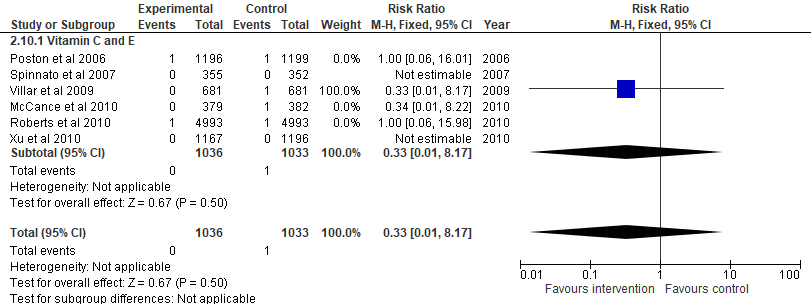


# 3. Vitamin D and/or calcium

## 3.1 Studies with low/unclear risk of bias only

### 3.1.1 Pre-eclampsia

####
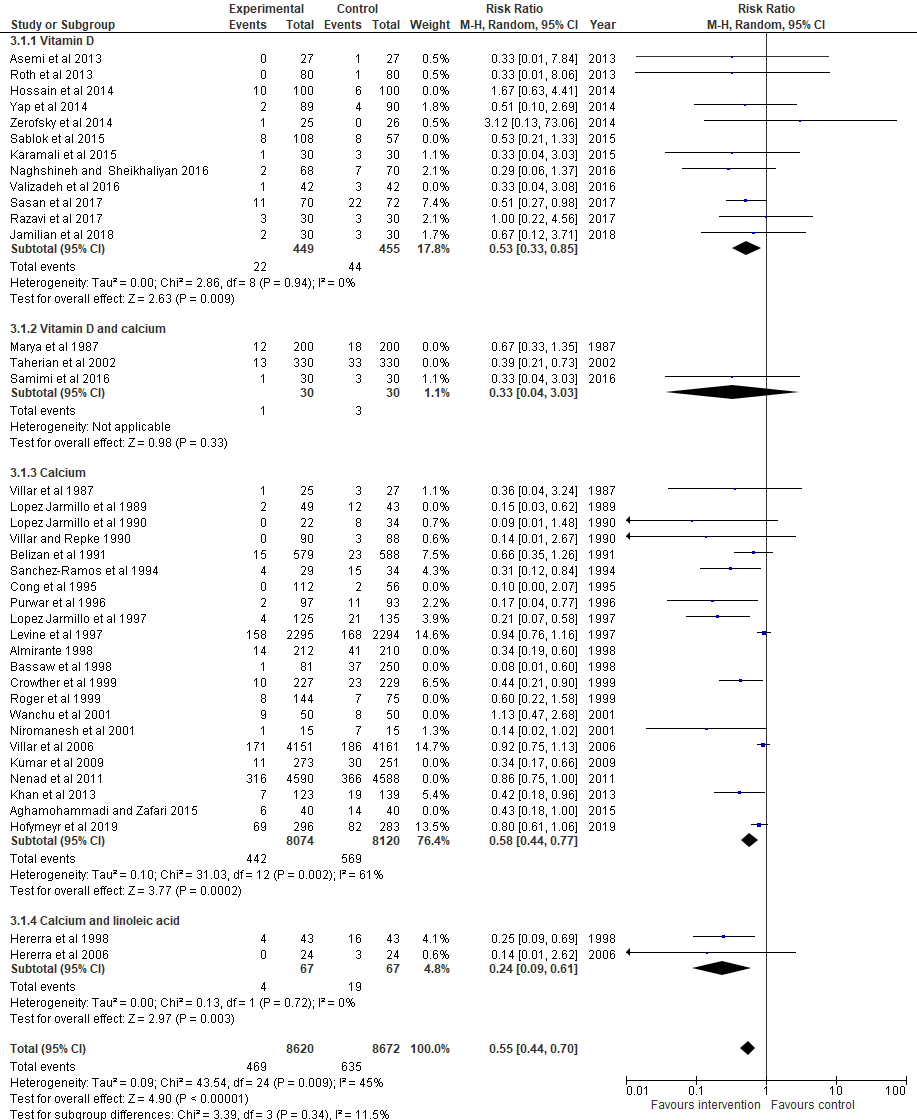


### 3.1.2 Small for gestational age


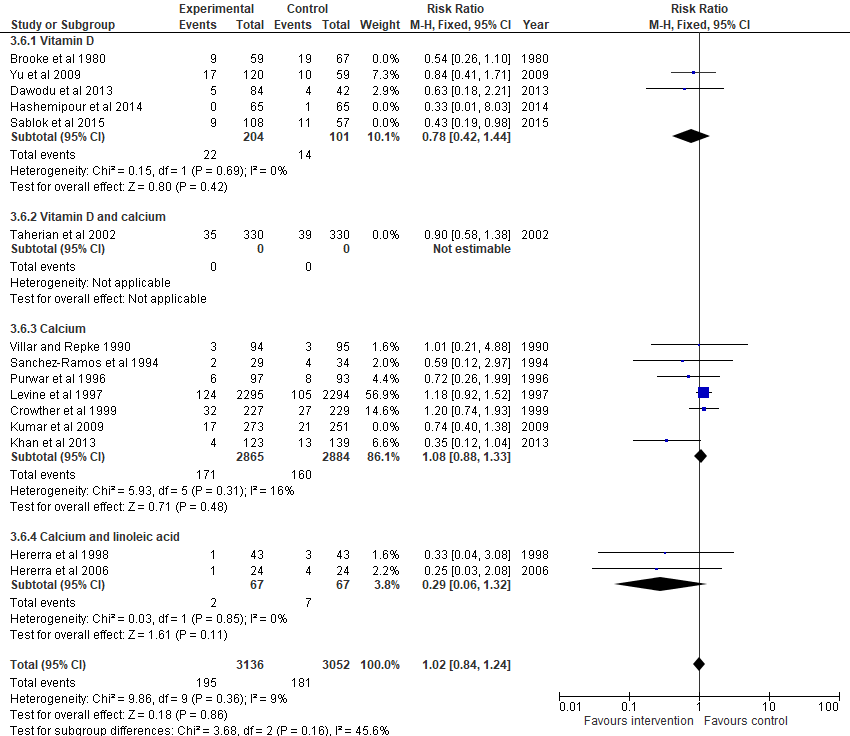


### 3.1.3 Low birthweight


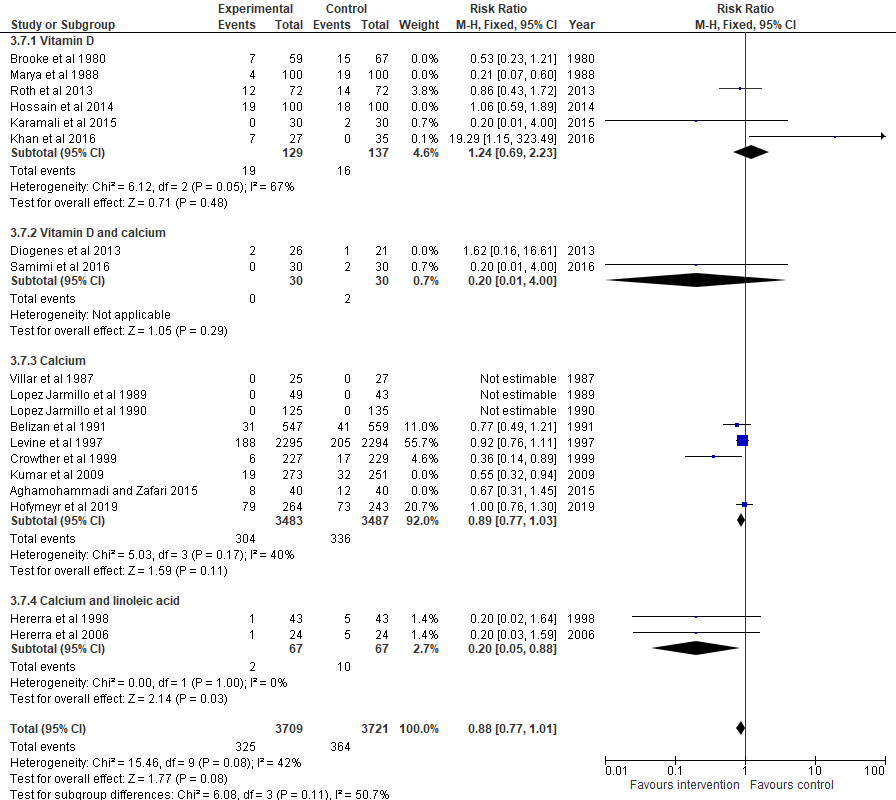


### 3.1.4 Preterm birth


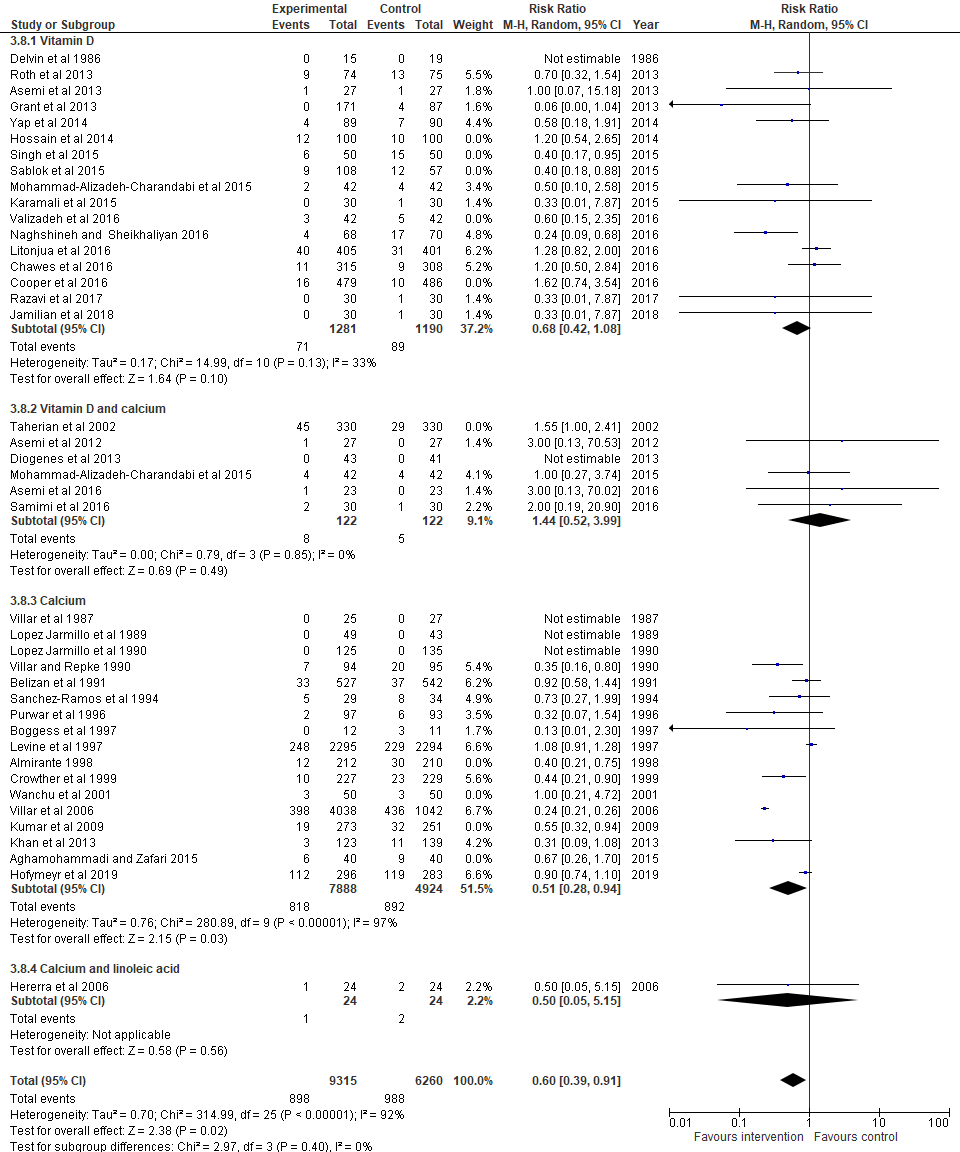


### 3.1.5 Stillbirth


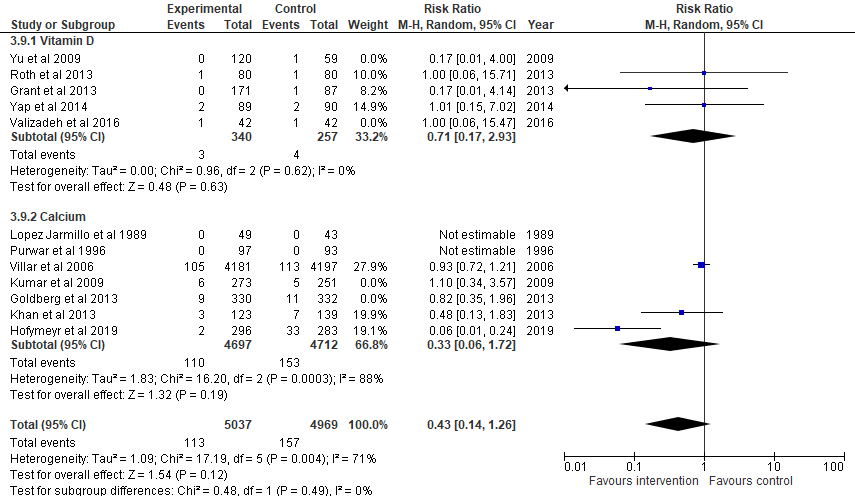


### 3.1.6 Maternal mortality


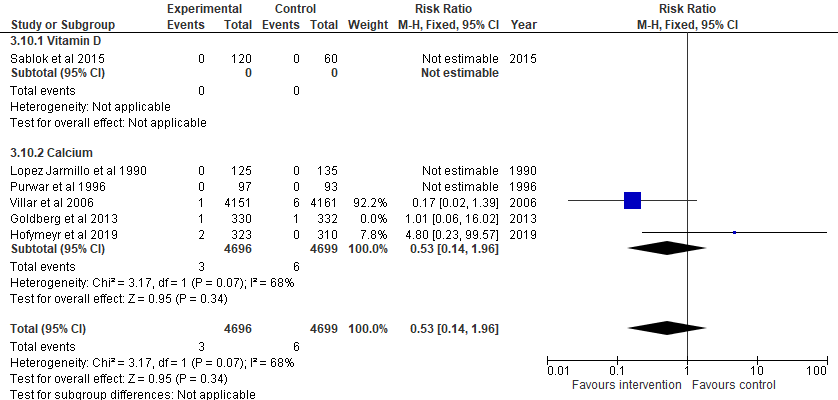


## 3.2 LMIC studies only

### 3.2.1 Pre-eclampsia


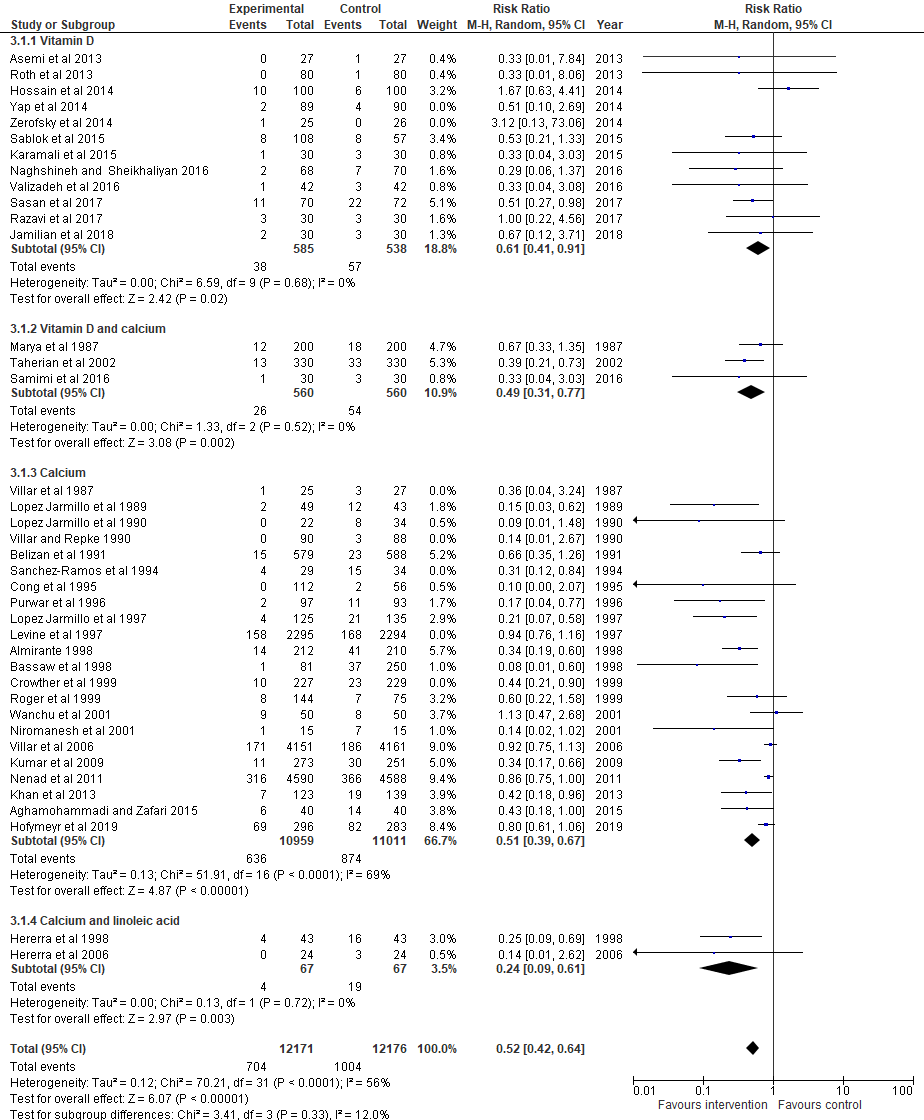


### 3.2.2 Small for gestational age


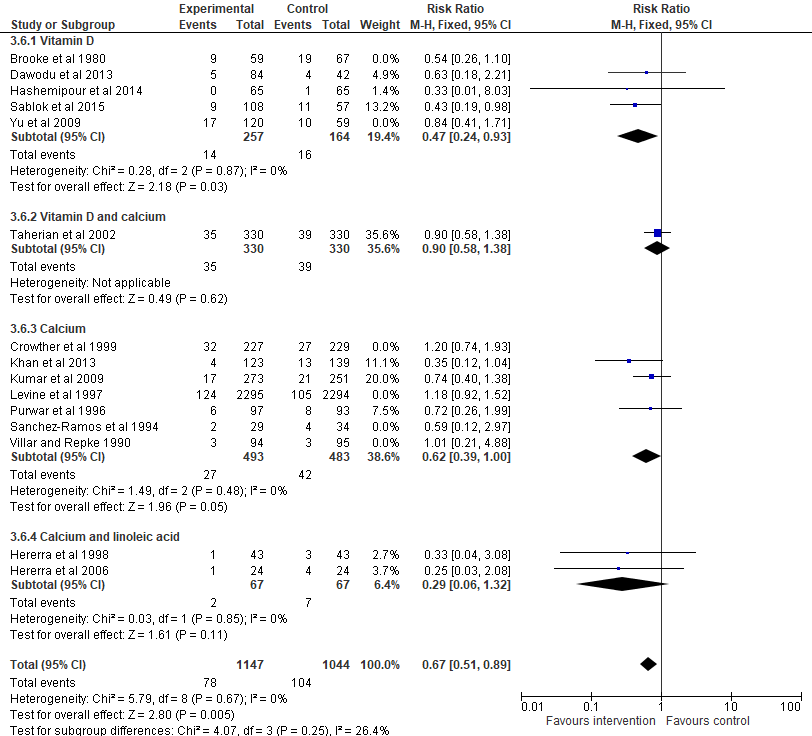


### 3.2.3 Low birthweight


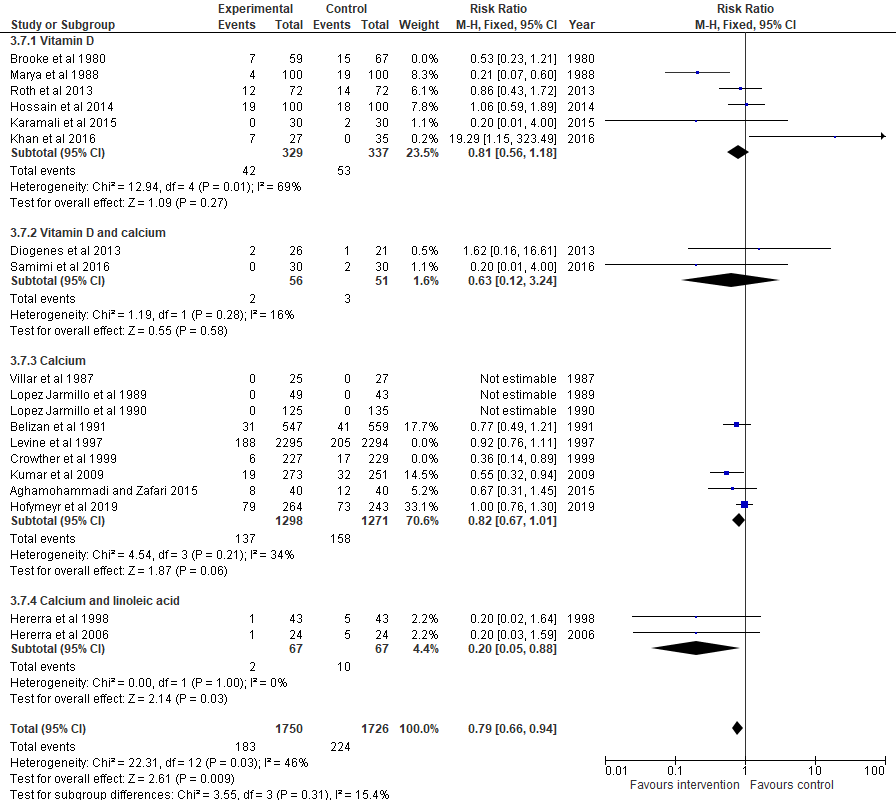


### 3.2.4 Preterm birth


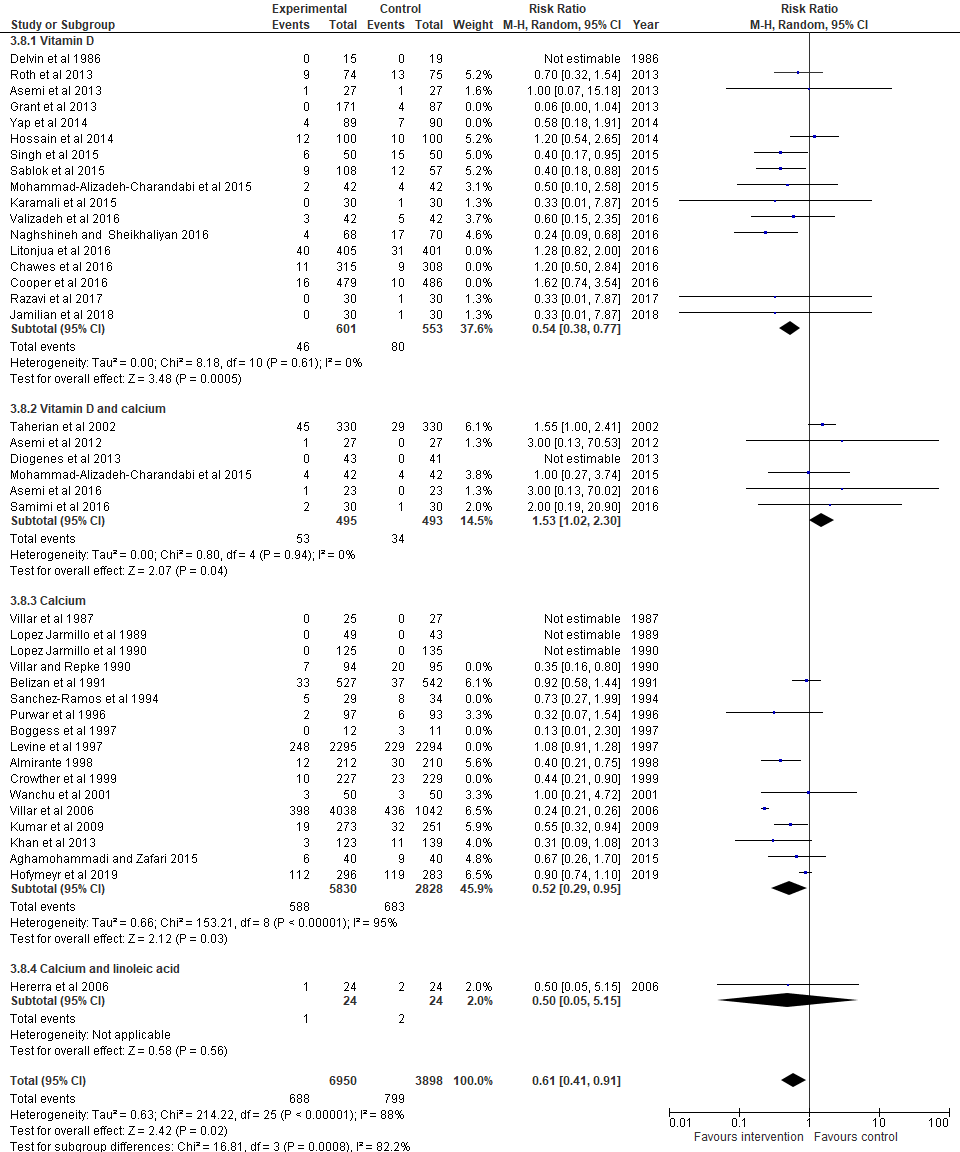


### 3.2.5 Stillbirth


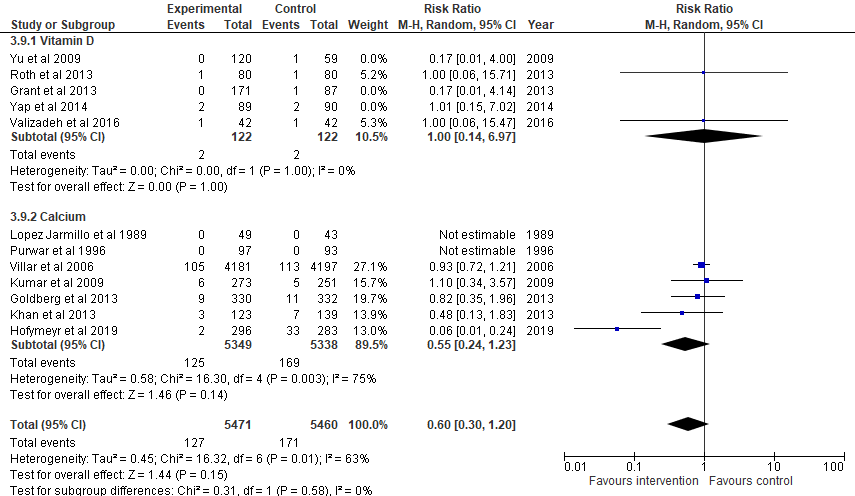


### 3.2.6 Maternal mortality

N/A – none included were studies from HICs

# 4. Iron and/or folic acid

## 4.1 Studies with low/unclear risk of bias only

### 4.1.1 Pre-eclampsia


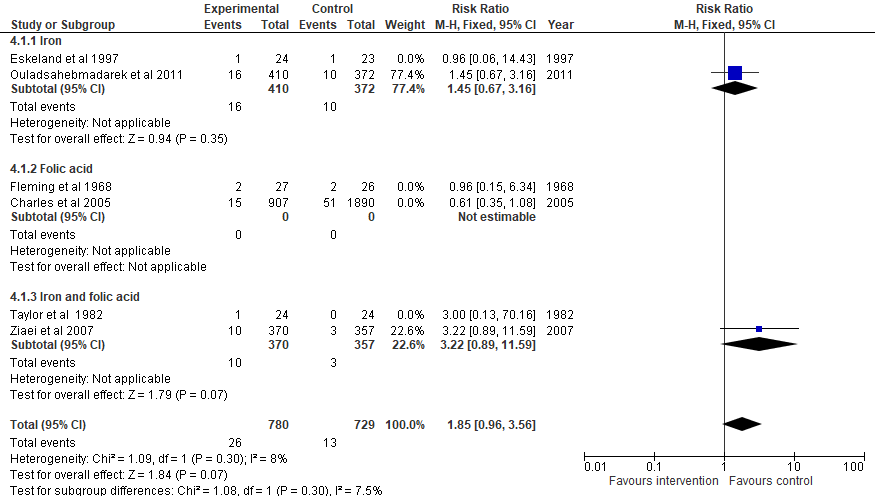


### 4.1.2 Small for gestational age


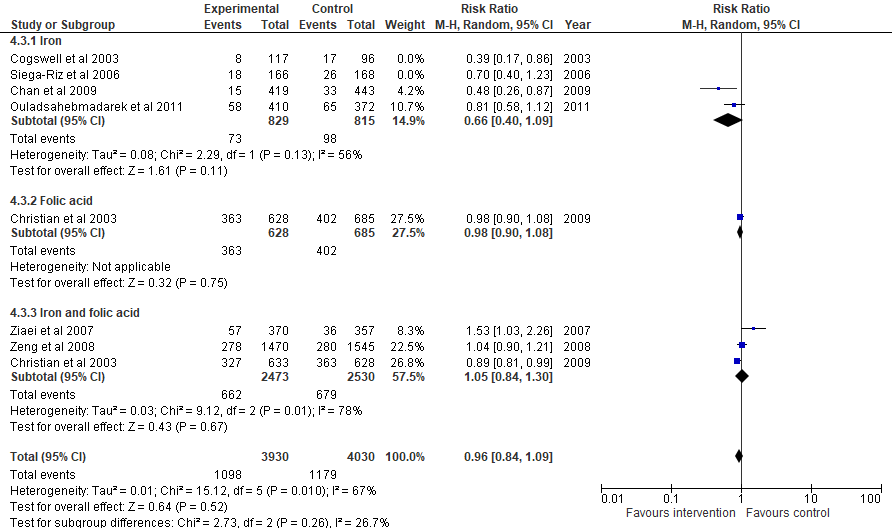


### 4.1.3 Low birthweight


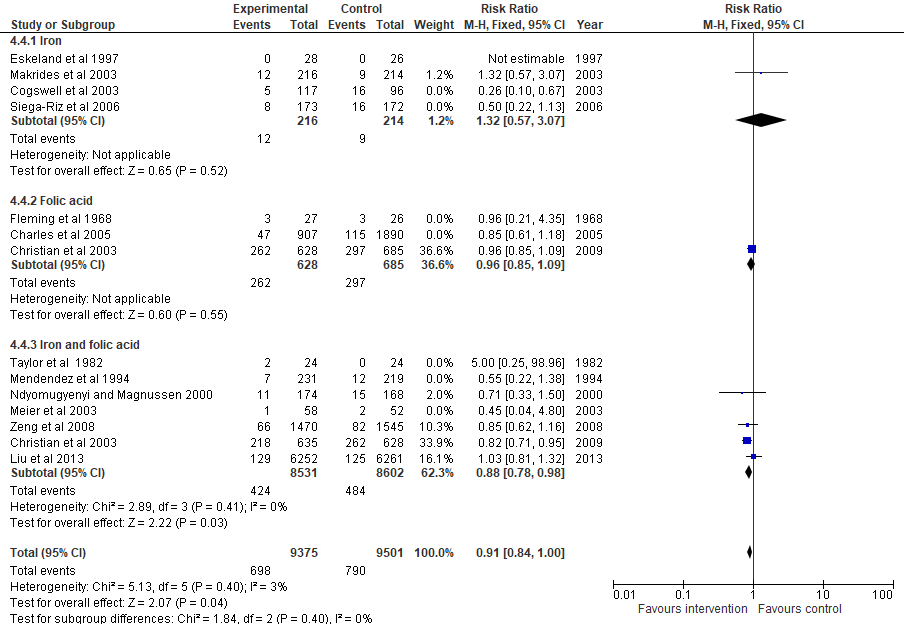


### 4.1.4 Preterm birth


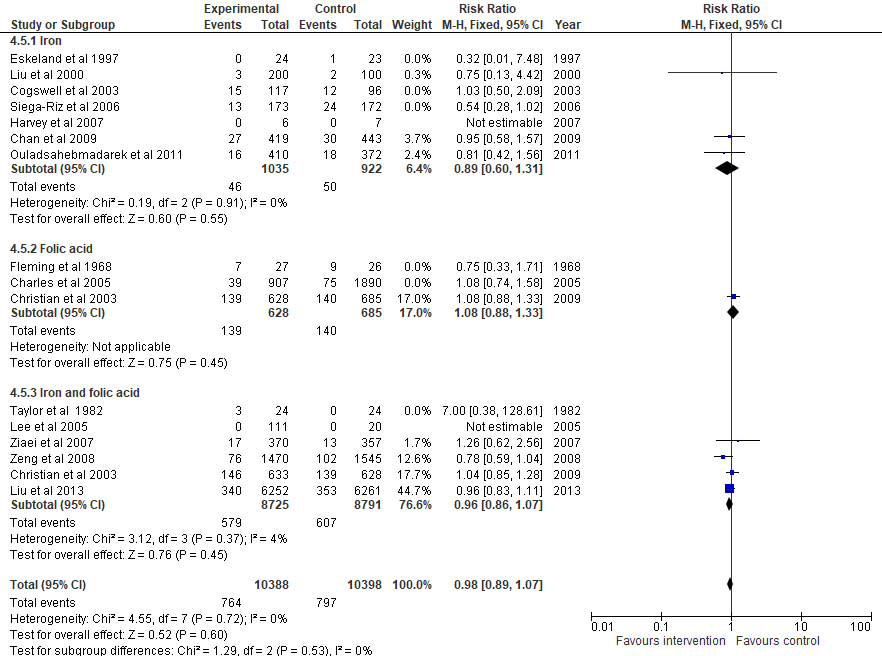


### 4.1.5 Stillbirths


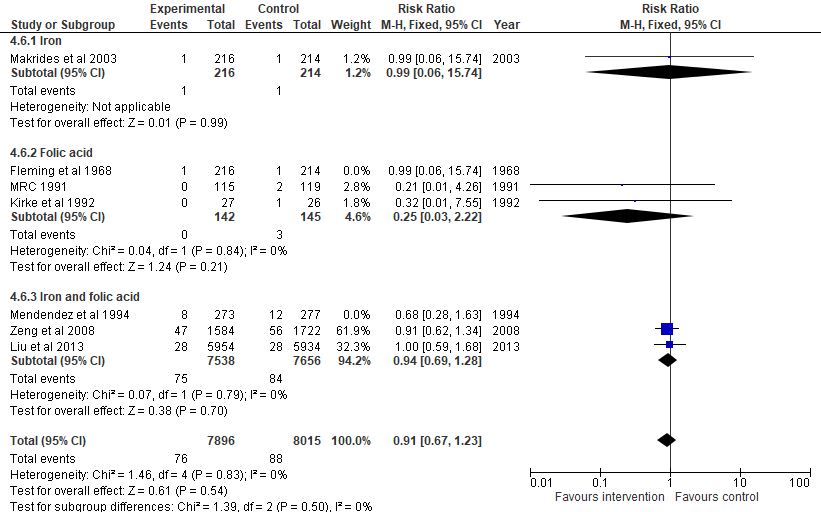


### 4.1.6 Maternal mortality

N/A – no included studies after high risk of bias studies excluded

## 4.2 LMIC studies only

### 4.2.1 Pre-eclampsia


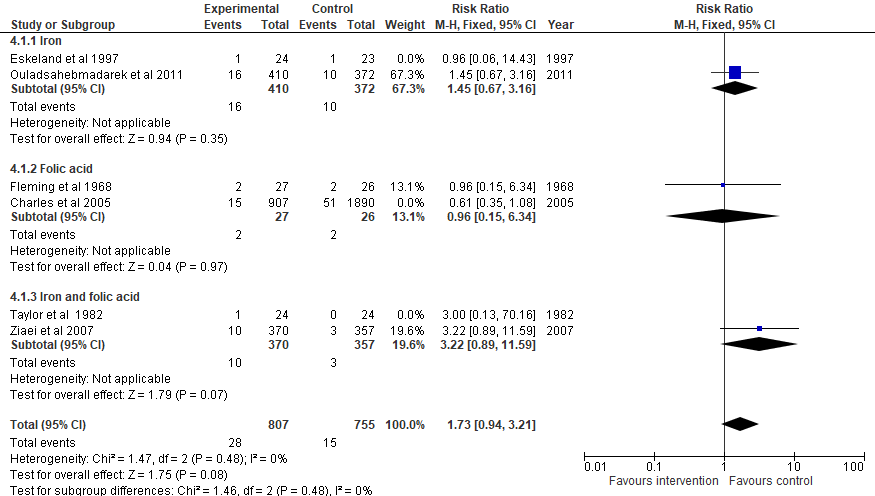


### 4.2.2 Small for gestational age


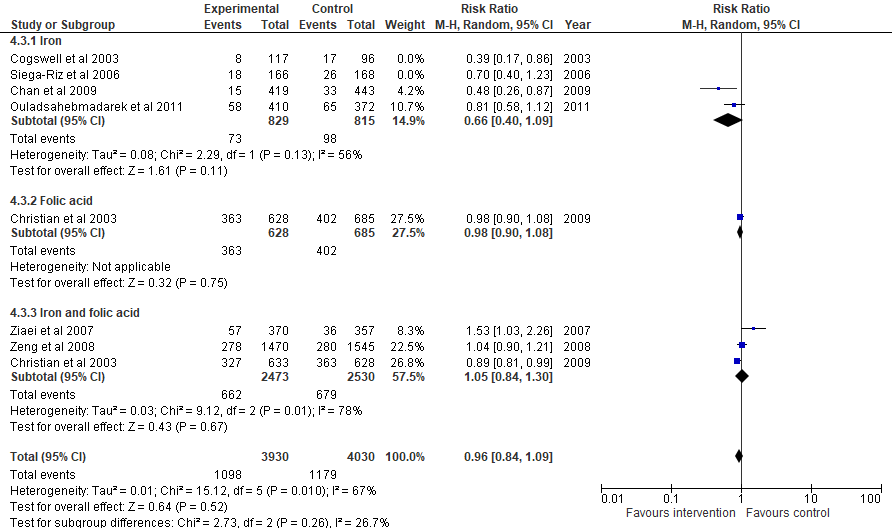


### 4.2.3 Low birthweight


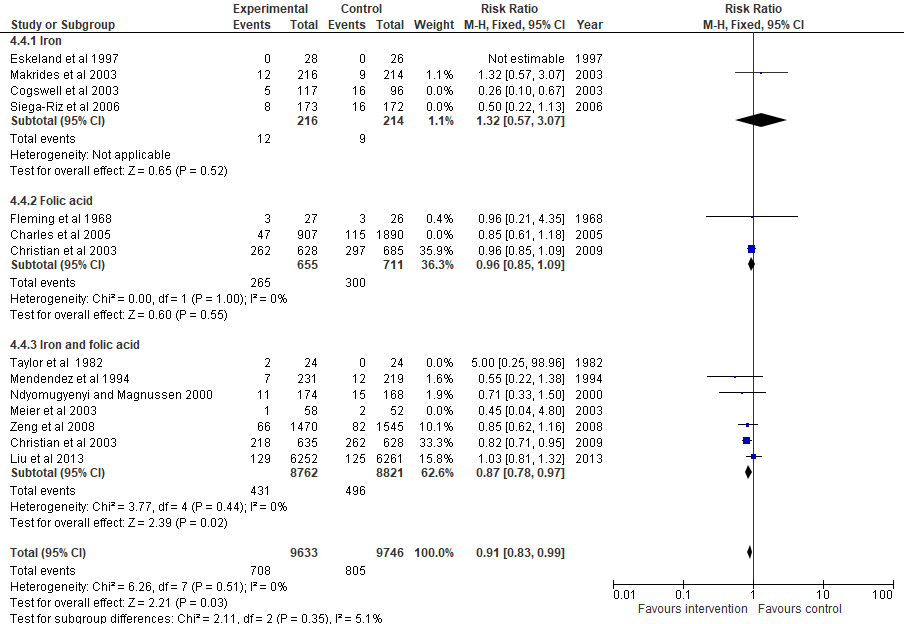


### 4.2.4 Preterm birth

####
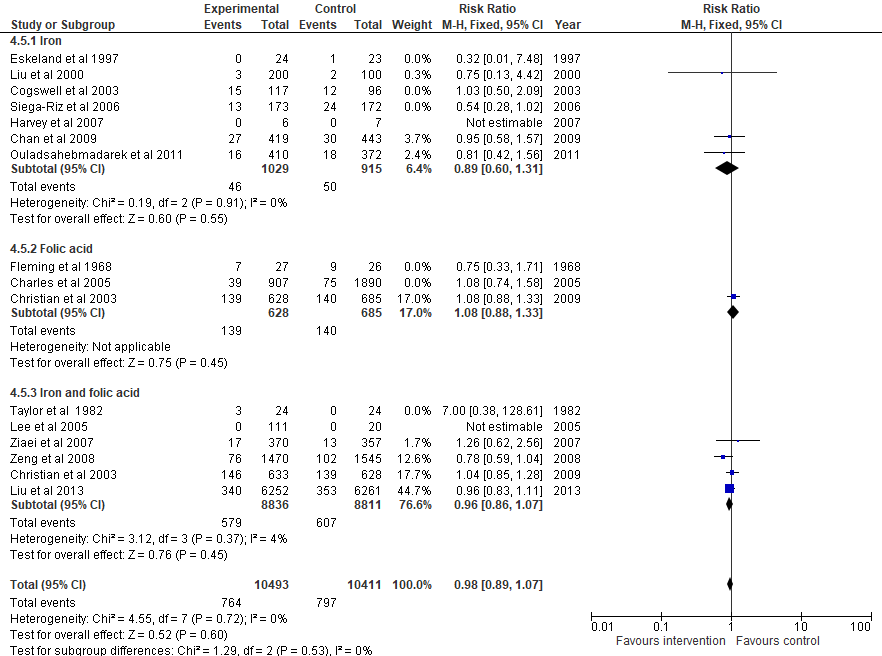


### 4.2.5 Stillbirth


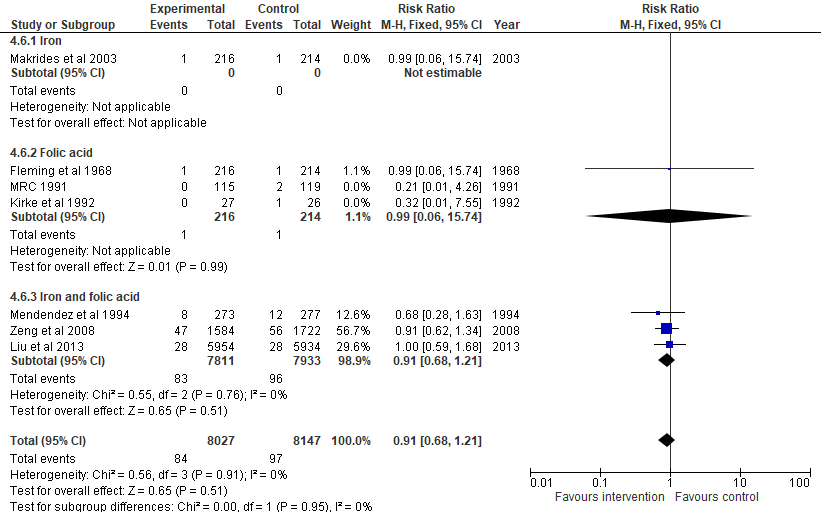


### 4.2.6 Maternal mortality

N/A – same as full analysis

# 5. Zinc

## 5.1 Studies with low/unclear risk of bias only

### 5.1.1 Pre-eclampsia


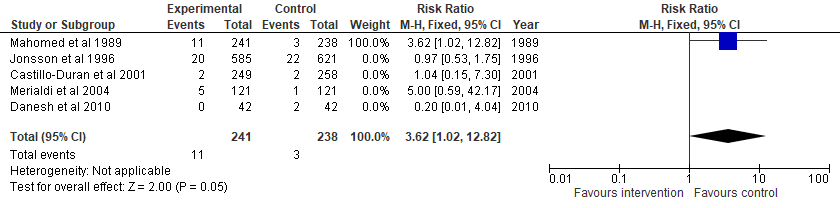


### 5.1.2 Small for gestational age


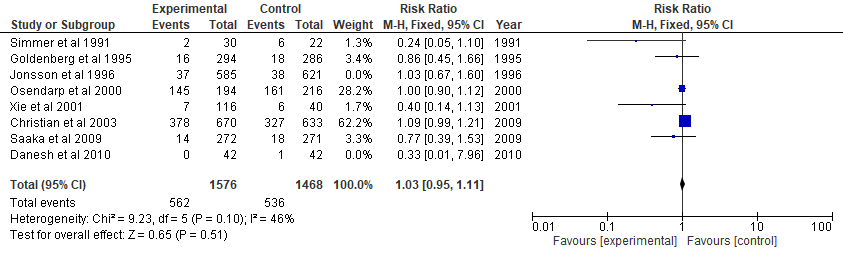


### 5.1.3 Low birthweight


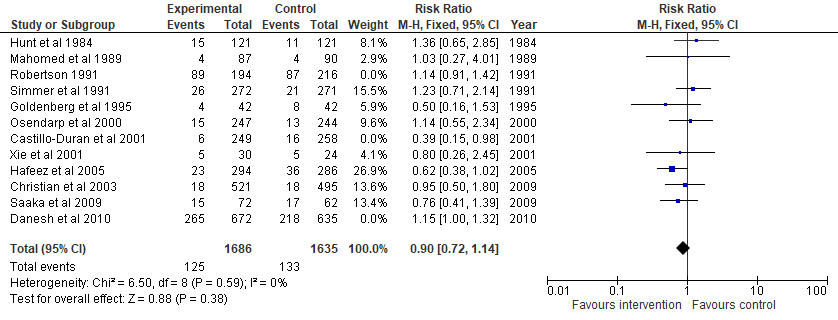


### 5.1.4 Preterm birth


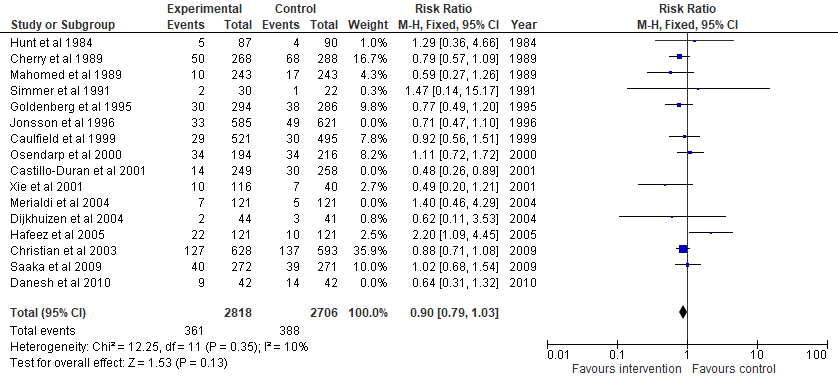


### 5.1.5 Stillbirth

N/A – both studies excluded

### 5.1.6 Maternal mortality

N/A – no change

## 5.2 LMIC studies only

### 5.2.1 Pre-eclampsia


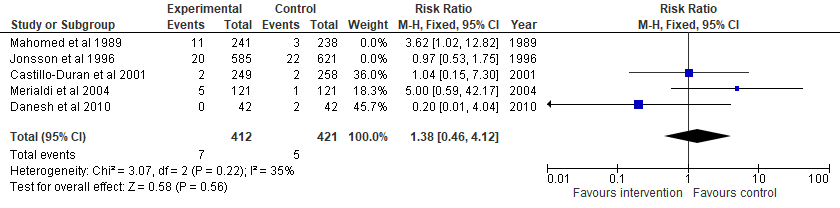


### 5.2.2 Small for gestational age


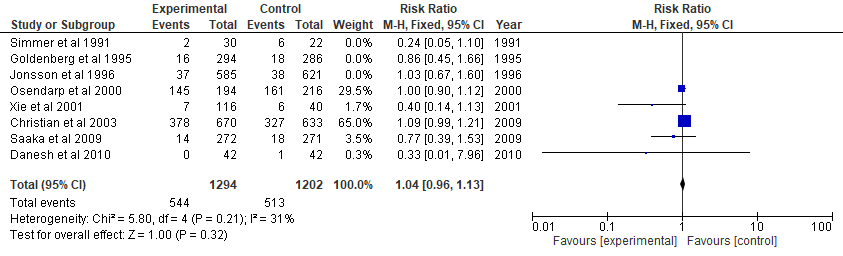


### 5.2.3 Low birthweight


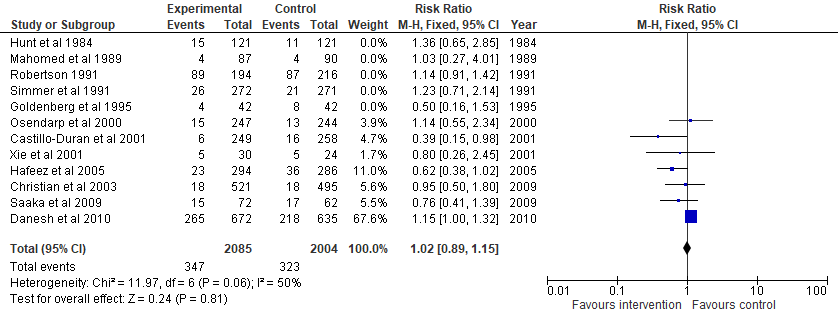


### 5.2.4 Preterm birth


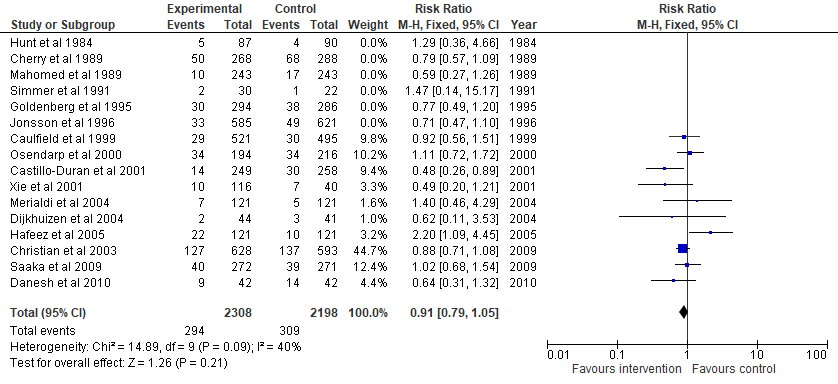


### 5.2.5 Stillbirth


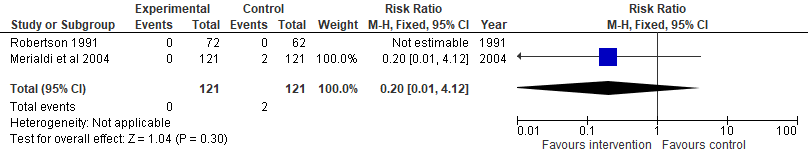


### 5.2.6 Maternal mortality

N/A – no change

# 6. Multiple micronutrients

## 6.1 Studies with low/unclear risk of bias only

### 6.1.1 Pre-eclampsia


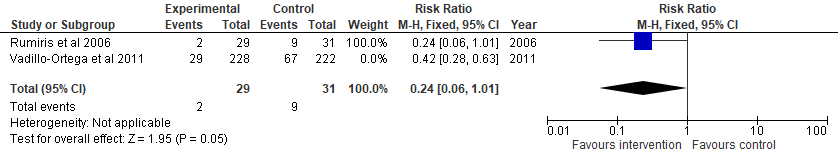


### 6.1.2 Small for gestational age


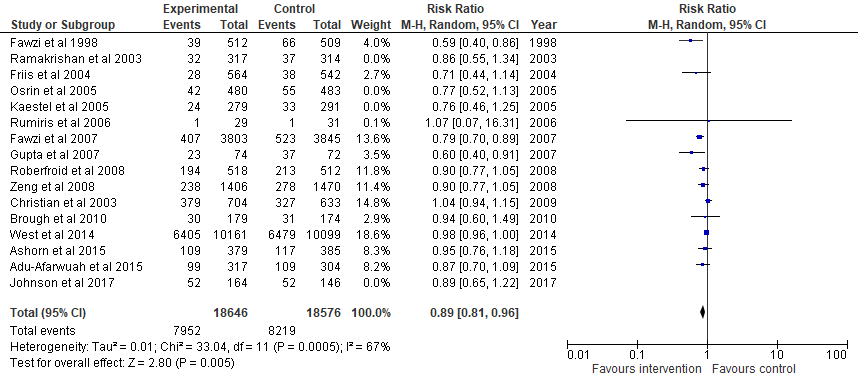


### 6.1.3 Low birthweight


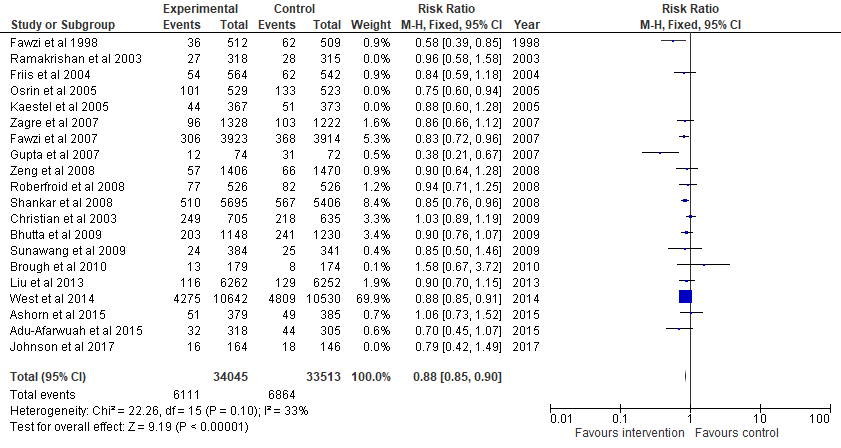


### 6.1.4 Preterm birth


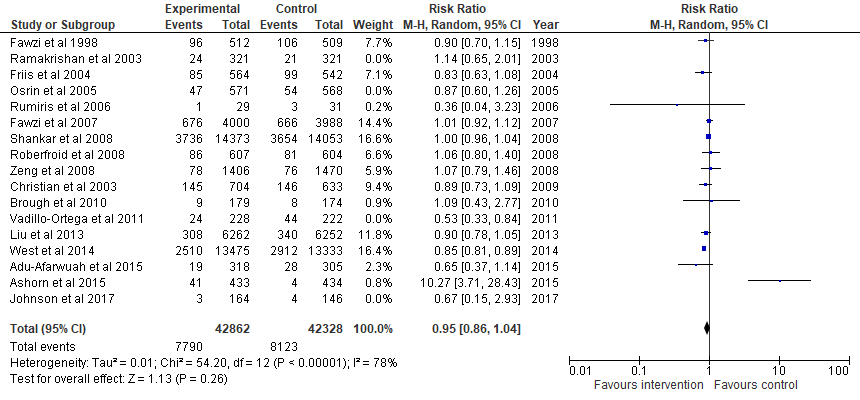


### 6.1.5 Stillbirth


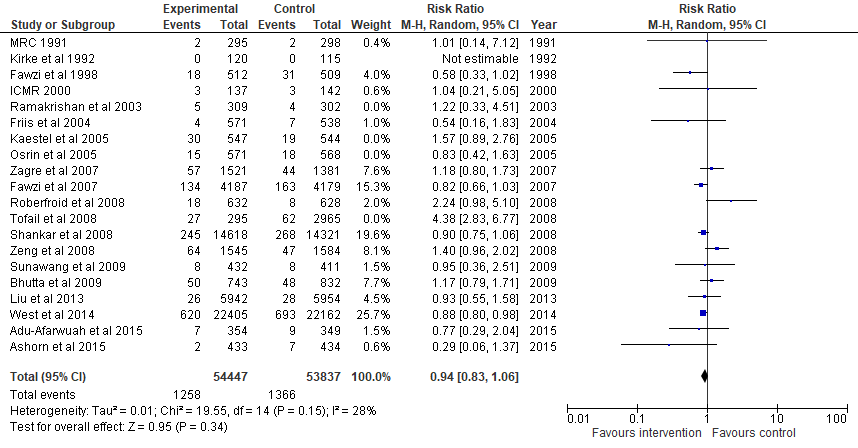


### 6.1.6 Maternal mortality


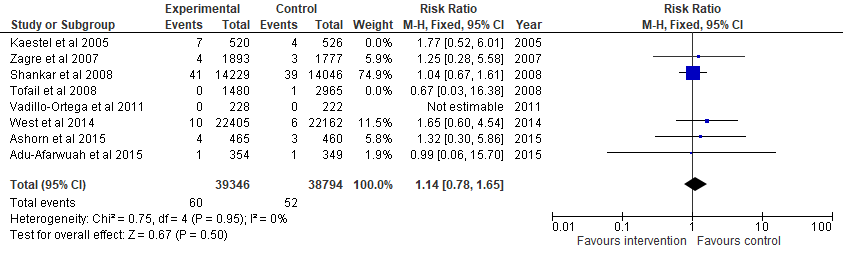


## 6.2 LMIC studies only

### 6.2.1 Pre-eclampsia

N/A – no change

### 6.2.2 Small for gestational age


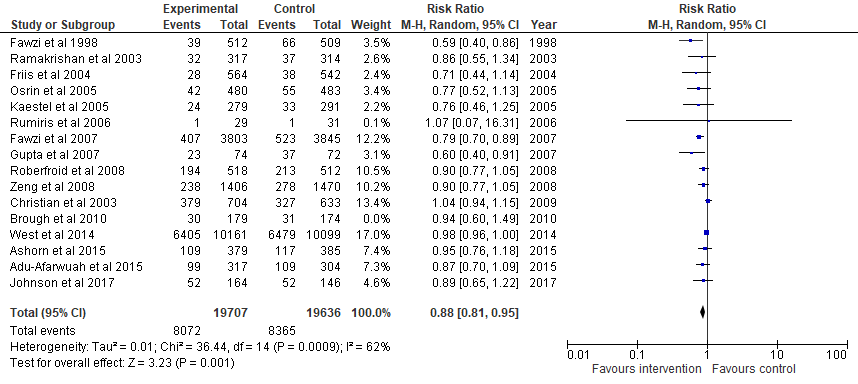


### 6.2.3 Low birthweight


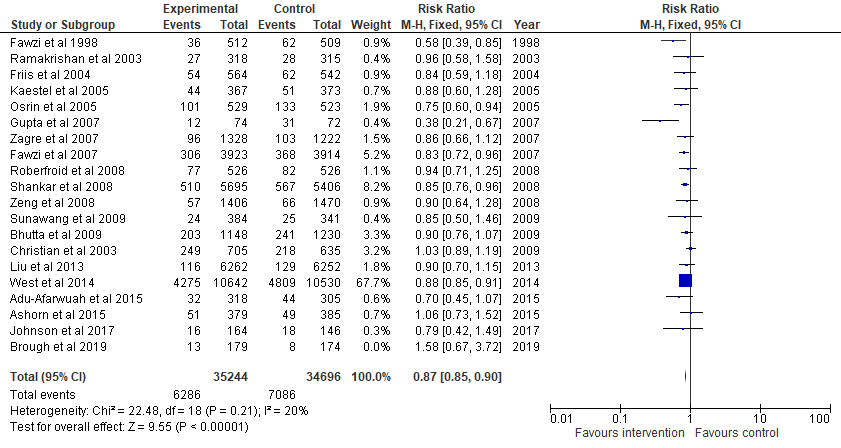


### 6.2.4 Preterm birth


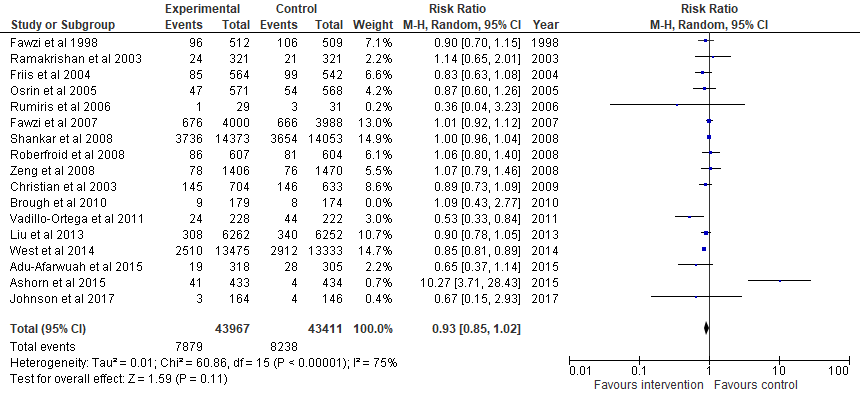


### 6.2.5 Stillbirth

N/A – no change

### 6.2.6 Maternal mortality

N/A – no change

# 7. Lipid-based nutrients

## 7.1 Studies with low/unclear risk of bias only

### 7.1.1 Small for gestational age


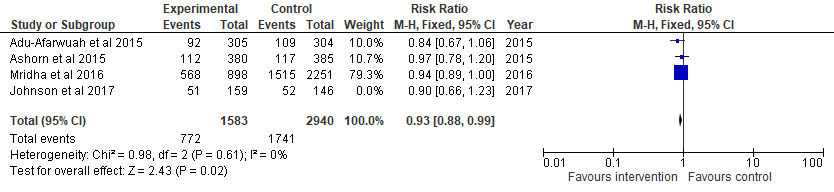


### 7.1.2 Low birthweight


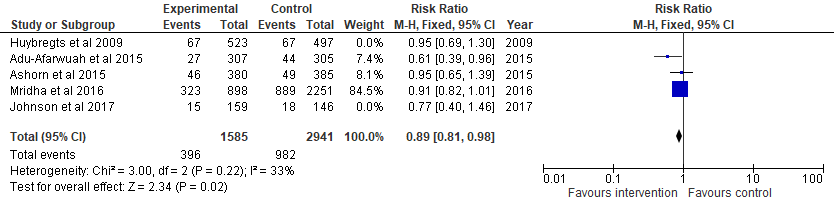


### 7.1.3 Preterm birth


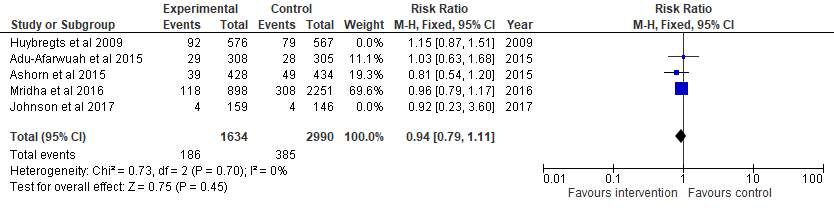


### 7.1.4 Stillbirth


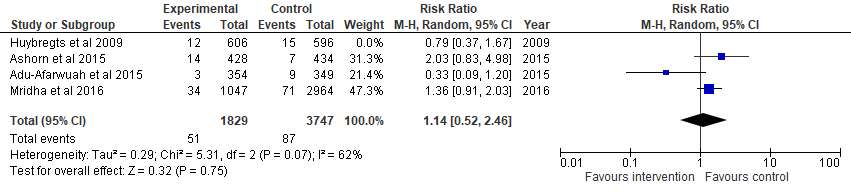


### 7.1.5 Maternal mortality

N/A – no change

## 7.2 LMIC studies only

N/A – all included were conducted in LMICs

# 9. Polyunsaturated omega-3 fatty acid

## 9.1 Studies with low/unclear risk of bias only

### 9.1.1 Pre-eclampsia


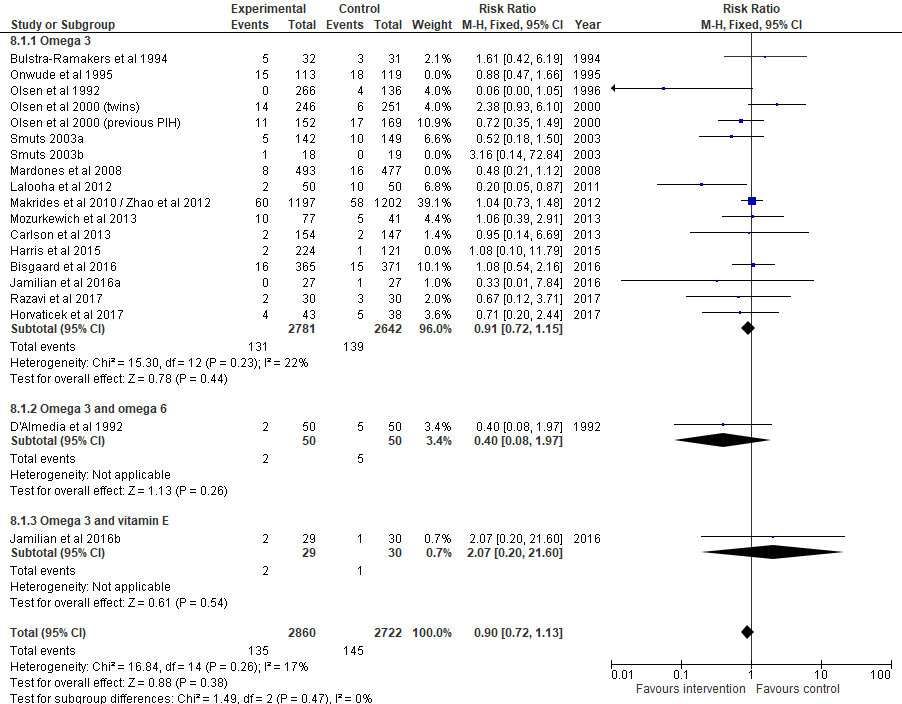


### 9.1.2 Small for gestational age


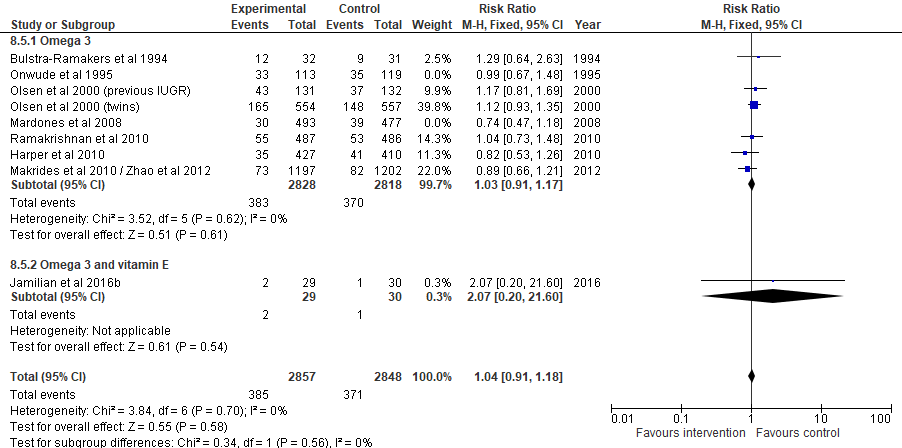


### 9.1.3 Low birthweight


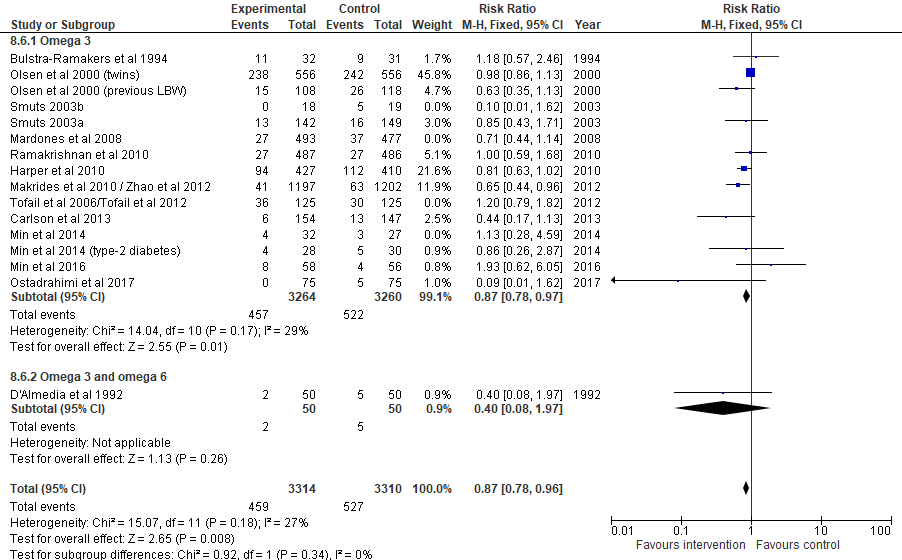


### 9.1.4 Preterm birth


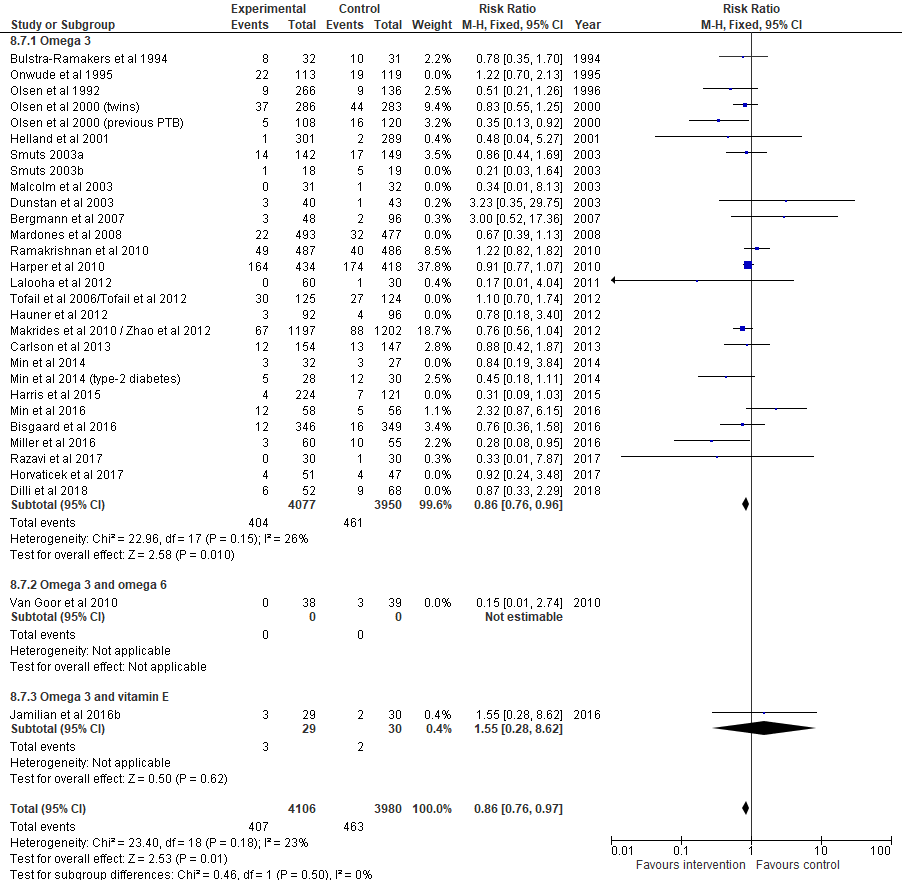


### 9.1.5 Stillbirth


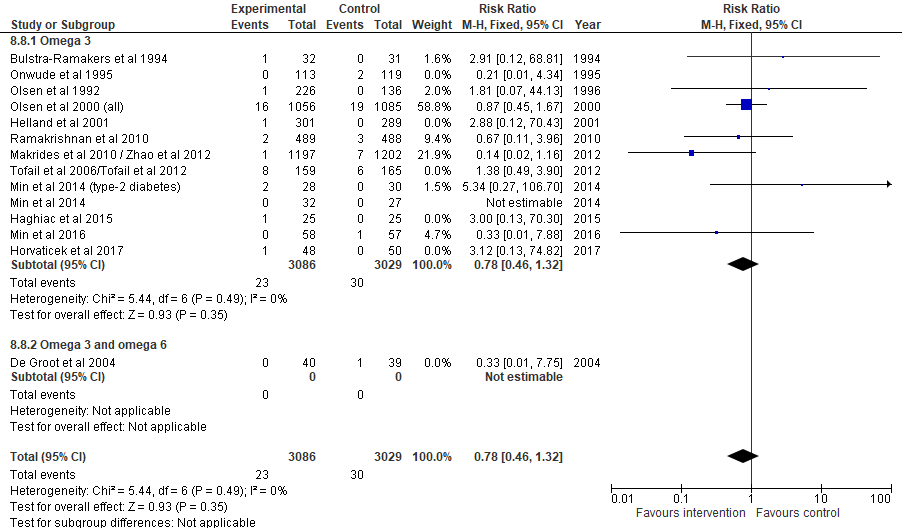


### 9.1.6 Maternal mortality

N/A- no changes

## 9.2 LMIC studies only

### 9.2.1 Pre-eclampsia


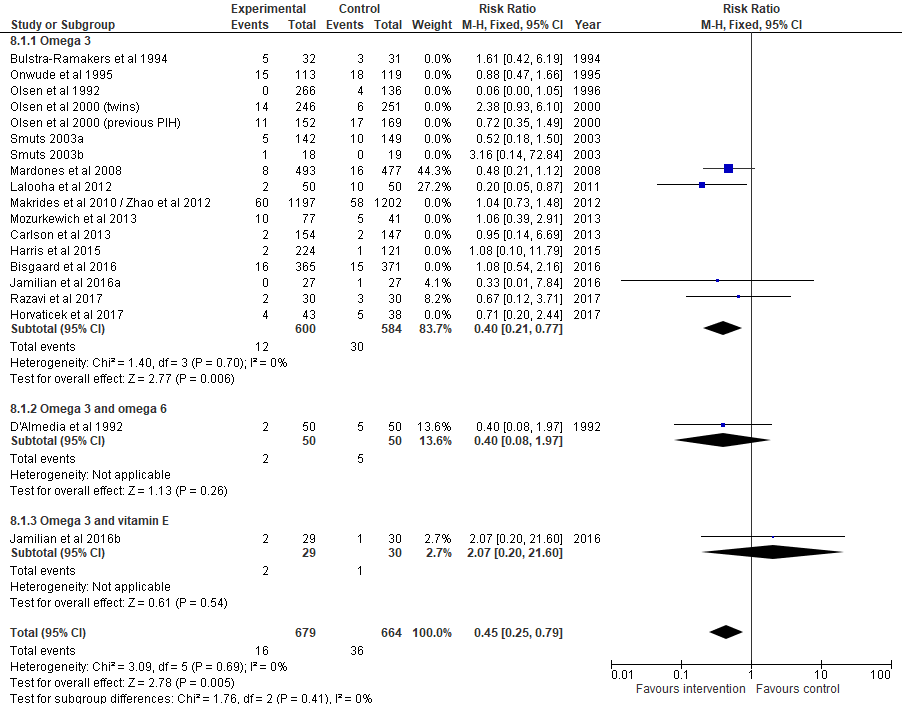


### 9.2.2 Small for gestational age


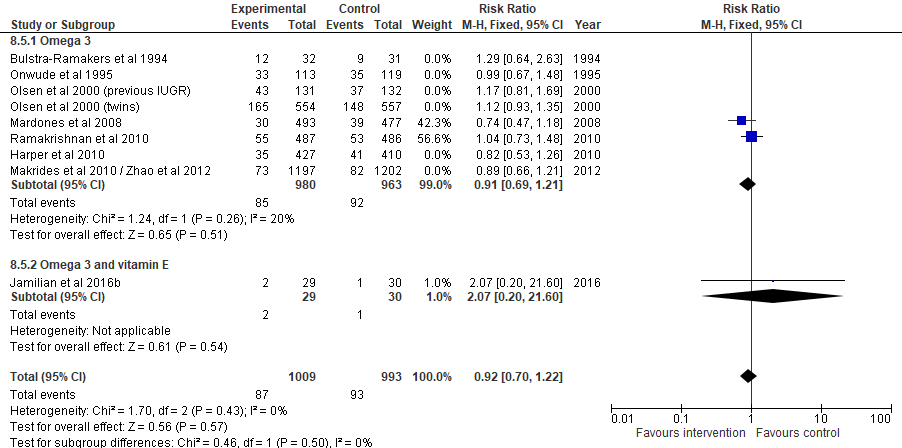


### 9.2.3 Low birthweight


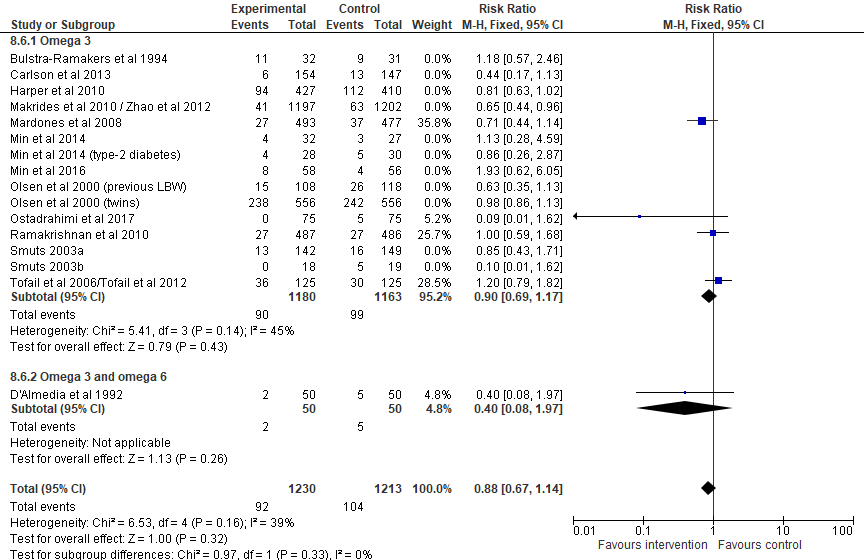


### 9.2.4 Preterm birth


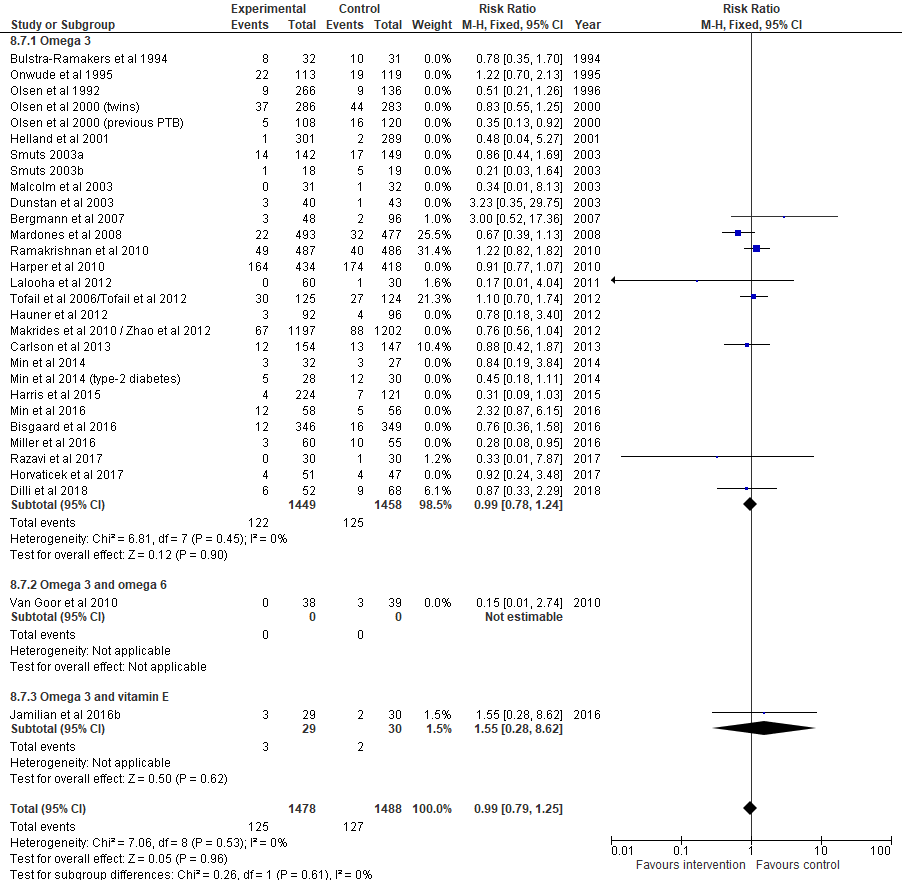


### 9.2.5 Stillbirth


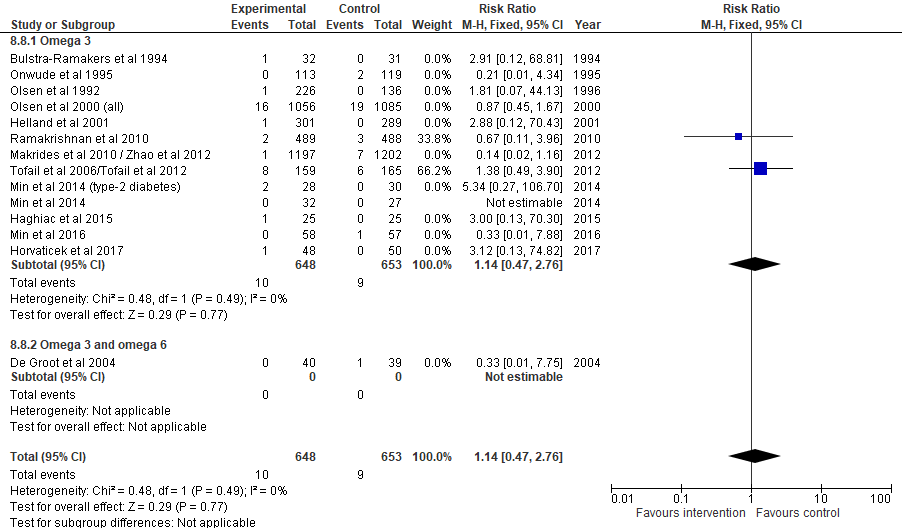


### 9.2.6 Maternal mortality

N/A – all studies were conducted in HICs

# 10. Antenatal dietary counselling

## 10.1 Studies with low/unclear risk of bias only

### 10.1.1 Pre-eclampsia


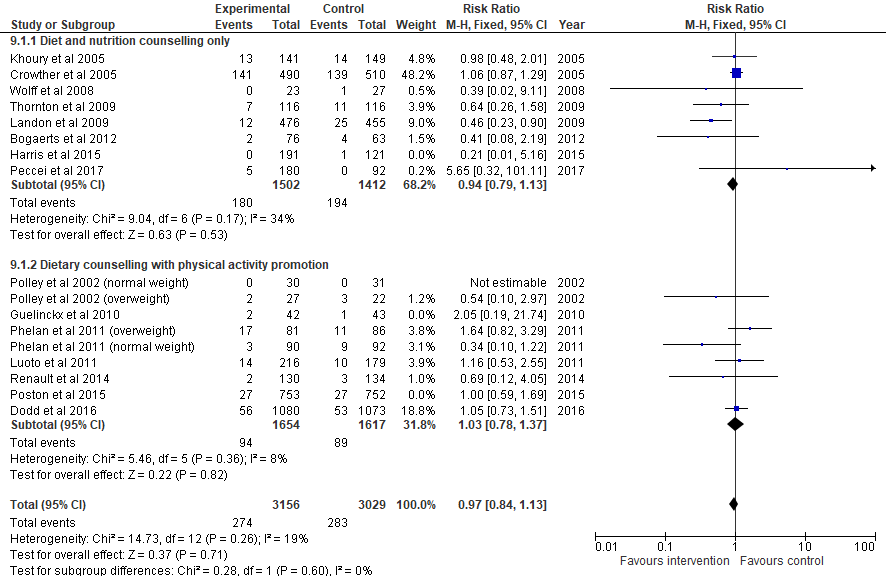


### 10.1.2 Small for gestational age


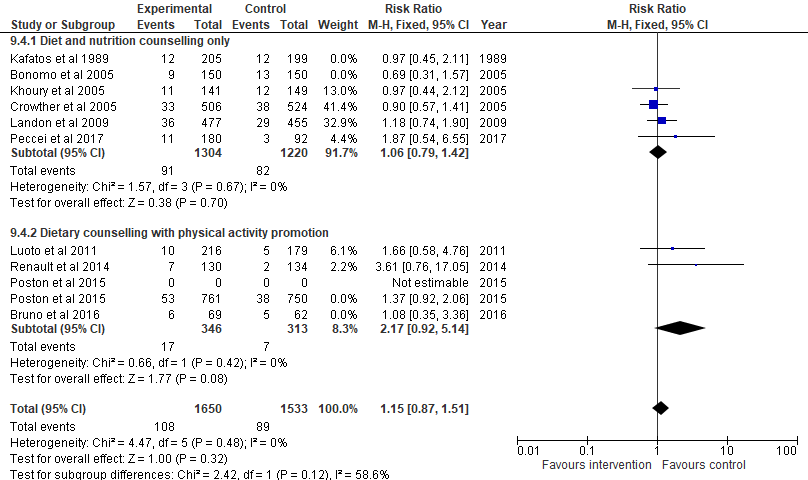


### 10.1.3 Low birthweight


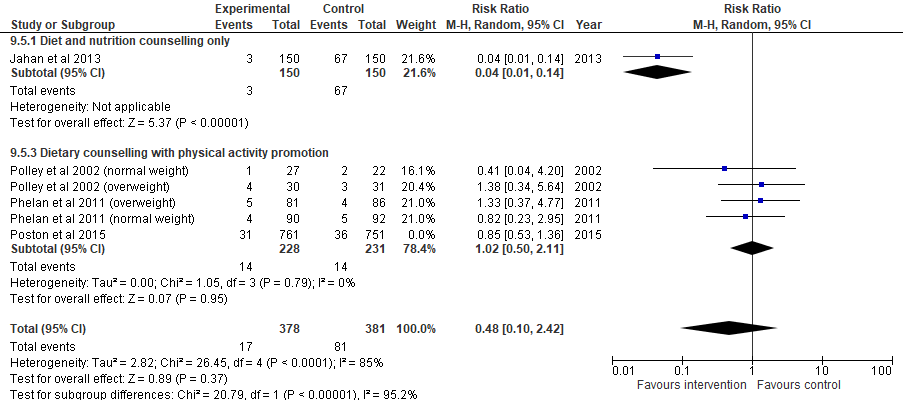


### 10.1.4 Preterm birth


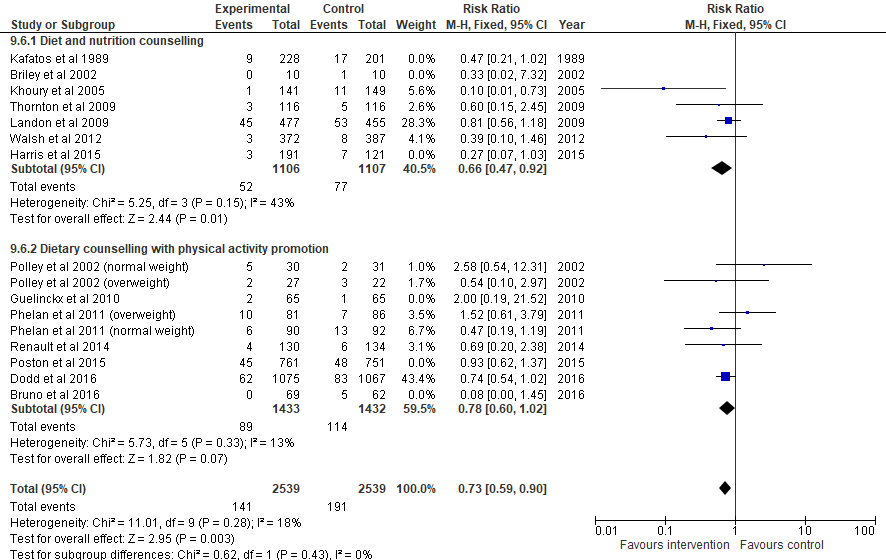


### 10.1.5 Stillbirth


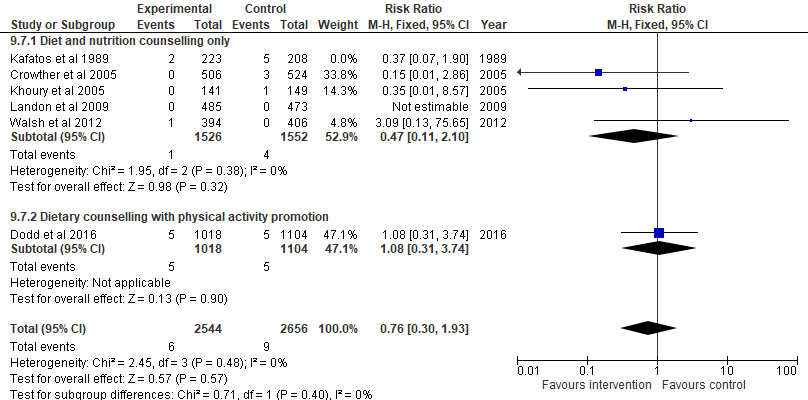


### 10.1.6 Maternal mortality

N/A – no change

## 10.2 LMIC studies only

### 10.2.1 Pre-eclampsia

N/A – all studies conducted in HICs

### 10.2.2 Small for gestational age

N/A – all studies conducted in HICs

### 10.2.3 Low birthweight


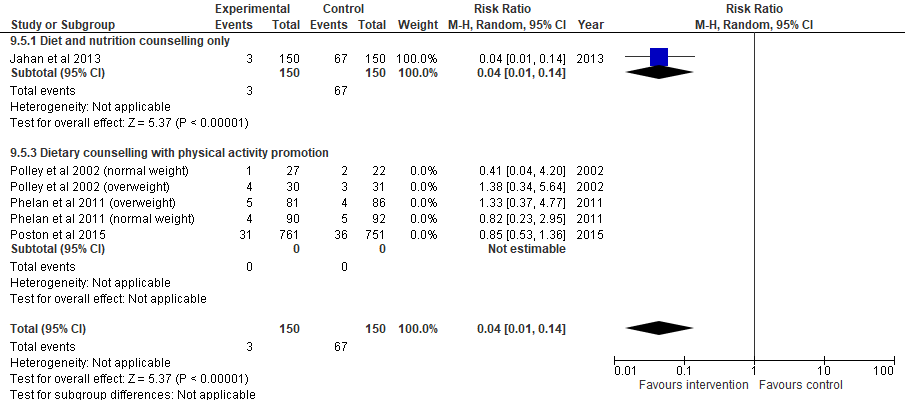


### 10.2.4 Preterm birth

N/A – all studies conducted in HICs

### 10.2.5 Stillbirth

N/A – all studies conducted in HICs

### 10.2.6 Maternal mortality

N/A – all studies conducted in HICs
