# Supplementary material for: Effects of Maternal Nutritional Supplements and Dietary Interventions on Placental Complications: An Umbrella Review, Meta-Analysis and Evidence Map
Source: Nutrients. 2021 Jan 30;13(2):472. doi: 10.3390/nu13020472 (PMC7912620; doi:10.3390/nu13020472)
Supplement: Supplementary file 1 [file nutrients-13-00472-s001.zip › Supplementary files/Table S10 - Risk of bias.docx]

**Table S10 – Risk of bias assessment**

**Risk of bias assessment reported from most recent, highest quality review (AMSTAR 2 high or moderate)*

| **Reference** | **Random sequence generation** | **Allocation concealment** | **Blinding of participants and personnel** | **Blinding of outcome assessment** | **Incomplete outcome data** | **Selective reporting** | **Other bias** | **Reporting review*** |
| --- | --- | --- | --- | --- | --- | --- | --- | --- |
| Abramovici et al 2015 | Low risk | Low risk | Low risk | Low risk | Low risk | Low risk | Low risk | See results for Roberts et al 2010 |
| Adu-Afarwuah et al 2015 (Dewey et al 2009) | Low risk | Low risk | Low risk | Low risk | Low risk | Low risk | Low risk | Keats et al 2019 |
| Aghamohammadi et al 2015 | Unclear risk | Unclear risk | High risk | High risk | High risk | High risk | Unclear risk | Khaing et al 2017 |
| Almirante 1998 | Unclear risk | Unclear risk | High risk | High risk | Unclear risk | Unclear risk | High risk | Hofymeyr et al 2018 |
| Asemi et al 2012 | Low risk | Low risk | High risk | High risk | Low risk | Unclear risk | Low risk | Palacios et al 2019 |
| Asemi et al 2013 | Low risk | Low risk | Low risk | Low risk | Low risk | Unclear risk | Low risk | Palacios et al 2019 |
| Asemi et al 2016 | Low risk | Low risk | Low risk | Low risk | Low risk | Low risk | Low risk | Fogacci et al 2019 |
| Ashorn et al 2015 (Ashorn et al 2010) | Low risk | Low risk | Low risk | Low risk | Low risk | Low risk | Low risk | Keats et al 2019 |
| Bassaw et al 1998 | Low risk | Low risk | Unclear risk | Low risk | High risk | Low risk | Low risk | Hofymeyr et al 2018 |
| Bastani et al 2011 | Low risk | Unclear risk | High risk | High risk | Unclear risk | Unclear risk | Unclear risk | Current review |
| Beazley et al 2005 | Unclear risk | Unclear risk | Unclear risk | Unclear risk | Low risk | Unclear risk | Unclear risk | Rumbold et al 2015a |
| Belizan et al 1991 | Low risk | Low risk | Low risk | Low risk | Low risk | Low risk | Low risk | Hofymeyr et al 2018 |
| Berghmann et al 2007 | Low risk | Unclear risk | Low risk | Low risk | Unclear risk | Low risk | Low risk | Middleton et al 2018 |
| Bhutta et al 2009 | Low risk | Low risk | Low risk | Low risk | Low risk | Low risk | Low risk | Keats et al 2019 |
| Bisgaard et al 2016 | Low risk | Low risk | Low risk | Low risk | Low risk | Low risk | Low risk | Middleton et al 2019 |
| Bogaerts et al 2012 | Selection bias: Low risk | NA | Unclear risk | Unclear risk | Low risk | Low risk | Unclear risk | Allen et al 2014 |
| Boggess et al 1997 | Low risk | Low risk | Low risk | Low risk | Unclear risk | Low risk | Low risk | Buppasiri et al 2015 |
| Bonomo et al 2005 | Low risk | Unclear risk | High risk | High risk | High risk | Low risk | Unclear risk | Current review |
| Briley et al 2002 | Unclear risk | Unclear risk | Unclear risk | High risk | High risk | High risk | Low risk | Current review |
| Brooke et al 1980 | Unclear risk | Unclear risk | Low risk | Unclear risk | High risk | Unclear risk | Low risk | Palacios et al 2019 |
| Brough et al 2010 | Unclear risk | Unclear risk | Low risk | Low risk | Low risk | Low risk | Low risk | Keats et al 2019 |
| Bruno et al 2016 | Low risk | Low risk | High risk | High risk | High risk | Low risk | Low risk | Current review |
| Bulstra-Ramakers et al 1994 | Unclear risk | Low risk | Low risk | Low risk | Unclear risk | Unclear risk | Unclear risk | Middleton et al 2019 |
| Carlson et al 2013 | Low risk | Low risk | Low risk | Low risk | Unclear risk | Low risk | Low risk | Middleton et al 2019 |
| Casanueva et al 2005 | Low risk | Low risk | Low risk | Unclear risk | Low risk | Unclear risk | Unclear risk | Rumbold et al 2015a |
| Castillo-Duran 2001 | Unclear risk | Unclear risk | Low risk | Unclear risk | High risk | Unclear risk | Low risk | Ota et al 2015 |
| Caulfield 1999 | Unclear risk | Low risk | Low risk | Low risk | Unclear risk | Unclear risk | Low risk | Ota et al 2015 |
| Chan et al 2009 | Low risk | Low risk | Unclear risk | Unclear risk | High risk | Unclear risk | Low risk | Pena-Rosas et al 2015 |
| Chappell et al 1999 | Low risk | Low risk | Low risk | Low risk | Low risk | Unclear risk | Low risk | Rumbold et al 2015a |
| Charles et al 2005 | Unclear risk | High risk | Low risk | Low risk | Unclear risk | Unclear risk | Unclear risk | Lassi et al 2013 |
| Chawes et al 2016 | Low risk | Low risk | Low risk | Low risk | Low risk | Low risk | Unclear risk | Bi et al 2018 |
| Cherry 1989 | Unclear risk | Unclear risk | Low risk | Low risk | Unclear risk | Unclear risk | Low risk | Ota et al 2015 |
| Christian et al 2003/ Christian et al 2009 | Low risk | Low risk | Low risk | Low risk | Low risk | Unclear risk | Low risk | Pena-Rosas et al 2015 |
| Cong et al 1995 | Unclear risk | Unclear risk | High risk | High risk | Unclear risk | Unclear risk | High risk | Hofymeyr et al 2018 |
| Cooper et al 2016 (Harvey et al 2012) | Low risk | Low risk | Low risk | Low risk | Low risk | Unclear risk | High risk | Palacios et al 2019 |
| Coutsoudis et al 1999 | Unclear risk | Unclear risk | Low risk | Unclear risk | Low risk | Unclear risk | Low risk | McCauley et al 2015 |
| Cox et al 2005 | Unclear risk | Unclear risk | Low risk | Unclear risk | Low risk | Unclear risk | Unclear risk | McCauley et al 2015 |
| Crowther et al 1999 | Low risk | Low risk | Low risk | Low risk | Low risk | Low risk | Unclear risk | Hofymeyr et al 2018 |
| Crowther et al 2005 | Selection bias: Low risk | NA | Unclear risk | Unclear risk | Low risk | Low risk | Unclear risk | Allen et al 2014 |
| D'Almedia et al 1992 | Low risk | Low risk | Low risk | Low risk | Unclear risk | Unclear risk | Unclear risk | Middleton et al 2019 |
| Danesh 2010 | Low risk | Low risk | Low risk | Unclear risk | High risk | Unclear risk | Unclear risk | Ota et al 2015 |
| Dawodu et al 2013 | Low risk | Low risk | Low risk | Low risk | Low risk | Low risk | Unclear risk | Bi et al 2018 |
| De Groot et al 2004 | Unclear risk | Unclear risk | Unclear risk | Unclear risk | High risk | Unclear risk | Unclear risk | Middleton et al 2019 |
| Delvin et al 1986 | Unclear risk | Unclear risk | High risk | Unclear risk | High risk | Unclear risk | Low risk | Palacios et al 2019 |
| Dijkhuizen et al 2004 | Unclear risk | Low risk | Low risk | Low risk | Low risk | Unclear risk | Low risk | McCauley et al 2015 |
| Dilli 2018 | Unclear risk | Unclear risk | Low risk | Unclear risk | High risk | Unclear risk | Unclear risk | Middleton et al 2019 |
| Diogenes et al 2013 | Low risk | Unclear risk | High risk | Unclear risk | High risk | Unclear risk | Unclear risk | Palacios et al 2019 |
| Dodd et al 2014 | Low risk | Low risk | Unclear risk | Low risk | Low risk | Low risk | Low risk | Current review |
| Dunstan et al 2003 (Dunstan et al 2008) | Unclear risk | Low risk | Low risk | Low risk | Unclear risk | Unclear risk | Unclear risk | Middleton et al 2019 |
| Eskeland et al 1997 | Low risk | Low risk | Low risk | Low risk | High risk | Unclear risk | Low risk | Pena-Rosas et al 2015 |
| Fawzi et al 1998 | Unclear risk | Low risk | Low risk | Low risk | Low risk | Unclear risk | Low risk | McCauley et al 2015 |
| Fawzi et al 2007 | Low risk | Low risk | Low risk | Low risk | Low risk | Low risk | Low risk | Keats et al 2019 |
| Fleming et al 1968 | High risk | Low risk | Low risk | Low risk | Low risk | Unclear risk | Unclear risk | Lassi et al 2013 |
| Friis et al 2004 | Low risk | Low risk | Low risk | Low risk | Low risk | Low risk | Low risk | Keats et al 2019 |
| Goldberg et al 2013 | Low risk | Low risk | Low risk | Low risk | High risk | Low risk | Low risk | Buppasiri et al 2015 |
| Goldenberg 1995 | Low risk | Unclear risk | Low risk | Low risk | Unclear risk | Unclear risk | Low risk | Ota et al 2015 |
| Grant et al 2013 | Low risk | Low risk | Low risk | Low risk | Low risk | Unclear risk | Low risk | Palacios et al 2019 |
| Guelinckx et al 2010 | Selection bias: Unclear risk | NA | Unclear risk | Unclear risk | Low risk | High risk | Unclear risk | Allen et al 2014 |
| Gupta et al 2007 | Low risk | Low risk | Low risk | Low risk | Unclear risk | Low risk | Unclear risk | Current review |
| Hafeez 2005 | Unclear risk | Unclear risk | Low risk | Low risk | Unclear risk | Unclear risk | Low risk | Ota et al 2015 |
| Haghiac et al 2015 | Low risk | Low risk | Low risk | Low risk | High risk | Unclear risk | Unclear risk | Middleton et al 2019 |
| Harper 2010 | Low risk | Low risk | Low risk | Low risk | Low risk | Low risk | Low risk | Middleton et al 2019 |
| Harris et al 2015 | Low risk | Low risk | Low risk | Low risk | High risk | Unclear risk | Low risk | Middleton et al 2019 |
| Harvey et al 2007 | Low risk | Low risk | Unclear risk | Low risk | Low risk | Unclear risk | Unclear risk | Pena-Rosas et al 2015 |
| Hashemipour et al 2014 | Low risk | Unclear risk | High risk | Unclear risk | Low risk | Low risk | Unclear risk | Bi et al 2018 |
| Hauner 2012 | Low risk | Unclear risk | High risk | High risk | High risk | Unclear risk | Unclear risk | Middleton et al 2019 |
| Helland 2001 | Low risk | Unclear risk | Low risk | Low risk | High risk | Unclear risk | Unclear risk | Middleton et al 2019 |
| Hererra et al 1998 | Low risk | Low risk | Low risk | Low risk | Low risk | Low risk | Low risk | Hofymeyr et al 2018 |
| Hererra et al 2006 | Low risk | Low risk | Unclear risk | Unclear risk | Low risk | Unclear risk | Low risk | Hofymeyr et al 2018 |
| Hofymeyr et al 2019 | Low risk | Low risk | Low risk | Low risk | Low risk | Low risk | Unclear risk | Hofymeyr et al 2019 |
| Horvaticek 2017 | Unclear risk | Unclear risk | Low risk | Unclear risk | High risk | High risk | Unclear risk | Middleton et al 2019 |
| Hossain et al 2014 | High risk | Unclear risk | High risk | Unclear risk | Low risk | Low risk | Unclear risk | Bi et al 2018 |
| Hunt 1984 | Unclear risk | Low risk | Low risk | Unclear risk | Unclear risk | Unclear risk | Low risk | Ota et al 2015 |
| Huria et al 2010 | Unclear risk | Unclear risk | Unclear risk | Unclear risk | High risk | Unclear risk | Unclear risk | Rumbold et al 2015a |
| Huybregts et al 2009 | Low risk | Low risk | Unclear risk | Unclear risk | High risk | Low risk | Low risk | Das et al 2018 |
| ICMR 2000 | Unclear risk | Low risk | Low risk | Unclear risk | Unclear risk | Unclear risk | Unclear risk | De-Regil et al 2015 |
| Jahan et al 2013 | Low risk | Unclear risk | High risk | Unclear risk | High risk | Unclear risk | Unclear risk | Ota et al 2015 |
| Jamilian et al 2016 | Low risk | Low risk | Low risk | Low risk | Unclear risk | Low risk | Low risk | Middleton et al 2019 |
| Jamilian et al 2018 | Low risk | Low risk | Low risk | Low risk | Low risk | Low risk | Low risk | Fogacci et al 2019 |
| Johnson et al 2017 (Moore et al 2009) | Low risk | Low risk | Unclear risk | Low risk | High risk | Low risk | Low risk | Keats et al 2019 |
| Jonsson 1996 | Unclear risk | Unclear risk | Low risk | Low risk | High risk | Unclear risk | Unclear risk | Ota et al 2015 |
| Kæstel et al 2005 | Low risk | Unclear risk | Low risk | Low risk | High risk | Low risk | Low risk | Keats et al 2019 |
| Kafatos et al 1989 | Low risk | Low risk | High risk | High risk | Low risk | Unclear risk | High risk | Ota et al 2015 |
| Kalpdev et al 2011 | Low risk | Unclear risk | High risk | Unclear risk | Low risk | Unclear risk | Low risk | Rumbold et al 2015a |
| Karamali et al 2015 | Low risk | Low risk | Low risk | Low risk | Low risk | Low risk | Unclear risk | Bi et al 2018 |
| Khan et al 2011 (Bhutta et al 2011) | Low risk | Low risk | Low risk | Low risk | Low risk | Unclear risk | Low risk | Palacios et al 2019 |
| Khan et al 2013 | Low risk | Unclear risk | Unclear risk | Unclear risk | Low risk | Unclear risk | Unclear risk | Hofymeyr et al 2019 |
| Khoury et al 2005 | Selection bias: Low risk | NA | Low risk | Low risk | Unclear risk | Unclear risk | Unclear risk | Allen et al 2014 |
| Kiondo et al 2014 | Low risk | Low risk | Low risk | Low risk | Low risk | Low risk | Low risk | Rumbold et al 2015a |
| Kirke 1992 | Low risk | Low risk | Low risk | Unclear risk | Unclear risk | Unclear risk | Unclear risk | De-Regil et al 2015 |
| Kirkwood et al 2010 | Low risk | Low risk | Low risk | Unclear risk | Low risk | Unclear risk | Low risk | McCauley et al 2015 |
| Kumar et al 2009 | Unclear risk | Low risk | Low risk | Low risk | Low risk | Low risk | High risk | Hofymeyr et al 2019 |
| Kumwenda et al 2002 | Low risk | Low risk | Low risk | Unclear risk | Low risk | Unclear risk | Low risk | McCauley et al 2015 |
| Lalooha et al 2012/ Ranjkesh et al 2011 | Unclear risk | Unclear risk | Low risk | Unclear risk | Low risk | Unclear risk | Low risk | Middleton et al 2019 |
| Landon et al 2009 | Selection bias: Low risk | NA | Unclear risk | Unclear risk | Low risk | Low risk | Unclear risk | Allen et al 2014 |
| Lee et al 2005 | Low risk | Unclear risk | High risk | Low risk | Low risk | Unclear risk | Unclear risk | Pena-Rosas et al 2015 |
| Levine et al 1997 | Low risk | Low risk | Low risk | Low risk | Low risk | Unclear risk | Low risk | Hofymeyr et al 2019 |
| Li et al 2000 | Unclear risk | Unclear risk | High risk | High risk | Unclear risk | Unclear risk | High risk | Hofymeyr et al 2019 |
| Litonjua et al 2016 | Low risk | Low risk | Low risk | Low risk | Low risk | Low risk | Unclear risk | Bi et al 2018 |
| Liu et al 2000 | Unclear risk | Unclear risk | Unclear risk | Low risk | Low risk | Unclear risk | Low risk | Pena-Rosas et al 2015 |
| Liu et al 2012 | Low risk | Low risk | Low risk | Low risk | Low risk | Low risk | Low risk | Pena-Rosas et al 2015 |
| Lopez Jaramillo et al 1989 | Unclear risk | Unclear risk | Unclear risk | Unclear risk | High risk | Unclear risk | Unclear risk | Hofymeyr et al 2019 |
| Lopez Jaramillo et al 1990 | Unclear risk | Unclear risk | Unclear risk | Unclear risk | Unclear risk | Unclear risk | Unclear risk | Hofymeyr et al 2019 |
| Lopez Jaramillo et al 1997 | Low risk | Low risk | Low risk | Low risk | Unclear risk | Unclear risk | Unclear risk | Hofymeyr et al 2019 |
| Luoto et al 2011 | Unclear risk | Unclear risk | Unclear risk | Unclear risk | Low risk | Low risk | Unclear risk | Current review |
| Mahdy et al 2013 | Low risk | Low risk | Low risk | Low risk | High risk | Unclear risk | Low risk | Current review |
| Mahomed 1989 | Low risk | Low risk | Low risk | Low risk | Low risk | Low risk | Low risk | Ota et al 2015 |
| Makrides et al 2003 | Low risk | Low risk | Low risk | Low risk | Low risk | Unclear risk | Unclear risk | Pena-Rosas et al 2015 |
| Makrides et al 2010 / Zhao et al 2012 | Low risk | Low risk | Low risk | Low risk | Low risk | Low risk | Low risk | Middleton et al 2019 |
| Malcolm 2003 | Unclear risk | Unclear risk | Low risk | Unclear risk | High risk | Unclear risk | Unclear risk | Middleton et al 2019 |
| Mardones 2008 | High risk | High risk | Unclear risk | Unclear risk | High risk | Unclear risk | Unclear risk | Middleton et al 2019 |
| Marya et al 1987 | Unclear risk | Unclear risk | High risk | Unclear risk | High risk | High risk | Low risk | Palacios et al 2019 |
| Marya et al 1988 | Unclear risk | Unclear risk | High risk | Unclear risk | Unclear risk | Unclear risk | Low risk | Palacios et al 2019 |
| McCance et al 2010 | Low risk | Low risk | Low risk | Low risk | Low risk | Low risk | Low risk | Rumbold et al 2015a |
| McEvoy et al 2014 | Unclear risk | Unclear risk | Low risk | Low risk | Low risk | Low risk | Low risk | Rumbold et al 2015a |
| Meier et al 2003 | Low risk | Unclear risk | Low risk | Low risk | High risk | Unclear risk | Unclear risk | Pena-Rosas et al 2015 |
| Mendendez et al 1994 | Unclear risk | High risk | High risk | Low risk | High risk | Unclear risk | Unclear risk | Pena-Rosas et al 2015 |
| Merialdi 2004 | Low risk | Low risk | Low risk | Low risk | Low risk | High risk | Low risk | Ota et al 2015 |
| Miller 2016 | Low risk | Unclear risk | Low risk | Low risk | Unclear risk | Unclear risk | Unclear risk | Middleton et al 2019 |
| Min 2014 | Low risk | Low risk | Low risk | Low risk | High risk | Unclear risk | Unclear risk | Middleton et al 2019 |
| Min 2016 | Low risk | Low risk | Low risk | Low risk | Unclear risk | Unclear risk | Unclear risk | Middleton et al 2019 |
| Mohammad-Alizadeh-Charandabi et al 2015 (  Mirghafourvand et al 2013) | Low risk | Unclear risk | Low risk | Unclear risk | Low risk | Unclear risk | Low risk | Palacios et al 2019 |
| Mojibian et al. 2015 | Low risk | Low risk | High risk | Low risk | Low risk | Low risk | Unclear risk | Bi et al 2018 |
| Mozurkewich 2013 | Low risk | Low risk | Low risk | Unclear risk | Low risk | Low risk | Low risk | Middleton et al 2019 |
| MRC 1991 | Unclear risk | Unclear risk | Low risk | Unclear risk | Unclear risk | Unclear risk | Unclear risk | De-Regil et al 2015 |
| Mridha et al 2016 | Low risk | Low risk | Unclear risk | Low risk | Low risk | Low risk | Low risk | Das et al 2018 |
| Naghshineh and Sheikhaliyan 2016 | Low risk | Unclear risk | Low risk | Low risk | Low risk | Unclear risk | Low risk | Palacios et al 2019 |
| Nasrolahi et al 2006 | High risk | High risk | High risk | Unclear risk | Low risk | Unclear risk | Unclear risk | Rumbold et al 2015a |
| Ndyomugyenyi and Magnussen 2000 | Unclear risk | Unclear risk | Low risk | Low risk | High risk | Unclear risk | Unclear risk | Current review |
| Nenad et al 2011 | Unclear risk | Unclear risk | High risk | High risk | Unclear risk | Unclear risk | High risk | Khaing et al 2017 |
| Niromanesh et al 2001 | Unclear risk | Low risk | Low risk | Low risk | Low risk | Low risk | Unclear risk | Hofymeyr et al 2019 |
| Olsen 1992 / Sorensen et al 1993 / Salvig et al 1996 | Unclear risk | Low risk | Unclear risk | Unclear risk | Low risk | Low risk | Low risk | Middleton et al 2019 |
| Olsen 2000 | Low risk | Low risk | Low risk | Low risk | Low risk | Unclear risk | Low risk | Middleton et al 2019 |
| Onwude 1995 | Low risk | Low risk | Low risk | Low risk | Low risk | High risk | Low risk | Middleton et al 2019 |
| Osendarp 2000 | Low risk | Unclear risk | Low risk | Low risk | Unclear risk | Unclear risk | Low risk | Ota et al 2015 |
| Osrin et al 2005 | Low risk | Low risk | Low risk | Low risk | High risk | Low risk | Low risk | Keats et al 2019 |
| Ostadrahimi et al 2017 (Khalili et al 2016) | Low risk | Low risk | Low risk | Low risk | Unclear risk | Low risk | Low risk | Middleton et al 2019 |
| Ouladsahebmadarek et al 2011 | Unclear risk | Unclear risk | Low risk | Low risk | Low risk | Unclear risk | Unclear risk | Pena-Rosas et al 2015 |
| Peccei et al 2017 | Low risk | Unclear risk | Unclear risk | Unclear risk | Low risk | Low risk | Unclear risk | Current review |
| Phelan et al 2011 | Low risk | Low risk | Low risk | Unclear risk | Low risk | Low risk | Unclear risk | Allen et al 2014 |
| Polley et al 2002 | Unclear risk | Unclear risk | Unclear risk | Unclear risk | Low risk | Low risk | Unclear risk | Allen et al 2014 |
| Poston et al 2006 | Low risk | Low risk | Low risk | Low risk | Low risk | Low risk | Low risk | Rumbold et al 2015a |
| Poston et al 2015 | Low risk | Unclear risk | High risk | High risk | Low risk | Low risk | Low risk | Current review |
| Purwar et al 1996 | Low risk | Low risk | Low risk | Low risk | Low risk | Low risk | Unclear risk | Hofymeyr et al 2019 |
| Radhika et al 2003 | Unclear risk | Unclear risk | Low risk | Unclear risk | Low risk | Unclear risk | Low risk | McCauley et al 2015 |
| Ramakrishan et al 2003 | Low risk | Low risk | Low risk | Low risk | High risk | Low risk | Low risk | Keats et al 2019 |
| Ramakrishnan 2010 | Low risk | Low risk | Low risk | Low risk | Unclear risk | Unclear risk | Low risk | Middleton et al 2019 |
| Razavi et al 2017 | Low risk | Low risk | Low risk | Low risk | Low risk | Unclear risk | Low risk | Middleton et al 2019 |
| Renault et al 2014 | Low risk | Low risk | Unclear risk | Unclear risk | Low risk | Low risk | Low risk | Current review |
| Roberfroid et al 2008 | Low risk | Low risk | Low risk | Low risk | Low risk | Low risk | Low risk | Keats et al 2019 |
| Roberts et al 2010 | Low risk | Low risk | Low risk | Low risk | Low risk | Low risk | Low risk | Rumbold et al 2015a |
| Robertson 1991 | Unclear risk | Unclear risk | Low risk | Unclear risk | Unclear risk | High risk | Low risk | Ota et al 2015 |
| Roger et al 1999 | High risk | High risk | High risk | High risk | High risk | Unclear risk | High risk | Hofymeyr et al 2019 |
| Roth et al 2013 (Roth et al 2010) | Low risk | Low risk | Low risk | Low risk | Low risk | Unclear risk | Low risk | Palacios et al 2019 |
| Rumbold et al 2010 | Low risk | Low risk | Low risk | Low risk | Low risk | Low risk | Low risk | Rumbold et al 2015a |
| Rumiris et al 2006 | Low risk | Low risk | Low risk | Low risk | Low risk | Unclear risk | Low risk | Hofymeyr et al 2018 |
| Saaka 2009 | Low risk | Unclear risk | Low risk | Unclear risk | Unclear risk | Unclear risk | Low risk | Ota et al 2015 |
| Sablok et al 2015 | Low risk | High risk | High risk | Unclear risk | High risk | Unclear risk | Low risk | Palacios et al 2019 |
| Samimi et al 2016 | Low risk | Low risk | Low risk | Unclear risk | Low risk | Unclear risk | Low risk | Palacios et al 2019 |
| Sanchez-Ramos et al 1994 | Low risk | Low risk | Low risk | Low risk | Low risk | Low risk | Low risk | Hofymeyr et al 2018 |
| Sasan et al 2017 | Low risk | Unclear risk | Low risk | Low risk | Low risk | Unclear risk | Low risk | Palacios et al 2019 |
| Shankar et al 2008 | Low risk | Low risk | Low risk | Low risk | Low risk | Low risk | Low risk | Keats et al 2019 |
| Siega-Riz et al 2001 | Low risk | Low risk | Low risk | Low risk | High risk | Unclear risk | Unclear risk | Pena-Rosas et al 2015 |
| Simmer 1991 | Low risk | Unclear risk | Low risk | Low risk | Low risk | Unclear risk | Low risk | Ota et al 2015 |
| Singh et al 2015 | Unclear risk | Unclear risk | High risk | Unclear risk | High risk | Unclear risk | Low risk | Palacios et al 2019 |
| Smuts et al 2003a | Low risk | Unclear risk | Low risk | Low risk | Unclear risk | Low risk | Low risk | Middleton et al 2019 |
| Smuts et al 2003b | Unclear risk | Unclear risk | Unclear risk | Unclear risk | High risk | Unclear risk | Unclear risk | Middleton et al 2019 |
| Spinnato II et al 2007 | Low risk | Low risk | Low risk | Low risk | Low risk | Low risk | Low risk | Rumbold et al 2015a |
| Steyn et al 2003 | Low risk | Low risk | Low risk | Unclear risk | Low risk | Unclear risk | Low risk | Rumbold et al 2015a |
| Sunawang et al 2009 | Unclear risk | Unclear risk | Unclear risk | Unclear risk | Low risk | Low risk | Low risk | Keats et al 2019 |
| Taghizadeh 2016/ Jamilian et al 2016b | Low risk | Low risk | Low risk | Low risk | Unclear risk | Low risk | Low risk | Middleton et al 2019 |
| Taghriri and Danesh 2007 | High risk | High risk | High risk | High risk | Unclear risk | High risk | Unclear risk | Current review |
| Taherian et al 2002 | Low risk | Unclear risk | High risk | Unclear risk | Low risk | Unclear risk | Low risk | Palacios et al 2019 |
| Taylor et al 1982 | Unclear risk | Unclear risk | High risk | Low risk | Low risk | Unclear risk | Unclear risk | Pena-Rosas et al 2015 |
| Thornton et al 2009 | Low risk | Unclear risk | Unclear risk | Unclear risk | Low risk | Unclear risk | Unclear risk | Allen et al 2014 |
| Tofail et al 2006/ Tofail et al 2012 | Unclear risk | Unclear risk | Low risk | Low risk | High risk | Low risk | Unclear risk | Middleton et al 2019 |
| Tofail et al 2008 / Persson et al 2012 | Unclear risk | Unclear risk | Low risk | Low risk | High risk | Low risk | Low risk | Keats et al 2019 |
| Vadillo-Ortega et al 2011 | Low risk | Low risk | Low risk | Unclear risk | Low risk | High risk | Unclear risk | Current review |
| Valizadeh et al 2016 | Unclear risk | Low risk | High risk | High risk | High risk | Low risk | Low risk | Current review |
| Van den Broek et al 2006 | Low risk | Low risk | Low risk | Low risk | Low risk | Unclear risk | Low risk | McCauley et al 2015 |
| Van Goor et al 2010 (Van Goor et al 2009) | Unclear risk | Unclear risk | Low risk | Unclear risk | High risk | Low risk | Unclear risk | Middleton et al 2019 |
| Villar et al 1987 | Unclear risk | Low risk | Low risk | Low risk | Low risk | Low risk | Unclear risk | Hofymeyr et al 2018 |
| Villar et al 1990 | Low risk | Low risk | Low risk | Low risk | Low risk | Low risk | Unclear risk | Hofymeyr et al 2018 |
| Villar et al 2006 | Low risk | Low risk | Low risk | Low risk | Low risk | Low risk | Low risk | Hofymeyr et al 2018 |
| Villar et al 2009 | Low risk | Low risk | Low risk | Low risk | Low risk | Low risk | Low risk | Rumbold et al 2015a |
| Walsh et al 2012 | Low risk | Unclear risk | Unclear risk | Unclear risk | Low risk | Low risk | Low risk | Current review |
| Wanchu et al 2001 | Unclear risk | Unclear risk | High risk | High risk | High risk | High risk | Unclear risk | Khaing et al 2017 |
| West et al 1999 | Low risk | Low risk | Low risk | Unclear risk | Low risk | Unclear risk | Low risk | McCauley et al 2015 |
| West et al 2011 | Low risk | Low risk | Low risk | Low risk | Low risk | Low risk | Low risk | McCauley et al 2015 |
| West et al 2014 | Low risk | Low risk | Low risk | Low risk | Low risk | Low risk | Low risk | Keats et al 2019 |
| Wolff et al 2008 | Low risk | Unclear risk | Unclear risk | Unclear risk | Low risk | Unclear risk | Unclear risk | Allen et al 2014 |
| Xie 2001 | Unclear risk | Low risk | Low risk | Unclear risk | Low risk | Unclear risk | Unclear risk | Ota et al 2015 |
| Xu et al 2010 | Low risk | Low risk | Low risk | Low risk | Low risk | Low risk | Unclear risk | Rumbold et al 2015a |
| Yap et al 2014 | Low risk | Low risk | Low risk | Low risk | Low risk | Low risk | Unclear risk | Bi et al 2018 |
| Yu et al 2009 (Yu et al 2008) | Low risk | Low risk | High risk | High risk | Low risk | Unclear risk | Unclear risk | Palacios et al 2019 |
| Zagre et al 2007 | Unclear risk | Unclear risk | Low risk | Low risk | High risk | Low risk | Low risk | Keats et al 2019 |
| Zeng et al 2009 | Low risk | Low risk | Unclear risk | Unclear risk | Low risk | Unclear risk | Unclear risk | Pena-Rosas et al 2015 |
| Zerofsky et al 2014 | Low risk | Low risk | Low risk | Low risk | Low risk | Low risk | Unclear risk | Bi et al 2018 |
| Ziaei et al 2007 | Low risk | Low risk | Low risk | Low risk | Low risk | Unclear risk | Unclear risk | Pena-Rosas et al 2015 |
