# Supplementary material for: Effects of Maternal Nutritional Supplements and Dietary Interventions on Placental Complications: An Umbrella Review, Meta-Analysis and Evidence Map
Source: Nutrients. 2021 Jan 30;13(2):472. doi: 10.3390/nu13020472 (PMC7912620; doi:10.3390/nu13020472)
Supplement: Supplementary file 1 [file nutrients-13-00472-s001.zip › Supplementary files/Table S11 - GRADE.docx]

**Table S12 – GRADE Scale for Quality Assessment of Evidence**

Contents

[1. Vitamin A compared to control 2](#_Toc55379660)

[2. Vitamin B6 compared to control 3](#_Toc55379661)

[3. Vitamin C and/or E compared to control 3](#_Toc55379662)

[3. Vitamin D compared to control 4](#_Toc55379663)

[4. Vitamin D and calcium compared to control 5](#_Toc55379664)

[5. Calcium compared to control 6](#_Toc55379665)

[6. Iodine compared to control 7](#_Toc55379666)

[7. Iron and/or folic acid compared to control 8](#_Toc55379667)

[8. Magnesium compared to control 9](#_Toc55379668)

[9. Zinc compared to control 10](#_Toc55379669)

[10. Garlic compared to control 11](#_Toc55379670)

[11. Multiple micronutrients compared to control 12](#_Toc55379671)

[12. Lipid-based nutrients compared to control 13](#_Toc55379672)

[13. Balanced protein/energy compared to control 14](#_Toc55379673)

[14. High protein compared to control 15](#_Toc55379674)

[15. Calf blood extract compared to control 15](#_Toc55379675)

[16. Glucose compared to control 16](#_Toc55379676)

[17. Galactose compared to control 16](#_Toc55379677)

[18. Polyunsaturated omega-3 fatty acid compared to control 17](#_Toc55379678)

[19. Salt restriction compared to control 18](#_Toc55379679)

[20. Caffeine restriction compared to control 19](#_Toc55379680)

[21. Antenatal dietary counselling compared to control 19](#_Toc55379681)

# 1. Vitamin A compared to control

| **Outcome** | **Certainty assessment** | | | | | | | **Effect** | **Certainty** |
| --- | --- | --- | --- | --- | --- | --- | --- | --- | --- |
|  | **Number of studies** | **Study design** | **Risk of bias** | **Inconsistency** | **Indirectness** | **Imprecision** | **Publication bias** | **Relative (95% CI)** |  |
| PE | 0 | N/A | N/A | N/A | N/A | N/A | N/A | N/A | N/A |
| SGA | 3 | RCTs | Not serious (all studies low/unclear RoB) | Not serious  (I^2^=0%) | Not serious | Serious (large sample but 95%CI crosses 1) | N/A | RR 1.00 (0.98-1.03) | Moderate |
| LBW | 6 | RCTs | Not serious (all studies low/unclear RoB) | Serious  (I^2^=51%) | Not serious | Serious (large sample but 95%CI crosses 1) | N/A | RR 0.92 (0.75-1.13) | Low |
| PTB | 6 | RCTs | Not serious (all studies low/unclear RoB) | Not serious  (I^2^=20%) | Not serious | Serious (large sample but 95%CI crosses 1) | N/A | RR 0.98 (0.94-1.01) | Moderate |
| Stillbirth | 4 | RCTs | Not serious (all studies low/unclear RoB) | Not serious  (I^2^=0%) | Not serious | Serious (large sample but 95%CI crosses 1) | N/A | RR 0.97 (0.92-1.03) | Moderate |
| Maternal mortality | 5 | RCTs | Not serious (all studies low/unclear RoB) | Serious  (I^2^=62%) | Not serious | Serious (large sample but 95%CI crosses 1) | N/A | RR 0.82 (0.56-1.19) | Low |

*PE – pre-eclampsia; SGA – small for gestational age; LBW – low birthweight; PTB – preterm birth; RCTs – randomized controlled trials; RoB – risk of bias*

# 2. Vitamin B6 compared to control

| **Outcome** | **Certainty assessment** | | | | | | | **Effect** | **Certainty** |
| --- | --- | --- | --- | --- | --- | --- | --- | --- | --- |
|  | **Number of studies** | **Study design** | **Risk of bias** | **Inconsistency** | **Indirectness** | **Imprecision** | **Publication bias** | **Relative (95% CI)** |  |
| PE | 2 | RCTs | Serious (Less than half low/unclear RoB) | N/A (only one trial with estimable outcomes) | Not serious | Serious (large sample but 95%CI crosses 1) | N/A | RR 1.71 (0.85-3.45) | Low |
| SGA | 0 | N/A | N/A | N/A | N/A | N/A | N/A | N/A | N/A |
| LBW | 0 | N/A | N/A | N/A | N/A | N/A | N/A | N/A | N/A |
| PTB | 0 | N/A | N/A | N/A | N/A | N/A | N/A | N/A | N/A |
| Stillbirth | 0 | N/A | N/A | N/A | N/A | N/A | N/A | N/A | N/A |
| Maternal mortality | 0 | N/A | N/A | N/A | N/A | N/A | N/A | N/A | N/A |

*PE – pre-eclampsia; SGA – small for gestational age; LBW – low birthweight; PTB – preterm birth; RCTs – randomized controlled trials; RoB – risk of bias*

# 3. Vitamin C and/or E compared to control

| **Outcome** | **Certainty assessment** | | | | | | | **Effect** | **Certainty** |
| --- | --- | --- | --- | --- | --- | --- | --- | --- | --- |
|  | **Number of studies** | **Study design** | **Risk of bias** | **Inconsistency** | **Indirectness** | **Imprecision** | **Publication bias** | **Relative (95% CI)** |  |
| PE | 19 | RCTs | Not serious (majority studies low/unclear RoB) | Not serious  (I^2^ 33%) | Not serious | Serious (large sample but 95%CI crosses 1) | Serious (asymmetrical funnel plot) | RR 0.96 (0.89-1.04 | Low |
| SGA | 13 | RCTs | Not serious (majority studies low/unclear RoB) | Not serious  (I^2^ 24%) | Not serious | Serious (large sample but 95%CI crosses 1) | Not serious (symmetrical funnel plot) | RR 0.96 (0.89-1.03 | Moderate |
| LBW | 7 | RCTs | Not serious (majority studies low/unclear RoB) | Serious  (I^2^ 86%) | Not serious | Serious (large sample but 95%CI crosses 1) | N/A | RR 0.93 (0.73-1.19 | Low |
| PTB | 17 | RCTs | Not serious (majority studies low/unclear RoB) | Serious  (I^2^ 53%) | Not serious | Serious (large sample but 95%CI crosses 1) | Not serious (symmetrical funnel plot) | RR 1.00 (0.90-1.11 | Low |
| Stillbirth | 9 | RCTs | Not serious (all studies low/unclear RoB) | Not serious  (I^2^ 0%) | Not serious | Serious (large sample but 95%CI crosses 1) | N/A | RR 1.21 (0.92-1.58 | Moderate |
| Maternal mortality | 6 | RCTs | Not serious (all studies low/unclear RoB) | Not serious  (I^2^ 0%) | Not serious | Serious (large sample but 95%CI crosses 1) | N/A | RR 0.60 (0.14-2.52) | Moderate |

*PE – pre-eclampsia; SGA – small for gestational age; LBW – low birthweight; PTB – preterm birth; RCTs – randomized controlled trials; RoB – risk of bias*

# 3. Vitamin D compared to control

| **Outcome** | **Certainty assessment** | | | | | | | **Effect** | **Certainty** |
| --- | --- | --- | --- | --- | --- | --- | --- | --- | --- |
|  | **Number of studies** | **Study design** | **Risk of bias** | **Inconsistency** | **Indirectness** | **Imprecision** | **Publication bias** | **Relative (95% CI)** |  |
| PE | 12 | RCTs | Not serious (majority studies low/unclear RoB) | Not serious  (I^2^ 0%) | Not serious | Not serious (large sample, 95%CI does not cross 1) | Not serious (symmetrical funnel plot) | RR 0.62 (0.43-0.91) | High |
| SGA | 5 | RCTs | Serious (Less than half low/unclear RoB) | Not serious  (I^2^ 0%) | Not serious | Serious (sample <1,000, 95%CI does not cross 1) | N/A | RR 0.59 (0.39-0.88) | Low |
| LBW | 6 | RCTs | Not serious (majority studies low/unclear RoB) | Serious  (I^2^ 64%) | Not serious | Serious (sample <1,000, 95%CI crosses 1) | N/A | RR 0.76 (0.54-1.06) | Low |
| PTB | 17 | RCTs | Not serious (majority studies low/unclear RoB) | Not serious  (I^2^ 39%) | Not serious | Serious (sample <1,000, 95%CI crosses 1) | Not serious (symmetrical funnel plot) | RR 0.70 (0.49- 1.00) | Moderate |
| Stillbirth | 5 | RCTs | Not serious (majority studies low/unclear RoB) | Not serious  (I^2^ 0%) | Not serious | Serious (sample <1,000, 95%CI crosses 1) | N/A | RR 0.62 (0.19-2.00) | Moderate |
| Maternal mortality | 0 | N/A | N/A | N/A | N/A | N/A | N/A | N/A | N/A |

*PE – pre-eclampsia; SGA – small for gestational age; LBW – low birthweight; PTB – preterm birth; RCTs – randomized controlled trials; RoB – risk of bias*

# 4. Vitamin D and calcium compared to control

| **Outcome** | **Certainty assessment** | | | | | | | **Effect** | **Certainty** |
| --- | --- | --- | --- | --- | --- | --- | --- | --- | --- |
|  | **Number of studies** | **Study design** | **Risk of bias** | **Inconsistency** | **Indirectness** | **Imprecision** | **Publication bias** | **Relative (95% CI)** |  |
| PE | 3 | RCTs | Serious (Less than half low/unclear RoB) | Not serious  (I^2^ 0%) | Not serious | Not serious (large sample, 95%CI does not cross 1) | N/A | RR 0.49 (0.31-0.77) | High |
| SGA | 1 | RCTs | Serious (None low/unclear RoB) | N/A (only one trial | Not serious | Serious (sample <1,000, 95%CI crosses 1) | N/A | RR 0.90 (0.58-1.38) | Low |
| LBW | 2 | RCTs | Serious (Less than half low/unclear RoB) | Not serious  (I^2 16^%) | Not serious | Serious (sample <1,000, 95%CI crosses 1) | N/A | RR 0.63 (0.12-3.24) | Low |
| PTB | 6 | RCTs | Not serious (Majority studies low/unclear RoB) | Not serious  (I^2^ 0%) | Not serious | Serious (sample <1,000, 95%CI does not cross 1) | N/A | RR 1.53 (1.02-2.30) | Moderate |
| Stillbirth | 0 | N/A | N/A | N/A | N/A | N/A | N/A | N/A | N/A |
| Maternal mortality | 0 | N/A | N/A | N/A | N/A | N/A | N/A | N/A | N/A |

*PE – pre-eclampsia; SGA – small for gestational age; LBW – low birthweight; PTB – preterm birth; RCTs – randomized controlled trials; RoB – risk of bias*

# 5. Calcium compared to control

| **Outcome** | **Certainty assessment** | | | | | | | **Effect** | **Certainty** |
| --- | --- | --- | --- | --- | --- | --- | --- | --- | --- |
|  | **Number of studies** | **Study design** | **Risk of bias** | **Inconsistency** | **Indirectness** | **Imprecision** | **Publication bias** | **Relative (95% CI)** |  |
| PE | 24 | RCTs | Not serious (Majority studies low/unclear RoB) | Serious  (I^2^ 67%) | Not serious | Not serious (large sample, 95%CI does not cross 1) | Serious (asymmetrical funnel plot) | RR 0.52 (0.41-0.65) | Low |
| SGA | 9 | RCTs | Not serious (Majority studies low/unclear RoB) | Not serious  (I^2^ 20%) | Not serious | Serious (large sample but 95%CI crosses 1) | N/A | RR 1.01 (0.83-1.23) | Moderate |
| LBW | 11 | RCTs | Not serious (Majority studies low/unclear RoB) | Not serious  (I^2^ 43%) | Not serious | Not serious (large sample, 95%CI does not cross 1) | Serious (asymmetrical funnel plot) | RR 0.84 (0.73- 0.96) | Moderate |
| PTB | 18 | RCTs | Not serious (Majority studies low/unclear RoB) | Serious  (I^2^ 95%) | Not serious | Not serious (large sample, 95%CI does not cross 1) | Serious (asymmetrical funnel plot) | RR 0.53 (0.33-0.86) | Low |
| Stillbirth | 7 | RCTs | Not serious (Majority studies low/unclear RoB) | Serious  (I^2^ 75%) | Not serious | Serious (large sample but 95%CI crosses 1) | N/A | RR 0.55 (0.24-1.23) | Low |
| Maternal mortality | 5 | RCTs | Not serious (Majority studies low/unclear RoB) | Not serious  (I^2^ 40%) | Not serious | Serious (large sample but 95%CI crosses 1) | N/A | RR 0.59 (0.18-1.92) | Moderate |

*PE – pre-eclampsia; SGA – small for gestational age; LBW – low birthweight; PTB – preterm birth; RCTs – randomized controlled trials; RoB – risk of bias*

# 6. Iodine compared to control

| **Outcome** | **Certainty assessment** | | | | | | | **Effect** | **Certainty** |
| --- | --- | --- | --- | --- | --- | --- | --- | --- | --- |
|  | **Number of studies** | **Study design** | **Risk of bias** | **Inconsistency** | **Indirectness** | **Imprecision** | **Publication bias** | **Relative (95% CI)** |  |
| PE | 0 | N/A | N/A | N/A | N/A | N/A | N/A | N/A | N/A |
| SGA | 2 | RCTs | Serious (None low/unclear RoB) | Not serious  (I^2^ 0%) | Not serious | Serious (sample <1,000, 95%CI crosses 1) | N/A | RR 1.26 (0.77-2.05) | Low |
| LBW | 2 | RCTs | Serious (None low/unclear RoB) | Not serious  (I^2^ 0%) | Not serious | Serious (sample <1,000, 95%CI crosses 1) | N/A | RR 0.56 (0.26-1.20) | Low |
| PTB | 2 | RCTs | Serious (None low/unclear RoB) | Not serious  (I^2^ 32%) | Not serious | Serious (sample <1,000, 95%CI crosses 1) | N/A | RR 0.71 (0.30-1.66) | Low |
| Stillbirth | 0 | N/A | N/A | N/A | N/A | N/A | N/A | N/A | N/A |
| Maternal mortality | 0 | N/A | N/A | N/A | N/A | N/A | N/A | N/A | N/A |

*PE – pre-eclampsia; SGA – small for gestational age; LBW – low birthweight; PTB – preterm birth; RCTs – randomized controlled trials; RoB – risk of bias*

# 7. Iron and/or folic acid compared to control

| **Outcome** | **Certainty assessment** | | | | | | | **Effect** | **Certainty** |
| --- | --- | --- | --- | --- | --- | --- | --- | --- | --- |
|  | **Number of studies** | **Study design** | **Risk of bias** | **Inconsistency** | **Indirectness** | **Imprecision** | **Publication bias** | **Relative (95% CI)** |  |
| PE | 6 | RCTs | Serious (Less than half low/unclear RoB) | Not serious  (I^2^ 0%) | Not serious | Serious (large sample but 95%CI crosses 1) | N/A | RR 0.99 (0.67-1.47) | Low |
| SGA | 7 | RCTs | Not serious (Majority studies low/unclear RoB) | Serious  (I^2^67%) | Not serious | Serious (large sample but 95%CI crosses 1) | N/A | RR 0.92 (0.80-1.06) | Low |
| LBW | 13 | RCTs | Serious (Less than half low/unclear RoB) | Not serious  (I^2^27%) | Not serious | Not serious (large sample, 95%CI does not cross 1) | Not serious (symmetrical funnel plot) | RR 0.87 (0.77-0.98) | Moderate |
| PTB | 15 | RCTs | Not serious (Majority studies low/unclear RoB) | Not serious  (I^2^ 0%) | Not serious | Serious (large sample but 95%CI crosses 1) | Not serious (symmetrical funnel plot) | RR 0.97 (0.89-1.06) | Moderate |
| Stillbirth | 7 | RCTs | Not serious (Majority studies low/unclear RoB) | Not serious  (I^2^ 0%) | Not serious | Serious (large sample but 95%CI crosses 1) | N/A | RR 0.88 (0.66-1.17) | Moderate |
| Maternal mortality | 0 | N/A | N/A | N/A | N/A | N/A | N/A | N/A | N/A |

*PE – pre-eclampsia; SGA – small for gestational age; LBW – low birthweight; PTB – preterm birth; RCTs – randomized controlled trials; RoB – risk of bias*

# 8. Magnesium compared to control

| **Outcome** | **Certainty assessment** | | | | | | | **Effect** | **Certainty** |
| --- | --- | --- | --- | --- | --- | --- | --- | --- | --- |
|  | **Number of studies** | **Study design** | **Risk of bias** | **Inconsistency** | **Indirectness** | **Imprecision** | **Publication bias** | **Relative (95% CI)** |  |
| PE | 3 | RCTs | Not serious (Majority studies low/unclear RoB) | Not serious  (I^2^ 0%) | Not serious | Serious (large sample but 95%CI crosses 1) | N/A | RR 0.87 (0.58-1.32) | Moderate |
| SGA | 3 | RCTs | Not serious (Majority studies low/unclear RoB) | Not serious  (I^2^ 7%) | Not serious | Serious (large sample but 95%CI crosses 1) | N/A | RR 0.76 (0.54-1.07) | Moderate |
| LBW | 5 | RCTs | Not serious (Majority studies low/unclear RoB) | Not serious  (I^2^ 22%) | Not serious | Serious (large sample but 95%CI crosses 1) | N/A | RR 0.95 (0.83-1.09) | Moderate |
| PTB | 7 | RCTs | Not serious (Majority studies low/unclear RoB) | Not serious  (I^2^ 37%) | Not serious | Serious (large sample but 95%CI crosses 1) | N/A | RR 0.89 (0.69-1.14) | Moderate |
| Stillbirth | 4 | RCTs | Serious (Less than half low/unclear RoB) | Not serious  (I^2^ 0%) | Not serious | Serious (large sample but 95%CI crosses 1) | N/A | RR 0.73 (0.43-1.25) | Low |
| Maternal mortality | 0 | N/A | N/A | N/A | N/A | N/A | N/A | N/A | N/A |

*PE – pre-eclampsia; SGA – small for gestational age; LBW – low birthweight; PTB – preterm birth; RCTs – randomized controlled trials; RoB – risk of bias*

# 9. Zinc compared to control

| **Outcome** | **Certainty assessment** | | | | | | | **Effect** | **Certainty** |
| --- | --- | --- | --- | --- | --- | --- | --- | --- | --- |
|  | **Number of studies** | **Study design** | **Risk of bias** | **Inconsistency** | **Indirectness** | **Imprecision** | **Publication bias** | **Relative (95% CI)** |  |
| PE | 5 | RCTs | Serious (Less than half low/unclear RoB) | Not serious  (I^2^ 39%) | Not serious | Serious (large sample but 95%CI crosses 1) | N/A | RR 1.31 (0.82-2.10) | Low |
| SGA | 8 | RCTs | Not serious (Majority studies low/unclear RoB) | Not serious  (I^2^ 28%) | Not serious | Serious (large sample but 95%CI crosses 1) | N/A | RR 1.03 (0.95-1.11) | Moderate |
| LBW | 12 | RCTs | Not serious (Majority studies low/unclear RoB) | Not serious  (I^2^ 26%) | Not serious | Serious (large sample but 95%CI crosses 1) | Serious (asymmetrical funnel plot) | RR 1.05 (0.94-1.17) | Low |
| PTB | 16 | RCTs | Not serious (Majority studies low/unclear RoB) | Not serious  (I^2^ 17%) | Not serious | Not serious (large sample, 95%CI does not cross 1) | Serious (asymmetrical funnel plot) | RR 0.86 (0.76-0.97) | Moderate |
| Stillbirth | 2 | RCTs | Serious (Less than half low/unclear RoB) | N/A (only one trial with estimable outcomes) | Not serious | Serious (sample <1,000, 95%CI crosses 1) | N/A | RR 0.20 (0.01-4.12) | Low |
| Maternal mortality | 1 | RCTs | Not serious (all studies low/unclear RoB) | N/A (only one trial | Not serious | Serious (sample <1,000, 95%CI crosses 1) | N/A | RR 0.31 (0.01-7.43) | Low |

*PE – pre-eclampsia; SGA – small for gestational age; LBW – low birthweight; PTB – preterm birth; RCTs – randomized controlled trials; RoB – risk of bias*

# 10. Garlic compared to control

| **Outcome** | **Certainty assessment** | | | | | | | **Effect** | **Certainty** |
| --- | --- | --- | --- | --- | --- | --- | --- | --- | --- |
|  | **Number of studies** | **Study design** | **Risk of bias** | **Inconsistency** | **Indirectness** | **Imprecision** | **Publication bias** | **Relative (95% CI)** |  |
| PE | 1 | RCTs | Serious (None low/unclear RoB) | N/A (only one trial | Not serious | Serious (sample <1,000, 95%CI crosses 1) | N/A | RR 0.78 (0.31-1.93) | Low |
| SGA | 0 | N/A | N/A | N/A | N/A | N/A | N/A | N/A | N/A |
| LBW | 0 | N/A | N/A | N/A | N/A | N/A | N/A | N/A | N/A |
| PTB | 0 | N/A | N/A | N/A | N/A | N/A | N/A | N/A | N/A |
| Stillbirth | 0 | N/A | N/A | N/A | N/A | N/A | N/A | N/A | N/A |
| Maternal mortality | 0 | N/A | N/A | N/A | N/A | N/A | N/A | N/A | N/A |

*PE – pre-eclampsia; SGA – small for gestational age; LBW – low birthweight; PTB – preterm birth; RCTs – randomized controlled trials; RoB – risk of bias*

# 11. Multiple micronutrients compared to control

| **Outcome** | **Certainty assessment** | | | | | | | **Effect** | **Certainty** |
| --- | --- | --- | --- | --- | --- | --- | --- | --- | --- |
|  | **Number of studies** | **Study design** | **Risk of bias** | **Inconsistency** | **Indirectness** | **Imprecision** | **Publication bias** | **Relative (95% CI)** |  |
| PE | 2 | RCTs | Serious (Less than half low/unclear RoB) | Not serious  (I^2^ 0%) | Not serious | Serious (sample <1,000, 95%CI does not cross 1) | N/A | RR 0.40 (0.27-0.59) | Low |
| SGA | 16 | RCTs | Not serious (Majority studies low/unclear RoB) | Serious  (I^2^59%) | Not serious | Not serious (large sample, 95%CI does not cross 1) | Not serious (symmetrical funnel plot) | RR 0.88 (0.82-0.95) | Moderate |
| LBW | 20 | RCTs | Not serious (Majority studies low/unclear RoB) | Not serious  (I^2^ 22%) | Not serious | Not serious (large sample, 95%CI does not cross 1) | Not serious (symmetrical funnel plot) | RR 0.87 (0.85-0.90) | High |
| PTB | 17 | RCTs | Not serious (Majority studies low/unclear RoB) | Serious  (I^2^74%) | Not serious | Serious (large sample but 95%CI crosses 1) | Not serious (symmetrical funnel plot) | RR 0.93 (0.85-1.02) | Low |
| Stillbirth | 20 | RCTs | Not serious (Majority studies low/unclear RoB) | Serious  (I^2^75%) | Not serious | Serious (large sample but 95%CI crosses 1) | Not serious (symmetrical funnel plot) | RR 1.09 (0.89-1.34) | Low |
| Maternal mortality | 8 | RCTs | Not serious (Majority studies low/unclear RoB) | Not serious  (I^2^ 0%) | Not serious | Serious (large sample but 95%CI crosses 1) | N/A | RR 1.17 (0.82-1.67) | Moderate |

*PE – pre-eclampsia; SGA – small for gestational age; LBW – low birthweight; PTB – preterm birth; RCTs – randomized controlled trials; RoB – risk of bias*

# 12. Lipid-based nutrients compared to control

| **Outcome** | **Certainty assessment** | | | | | | | **Effect** | **Certainty** |
| --- | --- | --- | --- | --- | --- | --- | --- | --- | --- |
|  | **Number of studies** | **Study design** | **Risk of bias** | **Inconsistency** | **Indirectness** | **Imprecision** | **Publication bias** | **Relative (95% CI)** |  |
| PE | 0 | N/A | N/A | N/A | N/A | N/A | N/A | N/A | N/A |
| SGA | 4 | RCTs | Not serious (Majority studies low/unclear RoB) | Not serious  (I^2^ 0%) | Not serious | Not serious (large sample, 95%CI does not cross 1) | N/A | RR 0.93 (0.88-0.98) | High |
| LBW | 5 | RCTs | Not serious (Majority studies low/unclear RoB) | Not serious  (I^2^ 0%) | Not serious | Not serious (large sample, 95%CI does not cross 1) | N/A | RR 0.89 (0.82-0.98) | High |
| PTB | 5 | RCTs | Not serious (Majority studies low/unclear RoB) | Not serious  (I^2^ 0%) | Not serious | Serious (large sample but 95%CI crosses 1) | N/A | RR 0.99 (0.86-1.14) | Moderate |
| Stillbirth | 4 | RCTs | Not serious (Majority studies low/unclear RoB) | Serious  (I^2^55%) | Not serious | Serious (large sample but 95%CI crosses 1) | N/A | RR 1.06 (0.60-1.87) | Low |
| Maternal mortality | 3 | RCTs | Not serious (Majority studies low/unclear RoB) | Not serious  (I^2^ 0%) | Not serious | Serious (large sample but 95%CI crosses 1) | N/A | RR 0.52 (0.12-2.28) | Moderate |

*PE – pre-eclampsia; SGA – small for gestational age; LBW – low birthweight; PTB – preterm birth; RCTs – randomized controlled trials; RoB – risk of bias*

# 13. Balanced protein/energy compared to control

| **Outcome** | **Certainty assessment** | | | | | | | **Effect** | **Certainty** |
| --- | --- | --- | --- | --- | --- | --- | --- | --- | --- |
|  | **Number of studies** | **Study design** | **Risk of bias** | **Inconsistency** | **Indirectness** | **Imprecision** | **Publication bias** | **Relative (95% CI)** |  |
| PE | 2 | RCTs | Serious (None low/unclear RoB) | N/A (only one trial with estimable outcomes) | Not serious | Serious (sample <1,000, 95%CI does not cross 1) | N/A | RR 1.48 (0.82-2.66) | Low |
| SGA | 7 | RCTs | Serious (Less than half low/unclear RoB) | Not serious  (I^2^ 16%) | Not serious | Not serious (large sample, 95%CI does not cross 1) | N/A | RR 0.79 (0.69-0.90) | Moderate |
| LBW | 0 | N/A | N/A | N/A | N/A | N/A | N/A | N/A | N/A |
| PTB | 5 | RCTs | Serious (Less than half low/unclear RoB) | Not serious  (I^2^ 0%) | Not serious | Serious (large sample but 95%CI crosses 1) | N/A | RR 0.96 (0.80-1.16) | Low |
| Stillbirth | 5 | RCTs | Serious (None low/unclear RoB) | Not serious  (I^2^10%) | Not serious | Not serious (large sample, 95%CI does not cross 1) | N/A | RR 0.60 (0.39-0.94) | Moderate |
| Maternal mortality | 0 | N/A | N/A | N/A | N/A | N/A | N/A | N/A | N/A |

*PE – pre-eclampsia; SGA – small for gestational age; LBW – low birthweight; PTB – preterm birth; RCTs – randomized controlled trials; RoB – risk of bias*

# 14. High protein compared to control

| **Outcome** | **Certainty assessment** | | | | | | | **Effect** | **Certainty** |
| --- | --- | --- | --- | --- | --- | --- | --- | --- | --- |
|  | **Number of studies** | **Study design** | **Risk of bias** | **Inconsistency** | **Indirectness** | **Imprecision** | **Publication bias** | **Relative (95% CI)** |  |
| PE | 0 | N/A | N/A | N/A | N/A | N/A | N/A | N/A | N/A |
| SGA | 1 | RCTs | Serious (None low/unclear RoB) | N/A (only one trial | Not serious | Serious (sample <1,000, 95%CI does not cross 1) | N/A | RR 1.58 (1.03-2.41) | Low |
| LBW | 0 | N/A | N/A | N/A | N/A | N/A | N/A | N/A | N/A |
| PTB | 0 | N/A | N/A | N/A | N/A | N/A | N/A | N/A | N/A |
| Stillbirth | 1 | RCTs | Serious (None low/unclear RoB) | N/A (only one trial | Not serious | Serious (sample <1,000, 95%CI crosses 1) | N/A | RR 0.81 (0.31-2.15,) | Low |
| Maternal mortality | 0 | N/A | N/A | N/A | N/A | N/A | N/A | N/A | N/A |

*PE – pre-eclampsia; SGA – small for gestational age; LBW – low birthweight; PTB – preterm birth; RCTs – randomized controlled trials; RoB – risk of bias*

# 15. Calf blood extract compared to control

| **Outcome** | **Certainty assessment** | | | | | | | **Effect** | **Certainty** |
| --- | --- | --- | --- | --- | --- | --- | --- | --- | --- |
|  | **Number of studies** | **Study design** | **Risk of bias** | **Inconsistency** | **Indirectness** | **Imprecision** | **Publication bias** | **Relative (95% CI)** |  |
| PE | 0 | N/A | N/A | N/A | N/A | N/A | N/A | N/A | N/A |
| SGA | 1 | RCTs | Serious (None low/unclear RoB) | N/A (only one trial | Not serious | Very serious (sample <50, 95%CI crosses 1) | N/A | RR 0.54 (0.20-1.47) | Very low |
| LBW | 0 | N/A | N/A | N/A | N/A | N/A | N/A | N/A | N/A |
| PTB | 0 | N/A | N/A | N/A | N/A | N/A | N/A | N/A | N/A |
| Stillbirth | 0 | N/A | N/A | N/A | N/A | N/A | N/A | N/A | N/A |
| Maternal mortality | 0 | N/A | N/A | N/A | N/A | N/A | N/A | N/A | N/A |

*PE – pre-eclampsia; SGA – small for gestational age; LBW – low birthweight; PTB – preterm birth; RCTs – randomized controlled trials; RoB – risk of bias*

# 16. Glucose compared to control

| **Outcome** | **Certainty assessment** | | | | | | | **Effect** | **Certainty** |
| --- | --- | --- | --- | --- | --- | --- | --- | --- | --- |
|  | **Number of studies** | **Study design** | **Risk of bias** | **Inconsistency** | **Indirectness** | **Imprecision** | **Publication bias** | **Relative (95% CI)** |  |
| PE | 0 | N/A | N/A | N/A | N/A | N/A | N/A | N/A | N/A |
| SGA | 1 | RCTs | Serious (None low/unclear RoB) | N/A (only one trial | Not serious | Very serious (sample <50, 95%CI crosses 1) | N/A | RR 1.11 (0.64-1.92) | Very low |
| LBW | 0 | N/A | N/A | N/A | N/A | N/A | N/A | N/A | N/A |
| PTB | 0 | N/A | N/A | N/A | N/A | N/A | N/A | N/A | N/A |
| Stillbirth | 0 | N/A | N/A | N/A | N/A | N/A | N/A | N/A | N/A |
| Maternal mortality | 0 | N/A | N/A | N/A | N/A | N/A | N/A | N/A | N/A |

*PE – pre-eclampsia; SGA – small for gestational age; LBW – low birthweight; PTB – preterm birth; RCTs – randomized controlled trials; RoB – risk of bias*

# 17. Galactose compared to control

| **Outcome** | **Certainty assessment** | | | | | | | **Effect** | **Certainty** |
| --- | --- | --- | --- | --- | --- | --- | --- | --- | --- |
|  | **Number of studies** | **Study design** | **Risk of bias** | **Inconsistency** | **Indirectness** | **Imprecision** | **Publication bias** | **Relative (95% CI)** |  |
| PE | 0 | N/A | N/A | N/A | N/A | N/A | N/A | N/A | N/A |
| SGA | 1 | RCTs | Serious (None low/unclear RoB) | N/A (only one trial | Not serious | Very serious (sample <50, 95%CI crosses 1) | N/A | RR 0.78 (0.39-1.54) | Very low |
| LBW | 0 | N/A | N/A | N/A | N/A | N/A | N/A | N/A | N/A |
| PTB | 0 | N/A | N/A | N/A | N/A | N/A | N/A | N/A | N/A |
| Stillbirth | 0 | N/A | N/A | N/A | N/A | N/A | N/A | N/A | N/A |
| Maternal mortality | 0 | N/A | N/A | N/A | N/A | N/A | N/A | N/A | N/A |

*PE – pre-eclampsia; SGA – small for gestational age; LBW – low birthweight; PTB – preterm birth; RCTs – randomized controlled trials; RoB – risk of bias*

# 18. Polyunsaturated omega-3 fatty acid compared to control

| **Outcome** | **Certainty assessment** | | | | | | | **Effect** | **Certainty** |
| --- | --- | --- | --- | --- | --- | --- | --- | --- | --- |
|  | **Number of studies** | **Study design** | **Risk of bias** | **Inconsistency** | **Indirectness** | **Imprecision** | **Publication bias** | **Relative (95% CI)** |  |
| PE | 18 | RCTs | Not serious (Majority studies low/unclear RoB) | Not serious  (I^2^ 9%) | Not serious | Serious (large sample but 95%CI crosses 1) | Not serious (symmetrical funnel plot) | RR 0.87 (0.71-1.07) | Moderate |
| SGA | 8 | RCTs | Not serious (Majority studies low/unclear RoB) | Not serious  (I^2^ 0%) | Not serious | Serious (large sample but 95%CI crosses 1) | N/A | RR 1.01 (0.90-1.13) | Moderate |
| LBW | 13 | RCTs | Not serious (Majority studies low/unclear RoB) | Not serious  (I^2^32%) | Not serious | Not serious (large sample, 95%CI does not cross 1) | Not serious (symmetrical funnel plot) | RR 0.87 (0.78- 0.96) | High |
| PTB | 28 | RCTs | Not serious (Majority studies low/unclear RoB) | Not serious  (I^2^13%) | Not serious | Not serious (large sample, 95%CI does not cross 1) | Not serious (symmetrical funnel plot) | RR 0.85 (0.76-0.95) | High |
| Stillbirth | 13 | RCTs | Serious (Less than half low/unclear RoB) | Not serious  (I^2^ 32%) | Not serious | Serious (large sample but 95%CI crosses 1) | Not serious (symmetrical funnel plot) | RR 0.89 (0.58-1.37) | Low |
| Maternal mortality | 0 | N/A | N/A | N/A | N/A | N/A | N/A | N/A | N/A |

*PE – pre-eclampsia; SGA – small for gestational age; LBW – low birthweight; PTB – preterm birth; RCTs – randomized controlled trials; RoB – risk of bias*

# 19. Salt restriction compared to control

| **Outcome** | **Certainty assessment** | | | | | | | **Effect** | **Certainty** |
| --- | --- | --- | --- | --- | --- | --- | --- | --- | --- |
|  | **Number of studies** | **Study design** | **Risk of bias** | **Inconsistency** | **Indirectness** | **Imprecision** | **Publication bias** | **Relative (95% CI)** |  |
| PE | 2 | RCTs | Serious (None low/unclear RoB) | Not serious  (I^2^ 0%) | Not serious | Serious (sample <1,000, 95%CI crosses 1) | N/A | RR 1.11 (0.46-2.66) | Low |
| SGA | 1 | RCTs | Serious (None low/unclear RoB) | N/A (only one trial | Not serious | Serious (sample <1,000, 95%CI crosses 1) | N/A | RR 1.50 (0.73-3.07) | Low |
| LBW | 0 | N/A | N/A | N/A | N/A | N/A | N/A | N/A | N/A |
| PTB | 1 | RCTs | Serious (None low/unclear RoB) | N/A (only one trial | Not serious | Serious (sample <1,000, 95%CI crosses 1) | N/A | RR 1.08 (0.46-2.56) | Low |
| Stillbirth | 0 | N/A | N/A | N/A | N/A | N/A | N/A | N/A | N/A |
| Maternal mortality | 0 | N/A | N/A | N/A | N/A | N/A | N/A | N/A | N/A |

*PE – pre-eclampsia; SGA – small for gestational age; LBW – low birthweight; PTB – preterm birth; RCTs – randomized controlled trials; RoB – risk of bias*

# 20. Caffeine restriction compared to control

| **Outcome** | **Certainty assessment** | | | | | | | **Effect** | **Certainty** |
| --- | --- | --- | --- | --- | --- | --- | --- | --- | --- |
|  | **Number of studies** | **Study design** | **Risk of bias** | **Inconsistency** | **Indirectness** | **Imprecision** | **Publication bias** | **Relative (95% CI)** |  |
| PE | 0 | N/A | N/A | N/A | N/A | N/A | N/A | N/A | N/A |
| SGA | 1 | RCTs | Not serious (One study low/unclear RoB) | N/A (only one trial | Not serious | Serious (large sample but 95%CI crosses 1) | N/A | RR 0.97 (0.57-1.64) | Low |
| LBW | 0 | N/A | N/A | N/A | N/A | N/A | N/A | N/A | N/A |
| PTB | 1 | RCTs | Not serious (One study low/unclear RoB) | N/A (only one trial | Not serious | Serious (large sample but 95%CI crosses 1) | N/A | RR 0.81 (0.48-1.37) | Low |
| Stillbirth | 0 | N/A | N/A | N/A | N/A | N/A | N/A | N/A | N/A |
| Maternal mortality | 0 | N/A | N/A | N/A | N/A | N/A | N/A | N/A | N/A |

*PE – pre-eclampsia; SGA – small for gestational age; LBW – low birthweight; PTB – preterm birth; RCTs – randomized controlled trials; RoB – risk of bias*

# 21. Antenatal dietary counselling compared to control

| **Outcome** | **Certainty assessment** | | | | | | | **Effect** | **Certainty** |
| --- | --- | --- | --- | --- | --- | --- | --- | --- | --- |
|  | **Number of studies** | **Study design** | **Risk of bias** | **Inconsistency** | **Indirectness** | **Imprecision** | **Publication bias** | **Relative (95% CI)** |  |
| PE | 15 | RCTs | Not serious (Majority studies low/unclear RoB) | Not serious  (I^2^ 14%) | Not serious | Serious (large sample but 95%CI crosses 1) | Not serious (symmetrical funnel plot) | RR 0.97 (0.82-1.13) | Moderate |
| SGA | 10 | RCTs | Not serious (Majority studies low/unclear RoB) | Not serious  (I^2^ 0%) | Not serious | Serious (large sample but 95%CI crosses 1) | Not serious (symmetrical funnel plot) | RR 1.15 (0.94-1.41) | Moderate |
| LBW | 4 | RCTs | Not serious (Majority studies low/unclear RoB) | Serious  (I^2^ 84%) | Not serious | Serious (large sample but 95%CI crosses 1) | N/A | RR 0.54 (0.17-1.71) | Low |
| PTB | 14 | RCTs | Not serious (Majority studies low/unclear RoB) | Not serious  (I^2^ 21%) | Not serious | Not serious (large sample, 95%CI does not cross 1) | Not serious (symmetrical funnel plot) | RR 0.72 (0.61-0.86) | High |
| Stillbirth | 6 | RCTs | Not serious (Majority studies low/unclear RoB) | Not serious  (I^2^ 0%) | Not serious | Serious (large sample but 95%CI crosses 1) | N/A | RR 0.63 (0.28-1.40) | Moderate |
| Maternal mortality | 1 | RCTs | Not serious (all studies low/unclear RoB) | N/A (only one trial | Not serious | Serious (large sample but 95%CI crosses 1) | N/A | RR 1.08 (0.07-17.32) | Low |

*PE – pre-eclampsia; SGA – small for gestational age; LBW – low birthweight; PTB – preterm birth; RCTs – randomized controlled trials; RoB – risk of bias*
