# Supplementary material for: Effects of Maternal Nutritional Supplements and Dietary Interventions on Placental Complications: An Umbrella Review, Meta-Analysis and Evidence Map
Source: Nutrients. 2021 Jan 30;13(2):472. doi: 10.3390/nu13020472 (PMC7912620; doi:10.3390/nu13020472)
Supplement: Supplementary file 1 [file nutrients-13-00472-s001.zip › Supplementary files/Table S2 - Search terms.docx]

**Table S2: Search terms**

**Search strategy for Medline Ovid**

| 1. Nutrit* supplement or vitamin* or mineral* or micronutrit* |
| --- |
| 1. (Minerals/ or Dietary Supplements/ or Micronutrients/ or Vitamins/ or exp Diet/) and Humans/ |
| 1. (Dietary Proteins/ or Potassium, Dietary/ or Dietary Exposure/ or Dietary Sucrose/ or Dietary Supplements/ or Dietary Carbohydrates/ or Sodium, Dietary/ or Dietary Sugars/ or Dietary Fats/ or Cholesterol, Dietary/ or Calcium, Dietary/ or Plant Proteins, Dietary/ or Sodium Chloride, Dietary/ or Dietary Fiber/ or Fatty Acids/) and Humans/ |
| 1. Diet* or diet* qualit* or health* diet* or diet* intervention* or Mediterranean diet* or Mediterranean style diet* or Mediterranean type diet* or Dietary approaches to stop hypertension or DASH diet* |
| 1. Diet, Fat-restricted/ or Diet, High-protein/ or diet, Mediterranean/ or Diet, Reducing/ or Diet, Sodium-restricted/ or Diet, Vegetarian/ or Dietary Approaches to Stop Hypertension/ or Healthy diet/ |
| 1. Diet* pattern* or Eat* pattern* or Diet* habit or Diet* guideline* or Diet* adhere* or Diet* complian* or Nutrition* adhere* or Nutrition* complian* or Diet* score* or Diet* index* or Diet* indices |
| 1. 1 or 2 or 3 or 4 or 5 or 6 |
| 1. Pregnan* or Pregnan* complicat* or Placenta* pathology or Birth outcome* or Obstetric* outcome* |
| 1. Pregnancy/ or Pregnancy Complications/ or Pregnancy Outcome/ or Maternal Nutritional Physiological Phenomena/ or Prenatal Nutritional Physiological Phenomena/ |
| 1. Matern*mortality or Matern* death* or pregnancy death* or motherhood death* or women death* |
| 1. Maternal Mortality/ or Maternal Death/ |
| 1. Pregnan* hypertension or gestational hypertension or chronic hypertension or pre-eclampsia or preeclampsia or toxemia or eclampsia or HEELP syndrome |
| 1. Hypertension/ or Hypertension, Pregnancy-Induced/ or Pregnancy Complications, Cardiovascular/ or Pre-Eclampsia/ or HELLP Syndrome/ |
| 1. Preterm deliver* or Prematur* deliver* or Preterm birth* or Gestation* duration or Gestation* length or Gestation* age or Gestation* time |
| 1. Small for gestational age or SGA or Growth restriction or Growth retardation or Intrauterine growth or IUGR or Fetal growth or Birth size or Birth weight or Birthweight or Weight at birth or Newborn weight or Neonat* weight |
| 1. Stillbirth* or Still-birth* or Stillborn* or Still-born* or Foetal death* or Fetal death* or Fetus death* or Foetus death* or death of fetus* or Death of foetus or intrauterine death* or antepartum death* or utero death* or perinatal death* or prenatal death* or perinatal mortality or antenatal death* |
| 1. Premature birth/ or Gestational Age/ or Stillbirth/ or Fetal Death/ |
| 1. 8 or 9 or 10 or 11 or 12 or 13 or 14 or 15 or 16 or 17 |
| 1. review.pt. |
| 1. (medline or medlars or embase or pubmed or cochrane).tw,sh. |
| 1. (scisearch or psychinfo or psycinfo).tw,sh. |
| 1. (psychlit or psyclit).tw,sh. |
| 1. cinahl.tw,sh. |
| 1. ((hand adj2 search$) or (manual$ adj2 search$)).tw,sh. |
| 1. (electronic database$ or bibliographic database$ or computeri?ed database$ or online database$).tw,sh. |
| 1. (pooling or pooled or mantel haenszel).tw,sh. |
| 1. (peto or dersimonian or der simonian or fixed effect).tw,sh. |
| 1. (retraction of publication or retracted publication).pt. |
| 1. or/20-28 |
| 1. 19 and 29 |
| 1. meta-analysis.pt. |
| 1. meta-analysis.sh. |
| 1. (meta-analys$ or meta analys$ or metaanalys$).tw,sh. |
| 1. (systematic$ adj5 review$).tw,sh. |
| 1. (systematic$ adj5 overview$).tw,sh. |
| 1. (quantitativ$ adj5 review$).tw,sh. |
| 1. (quantitativ$ adj5 overview$).tw,sh. |
| 1. (quantitativ$ adj5 synthesis$).tw,sh. |
| 1. (methodologic$ adj5 review$).tw,sh. |
| 1. (methodologic$ adj5 overview$).tw,sh. |
| 1. (integrative research review$ or research integration).tw. |
| 1. or/31-41 |
| 1. 30 or 42 |
| 1. 7 and 18 and 43 |
