# Supplementary material for: Effects of Maternal Nutritional Supplements and Dietary Interventions on Placental Complications: An Umbrella Review, Meta-Analysis and Evidence Map
Source: Nutrients. 2021 Jan 30;13(2):472. doi: 10.3390/nu13020472 (PMC7912620; doi:10.3390/nu13020472)
Supplement: Supplementary file 1 [file nutrients-13-00472-s001.zip › Supplementary files/Table S3 - Excluded studies.docx]

**Table S3: Excluded studies**

| **Reference** | **Title** | **Description of reason for exclusion** | **Divergence from current review PICOS** |
| --- | --- | --- | --- |
| Abe et al 2016 | Supplementation with multiple micronutrients for breastfeeding women for improving outcomes for the mother and baby | Not outcomes of interest | Outcomes |
| Achamrah and Ditisheim 2018 | Nutritional approach to preeclampsia prevention | Overview review – see Table S4 | Overview review |
| Achón et al 2018 | Effects of Milk and Dairy Product Consumption on Pregnancy and Lactation Outcomes: A Systematic Review. | Not RCTs | Design |
| Agarwal et al 2018 | Vitamin D and its impact on maternal-fetal outcomes in pregnancy: A critical review. | Narrative synthesis only | Did not report meta-analyses of RCTs |
| Amati et al 2019 | The Impact of Mediterranean Dietary Patterns During Pregnancy on Maternal and Offspring Health | Observational studies not separated from RCTs and outcomes lacking in clarity | Design |
| An et al 2015 | Calcium supplementation reducing the risk of hypertensive disorders of pregnancy and related problems: A meta-analysis of multicentre randomized controlled trials | Duplicate that was not caught until full-text review | N/A |
| Araini et al 2015 | Review-Vitamin D and the prevention of preeclampsia: A systematic review. | No RCTs | Design |
| Atallah et al 2010 | Calcium supplementation during pregnancy for preventing hypertensive disorders and related problems | Authorship list is in wrong order thus the duplicate. Authorship list as shown on Cochrane site is: G Justus Hofmeyr, Álvaro N Atallah, Lelia Duley | N/A |
| Bourassa et al 2019 | Review of the evidence regarding the use of antenatal multiple micronutrient supplementation in low- and middle-income countries | Overview review – see Table S4 | Overview review |
| Buppasiri et al 2011 | Calcium supplementation (other than for preventing or treating hypertension) for improving pregnancy and infant outcomes | This is an older version of a Cochrane review that has since been revised. Most recent Cochrane review on the topic included instead. | N/A |
| Christesen et al 2012 | The impact of vitamin D on pregnancy: a systematic review. | Outcomes of interest not reported in RCTs included | Outcomes |
| Christian and Tielsch 2012 | Evidence for multiple micronutrient effects based on randomized controlled trials and meta-analyses in developing countries | Commentary | Design |
| Cruz et al 2018 | Impact of Vitamin A Supplementation on Pregnant Women and on Women Who Have Just Given Birth: A Systematic Review. | Unclear reporting of results according the our review's outcomes | Outcomes |
| Curtis et al 2018 | Maternal vitamin D supplementation during pregnancy. | Observational studies | Design |
| da Silva Lopes et al 2017 | Effects of nutrition interventions during pregnancy on low birth weight: an overview of systematic reviews. | Overview review – see Table S4 | Overview review |
| Das et al 2013 | Systematic review of zinc fortification trials | Not outcomes of interest (for child growth) | Outcomes |
| Das et al 2013 | Micronutrient fortification of food and its impact on woman and child health: a systematic review. | Not outcomes of interest | Outcomes |
| De-Regil et al 2012 | Vitamin D supplementation for women during pregnancy. | This is an older version of a Cochrane review that has since been revised. Most recent Cochrane review on the topic included instead. | N/A |
| De-Regil et al 2016 | Vitamin D supplementation for women during pregnancy | This is an older version of a Cochrane review that has since been revised. Most recent Cochrane review on the topic included instead. | N/A |
| Dodd et al 2008 | Dietary and lifestyle interventions to limit weight gain during pregnancy for obese or overweight women: A systematic review | Narrative synthesis only | Did not report meta-analyses of RCTs |
| Dror and Allen 2012 | Interventions with Vitamins B6, B12 and C in Pregnancy | Meta-analysis did not report RCT separately | Did not report meta-analyses of RCTs |
| Dror and Allen 2012 | Interventions with Vitamins B6, B12 and C in Pregnancy | Meta-analysis did not report RCT separately | Did not report meta-analyses of RCTs |
| Eddib and Yeh 2009 | Prevention of preeclampsia: is it still a disappointment? | Overview review – see Table S4 | Overview review |
| Etwel and Koren 2015 | When positive studies of novel therapies are subsequently nullified: cumulative meta-analyses in preeclampsia | Re-analysis of two existing meta-analyses | Design |
| Grieger and Clifton 2014 | A review of the impact of dietary intakes in human pregnancy on infant birthweight. | Overview review – see Table S4 | Overview review |
| Gulmezoglu and Hofmeyr 2000 | Maternal nutrient supplementation for suspected impaired fetal growth. | This is an older version of a Cochrane review that has since been revised. Most recent Cochrane review on the topic included instead. | N/A |
| Gulmezoglu et al 1997 | Effectiveness of interventions to prevent or treat impaired fetal growth | Overview review – see Table S4 | Overview review |
| Haider and Bhutta 2006 | Multiple-micronutrient supplementation for women during pregnancy. | This is an older version of a Cochrane review that has since been revised. Most recent Cochrane review on the topic included instead. | N/A |
| Haider and Bhutta 2017 | Multiple-micronutrient supplementation for women during pregnancy. | This is an older version of a Cochrane review that has since been revised. Most recent Cochrane review on the topic included instead. | N/A |
| Harvey et al 2017 | Vitamin D supplementation in pregnancy: a systematic review | Did not measure outcomes of interest with study designs of interest (meta-analysis of intervention studies only for infant birth weight - WMD, not LBW <2500g, pre-eclampsia examined only in observational studies) | Outcomes |
| Hess et al 2009 | Effects of maternal zinc supplementation on pregnancy and lactation outcomes. | Narrative synthesis only | Did not report meta-analyses of RCTs |
| Hodgetts et al 2015 | Effectiveness of folic acid supplementation in pregnancy on reducing the risk of small-for-gestational age neonates: a population study, systematic review and meta-analysis. | Meta-analysis did not report RCT separately | Did not report meta-analyses of RCTs |
| Hofmeyr and Manyame 2017 | Calcium supplementation commencing before or early in pregnancy, or food fortification with calcium, for preventing hypertensive disorders of pregnancy | This is an older version of a Cochrane review that has since been revised. Most recent Cochrane review on the topic included instead. | N/A |
| Hofmeyr et al 2002 | Calcium supplementation during pregnancy for preventing hypertensive disorders and related problems | This is an older version of a Cochrane review that has since been revised. Most recent Cochrane review on the topic included instead. | N/A |
| Hofmeyr et al 2003 | Calcium supplementation to prevent pre-eclampsia - A systematic review | Duplicate that was not caught until full-text review | N/A |
| Hofmeyr et al 2003 | Calcium supplementation to prevent pre-eclampsia--a systematic review | Duplicate that was not caught until full-text review | N/A |
| Hofmeyr et al 2006 | Calcium supplementation during pregnancy for preventing hypertensive disorders and related problems. | This is an older version of a Cochrane review that has since been revised. Most recent Cochrane review on the topic included instead. | N/A |
| Hofmeyr et al 2007 | Low‐dose calcium supplementation for preventing pre‐eclampsia: a systematic review and commentary | Duplicate - same abstract as "Dietary calcium supplementation for prevention of pre-eclampsia and related problems: a systematic review and commentary" 2007 | N/A |
| Hofmeyr et al 2010 | Calcium supplementation during pregnancy for preventing hypertensive disorders and related problems. | This is an older version of a Cochrane review that has since been revised. Most recent Cochrane review on the topic included instead. | N/A |
| Hofmeyr et al 2014 | Calcium supplementation during pregnancy for preventing hypertensive disorders and related problems. | This is an older version of a Cochrane review that has since been revised. Most recent Cochrane review on the topic included instead. | N/A |
| Hofmeyr et al 2014 | Low‐dose calcium supplementation for preventing pre‐eclampsia: a systematic review and commentary | Duplicate that was not caught until full-text review | N/A |
| Hofmeyr et al 2014 | Calcium supplementation during pregnancy for preventing hypertensive disorders and related problems | Duplicate that was not caught until full-text review | N/A |
| Hosli et al 2007 | Role of omega 3-fatty acids and multivitamins in gestation | Overview review – see Table S4 | Overview review |
| Hovdenak and Haram 2012 | Influence of mineral and vitamin supplements on pregnancy outcome | Narrative synthesis only | Did not report meta-analyses of RCTs |
| Hovdenak and Haram 2012 | Influence of mineral and vitamin supplements on pregnancy outcome | Narrative synthesis only | Did not report meta-analyses of RCTs |
| Hovdenak and Haram 2012 | Influence of mineral and vitamin supplements on pregnancy outcome | Narrative synthesis only | Did not report meta-analyses of RCTs |
| Hovdenak and Haram 2012 | Influence of mineral and vitamin supplements on pregnancy outcome | Narrative synthesis only | Did not report meta-analyses of RCTs |
| Hovdenak and Haram 2012 | Influence of mineral and vitamin supplements on pregnancy outcome | Narrative synthesis only | Did not report meta-analyses of RCTs |
| Hovdenak and Haram 2012 | Influence of mineral and vitamin supplements on pregnancy outcome | Narrative synthesis only | Did not report meta-analyses of RCTs |
| Hovdenak and Haram 2012 | Influence of mineral and vitamin supplements on pregnancy outcome | Narrative synthesis only | Did not report meta-analyses of RCTs |
| Hovdenak and Haram 2012 | Influence of mineral and vitamin supplements on pregnancy outcome | Narrative synthesis only | Did not report meta-analyses of RCTs |
| Hovdenak and Haram 2012 | Influence of mineral and vitamin supplements on pregnancy outcome | Narrative synthesis only | Did not report meta-analyses of RCTs |
| Hovdenak and Haram 2012 | Influence of mineral and vitamin supplements on pregnancy outcome | Narrative synthesis only | Did not report meta-analyses of RCTs |
| Hypponen et al 2012 | Vitamin D and Pre-Eclampsia: A Systematic Review and Meta-Analysis | Duplicate that was not caught until full-text review | N/A |
| Imdad and Bhutta 2012 | Routine iron/folate supplementation during pregnancy: effect on maternal anaemia and birth outcomes. | Meta-analysis did not report RCT separately | Did not report meta-analyses of RCTs |
| Imdad and Bhutta 2012 | Maternal nutrition and birth outcomes: effect of balanced protein-energy supplementation. | Meta-analysis did not report RCT separately | Did not report meta-analyses of RCTs |
| Imdad et al 2011 | Effect of balanced protein energy supplementation during pregnancy on birth outcomes | RCTs not separated from quasi-interventions | Design |
| Imdad et al 2011 | The effect of folic acid, protein energy and multiple micronutrient supplements in pregnancy on stillbirths. | RCTs not separated from quasi-interventions | Design |
| Iqbal et al 2019 | Maternal and neonatal outcomes related to iron supplementation or iron status: a summary of meta-analyses. | Overview review – see Table S4 | Overview review |
| i-WIP 2017 | Effect of Diet and Physical Activity Based Interventions in Pregnancy on Gestational Weight Gain and Pregnancy Outcomes: Meta-analysis of Individual Participant Data From Randomized Trials | Duplicate that was not caught until full-text review | N/A |
| Karras et al 2015 | Maternal vitamin D status in pregnancy: a critical appraisal of current analytical data on maternal and neonatal outcomes | Observational studies | Design |
| Kiely et al 2017 | Vitamin D in pregnancy: current perspectives and future directions. | Overview review – see Table S4 | Overview review |
| Kramer 1993 | Effects of energy and protein intakes on pregnancy outcome: an overview of the research evidence from controlled clinical trials. | RCTs not separated from quasi-experimental studies | Design |
| Kramer 2000 | Balanced protein/energy supplementation in pregnancy. | This is an older version of a Cochrane review that has since been revised. Most recent Cochrane review on the topic included instead. | N/A |
| Kramer 2000 | High protein supplementation in pregnancy. | This is an older version of a Cochrane review that has since been revised. Most recent Cochrane review on the topic included instead. | N/A |
| Kramer and Kakuma 2003 | Energy and protein intake in pregnancy. | This is an older version of a Cochrane review that has since been revised. Most recent Cochrane review on the topic included instead. | N/A |
| Liberato et al 2013 | Effects of protein energy supplementation during pregnancy on fetal growth: a review of the literature focusing on contextual factors | Narrative synthesis only | Did not report meta-analyses of RCTs |
| Liu et al 2018 | Supplementation of folic acid in pregnancy and the risk of preeclampsia and gestational hypertension: a meta-analysis. | Cohort studies not separated from RCTs | Design |
| Long et al 2015 | Benefits of iron supplementation for low birth weight infants: a systematic review | Supplementation of children | Population |
| Mahomed 2000 | Iron supplementation in pregnancy. | This is an older version of a Cochrane review that has since been revised. Most recent Cochrane review on the topic included instead. | N/A |
| Mahomed 2006 | Zinc supplementation in pregnancy. | This is an older version of a Cochrane review that has since been revised. Most recent Cochrane review on the topic included instead. | N/A |
| Mahomed et al 2007 | Zinc supplementation for improving pregnancy and infant outcome | This is an older version of a Cochrane review that has since been revised. Most recent Cochrane review on the topic included instead. | N/A |
| Mahomed et al 2007 | Zinc supplementation for improving pregnancy and infant outcome. | Duplicate that was not caught until full-text review | N/A |
| Makrides and Crowther 2000 | Magnesium supplementation in pregnancy | This is an older version of a Cochrane review that has since been revised. Most recent Cochrane review on the topic included instead. | N/A |
| Makrides et al 2006 | Marine oil, and other prostaglandin precursor, supplementation for pregnancy uncomplicated by pre-eclampsia or intrauterine growth restriction. | This is an older version of a Cochrane review that has since been revised. Most recent Cochrane review on the topic included instead. | N/A |
| Makrides et al 2019 | Magnesium supplementation in pregnancy | Year should be 2014 | N/A |
| Margetts et al 2009 | Multiple micronutrient supplementation during pregnancy in low-income countries: review of methods and characteristics of studies included in the meta-analyses. | Describes characteristics of a meta-analysis but not the outcomes itself | Outcomes |
| Matei et al 2019 | Primary and secondary prevention of preterm birth: a review of systematic reviews and ongoing randomized controlled trials. | Overview review – see Table S4 | Overview review |
| Mateussi et al 2017 | What do Cochrane systematic reviews say about interventions for vitamin D supplementation?. | Overview review – see Table S4 | Overview review |
| Mathews 1996 | Antioxidant nutrients in pregnancy: a systematic review of the literature | Lack of clarity on methodology and whether included trials were RCTs | Design |
| McCauley et al 2016 | Vitamin A supplementation during pregnancy for maternal and newborn outcomes | Duplicate that was not caught until full-text review | N/A |
| Medley et al 2018 | Interventions during pregnancy to prevent preterm birth: an overview of Cochrane systematic reviews | Overview review | Design |
| Medley et al 2018 | Interventions during pregnancy to prevent preterm birth: an overview of Cochrane systematic reviews | Duplicate that was not caught until full-text review | N/A |
| Merialdi et al 2003 | Nutritional interventions during pregnancy for the prevention or treatment of impaired fetal growth: an overview of randomized controlled trials | Overview review – see Table S4 | Overview review |
| Mills et al 2005 | Vitamin supplementation for prevention of mother-to-child transmission of HIV and pre-term delivery: a systematic review of randomized trial including more than 2800 women. | Not outcomes of interest (MTCT and infant mortality at 1 yr) | Outcomes |
| Mohsenzadeh-Ledari et al 2019 | Appropriate Interventions for Pregnant Women with Indicators of Metabolic Syndrome on Pregnancy Outcomes: A Systematic Review. | Lack of clarity on outcomes and methodology | Outcomes |
| Mori et al 2012 | Zinc supplementation for improving pregnancy and infant outcome. | This is an older version of a Cochrane review that has since been revised. Most recent Cochrane review on the topic included instead. | N/A |
| Mori et al 2015 | Zinc supplementation for improving pregnancy and infant outcome. | Authorship list is in wrong order thus the duplicate. | N/A |
| Moutquin et al 1994 | Report of the Canadian Hypertension Society Consensus Conference: 2. Nonpharmacologic management and prevention of hypertensive disorders in pregnancy. | Overview review – see Table S4 | Overview review |
| Mwangi et al 2017 | Safety and benefits of antenatal oral iron supplementation in low-income countries: a review | Overview review – see Table S4 | Overview review |
| O'Brien et al 2015 | Systematic review of antenatal dietary and lifestyle interventions in women with normal body mass index | Duplicate that was not caught until full-text review | N/A |
| O'Brien et al 2016 | Systematic review of antenatal dietary and lifestyle interventions in women with a normal body mass index. | Dietary interventions not separated from lifestyle interventions | Intervention |
| Oliveira et al 2007 | [Evidence of the impact of vitamin A supplementation on maternal and child health]. | Duplicate that was not caught until full-text review | N/A |
| Oliveira-Menegozzo et al 2010 | Vitamin A supplementation for postpartum women. | Not outcomes of interest | Outcomes |
| Palacios et al 2019 | Regimens of vitamin D supplementation for women during pregnancy | Compared dosages of vitamin D, thus supplement was provided to both intervention and comparison groups | Comparison |
| Peaceman et al 2018 | Lifestyle Interventions Limit Gestational Weight Gain in Women with Overweight or Obesity: LIFE-Moms Prospective Meta-Analysis | Lifestyle interventions not specifically on nutrition | Intervention |
| Pena-Rosas and Viteri 2006 | Effects of routine oral iron supplementation with or without folic acid for women during pregnancy. | This is an older version of a Cochrane review that has since been revised. Most recent Cochrane review on the topic included instead. | N/A |
| Pena-Rosas et al 2009 | Effects and safety of preventive oral iron or iron+folic acid supplementation for women during pregnancy. | This is an older version of a Cochrane review that has since been revised. Most recent Cochrane review on the topic included instead. | N/A |
| Pena-Rosas et al 2012 | Intermittent oral iron supplementation during pregnancy | Comparing dosage regimes - supplement was given to both intervention and comparator | Comparison |
| Pena-Rosas et al 2012 | Daily oral iron supplementation during pregnancy. | This is an older version of a Cochrane review that has since been revised. Most recent Cochrane review on the topic included instead. | N/A |
| Pena-Rosas et al 2015 | Intermittent oral iron supplementation during pregnancy | Comparing dosage regimes - supplement was given to both intervention and comparator | Comparison |
| Pena-Rosas et al 2015 | Daily oral iron supplementation during pregnancy | Duplicate that was not caught until full-text review | N/A |
| Ramakrishnan et al 2012 | Effect of Women's Nutrition before and during Early Pregnancy on Maternal and Infant Outcomes: A Systematic Review | Narrative synthesis only | Did not report meta-analyses of RCTs |
| Ramakrishnan et al 2014 | Maternal nutrition interventions to improve maternal, newborn, and child health outcomes. | Lack of clarity around methodology | Design |
| Reijnders et al 2019 | The impact of periconceptional maternal lifestyle on clinical features and biomarkers of placental development and function: a systematic review. | Not RCTs | Design |
| Rogozinksa et al 2017 | Effect of diet and physical activity based interventions in pregnancy on gestational weight gain and pregnancy outcomes: meta-analysis of individual participant data from randomised trials (vol 358, j3119, 2017) | Diet and lifestyle interventions not separated | Intervention |
| Ross et al 2005 | Evaluating effectiveness of complex interventions aimed at reducing maternal mortality in developing countries. | Narrative synthesis only | Did not report meta-analyses of RCTs |
| Rotondi et al 2010 | Vitamin A supplementation and neonatal mortality in the developing world: a meta-regression of cluster-randomized trials. | Not outcomes of interest (infant mortality) | Outcomes |
| Rumbold and Crowther 2005 | Vitamin C supplementation in pregnancy | This is an older version of a Cochrane review that has since been revised. Most recent Cochrane review on the topic included instead. | N/A |
| Rumbold and Crowther 2005 | Vitamin E supplementation in pregnancy. | This is an older version of a Cochrane review that has since been revised. Most recent Cochrane review on the topic included instead. | N/A |
| Rumbold et al 2005 | Antioxidants for preventing pre-eclampsia | This is an older version of a Cochrane review that has since been revised. Most recent Cochrane review on the topic included instead. | N/A |
| Samuel et al 2019 | Preterm Birth: A Narrative Review of the Current Evidence on Nutritional and Bioactive Solutions for Risk Reduction. | Narrative synthesis only | Did not report meta-analyses of RCTs |
| Samuel et al 2019 | Preterm Birth: A Narrative Review of the Current Evidence on Nutritional and Bioactive Solutions for Risk Reduction. | Narrative synthesis only | Did not report meta-analyses of RCTs |
| Samuel et al 2019 | Preterm Birth: A Narrative Review of the Current Evidence on Nutritional and Bioactive Solutions for Risk Reduction. | Narrative synthesis only | Did not report meta-analyses of RCTs |
| Samuel et al 2019 | Preterm Birth: A Narrative Review of the Current Evidence on Nutritional and Bioactive Solutions for Risk Reduction. | Narrative synthesis only | Did not report meta-analyses of RCTs |
| Samuel et al 2019 | Preterm Birth: A Narrative Review of the Current Evidence on Nutritional and Bioactive Solutions for Risk Reduction. | Narrative synthesis only | Did not report meta-analyses of RCTs |
| Samuel et al 2019 | Preterm Birth: A Narrative Review of the Current Evidence on Nutritional and Bioactive Solutions for Risk Reduction. | Narrative synthesis only | Did not report meta-analyses of RCTs |
| Samuel et al 2019 | Preterm Birth: A Narrative Review of the Current Evidence on Nutritional and Bioactive Solutions for Risk Reduction. | Narrative synthesis only | Did not report meta-analyses of RCTs |
| Secher 2007 | Does fish oil prevent preterm birth?. | Overview review – see Table S4 | Overview review |
| Shimpton et al 2009 | Multiple micronutrient supplementation during pregnancy in developing-country settings: policy and program implications of the results of a meta-analysis. | Not outcomes of interest in this publication. Part of a series and outcomes of interest are reported in other articles. | Outcomes |
| Soltani et al 2015 | A systematic review of the effects of dietary interventions on neonatal outcomes in adolescent pregnancy. | Narrative synthesis only | Did not report meta-analyses of RCTs |
| Soltani et al 2015 | A systematic review of the effects of dietary interventions on neonatal outcomes in adolescent pregnancy. | Narrative synthesis only | Did not report meta-analyses of RCTs |
| Suchdev et al 2011 | Multiple micronutrient powders for home (point of use) fortification of foods in pregnant women: a systematic review. | Intended meta-analysis but no studies found | Did not report meta-analyses of RCTs |
| Swaney et al 2014 | Vitamin C Supplementation in Pregnancy--Does It Decrease Rates of Preterm Birth? A Systematic Review. | Narrative synthesis only | Did not report meta-analyses of RCTs |
| Thaver et al 2006 | Pyridoxine (vitamin B6) supplementation in pregnancy. | This is an older version of a Cochrane review that has since been revised. Most recent Cochrane review on the topic included instead. | N/A |
| Theodoratou et al 2014 | Vitamin D and multiple health outcomes: umbrella review of systematic reviews and meta-analyses of observational studies and randomised trials. | Overview review – see Table S4 | Overview review |
| van den Broek et al 2010 | Vitamin A supplementation during pregnancy for maternal and newborn outcomes. | This is an older version of a Cochrane review that has since been revised. Most recent Cochrane review on the topic included instead. | N/A |
| Van et al 2002 | Vitamin A supplementation during pregnancy. | This is an older version of a Cochrane review that has since been revised. Most recent Cochrane review on the topic included instead. | N/A |
| Van et al 2002 | Vitamin A supplementation during pregnancy | Duplicate that was not caught until full-text review | N/A |
| Villar et al 1998 | Nutritional and antimicrobial interventions to prevent preterm birth: an overview of randomized controlled trials | Overview review – see Table S4 | Overview review |
| Villar et al 2003 | Nutritional interventions during pregnancy for the prevention or treatment of maternal morbidity and preterm delivery: an overview of randomized controlled trials | Overview review – see Table S4 | Overview review |
| Wachtel 1989 | New perspectives in the nutrition of premature and low birth weight infants. | Commentary | Design |
| Wei 2013 | Vitamin D and pregnancy outcomes | Meta-analysis of observational studies | Design |
| Wilson et al 1989 | Association between Maternal Zinc Status, Dietary Zinc Intake and Pregnancy Complications: A Systematic Review. | Not RCTs | Design |
| Wolf et al 2017 | Multivitamin use and adverse birth outcomes in high-income countries: a systematic review and meta-analysis. | Did not do meta-analysis for the RCTs | Did not report meta-analyses of RCTs |
| Yakoob et al 2009 | Reducing stillbirths: behavioural and nutritional interventions before and during pregnancy | Observational studies | Design |
| Yang et al 2016 | Periconceptional folic acid fortification for the risk of gestational hypertension and pre-eclampsia: a meta-analysis of prospective studies. | Not RCTs | Design |
| Zerfu et al 2013 | Micronutrients and pregnancy; effect of supplementation on pregnancy and pregnancy outcomes: a systematic revie | Narrative synthesis only | Did not report meta-analyses of RCTs |
| Zerfu et al 2013 | Micronutrients and pregnancy; effect of supplementation on pregnancy and pregnancy outcomes: a systematic review | Narrative synthesis only | Did not report meta-analyses of RCTs |
| Zerfu et al 2013 | Micronutrients and pregnancy; effect of supplementation on pregnancy and pregnancy outcomes: a systematic review | Narrative synthesis only | Did not report meta-analyses of RCTs |
| Zhou et al 2017 | Vitamin D and risk of preterm birth: Up-to-date meta-analysis of randomized controlled trials and observational studies | Duplicate that was not caught until full-text review | N/A |
