# Supplementary material for: Effects of Maternal Nutritional Supplements and Dietary Interventions on Placental Complications: An Umbrella Review, Meta-Analysis and Evidence Map
Source: Nutrients. 2021 Jan 30;13(2):472. doi: 10.3390/nu13020472 (PMC7912620; doi:10.3390/nu13020472)
Supplement: Supplementary file 1 [file nutrients-13-00472-s001.zip › Supplementary files/Table S4 - Summary of overview reviews.docx]

**Table S4: Summary of overview reviews found in searches**

| **Author year** | **Type of review** | **Methodology** | **Focus of review** | **Main findings** | **Comments** |
| --- | --- | --- | --- | --- | --- |
| Achamrah and Ditisheim 2018 | Narrative (reviews of observational studies and reviews of RCTs, focus on Cochrane reviews), search methodology unclear | Unclear | Pre-eclampsia | - Calcium intake (>1 g/day) may reduce the risk of preeclampsia in women with low-calcium diet. - Routine supplementation of vitamins C, E or D for either the prevention or treatment of preeclampsia not supported - Zinc or folic acid supplementation not supported | Of 8 included reviews in Achamrah and Ditisheim 2018, four Cochrane reviews updated in the current review (Haider et al 2017 🡪 Keats et al 2019; Hofymeyr et al 2017 🡪 Hofymeyr et al 2019; De-Regil et al 2016 🡪 Palacios et al 2019; Hofmeyr et al 2014 🡪 Hofmeyr et al 2018) |
| Bourassa et al 2019 | Narrative (review of RCTs) | Unclear | Multiple micronutrient supplementation | - Multiple micronutrient supplementation has been shown to reduce rates of preterm birth, low birth weight, and small for gestational age in comparison with iron-folic acid on its own, particularly for malnourished women (anaemic, underweight) and female infants, without increased risk of harm | Keats 2019 and Smith 2017 both already included in current review |
| da Silva Lopes et al 2017 | Narrative (reviews of RCTs) | Systematic | Low birth weight | - Vitamin A, low-dose calcium, zinc, multiple micronutrients, nutritional education and provision of preventive anti-malarials associated with a decreased risk of LBW. - MMN and balanced protein/energy supplementation had a positive effect on SGA - High protein supplementation increased the risk of SGA. - High-dose calcium, zinc or long-chain n-3 fatty acid supplementation and nutritional education decreased the risk of PTB | One included publication did not meet eligibility for current review (Salamet et al 2015 as antihelminthics is beyond the scope of the current review on nutritional supplements and interventions) |
| Eddib and Yeh 2009 | Narrative (reviews of observational studies and reviews of RCTs) | Systematic but limited information reported | Pre-eclampsia | - Calcium supplementation reduces risk of pre-eclampsia among both high and low risk women - Vitamins C and E, zinc, fish oil, and magnesium supplementation shown not effective in preventing pre-eclampsia - Nitric oxide, folic acid, and antithrombotics inconclusive | Five included Cochrane reviews have since been updated (Hofmeyr 2002 🡪 Hofmeyr 2018; Rumbold 2002 🡪 2008; Rumbold 2007 🡪 Rumbold 2015; Mahomed 2007 🡪 Ota 2015; Makrides 2007 🡪 Makrides 2014) |
| Grieger and Clifton 2014 | Narrative (reviews of observational studies and reviews of RCTs) | Systematic but limited information reported | Low birth weight | - Insufficient evidence for omega-3 fatty acid, zinc, calcium, and/or vitamin D supplementation to reduce risk of LBW - Iron and folic acid supplementation appears to increased infant birthweight, though folic acid supplements does not reduce risk for LBW | Two included Cochrane reviews have since been updated (Mori 2012 🡪 Ota 2015; De-Regil 2012 🡪 Palacio 2019) |
| Gulmezoglu et al 1997 | Narrative (review of RCTs, Cochrane reviews) | Systematic | Fetal growth restriction | - Balanced protein/energy supplementation likely beneficial to prevent or manage impaired fetal growth - Zinc, folate, and magnesium supplementation during gestation merits further research | Lack of clarity on which systematic review contributed to which sub-group and outcome |
| Hosli et al 2007 | Narrative (Cochrane reviews, RCTs and observational studies) | Unclear | Omega-3 fatty acids, multivitamins | - Deficiencies of omega 3 fatty acids may be a contributing factor for pregnancy complications though research is inconsistent. | Two included Cochrane reviews has since been updated (Makrides et al 2006 🡪 Middleton et al 2018; Pena-Rosas 2006 🡪 Pena-Rosas 2015)  Review by Szajewska et al 2006 already included |
| Iqbal and Ekmekcioglu 2019 | Narrative (reviews of observational studies and reviews of RCTs) | Systematic | Maternal and neonatal outcomes with iron supplementation and status | - Maternal multi-micronutrient supplements had significant risk reducing effects on low birth weight and small for gestational age compared to those only on iron/folic acid supplements | Two included studies did not meet eligibility for current review (Reviez 2011 because the review examined delivery of iron (oral or intravenous) rather than if iron was effective as a nutritional supplement). |
| Kiely et al 2017 | Narrative (reviews of observational studies and reviews of RCTs) | Unclear | Vitamin D and pregnancy outcomes | - Limited evidence for a role of vitamin D supplementation in preventing PE, low birth weight and preterm birth - There are insufficient trial data to justify setting pregnancy-specific recommendations for vitamin D | Included Cochrane review has since been updated (De-Regil 2016 🡪 Palacio 2019), Thorne-Lyman and Fawzi 2012 already included in current review, other reviews consisted of observational studies |
| Khiang et al 2017 | Meta-analysis and network analysis (reviews of RCTs and individual RCTs) | Systematic | Calcium and hypertensive diseases of pregnancy | - Effects of calcium supplement on preeclampsia were robust and consistent for both direct and indirect meta-analyses - Supplementation of vitamin D with/without calcium ranked higher than calcium supplementation alone, though this needs to be confirmed direct comparison trials | 29 systematic reviews were found. However, reviews were not reported but included RCTs were screened and included in the meta-analysis. Consequently, this review will be treated as a meta-analysis of individual studies. |
| Kulier et al 1998 | Meta-analysis (overview of systematic reviews and individual RCTs | Systematic | Maternal morbidities | - Routine calcium supplementation seems to be promising intervention for pregnancy hypertension/pre-eclampsia (RR 0.22. 95% CI, 0.11-0.43 in high risk women, RR 0.32; CI, 0.21-0.49 in women with low dietary intake) - Iron/folate supplementation in antenatal care recommended in populations of high nutritional anaemia but unclear if adding vitamin A increases benefit | Did not report the reviews found so will be treated as a meta-analysis of individual studies |
| Matei et al 2019 | Narrative (review or IPD of RCTs) | Systematic | Preterm birth | - Cerclage, progesterone, low dose aspirin, and lifestyle and behavioural changes were most promising interventions to prevent preterm birth - Positive effects of calcium and zinc supplementation and nutritional education in preterm birth prevention - Lack of consistent evidence for iron, folic acid, vitamin A, vitamin D, vitamin E pyridoxine, fish oil, marine oil, and omega-3 fatty acids or zinc supplementation to support protective effect on preterm birth | One included Cochrane review has since been updated (De-Regil 2012 updated 🡪 Palacio 2019)  Two included studies did not meet eligibility for current review (Othman 2007 because review examined efficacy of probiotics, which is beyond the scope of the current review; Al Dorazi 2014 because full text could not be located as link was broken on the journal)  One review considered but was ultimately not included in the current review (Suchdev 2014). Though it planned to complete meta-analyses, no results were found |
| Mateussi et al 2017 | Narrative (Cochrane reviews) | Systematic | Vitamin D supplementation, overall health outcomes | - Vitamin D supplementation reduces risk of preterm birth and low birth weight but no statistical difference in risk of pre-eclampsia or gestational diabetes | One Cochrane review included on pregnancy outcomes, of which an updated version is included in the current review (De-Regil 2016 🡪 Palacio 2019) |
| Medley et al 2018 | Narrative (Cochrane reviews) | Systematic | Preterm birth | - Clear benefit of zinc supplementation for pregnant women without systemic illness on reducing risk of preterm birth - Vitamin D supplements alone for women without health problems may have helped some groups of pregnant women avoid preterm birth, but we have less confidence - Taking vitamin D supplements, calcium and other minerals for pregnant women without health problems may have made things worse for some pregnant women in terms of preterm birth | Two included Cochrane reviews have since been updated (Haider 2017 🡪 Keats 2019; De-Regil 2016 🡪 Palacios 2019) |
| Merialdi et al 2003 | Narrative (Cochrane review and individual RCTs) | Systematic | Fetal growth restriction | - Balanced protein energy supplementation reduced the risk of small for gestational age (SGA) by 30% (95%CI: 20% to 43%) - Calcium supplementation protected against low birth weight (RR 0.83; 95%CI: 0.71–0.98). | A number of the included Cochrane reviews has since been withdrawn (Kramer 2002 Nutritional advice in pregnancy; Kramer 2002 Energy/protein restriction for high weight-for height or weight gain during pregnancy; Duley 1995 Prophylactic fish oil in pregnancy; Mahomed 2002 Folate supplementation in pregnancy; Mahomed 2002 Iron supplementation in pregnancy)  A number of the included Cochrane reviews has since been updated (Hofymeyr 2002 🡪 Hofymeyr 2019; Duley 2002 🡪 2005; Kramer 2002 Balanced protein/energy supplementation in pregnancy 🡪 Ota 2015)  Two included studies did not meet eligibility for current review (Cuervo 2002 because the review examined delivery of iron (oral or intravenous) rather than if iron was effective as a nutritional supplement; Caufield 1998 because not a meta-analysis). |
| Moutquin et al 1994 | Narrative (Cochrane reviews and Medline search for individual RCTs) | Systematic | Hypertensive disorders of pregnancy | - A normal diet without salt restriction is advised. - Promising preventive interventions that may reduce the incidence of gestational hypertension, especially   with proteinuria, include calcium supplementation (2 g/d), fish oil supplementation and low-dose acetylsalicylic acid therapy, particularly in high risk women. | A number of the included Cochrane reviews has since been withdrawn (Kramer 1993 isocaloric balanced protein; Kramer 1993 Energy/protein restriction for high weight-for height or weight gain during pregnancy; Mahomed 1993 Folate supplementation in pregnancy; Mahomed 1993 Iron supplementation in pregnancy)  A number of the included Cochrane reviews has since been updated (Kramer 1993 Balanced protein/energy supplementation in pregnancy 🡪 Ota 2015; Duley 1993 salt 🡪 1999; Dudley 1993 calcium 🡪 Hofymeyr 2019) |
| Mwangi et al 2017 | Narrative (observational, Cochrane review) | Unclear | Iron | - For neonates, there was a reduced prematurity risk, and only a small or no effect on birth weight. - Benefits of antenatal iron   supplementation on vary by maternal iron status. | One included Cochrane review has since been updated (Pena-Rosas 2012 🡪 Pena-Rosas 2015) |
| O’Callaghan and Kiely 2018 | Narrative (observational and reviews of observational studies/RCTs) | Systematic | Vitamin D and hypertensive disorders of pregnancy | - Evidence from RCTs supports a protective effect of combined vitamin D and calcium supplementation against pre-eclampsia incidence - Conflicting data in observational studies highlight caution in dosing in trials. There is a lack of data on long term safety of vitamin D supplementation. | One included Cochrane review has since been updated (De-Regil 2016 🡪 Palacios 2019) |
| Secher 2007 | Narrative (observational and reviews of observational studies/RCTs) | Unclear | Fish oil (omega 3) and preterm birth | - A weak effect of fish oil supplementation on preterm birth rates, which may be due to low compliance. | One included Cochrane reviews has since been updated (Makrides 2006 🡪 Middleton 1998).  Other included meta-analysis (Szajewska 2006) already included in current review. |
| Villar et al 2003 | Narrative (Cochrane reviews and other reviews of RCTs) | Systematic | Preterm birth | - Routine iron supplementation is inconclusive on reducing the rate of preterm birth (statistically non-significant beneficial direction of effect) - Potential for zinc, magnesium, and fish oil supplementation to reduce preterm birth rates but current evidence is limited - Calcium supplementation may reduce the rate of preterm delivery in women at high risk of developing pregnancy hypertension | Lack of clarity on which systematic review contributed to which sub-group and outcome. |
| Villar et al 1998 | Narrative (Cochrane reviews, other reviews and individual RCTs) | Systematic | Preterm birth | - No specific nutrient supplementation was significantly associated with lowered risk of preterm delivery. - Fish oil and vitamins E and C ave potential for preventing preeclampsia and preterm delivery but need further testing. - Calcium supplementation for women at high risk of pregnancy hypertension or low baseline calcium intake reduced incidence of preeclampsia and hypertension. | A number of the included Cochrane reviews has since been withdrawn (Kramer 2002 Nutritional advice in pregnancy;  Kramer 2002 Isocaloric balanced protein supplementation in  Pregnancy; Kramer 2002 Energy/protein restriction for high weight-for heigh  or weight gain during pregnancy; Duley 1995 Prophylactic fish oil in pregnancy; Mahomed 2002 Iron and folate supplementation in pregnancy; Mahomed 2002 Folate supplementation in pregnancy; Mahomed 2002 Iron supplementation in pregnancy)  A number of the included Cochrane reviews has since been updated (Hofymeyr 2002 🡪 Hofymeyr 2019; Makrides 2002 🡪 Makrides 2014; Duley 2002 🡪 2005; Mahomed 2002 Zinc supplementation in pregnancy 🡪 Ota 2015; Kramer 2002 Balanced protein/energy supplementation in pregnancy 🡪 Ota 2015) |
