# Supplementary material for: Effects of Maternal Nutritional Supplements and Dietary Interventions on Placental Complications: An Umbrella Review, Meta-Analysis and Evidence Map
Source: Nutrients. 2021 Jan 30;13(2):472. doi: 10.3390/nu13020472 (PMC7912620; doi:10.3390/nu13020472)
Supplement: Supplementary file 1 [file nutrients-13-00472-s001.zip › Supplementary files/Table S5 - Included review by dietary factor.docx]

**Table S5: Included reviews by dietary factor**

| **Nutrient** | **Type of review** | **Reference** | **Title** |
| --- | --- | --- | --- |
| Vitamin A | Meta-analysis | Kongnyuy et al 2009 | A systematic review of randomized controlled trials of prenatal and postnatal vitamin A supplementation of HIV-infected women |
| Vitamin A | Cochrane meta-analysis | McCauley et al 2015 | Vitamin A supplementation during pregnancy for maternal and newborn outcomes. |
| Vitamin A | Meta-analysis | Thorne-Lyman and Fawzi 2012a | Vitamin A and carotenoids during pregnancy and maternal, neonatal and infant health outcomes: a systematic review and meta-analysis. |
| Vitamin B6 | Cochrane meta-analysis | Salam et al 2015 | Pyridoxine (vitamin B6) supplementation during pregnancy or labour for maternal and neonatal outcomes (Review) |
| Vitamin C | Cochrane meta-analysis | Rumbold et al 2015a | Vitamin C supplementation in pregnancy |
| Vitamin C and E | Meta-analysis | Basaran et al 2010 | Combined vitamin C and E supplementation for the prevention of preeclampsia: a systematic review and meta-analysis. |
| Vitamin C and E | Meta-analysis | Conde-Agudelo et al 2011 | Supplementation with vitamins C and E during pregnancy for the prevention of preeclampsia and other adverse maternal and perinatal outcomes: a systematic review and metaanalysis. |
| Vitamin C and E | Meta-analysis | Polyzos et al 2007 | Combined vitamin C and E supplementation during pregnancy for preeclampsia prevention: a systematic review |
| Vitamin C and E | Meta-analysis | Rahimi et al 2009 | A Meta-Analysis on the Efficacy and Safety of Combined Vitamin C and E Supplementation in Preeclamptic Women |
| Vitamin C and E | Meta-analysis | Rossi et al 2011 | Prevention of pre-eclampsia with low-dose aspirin or vitamins C and E in women at high or low risk: a systematic review with meta-analysis |
| Vitamin C and E | Meta-analysis | Fu et al 2018 | Vitamins supplementation affects the onset of preeclampsia. |
| Vitamin E | Cochrane meta-analysis | Rumbold et al 2015b | Vitamin E supplementation in pregnancy. |
| Vitamin D | Meta-analysis | Bi et al 2018 | Association Between Vitamin D Supplementation During Pregnancy and Offspring Growth, Morbidity, and Mortality: A Systematic Review and Meta-analysis. |
| Vitamin D | Meta-analysis | Fogacci et al 2019 | Vitamin D supplementation and incident preeclampsia: A systematic review and meta-analysis of randomized clinical trials. |
| Vitamin D | Meta-analysis | Fu et al 2018 | Vitamins supplementation affects the onset of preeclampsia. |
| Vitamin D | Meta-analysis | Gallo et al 2019 | Vitamin D Supplementation during Pregnancy: An Evidence Analysis Center Systematic Review and Meta-Analysis. |
| Vitamin D | Meta-analysis | Hypponen et al 2013 | Vitamin D and Pre-Eclampsia: Original Data, Systematic Review and Meta-Analysis. |
| Vitamin D | Meta-analysis | Khaing et al 2017 | Calcium and Vitamin D Supplementation for Prevention of Preeclampsia: A Systematic Review and Network Meta-Analysis. |
| Vitamin D | Meta-analysis | Maugeri et al 2019 | Effects of Vitamin D Supplementation During Pregnancy on Birth Size: A Systematic Review and Meta-Analysis of Randomized Controlled Trials. |
| Vitamin D | Meta-analysis | Palacios et al 2016 | Vitamin D supplementation during pregnancy: Updated meta-analysis on maternal outcomes |
| Vitamin D | Cochrane meta-analysis | Palacios et al 2019 | Vitamin D supplementation for women during pregnancy |
| Vitamin D | Meta-analysis | Perez-Lopez et al 2015 | Effect of vitamin D supplementation during pregnancy on maternal and neonatal outcomes: a systematic review and meta-analysis of randomized controlled trials. |
| Vitamin D | Meta-analysis | Roth et al 2017 | Vitamin D supplementation during pregnancy: state of the evidence from a systematic review of randomised trials |
| Vitamin D | Meta-analysis | Thorne-Lyman and Fawzi 2012b | Vitamin D during pregnancy and maternal, neonatal and infant health outcomes: a systematic review and meta-analysis |
| Vitamin D | Meta-analysis | Zhou et al 2017 | Vitamin D and risk of preterm birth: Up-to-date meta-analysis of randomized controlled trials and observational studies. |
| Vitamin D and calcium | Cochrane meta-analysis | Hofymeyr et al 2018 | Calcium supplementation during pregnancy for preventing hypertensive disorders and related problems. |
| Vitamin D and calcium | Meta-analysis | Khaing et al 2017 | Calcium and Vitamin D Supplementation for Prevention of Preeclampsia: A Systematic Review and Network Meta-Analysis. |
| Vitamin D and calcium | Meta-analysis | Palacios et al 2016 | Vitamin D supplementation during pregnancy: Updated meta-analysis on maternal outcomes |
| Vitamin D and calcium | Cochrane meta-analysis | Palacios et al 2019 | Vitamin D supplementation for women during pregnancy |
| Calcium | Meta-analysis | An et al 2015 | Calcium supplementation reducing the risk of hypertensive disorders of pregnancy and related problems: A meta-analysis of multicentre randomized controlled trials. |
| Calcium | Meta-analysis | Bucher et al 1996 | Effect of calcium supplementation on pregnancy-induced hypertension and preeclampsia: a meta-analysis of randomized controlled trials |
| Calcium | Cochrane meta-analysis | Buppasiri et al 2015 | Calcium supplementation (other than for preventing or treating hypertension) for improving pregnancy and infant outcomes |
| Calcium | Meta-analysis | Carroli et al 1994 | Calcium supplementation during pregnancy: a systematic review of randomised controlled trials. |
| Calcium | Meta-analysis | Hofymeyr et al 2003 | Calcium supplementation to prevent pre-eclampsia-a systematic review. |
| Calcium | Meta-analysis | Hofymeyr et al 2007 | Dietary calcium supplementation for prevention of pre-eclampsia and related problems: a systematic review and commentary. |
| Calcium | Meta-analysis | Hofymeyr et al 2014 | Low-dose calcium supplementation for preventing pre-eclampsia: a systematic review and commentary |
| Calcium | Cochrane meta-analysis | Hofymeyr et al 2018 | Calcium supplementation during pregnancy for preventing hypertensive disorders and related problems. |
| Calcium | Cochrane meta-analysis | Hofymeyr et al 2019 | Calcium supplementation commencing before or early in pregnancy, for preventing hypertensive disorders of pregnancy |
| Calcium | Meta-analysis | Imdad and Bhutta 2012 | Effects of calcium supplementation during pregnancy on maternal, fetal and birth outcomes. |
| Calcium | Meta-analysis | Imdad et al 2011 | Role of calcium supplementation during pregnancy in reducing risk of developing gestational hypertensive disorders: a meta-analysis of studies from developing countries. |
| Calcium | Meta-analysis | Jabeen et al 2011 | Impact of interventions to prevent and manage preeclampsia and eclampsia on stillbirths. |
| Calcium | Meta-analysis | Khaing et al 2017 | Calcium and Vitamin D Supplementation for Prevention of Preeclampsia: A Systematic Review and Network Meta-Analysis. |
| Calcium | Meta-analysis | Kulier et al 1998 | Nutritional interventions for the prevention of maternal morbidity |
| Calcium | Meta-analysis | Park et al 2019 | Association of Early Interventions With Birth Outcomes and Child Linear Growth in Low-Income and Middle-Income Countries: Bayesian Network Meta-analyses of Randomized Clinical Trials. |
| Calcium | Meta-analysis | Patrelli et al 2012 | Calcium supplementation and prevention of preeclampsia: a meta-analysis |
| Calcium | Meta-analysis | Sun et al 2019 | The association between calcium supplement and preeclampsia and gestational hypertension: a systematic review and meta-analysis of randomized trials. |
| Calcium | Meta-analysis | Tang et al 2015 | Limited evidence for calcium supplementation in preeclampsia prevention: a meta-analysis and systematic review. |
| Calcium | Meta-analysis | Villar et al 2000 | Same nutrient, different hypotheses: disparities in trials of calcium supplementation during pregnancy |
| Iodine | Cochrane meta-analysis | Harding et al 2017 | Iodine supplementation for women during the preconception, pregnancy and postpartum period |
| Iron | Meta-analysis | Cantor et al 2015 | Routine iron supplementation and screening for iron deficiency anemia in pregnancy: a systematic review for the U.S. Preventive Services Task Force. |
| Iron | Meta-analysis | Haider et al 2013 | Anaemia, prenatal iron use, and risk of adverse pregnancy outcomes: systematic review and meta-analysis. |
| Iron | Meta-analysis | Park et al 2019 | Association of Early Interventions With Birth Outcomes and Child Linear Growth in Low-Income and Middle-Income Countries: Bayesian Network Meta-analyses of Randomized Clinical Trials. |
| Iron | Cochrane meta-analysis | Pena-Rosas et al 2015 | Daily oral iron supplementation during pregnancy. |
| Folic acid | Cochrane meta-analysis | De-Regil et al 2015 | Effects and safety of periconceptional oral folate supplementation for preventing birth defects |
| Folic acid | Meta-analysis | Hua et al 2016 | Effect of folic acid supplementation during pregnancy on gestational hypertension/preeclampsia: A systematic review and meta-analysis |
| Folic acid | Cochrane meta-analysis | Lassi et al 2013 | Folic acid supplementation during pregnancy for maternal health and pregnancy outcomes. |
| Folic acid | Meta-analysis | Park et al 2019 | Association of Early Interventions With Birth Outcomes and Child Linear Growth in Low-Income and Middle-Income Countries: Bayesian Network Meta-analyses of Randomized Clinical Trials. |
| Folic acid | Meta-analysis | Saccone and Berghella 2016 | Folic acid supplementation in pregnancy to prevent preterm birth: a systematic review and meta-analysis of randomized controlled trials. |
| Iron-folic acid | Meta-analysis | Kulier et al 1998 | Nutritional interventions for the prevention of maternal morbidity |
| Iron-folic acid | Meta-analysis | Park et al 2019 | Association of Early Interventions With Birth Outcomes and Child Linear Growth in Low-Income and Middle-Income Countries: Bayesian Network Meta-analyses of Randomized Clinical Trials. |
| Magnesium | Cochrane meta-analysis | Makrides and Crowther 2014 | Magnesium supplementation in pregnancy |
| Zinc | Meta-analysis | Chaffee et al 2012 | Effect of zinc supplementation on pregnancy and infant outcomes: a systematic review. |
| Zinc | Cochrane meta-analysis | Ota et al 2015a | Zinc supplementation for improving pregnancy and infant outcome |
| Zinc | Meta-analysis | Park et al 2019 | Association of Early Interventions With Birth Outcomes and Child Linear Growth in Low-Income and Middle-Income Countries: Bayesian Network Meta-analyses of Randomized Clinical Trials. |
| Antioxidants | Cochrane meta-analysis | Rumbold et al 2008 | Antioxidants for preventing pre-eclampsia |
| Antioxidants | Meta-analysis | Salles et al 2008 | Antioxidants for Preventing Preeclampsia: A Systematic Review |
| Antioxidants | Meta-analysis | Tenorio et al 2018 | Oral antioxidant therapy for prevention and treatment of preeclampsia: Meta-analysis of randomized controlled trials. |
| Garlic | Cochrane Meta-analysis | Meher and Duley 2010 | Garlic for preventing pre-eclampsia and its complications |
| L'arginine | Meta-analysis | Dorniak-Wall et al 2014 | The role of L-arginine in the prevention and treatment of pre-eclampsia: a systematic review of randomised trials |
| L'arginine | Meta-analysis | Gui et al 2014 | Arginine supplementation for improving maternal and neonatal outcomes in hypertensive disorder of pregnancy: a systematic review |
| Multiple micronutrients | Meta-analysis | Fall et al 2009 | Multiple micronutrient supplementation during pregnancy in low-income countries: a meta-analysis of effects on birth size and length of gestation. |
| Multiple micronutrients | Meta-analysis | Fu et al 2018 | Vitamins supplementation affects the onset of preeclampsia. |
| Multiple micronutrients | Meta-analysis | Haider et al 2011 | Effect of multiple micronutrient supplementation during pregnancy on maternal and birth outcomes |
| Multiple micronutrients | Meta-analysis | Kawai et al 2011 | Maternal multiple micronutrient supplementation and pregnancy outcomes in developing countries: meta-analysis and meta-regression. |
| Multiple micronutrients | Cochrane meta-analysis | Keats et al 2019 | Multiple-micronutrient supplementation for women during pregnancy |
| Multiple micronutrients | Meta-analysis | Park et al 2019 | Association of Early Interventions With Birth Outcomes and Child Linear Growth in Low-Income and Middle-Income Countries: Bayesian Network Meta-analyses of Randomized Clinical Trials. |
| Multiple micronutrients | Meta-analysis | Ronsmans et al 2009 | Multiple micronutrient supplementation during pregnancy in low-income countries: a meta-analysis of effects on stillbirths and on early and late neonatal mortality |
| Multiple micronutrients | Meta-analysis | Shah et al 2009 | Effects of prenatal multimicronutrient supplementation on pregnancy outcomes: a meta-analysis |
| Multiple micronutrients | Meta-analysis | Smith et al 2017 | Modifiers of the effect of maternal multiple micronutrient supplementation on stillbirth, birth outcomes, and infant mortality: a meta-analysis of individual patient data from 17 randomised trials in low-income and middle-income countries |
| Polyunsaturated omega-3 fatty acid | Meta-analysis | Allen et al 2014 | Effect of diet- And lifestyle-based metabolic risk-modifying interventions on preeclampsia: A meta-analysis |
| Polyunsaturated omega-3 fatty acid | Meta-analysis | Chen et al 2015 | Fish Oil Supplementation does not Reduce Risks of Gestational Diabetes Mellitus, Pregnancy-Induced Hypertension, or Pre-Eclampsia: A Meta-Analysis of Randomized Controlled Trials. |
| Polyunsaturated omega-3 fatty acid | Meta-analysis | Chen et al 2016 | Fish oil supplementation improves pregnancy outcomes and size of the newborn: a meta-analysis of 21 randomized controlled trials. |
| Polyunsaturated omega-3 fatty acid | Meta-analysis | Horvath et al 2007 | Effect of supplementation of women in high-risk pregnancies with long-chain polyunsaturated fatty acids on pregnancy outcomes and growth measures at birth: a meta-analysis of randomized controlled trials. |
| Polyunsaturated omega-3 fatty acid | Meta-analysis | Imhoff-Kunsch et al 2015 | Effect of n-3 long-chain polyunsaturated fatty acid intake during pregnancy on maternal, infant, and child health outcomes: a systematic review. |
| Polyunsaturated omega-3 fatty acid | Meta-analysis | Kar et al 2015 | Effects of omega-3 fatty acids in prevention of early preterm delivery: a systematic review and meta-analysis of randomized studies. |
| Polyunsaturated omega-3 fatty acid | Cochrane meta-analysis | Middleton et al 2018 | Omega-3 fatty acid addition during pregnancy |
| Polyunsaturated omega-3 fatty acid | Meta-analysis | Newberry et al 2016 | Omega-3 Fatty Acids and Maternal and Child Health: An Updated Systematic Review |
| Polyunsaturated omega-3 fatty acid | Meta-analysis | Saccone et al 2015 | Omega-3 supplementation during pregnancy to prevent recurrent intrauterine growth restriction: systematic review and meta-analysis of randomized controlled trials. |
| Polyunsaturated omega-3 fatty acid | Meta-analysis | Saccone and Berghella 2015a | Omega-3 long chain polyunsaturated fatty acids to prevent preterm birth: a systematic review and meta-analysis. |
| Polyunsaturated omega-3 fatty acid | Meta-analysis | Saccone and Berghella 2015b | Omega-3 supplementation to prevent recurrent preterm birth: a systematic review and metaanalysis of randomized controlled trials. |
| Polyunsaturated omega-3 fatty acid | Meta-analysis | Saccone et al 2016 | Omega-3 long-chain polyunsaturated fatty acids and fish oil supplementation during pregnancy: which evidence? |
| Polyunsaturated omega-3 fatty acid | Meta-analysis | Salvig et al 2011 | Evidence regarding an effect of marine n-3 fatty acids on preterm birth: a systematic review and meta-analysis |
| Polyunsaturated omega-3 fatty acid | Meta-analysis | Szajewska et al 2006 | Effect of n-3 long-chain polyunsaturated fatty acid supplementation of women with low-risk pregnancies on pregnancy outcomes and growth measures at birth: a meta-analysis of randomized controlled trials. |
| Protein-energy / high protein supplementation | Cochrane meta-analysis | Ota et al 2015b | Antenatal dietary education and supplementation to increase energy and protein intake. |
| Calf blood extract supplementation | Cochrane meta-analysis | Say et al 2003 | Maternal nutrient supplementation for suspected impaired fetal growth. |
| Glucose supplementation | Cochrane meta-analysis | Say et al 2003 | Maternal nutrient supplementation for suspected impaired fetal growth. |
| Galactose supplementation | Cochrane meta-analysis | Say et al 2003 | Maternal nutrient supplementation for suspected impaired fetal growth. |
| Lipid-based supplementation | Cochrane meta-analysis | Das et al 2018 | Lipid-based nutrient supplements for maternal, birth, and infant developmental outcomes. |
| Lipid-based supplementation | Meta-analysis | Goto 2019 | Effectiveness of Prenatal Lipid-Based Nutrient Supplementation to Improve Birth Outcomes: A Meta-analysis |
| Food and fortified food products | Meta-analysis | Gresham et al 2016 | Effects of dietary interventions on pregnancy outcomes: a systematic review and meta-analysis. |
| Dietary salt restriction | Cochrane meta-analysis | Duley et al 2005 | Altered dietary salt for preventing pre-eclampsia, and its complications. |
| Dietary caffine restriction | Cochrane meta-analysis | Jahanfar and Jaafar 2015 | Effects of restricted caffeine intake by mother on fetal, neonatal and pregnancy outcomes |
| Diet and nutritional counselling | Meta-analysis | Allen et al 2014 | Effect of diet- And lifestyle-based metabolic risk-modifying interventions on preeclampsia: A meta-analysis |
| Diet and nutritional counselling | Meta-analysis | Gresham et al 2014 | Effects of dietary interventions on neonatal and infant outcomes: a systematic review and meta-analysis. |
| Diet and nutritional counselling | Meta-analysis | Gresham et al 2016 | Effects of dietary interventions on pregnancy outcomes: a systematic review and meta-analysis. |
| Diet and nutritional counselling | Cochrane meta-analysis | Ota et al 2015 | Antenatal dietary education and supplementation to increase energy and protein intake. |
| Diet and nutritional counselling | Meta-analysis | Syngelaki et al 2019 | Diet and exercise for preeclampsia prevention in overweight and obese pregnant women: systematic review and meta-analysis. |
| Diet and nutritional counselling | Meta-analysis | Thangaratinam et al 2012 | Effects of interventions in pregnancy on maternal weight and obstetric outcomes: meta-analysis of randomised evidence |
| Diet and nutritional counselling | Meta-analysis | Zhang et al 2018 | Effects of low-glycemic-index diets in pregnancy on maternal and newborn outcomes in pregnant women: a meta-analysis of randomized controlled trials. |
