# Supplementary material for: Effects of Maternal Nutritional Supplements and Dietary Interventions on Placental Complications: An Umbrella Review, Meta-Analysis and Evidence Map
Source: Nutrients. 2021 Jan 30;13(2):472. doi: 10.3390/nu13020472 (PMC7912620; doi:10.3390/nu13020472)
Supplement: Supplementary file 1 [file nutrients-13-00472-s001.zip › Supplementary files/Table S6 - Quality assessment for included reviews.docx]

**Table S6 – AMSTAR 2 quality assessment**

1. Did the research questions and inclusion criteria for the review include the components of PICO?
2. Did the report of the review contain an explicit statement that the review methods were established prior to the conduct of the review and did the report justify any significant deviations from the protocol?
3. Did the review authors explain their selection of the study designs for inclusion in the review?
4. Did the review authors use a comprehensive literature search strategy?
5. Did the review authors perform study selection in duplicate?
6. Did the review authors perform data extraction in duplicate?
7. Did the review authors provide a list of excluded studies and justify the exclusions?
8. Did the review authors describe the included studies in adequate detail?
9. Did the review authors use a satisfactory technique for assessing the risk of bias (RoB) in individual studies that were included in the review?
10. Did the review authors report on the sources of funding for the studies included in the review?"
11. If meta-analysis was performed did the review authors use appropriate methods for statistical combination of results?
12. If meta-analysis was performed, did the review authors assess the potential impact of RoB in individual studies on the results of the meta-analysis or other evidence synthesis?
13. Did the review authors account for RoB in individual studies when interpreting/ discussing the results of the review?
14. Did the review authors provide a satisfactory explanation for, and discussion of, any heterogeneity observed in the results of the review?
15. If they performed quantitative synthesis did the review authors carry out an adequate investigation of publication bias (small study bias) and discuss its likely impact on the results of the review?"
16. Did the review authors report any potential sources of conflict of interest, including any funding they received for conducting the review?

| **Reference** | **1** | **2** | **3** | **4** | **5** | **6** | **7** | **8** | **9** | **10** | **11** | **12** | **13** | **14** | **15** | **16** | **Review quality** |
| --- | --- | --- | --- | --- | --- | --- | --- | --- | --- | --- | --- | --- | --- | --- | --- | --- | --- |
| Allen et al 2014 | Y | Y | N | PY | Y | Y | PY | Y | Y | N | Y | Y | Y | Y | Y | Y | Moderate |
| An et al 2015 | Y | N | PY | Y | Y | Y | Y | Y | PY | N | Y | Y | Y | Y | Y | Y | Moderate |
| Basaran et al 2010 | Y | N | N | PY | Y | Y | PY | Y | PY | N | Y | Y | Y | Y | Y | Y | Moderate |
| Bi et al 2018 | Y | N | N | PY | Y | Y | PY | Y | Y | N | Y | Y | Y | Y | Y | Y | Moderate |
| Buppasiri et al 2015 | Y | Y | Y | Y | Y | Y | Y | Y | Y | Y | Y | Y | Y | Y | Y | Y | High |
| Burcher et al 1996 | N | N | N | N | Y | N | N | N | N | N | N | N | N | Y | N | N | Critically low |
| Cantor et al 2015 | Y | N | N | PY | Y | N | PY | N | N | N | Y | N | Y | N | N | Y | Low |
| Carroli et al 1994 | N | N | Y | N | N | N | Y | PY | PY | N | N | N | Y | N | N | N | Critically low |
| Chaffee and King 2012 | Y | N | N | PY | Y | N | N | PY | Y | N | Y | N | Y | N | Y | Y | Moderate |
| Chen et al 2015 | Y | N | N | PY | Y | Y | PY | Y | Y | N | Y | N | N | Y | Y | Y | Low |
| Chen et al 2016 | Y | N | N | PY | Y | Y | PY | Y | Y | N | Y | N | N | Y | Y | Y | Low |
| Conde-Agudelo et al 2011 | Y | PY | N | Y | Y | Y | PY | Y | Y | N | Y | Y | Y | Y | Y | N | Moderate |
| Das et al 2018 | Y | Y | N | Y | Y | Y | Y | Y | Y | Y | Y | Y | Y | Y | Y | Y | High |
| De-Regil et al 2015 | Y | Y | Y | Y | Y | Y | Y | Y | Y | Y | Y | Y | Y | Y | Y | Y | High |
| Dorniak-Wall et al 2014 | Y | N | N | PY | Y | N | Y | PY | Y | N | Y | N | N | N | N | Y | Critically low |
| Duley et al 2005 | Y | Y | Y | Y | Y | Y | Y | Y | PY | N | Y | Y | Y | Y | Y | Y | Moderate |
| Fall et al 2009 | N | N | N | N | N | N | Y | N | N | N | Y | N | N | N | N | N | Critically low |
| Fogacci et al 2019 | Y | Y | N | PY | Y | Y | Y | PY | Y | N | Y | Y | Y | Y | Y | Y | Moderate |
| Fu et al 2018 | Y | N | N | PY | N | Y | PY | PY | N | N | Y | N | N | N | Y | Y | Critically low |
| Gallo et al 2019 | Y | N | Y | N | Y | N | PY | Y | PY | N | Y | N | N | Y | Y | Y | Critically low |
| Goto et al 2019 | Y | N | N | PY | N | N | PY | PY | Y | N | Y | Y | Y | Y | Y | Y | Moderate |
| Gresham et al 2014 | Y | N | N | PY | Y | Y | PY | PY | PY | N | Y | Y | Y | Y | Y | Y | Moderate |
| Gresham et al 2016 | Y | N | N | PY | Y | Y | PY | N | PY | N | Y | Y | Y | Y | Y | Y | Moderate |
| Gui et al 2014 | Y | N | N | PY | Y | Y | PY | Y | Y | N | Y | Y | Y | Y | N | Y | Moderate |
| Haider et al 2011 | Y | N | Y | Y | N | Y | N | Y | Y | N | Y | Y | Y | Y | N | Y | Moderate |
| Haider et al 2013 | Y | N | Y | Y | Y | Y | Y | Y | PY | N | Y | Y | Y | Y | Y | Y | Moderate |
| Harding et al 2017 | Y | Y | N | Y | Y | N | Y | Y | Y | Y | Y | Y | Y | Y | Y | Y | Moderate |
| Hofmeyr et al 2003 | Y | N | N | N | N | N | N | N | N | N | Y | N | Y | N | N | N | Critically low |
| Hofmeyr et al 2007 | Y | N | N | Y | Y | Y | PY | Y | PY | N | Y | Y | Y | N | N | Y | Moderate |
| Hofmeyr et al 2013 | Y | N | N | Y | Y | Y | N | Y | PY | N | Y | Y | Y | N | N | Y | Moderate |
| Hofmeyr et al 2018 | Y | Y | Y | Y | Y | Y | Y | Y | Y | Y | Y | Y | Y | Y | Y | Y | High |
| Hofmeyr et al 2019 | Y | Y | Y | Y | Y | Y | Y | Y | Y | Y | Y | Y | Y | Y | Y | Y | High |
| Horvath et al 2007 | Y | N | N | PY | Y | Y | Y | PY | PY | N | Y | Y | Y | N | N | N | Moderate |
| Hua et al 2016 | Y | N | Y | PY | Y | N | N | PY | PY | N | Y | Y | Y | Y | Y | Y | Moderate |
| Hyppönen et al 2013 | Y | N | N | PY | Y | N | PY | N | N | N | Y | N | N | Y | Y | Y | Critically low |
| Imdad and Bhutta 2012 | Y | N | N | PY | N | N | Y | PY | Y | N | Y | Y | Y | N | Y | Y | Moderate |
| Imdad et al 2011 | Y | N | Y | PY | N | N | Y | PY | N | N | Y | Y | Y | Y | Y | Y | Low |
| Imhoff-Kunsch et al 2015 | Y | N | Y | PY | N | N | N | Y | Y | N | Y | N | Y | N | N | Y | Moderate |
| Jabeen et al 2011 | Y | N | N | PY | N | Y | N | PY | PY | N | Y | N | N | Y | N | Y | Low |
| Jahanfar and Jaafar 2015 | Y | Y | N | Y | Y | Y | Y | Y | Y | N | Y | Y | Y | Y | Y | Y | Moderate |
| Kar et al 2015 | Y | N | N | PY | Y | Y | PY | PY | Y | N | Y | Y | Y | Y | Y | Y | Moderate |
| Kawai et al 2011 | Y | N | N | PY | N | N | PY | PY | N | N | Y | N | N | Y | Y | Y | Critically low |
| Keats et al 2019 | Y | Y | N | Y | Y | Y | Y | Y | Y | Y | Y | Y | Y | Y | Y | Y | High |
| Khaing et al 2017 | Y | Y | N | PY | Y | Y | PY | PY | Y | N | Y | N | Y | Y | Y | Y | Moderate |
| Kongnyuy et al 2009 | Y | N | N | N | Y | N | N | Y | N | N | N | N | N | N | N | N | Critically low |
| Kulier et al 1998 | N | N | N | N | N | N | N | PY | N | N | N | N | N | N | N | N | Critically low |
| Lassi et al 2013 | Y | Y | Y | Y | Y | Y | Y | Y | Y | N | Y | Y | Y | Y | Y | Y | High |
| Makrides and Crowther 2014 | Y | Y | N | Y | Y | Y | Y | Y | Y | N | Y | Y | Y | Y | Y | Y | Moderate |
| Maugeri et al 2019 | Y | N | Y | PY | Y | Y | Y | Y | Y | N | Y | Y | Y | Y | Y | Y | Moderate |
| McCauley et al 2015 | Y | Y | N | Y | Y | Y | Y | Y | Y | N | Y | Y | Y | Y | Y | Y | Moderate |
| Meher and Duley 2006 | Y | Y | N | Y | Y | Y | Y | Y | PY | N | Y | Y | Y | Y | Y | Y | Moderate |
| Middleton et al 2018 | Y | Y | Y | Y | Y | Y | Y | Y | Y | Y | Y | Y | Y | Y | Y | Y | High |
| Newberry et al 2016 | Y | PY | N | PY | Y | N | Y | Y | Y | Y | Y | N | Y | Y | Y | Y | Moderate |
| Ota et al 2015a (zinc) | Y | Y | Y | Y | Y | Y | Y | Y | Y | N | Y | Y | Y | Y | Y | Y | High |
| Ota et al 2015b (energy/protein) | Y | Y | N | Y | Y | Y | Y | Y | Y | N | Y | Y | Y | Y | Y | Y | Moderate |
| Palacios et al 2016 | Y | PY | Y | Y | Y | N | Y | Y | PY | N | Y | Y | Y | N | Y | Y | Moderate |
| Palacios et al 2019 | Y | Y | Y | Y | Y | Y | Y | Y | Y | Y | Y | Y | Y | Y | Y | Y | High |
| Park et al 2019 | Y | Y | N | PY | Y | Y | Y | Y | Y | N | Y | Y | Y | Y | Y | Y | Moderate |
| Patrelli et al 2012 | Y | N | N | N | N | N | N | N | N | N | N | N | N | N | N | Y | Critically low |
| Peña-Rosas et al 2015 | Y | Y | Y | Y | Y | Y | Y | Y | Y | N | Y | Y | Y | Y | Y | Y | High |
| Perez-Lopez et al 2015 | Y | N | N | Y | Y | Y | PY | Y | Y | Y | Y | Y | Y | Y | Y | Y | Moderate |
| Polyzos et al 2007 | Y | N | Y | PY | Y | N | N | N | PY | N | Y | Y | Y | Y | Y | Y | Moderate |
| Rahimi et al 2009 | Y | N | N | PY | Y | Y | Y | N | PY | N | N | Y | Y | Y | Y | Y | Low |
| Ronsmans et al 2009 / Margetts et al 2009 | Y | N | N | N | N | N | N | Y | N | N | Y | N | N | Y | N | N | Critically low |
| Rossi and Mullin 2011 | Y | N | N | PY | Y | N | N | N | N | N | N | N | N | N | Y | N | Critically low |
| Roth et al 2017 | Y | Y | Y | Y | Y | Y | Y | Y | Y | N | Y | Y | Y | Y | Y | Y | High |
| Rumbold et al 2008 | Y | Y | Y | Y | Y | Y | Y | Y | PY | N | Y | Y | Y | Y | Y | Y | High |
| Rumbold et al 2015a (vitamin C) | Y | Y | N | Y | Y | Y | Y | Y | Y | N | Y | Y | Y | Y | Y | Y | Moderate |
| Rumbold et al 2015 (vitamin E) | Y | Y | N | Y | Y | Y | Y | Y | Y | N | Y | Y | Y | Y | Y | Y | Moderate |
| Saccone and Berghella 2015a (preterm birth) | Y | Y | N | PY | N | Y | Y | Y | Y | N | Y | Y | Y | Y | Y | N | Moderate |
| Saccone and Berghella 2015b (preterm birth in women with previous preterm birth | Y | PY | N | Y | N | N | Y | Y | Y | N | Y | Y | Y | N | Y | Y | Moderate |
| Saccone and Berghella 2016 | Y | PY | N | PY | N | Y | Y | Y | Y | N | Y | N | Y | Y | Y | Y | Moderate |
| Saccone et al 2015 | Y | Y | Y | Y | Y | Y | Y | Y | Y | N | Y | Y | Y | Y | Y | N | Moderate |
| Saccone et al 2016 | Y | PY | Y | Y | Y | Y | Y | PY | Y | N | Y | Y | Y | Y | Y | Y | High |
| Salam et al 2015 | Y | Y | N | Y | Y | Y | Y | Y | Y | N | Y | Y | Y | Y | Y | Y | Moderate |
| Salles et al 2012 | Y | N | N | PY | Y | Y | Y | Y | PY | N | Y | Y | Y | Y | Y | Y | Moderate |
| Salvig and Lamont 2011 | Y | N | N | PY | Y | Y | Y | Y | PY | N | N | Y | Y | N | N | Y | Low |
| Say et al 2003 | Y | Y | N | Y | Y | Y | Y | Y | PY | N | Y | N | Y | Y | Y | Y | Moderate |
| Shah et al 2009 | Y | N | Y | PY | Y | Y | Y | Y | Y | N | Y | Y | Y | Y | Y | Y | Moderate |
| Smith et al 2017 | Y | PY | N | Y | N | N | N | Y | Y | N | Y | Y | Y | Y | Y | Y | Moderate |
| Sun et al 2019 | Y | N | N | PY | Y | Y | PY | Y | N | Y | Y | Y | Y | Y | Y | Y | Moderate |
| Syngelaki et al 2019 | Y | Y | N | PY | Y | N | N | Y | Y | N | Y | N | Y | Y | Y | Y | Moderate |
| Szajewska, Borvath and Kolezko 2006 | Y | N | Y | PY | N | Y | Y | Y | Y | N | Y | Y | Y | Y | Y | Y | Moderate |
| Tang et al 2015 | Y | N | N | PY | Y | Y | PY | Y | Y | N | Y | N | N | Y | Y | Y | Low |
| Tentorio et al 2018 | Y | Y | N | Y | Y | Y | PY | PY | Y | N | Y | N | Y | Y | Y | Y | Moderate |
| Thangaratinam et al 2012 | Y | N | N | Y | Y | Y | PY | Y | Y | N | Y | Y | Y | Y | Y | Y | Moderate |
| Thorne-Lyman and Fawzi 2012a (vitamin A) | N | N | Y | PY | N | N | PY | Y | N | N | Y | Y | Y | Y | N | N | Low |
| Thorne-Lyman and Fawzi 2012b (vitamin D) | N | N | Y | PY | N | N | PY | Y | N | N | Y | Y | Y | Y | N | N | Low |
| Villar and Belizan 2000 | N | N | N | N | N | N | N | N | PY | N | N | N | Y | N | N | N | Critically low |
| Zhang et al 2018 | Y | N | N | PY | Y | Y | Y | PY | PY | N | Y | Y | N | Y | Y | Y | Low |
| Zhou et al 2017 | Y | N | Y | PY | N | Y | PY | Y | Y | N | Y | Y | Y | Y | Y | Y | Moderate |
