# Supplementary material for: Effects of Maternal Nutritional Supplements and Dietary Interventions on Placental Complications: An Umbrella Review, Meta-Analysis and Evidence Map
Source: Nutrients. 2021 Jan 30;13(2):472. doi: 10.3390/nu13020472 (PMC7912620; doi:10.3390/nu13020472)
Supplement: Supplementary file 1 [file nutrients-13-00472-s001.zip › Supplementary files/Table S7 - Summary of outcomes by reviews.docx]

**Table S7 – Summary of outcomes by reviews**

Contents

[Pre-eclampsia (PE) 3](#_Toc55376154)

[Nutrient supplementation 3](#_Toc55376155)

[Dietary intervention 8](#_Toc55376156)

[Severe pre-eclampsia (Severe PE) 8](#_Toc55376157)

[Nutrient supplementation 8](#_Toc55376158)

[Dietary intervention 9](#_Toc55376159)

[Gestational hypertension (GH) 10](#_Toc55376160)

[Nutrient supplementation 10](#_Toc55376161)

[Dietary intervention 12](#_Toc55376162)

[Eclampsia 12](#_Toc55376163)

[Nutrient supplementation 12](#_Toc55376164)

[Dietary intervention 13](#_Toc55376165)

[HELLP syndrome 13](#_Toc55376166)

[Nutrient supplementation 13](#_Toc55376167)

[Dietary intervention 14](#_Toc55376168)

[Pregnancy hypertension general (hypertensive diseases of pregnancy (HDP), pregnancy-induced hypertension (PIH), multiple pregnancy hypertension conditions) 14](#_Toc55376169)

[Nutrient supplementation 14](#_Toc55376170)

[Dietary intervention 15](#_Toc55376171)

[Preterm birth (PTB) 16](#_Toc55376172)

[Nutrient supplementation 16](#_Toc55376173)

[Dietary intervention 23](#_Toc55376174)

[Small for gestational age (SGA) 24](#_Toc55376175)

[Nutrient supplementation 24](#_Toc55376176)

[Dietary intervention 29](#_Toc55376177)

[Low birth weight (LBW) 30](#_Toc55376178)

[Nutrient supplementation 30](#_Toc55376179)

[Dietary intervention 35](#_Toc55376180)

[Stillbirth 35](#_Toc55376181)

[Nutrient supplementation 35](#_Toc55376182)

[Dietary intervention 38](#_Toc55376183)

[Maternal mortality 38](#_Toc55376184)

[Nutrient supplementation 38](#_Toc55376185)

[Dietary intervention 40](#_Toc55376186)

# Pre-eclampsia (PE)

## Nutrient supplementation

| **Reference** | **Nutritional supplement or dietary intervention** | **Outcome** | **No. of RCT studies** | **No. participants** | **Variation between studies (I^2^)** | **Review pooled results (95% confidence intervals)** | **Strength of association (Harvard Cancer Index)** | **Quality assessment (AMSTAR2)** |
| --- | --- | --- | --- | --- | --- | --- | --- | --- |
| Salam et al 2015 | Vitamin B6 (pyridoxine) | PE | 2 | 1197 | NA | RR 1.71 (0.85-3.45) | NA – not significant effect | Moderate |
| Rumbold et al 2015a | Vitamin C (alone or with other supplements) | PE | 16 | 21,956 | 41% | RR 0.92 (0.80-1.05) | NA – not significant effect | Moderate |
| Rumbold et al 2015b | Vitamin E (alone or with other supplements) | PE | 14 | 20,878 | 48% | RR 0.91 (0.80-1.05) | NA – not significant effect | Moderate |
| Polyzos et al 2007 | Vitamin C and E | PE | 4 | 4,680 | 49% | RR 0.97 (0.82–1.13) | NA – not significant effect | Moderate |
| Rahimi et al 2009 | Vitamin C and E (high risk women) | PE | 7 | 5,869 | NA | RR 0.7 (0.58–1.08) | NA – not significant effect | Low |
| Basaran et al 2010 | Vitamin C and E | PE | 9 | 19,675 | 35% | RR: 0.98 (0.87–1.10) | NA – not significant effect | Moderate |
| Conde-Agudelo et al 2011 | Vitamin C and E | PE | 9 | 19,810 | 13% | RR 1.00 (0.92–1.09) | NA – not significant effect | Moderate |
| Rossi and Mullin 2011 | Vitamin C and E (high risk women) | PE | 4 | 3,485 | 32% | OR 0.84 (0.63-1.12) | NA – not significant effect | Critically low |
| Dror and Allen 2012 | Vitamins C and E | PE | 9 | 19,798 | 14% | RR 1.00 (0.91–1.10) | NA – not significant effect | Critically low |
| Fu et al 2018 | Vitamin C and E | PE | 11 | 20,665 | 0% | OR 0.94 (0.87-1.02) | NA – not significant effect | Critically low |
| Hyppönen et al 2013 | Vitamin D | PE | 4 | NA | 0% | OR 0.66 (0.52–0.83) | Moderate | Critically low |
| Perez-Lopez et al 2015 | Vitamin D | PE | 3 | 654 | 24% | RR 0.88 (0.51-1.52) | NA – not significant effect | Moderate |
| Palacios et al 2016 | Vitamin D | PE | 2 | 219 | NA | RR 0.52 (0.25-1.05) | NA – not significant effect | Moderate |
| Khaing et al 2017 | Vitamin D | PE | 3 | 357 | 0% | RR 0.47 (0.24-0.89) | Moderate | Moderate |
| Roth et al 2017 | Vitamin D | PE | 3 | 706 | 67% | RR 1.09 (0.43-2.76) | NA – not significant effect | High |
| Fu et al 2018 | Vitamin D | PE | 5 | 364 | 0% | RR 0.41 (0.22-0.78) | Moderate | Critically low |
| Gallo et al 2019 | Vitamin D | PE | 5 | 640 | 16% | OR 0.69 (0.35-1.39) | NA – not significant effect | Critically low |
| Fogacci et al 2019 | Vitamin D | PE | 12 | 1676 | 0% | OR 0.37 (0.26- 0.52) | Strong | Moderate |
| Palacios et al 2019 | Vitamin D | PE | 4 | 499 | 0% | RR 0.48 (0.30-0.79) | Moderate | High |
| Palacios et al 2016 | Vitamin D and calcium | PE | 3 | 1114 | NA | RR 0.51 (0.32- 0.80) | Moderate | Moderate |
| Khaing et al 2017 | Vitamin D and calcium | PE | 4 | 1169 | 0% | RR 0.50 (0.32-0.78) | Moderate | Moderate |
| Hofmeyr et al 2018 | Vitamin D and calcium (low dose, <1g/day) | PE | 2 | 1060 | 17% | RR 0.49 (0.31-0.78) | Moderate | High |
| Palacios et al 2019 | Vitamin D and calcium | PE | 4 | 1174 | 0% | RR 0.50 (0.32-0.78) | Moderate | High |
| Carroli et al 1994 | Calcium | PE | 6 | 1,729 | NA | OR 0.34 (0.22-0.54) | Strong | Critically low |
| Bucher et al 1996 | Calcium | PE | 9 | 2,260 | NA | OR 0.38 (0.22-0.65) | Strong | Critically low |
| Kulier et al 1998 | Calcium | PE | 9 | 6408 | NA | RR 0.72 (0.60-0.86) | Weak | Critically low |
| Villar and Belizan 2000 | Calcium (low risk women) | PE | 6 | 6307 | NA | RR 0.79  (0.6-0.94) | Weak | Critically low |
| Villar and Belizan 2000 | Calcium (high risk women) | PE | 4 | 557 | NA | RR 0.22 (0.11- 0.43) | Strong | Critically low |
| Villar and Belizan 2000 | Calcium (adequate baseline intake) | PE | 4 | 4793 | NA | RR 0.86 (0.71- 1.05) | NA – not significant effect | Critically low |
| Villar and Belizan 2000 | Calcium (low baseline intake) | PE | 6 | 1842 | NA | RR 0.32 (0.21-0.49) | Strong | Critically low |
| Hofymeyr et al 2003 | Calcium | PE | 11 | 6894 | NA | RR 0.68 (0.57-0.81) | Moderate | Critically low |
| Hofmeyr et al 2007 | Calcium (high dose, ≥1g/day) | PE | 12 | 15206 | 68% | RR 0.48 (0.33-0.69) | Moderate | Moderate |
| Imdad and Bhutta 2011 | Calcium (developing countries) | PE | 10 | 11405 | 74% | RR 0.41 (0.24-0.69) | Moderate | Low |
| Imdad and Bhutta 2012 | Calcium | PE | 15 | 941 | 70% | RR 0.48 (0.34-0.67) | Moderate | Moderate |
| Patrelli et al 2012 | Calcium (adequate baseline intake) | PE | 6 | 9641 | 46% | RR 0.88 (0.77-1.02) | NA – not significant effect | Critically low |
| Patrelli et al 2012 | Calcium (low baseline intake) | PE | 7 | 10154 | 75% | RR 0.73 (0.61-0.87) | Weak | Critically low |
| Hofmeyr et al 2013 | Calcium (low dose, <1g/day) | PE | 9 | 2234 | 0% | RR 0.38 (0.28-0.52) | Strong | Moderate |
| An et al 2015 | Calcium | PE | 4 | 14,524 | 39% | RR 0.89 (0.77–1.02) | NA – not significant effect | Moderate |
| Tang et al 2015 | Calcium | PE | 10 | 24787 | 73% | RR 0.62 (0.49-0.81) | Moderate | Low |
| Khaing et al 2017 | Calcium | PE | 16 | 25936 | 73% | RR 0.54 (0.41-0.70) | Moderate | Moderate |
| Hofmeyr et al 2018 | Calcium (high dose, ≥1g/day) | PE | 13 | 15,730 | 70% | RR 0.45 (0.31-0.65) | Moderate | High |
| Hofmeyr et al 2018 | Calcium (low dose, <1g/day) | PE | 4 | 980 | 0% | RR 0.36 (0.23-0.57) | Strong | High |
| Hofmeyr et al 2019 | Calcium (pre/early pregnancy) | PE | 1 | 579 | NA | RR 0.80 (0.61- 1.06) | NA – not significant effect | High |
| Sun et al 2019 | Calcium | PE | 25 | 27770 | 66% | RR 0.51 (0.40-0.64) | Moderate | Moderate |
| Peña-Rosas et al 2015 | Iron | PE | 1 | 47 | NA | RR 0.96 (0.06-14.43) | NA – not significant effect | High |
| Peña-Rosas et al 2015 | Iron (with or without folic acid) | PE | 4 | 1704 | 0% | RR 1.63 (0.87-3.07) | NA – not significant effect | High |
| Peña-Rosas et al 2015 | Iron and folic acid | PE | 1 | 48 | NA | RR 3.0 (0.13-70.16) | NA – not significant effect | High |
| Makrides et al 2014 | Magnesium | PE | 3 | 1,042 | 0% | RR 0.87 (0.58-1.32) | NA – not significant effect | Moderate |
| Rumbold et al 2008 | Antioxidants (vitamin C and E, lycopene, red palm oil, selenium) | PE | 9 | 5446 | 56% | RR 0.73 (0.51-1.06) | NA – not significant effect | High |
| Salles et al 2012 | Antioxidants | PE | 15 | 21, 012 | 37%) | RR 0.92 (0.82-1.04) | NA – not significant effect | Moderate |
| Tentorio et al 2018 | Antioxidants (vitamins C and E, selenium,  L-arginine, allicin, lycopene and coenzyme Q10) | PE | 19 | 21,835 | 39% | RR 0.89 (0.79-1.02) | NA – not significant effect | Moderate |
| Meher and Duley 2006 | Garlic (allicin) | PE | 1 | 100 | NA | RR 0.78 (0.31-1.93) | NA – not significant effect | Moderate |
| Dorniak-Wall et al 2014 | L-arginine | PE | 1 | 450 | NA | RR 0.34 (0.21–0.55) | Strong | Critically low |
| Fu et al 2018 | Multi-vitamin supplementation | PE | 2 | 504 | 57% | RR 0.69 (0.51-0.93) | Moderate | Critically low |
| Allen et al 2014 | LC-PUFA (fish oil and gamma-linoleic acid) | PE | 6 | 4579 | 15% | RR 0.92 (0.71–1.18) | NA – not significant effect | Moderate |
| Chen et al 2015 | LC-PUFA (Marine oil omega 3) | PE | 12 | 5,685 | 5% | RR 0.93 (0.74–1.16) | NA – not significant effect | Low |
| Middleton et al 2018 | LC-PUFA (omega 3) | PE | 20 | 8,306 | 13% | RR 0.84  (0.69 -1.01) | NA – not significant effect | High |
| Ota et al 2015b | Balanced protein/energy supplementation | PE | 2 | 463 | NA | RR 1.48 (0.82-2.66) | NA – not significant effect | Moderate |

## Dietary intervention

| **Reference** | **Nutritional supplement or dietary intervention** | **Outcome** | **No. of RCT studies** | **No. participants** | **Variation between studies (I^2^)** | **Review pooled results (95% confidence intervals)** | **Strength of association (Harvard Cancer Index)** | **Quality assessment (AMSTAR2)** |
| --- | --- | --- | --- | --- | --- | --- | --- | --- |
| Duley et al 2005 | Low salt intake (vs normal intake) | PE | 2 | 603 | 0% | RR 1.11 (0.46-2.66) | NA – not significant effect | Moderate |
| Allen et al 2014 | Diet and nutritional counselling | PE | 6 | 2695 | 0% | RR 0.67 (0.53-0.85) | Moderate | Moderate |
| Syngelaki et al 2019 | Diet and nutritional counselling for obese or overweight women | PE | 11 | 5023 | 0% | RR 1.00 (0.78-1.27) | NA – not significant effect | Moderate |
| Thangaratinam et al 2012 | Diet and nutritional counselling | PE | 6 | 2624 | 0% | RR 0.67 (0.53-0.85) | Moderate | Moderate |

# Severe pre-eclampsia (Severe PE)

## Nutrient supplementation

| **Reference** | **Nutritional supplement or dietary intervention** | **Outcome** | **No. of RCT studies** | **No. participants** | **Variation between studies (I^2^)** | **Review pooled results (95% confidence intervals)** | **Strength of association (Harvard Cancer Index)** | **Quality assessment (AMSTAR2)** |
| --- | --- | --- | --- | --- | --- | --- | --- | --- |
| Basaran et al 2010 | Vitamin C and E | Severe PE | 6 | 16,342 | 0% | RR: 1.00 (0.84–1.18) | NA – not significant effect | Moderate |
| Conde-Agudelo et al 2011 | Vitamin C and E | Severe PE | 6 | 16,465 | 0% | RR 1.00 (0.84–1.18) | NA – not significant effect | Moderate |
| An et al 2015 | Calcium | Severe PE | 3 | 13,357 | 0% | RR 0.80 (0.60–1.05) | NA – not significant effect | Moderate |
| Hofmeyr et al 2007 | Calcium (high dose, ≥1g/day) | Severe PE | 1 | 8302 | NA | RR 0.74 (0.48-1.15) | Moderate | Moderate |
| Imdad and Bhutta 2011 | Calcium (developing countries) | Severe PE | 3 | 9072 | 0% | RR 0.70 (0.46-1.05) | NA – not significant effect | Low |
| Imdad and Bhutta 2012 | Calcium | Severe PE | 5 | 209 | 0% | RR 0.75 (0.57-0.98) | Weak | Moderate |
| An et al 2015 | Calcium | Severe PE | 3 | 13,357 | 0% | RR 0.80 (0.60–1.05) | NA – not significant effect | Moderate |
| Hofmeyr et al 2018 | Calcium (high dose, ≥1g/day) | Severe PE | 1 | 8302 | NA | 0.74 [0.48, 1.15] | NA – not significant effect | High |
| Rumbold et al 2008 | Antioxidants (vitamin C and E, lycopene, red palm oil, selenium) | Severe PE | 2 | 2495 | 0% | RR 1.25 (0.89-1.76) | NA – not significant effect | High |
| Salles et al 2012 | Antioxidants | Severe PE | 6 | 16, 341 | 0% | RR 1.03 (0.87-1.22) | NA – not significant effect | Moderate |

## Dietary intervention

None reported

# Gestational hypertension (GH)

## Nutrient supplementation

| **Reference** | **Nutritional supplement or dietary intervention** | **Outcome** | **No. of RCT studies** | **No. participants** | **Variation between studies (I^2^)** | **Review pooled results (95% confidence intervals)** | **Strength of association (Harvard Cancer Index)** | **Quality assessment (AMSTAR2)** |
| --- | --- | --- | --- | --- | --- | --- | --- | --- |
| Rahimi et al 2009 | Vitamin C and E (high risk women) | GH | 3 | 4,432 | NA | RR 1.3 (1.08-1.57) | Weak | Low |
| Basaran et al 2010 | Vitamin C and E | GH | 6 | 16,991 | 17% | RR: 1.11 (1.05–1.17) | Weak | Moderate |
| Conde-Agudelo et al 2011 | Vitamin C and E | GH | 7 | 19,003 | 0% | RR 1.11 (1.05–1.17) | Weak | Moderate |
| Roth et al 2017 | Vitamin D | GH | 2 | 564 | 0% | RR 1.69 (0.73-3.92) | NA – not significant effect | High |
| Gallo et al 2019 | Vitamin D | GH | 3 | 504 | 48% | OR 0.81 (0.30-2.15) | NA – not significant effect | Critically low |
| Palacios et al 2019 | Vitamin D | GH | 2 | 1130 | 0% | RR 0.78 (0.41- 1.49) | NA – not significant effect | High |
| Palacios et al 2019 | Vitamin D and calcium | GH | 1 | 59 | NA | RR 0.26 (0.06- 1.12) | NA – not significant effect | High |
| Carroli et al 1994 | Calcium | GH | 6 | 1,729 | NA | OR 0.44 (0.33-0.59) | Moderate | Critically low |
| Imdad and Bhutta 2011 | Calcium (developing countries) | GH | 6 | 9861 | 82% | RR 0.55 (0.36-0.85) | Moderate | Low |
| Imdad and Bhutta 2012 | Calcium | GH | 12 | 2732 | 74% | RR 0.65 (0.53-0.81) | Moderate | Moderate |
| An et al 2015 | Calcium | GH | 4 | 14,524 | 14% | RR 0.91  (0.84–0.99) | Not discernible | Moderate |
| Hofmeyr et al 2018 | Calcium (high dose, ≥1g/day) | GH | 12 | 15470 | 74% | RR 0.65 (0.53-0.81) | Moderate | High |
| Sun et al 2019 | Calcium | GH | 19 | 25529 | 65% | RR 0.70 (0.60-0.82) | Weak | Moderate |
| Rumbold et al 2008 | Antioxidants (vitamin C and E, lycopene, red palm oil, selenium) | GH | 7 | 5817 | 65% | RR 0.89  (0.62 -1.26) | NA – not significant effect | High |
| Meher and Duley 2006 | Garlic (allicin) | GH | 1 | 100 | NA | RR 0.50 (0.25- 1.00) | NA – not significant effect | Moderate |
| Newberry et al 2016 | LC-PUFA (Omega 3, low risk women) | GH | 3 | 2,875 | 0% | OR 0.94 (0.66-1.34) | NA – not significant effect | Moderate |
| Newberry et al 2016 | LC-PUFA (Marine oil omega 3, high risk women) | GH | 3 | 582 | 0% | OR 1.04 (0.76-1.42) | NA – not significant effect | Moderate |

## Dietary intervention

| **Reference** | **Nutritional supplement or dietary intervention** | **Outcome** | **No. of RCT studies** | **No. participants** | **Variation between studies (I^2^)** | **Review pooled results (95% confidence intervals)** | **Strength of association (Harvard Cancer Index)** | **Quality assessment (AMSTAR2)** |
| --- | --- | --- | --- | --- | --- | --- | --- | --- |
| Duley et al 2005 | Low salt intake (vs normal intake) | GH | 1 | 242 | NA | RR 0.98 (0.49- 1.94) | NA – not significant effect | Moderate |
| Thangaratinam et al 2012 | Diet and nutritional counselling | GH | 2 | 282 | 0% | RR 0.30 (0.10 -0.88) | Strong | Moderate |

# Eclampsia

## Nutrient supplementation

| **Reference** | **Nutritional supplement or dietary intervention** | **Outcome** | **No. of RCT studies** | **No. participants** | **Variation between studies (I^2^)** | **Review pooled results (95% confidence intervals)** | **Strength of association (Harvard Cancer Index)** | **Quality assessment (AMSTAR2)** |
| --- | --- | --- | --- | --- | --- | --- | --- | --- |
| Rumbold et al 2015a | Vitamin C (alone or with other supplements) | E | 9 | 20,304 | 0% | RR 1.42 (0.72-2.78]) | NA – not significant effect | Moderate |
| Rumbold et al 2015b | Vitamin E (alone or with other supplements) | E | 8 | 19,471 | 0% | RR 1.67 (0.82-3.41) | NA – not significant effect | Moderate |
| Conde-Agudelo et al 2011 | Vitamin C and E | E | 5 | 8,187 | 0% | RR 1.66 (0.77–3.57) | NA – not significant effect | Moderate |
| Hofmeyr et al 2007 | Calcium (high dose, ≥1g/day) | E | 2 | 12901 | NA | RR 0.73 (0.41-1.27) | NA – not significant effect | Moderate |
| Hofmeyr et al 2018 | Calcium (high dose, ≥1g/day) | E | 1 | 168 | NA | RR 0.17 (0.01-4.06) | NA – not significant effect | High |
| Makrides et al 2014 | Magnesium | E | 1 | 100 | NA | RR 0.14 (0.01-2.70) | NA – not significant effect | Moderate |
| Middleton et al 2018 | LC-PUFA (Omega 3) | E | 1 | 100 | NA | RR 0.14 (0.01-2.7) | NA – not significant effect | High |

## Dietary intervention

None reported

# HELLP syndrome

## Nutrient supplementation

| **Reference** | **Nutritional supplement or dietary intervention** | **Outcome** | **No. of RCT studies** | **No. participants** | **Variation between studies (I^2^)** | **Review pooled results (95% confidence intervals)** | **Strength of association (Harvard Cancer Index)** | **Quality assessment (AMSTAR2)** |
| --- | --- | --- | --- | --- | --- | --- | --- | --- |
| Conde-Agudelo et al 2011 | Vitamin C and E | HELLP | 5 | 8,187 | 36% | RR 1.09 (0.60–1.97) | NA – not significant effect | Moderate |
| Hofmeyr et al 2007 | Calcium (high dose, ≥1g/day) | HELLP | 2 | 12901 | NA | RR 2.67 (1.05-6.82) | Moderate | Moderate |
| Hofmeyr et al 2018 | Calcium (high dose, ≥1g/day) | HELLP | 2 | 12901 | 0% | 2.67 (1.05-6.82) | Moderate | High |

## Dietary intervention

None reported

# Pregnancy hypertension general (hypertensive diseases of pregnancy (HDP), pregnancy-induced hypertension (PIH), multiple pregnancy hypertension conditions)

## Nutrient supplementation

| **Reference** | **Nutritional supplement or dietary intervention** | **Outcome** | | **No. of RCT studies** | | **No. participants** | | **Variation between studies (I^2^)** | | **Review pooled results (95% confidence intervals)** | | **Strength of association (Harvard Cancer Index)** | **Quality assessment (AMSTAR2)** |
| --- | --- | --- | --- | --- | --- | --- | --- | --- | --- | --- | --- | --- | --- |
| Hofmeyr et al 2007 | Calcium (high dose, ≥1g/day) | High blood pressure (w or w/o proteinuria) | | 11 | | 14 946 | | NA | | RR 0.70 (0.57-0.86) | | Moderate | Moderate |
| Hofmeyr et al 2018 | Calcium (high dose, ≥1g/day) | High blood pressure (w or w/o proteinuria) | | 5 | | 665 | | 0% | | RR 0.53 (0.38-0.74) | | Moderate | High |
| Hua et al 2016 | Folic acid | GH/ PE | | 2 | | 3774 | | 0% | | RR 0.62 (0.45–0.87) | | Moderate | Moderate |
| Gui et al 2014 | L-arginine | PE/ E | | 2 | | 524 | | 9% | | OR 0.384 (0.25-0.58) | | Strong | Moderate |
| Ota et al 2015a | Zinc | Pregnancy hypertension/ PE | | 7 | | 2975 | | 26% | | RR 0.83 (0.64-1.08) | | NA – not significant effect | High |
| Szajewska, Borvath and Kolezko 2006 | LC-PUFA (Omega 3) | PE/ E | | 2 | | 328 | | NA | | RR 0.73 (0.22-2.37) | | NA – not significant effect | Moderate |
| Chen et al 2015 | LC-PUFA (Marine oil omega 3) | PIH | | 5 | | 4,130 | | 0% | | RR 1.03 (0.89–1.20) | | NA – not significant effect | Low |
| Gresham et al 2016 | Food and fortified food products | HDP | 2 | | 344 | | 0% | | SMD −0.29 (−0.86 to 0.29) | |  |  |  |

## Dietary intervention

| **Reference** | **Nutritional supplement or dietary intervention** | **Outcome** | **No. of RCT studies** | **No. participants** | **Variation between studies (I^2^)** | **Review pooled results (95% confidence intervals)** | **Strength of association (Harvard Cancer Index)** | **Quality assessment (AMSTAR2)** |
| --- | --- | --- | --- | --- | --- | --- | --- | --- |
| Gresham et al 2016 | Diet and nutritional counselling | HDP | 7 | 1,602 | 0% | SMD −0.12 (-0.30 to 0.06) | NA – not significant effect | Moderate |
| Syngelaki et al 2019 | Diet and nutritional counselling for obese or overweight women | HDP | 14 | 4345 | 53% | RR 0.84 (0.83-1.13) | NA – not significant effect | Moderate |

# Preterm birth (PTB)

## Nutrient supplementation

| **Reference** | | **Nutritional supplement or dietary intervention** | **Outcome** | **No. of RCT studies** | **No. participants** | **Variation between studies (I^2^)** | | | **Review pooled results (95% confidence intervals)** | **Strength of association (Harvard Cancer Index)** | **Quality assessment (AMSTAR2)** |
| --- | --- | --- | --- | --- | --- | --- | --- | --- | --- | --- | --- |
| Kongnyuy et al 2009 | | Vitamin A (HIV+ women) | PTB <37 weeks | 3 | 2,110 | 58% | | | RR 0.88 (0.54-1.19) | NA – not significant effect | Critically low |
| McCauley et al 2015 | | Vitamin A | PTB <37 weeks | 5 | 48,007 | 29% | | | RR 0.98 (0.94-1.01) | NA – not significant effect | Moderate |
| Thorne-Lyman and Fawzi 2012a | | Vitamin A or beta-carotene (HIV+ women) | PTB <37 weeks | 2 | NA | 76% | | | RR 0.85 (0.54-1.36) | NA – not significant effect | Low |
| Thorne-Lyman and Fawzi 2012a | | Vitamin A or beta-carotene (HIV- women) | PTB <37 weeks | 5 | NA | 0% | | | RR 1.01 (0.89-1.15) | NA – not significant effect | Low |
| Rumbold et al 2015a | | Vitamin C (alone or with other supplements) | PTB <37 weeks | 16 | 22,250 | 49% | | | RR 0.99 (0.90-1.10) | NA – not significant effect | Moderate |
| Rumbold et al 2015b | | Vitamin E (alone or with other supplements) | PTB <37 weeks | 11 | 20,565 | 52% | | | RR 0.98 (0.88-1.09) | NA – not significant effect | Moderate |
| Polyzos et al 2007 | | Vitamin C and E | PTB <37 weeks | 4 | 4,680 | 54% | | | RR 0.94 (0.74–1.19) | NA – not significant effect | Moderate |
| Rahimi et al 2009 | | Vitamin C and E (high risk women) | PTB <37 weeks | 5 | 5,232 | NA | | | RR 1.12 (0.96–1.32) | NA – not significant effect | Low |
| Basaran et al 2010 | | Vitamin C and E | PTB <37 weeks | 9 | 19,856 | 29% | | | RR: 1.01 (0.95–1.08) | NA – not significant effect | Moderate |
| Conde-Agudelo et al 2011 | | Vitamin C and E | PTB <37 weeks | 9 | 20,533 | 19% | | | RR 1.00 (0.94–1.06) | NA – not significant effect | Moderate |
| Dror and Allen 2012 | | Vitamins C and E | PTB <37 weeks | 9 | 19,632 | 51% | | | RR 1.00 (0.89–1.12) | NA – not significant effect | Critically low |
| Thorne-Lyman and Fawzi 2012b | | Vitamin D | PTB <37 weeks | 2 | 529 | 46% | | | RR 0.77 (0.35-1.66) | NA – not significant effect | Low |
| Perez-Lopez et al 2015 | | Vitamin D | PTB <37 weeks | 3 | 384 | 0% | | | RR 1.12 (0.60-2.63) | NA – not significant effect | Moderate |
| Roth et al 2017 | | Vitamin D | PTB <37 weeks | 13 | 3757 | 0% | | | RR 1.00 (0.77-1.30) | NA – not significant effect | High |
| Zhou et al 2017 | | Vitamin D | PTB <37 weeks | 6 | 1687 | 26% | | | RR 0.57 (0.36-0.91) | Moderate | Moderate |
| Bi et al 2018 | | Vitamin D | PTB | 11 | 3822 | 33% | | | RR 0.98 (0.77-1.26) | NA – not significant effect | Moderate |
| Palacios et al 2019 | | Vitamin D | PTB <37 weeks | 7 | 1640 | 45% | | | RR 0.66 (0.34- 1.30) | NA – not significant effect | High |
| Hofmeyr et al 2018 | | Vitamin D and calcium (low dose, <1g/day) | PTB | 3 | 760 | 0% | | | RR 1.59 (1.03-2.45) | Moderate | High |
| Palacios et al 2019 | | Vitamin D and calcium | PTB <37 weeks | 5 | 942 | 0% | | | RR 1.52 (1.01 -2.28) | Moderate | High |
| Carroli et al 1994 | | Calcium | PTB | 5 | 1,509 | NA | | | OR 0.66 (0.45-0.97) | Moderate | Critically low |
| Bucher et al 1996 | | Calcium | PTB | 5 | 1,568 | NA | | | OR 0.69 0.48-1.01) | NA – not significant effect | Critically low |
| Hofymeyr et al 2003 | | Calcium (high risk women) | PTB <37 weeks | 4 | 568 | NA | | | RR 0.42 (0.23-0.78) | Moderate | Critically low |
| Hofmeyr et al 2007 | | Calcium (high dose, ≥1g/day) | PTB <37 weeks | 10 | 14751 | NA | | | RR 0.81 (0.64-1.03) | NA – not significant effect | Moderate |
| Imdad and Bhutta 2011 | | Calcium (developing countries) | PTB | 5 | 967 | 36% | | | RR 0.88 (0.78-0.99) | Weak | Low |
| Imdad and Bhutta 2012 | | Calcium | PTB | 11 | 1517 | 60% | | | RR 0.76 (0.60-0.97) | Weak | Moderate |
| Hofmeyr et al 2013 | | Calcium (low dose, <1g/day) | PTB <37 weeks | 2 | 108 | NA | | | RR 0.41 (0.08–2.05) | NA – not significant effect | Moderate |
| An et al 2015 | | Calcium | PTB | 4 | 14,292 | 58% | | | RR 0.93 (0.76–1.13) | NA – not significant effect | Moderate |
| Buppasiri et al 2015 | | Calcium | PTB <37 weeks | 13 | 16139 | 57% | | | RR 0.86 (0.70- 1.05) | NA – not significant effect | High |
| Hofmeyr et al 2018 | | Calcium (high dose, ≥ 1 g/day) | PTB <37 weeks | 11 | 15,275 | 60% | | | RR 0.76 (0.60-0.97) | Weak | High |
| Hofmeyr et al 2018 | | Calcium (low dose, <1g/day) | PTB | 1 | 422 | NA | | | RR 0.40 (0.21-0.75) | Moderate | High |
| Park et al 2019 | | Calcium | PTB <37 weeks | 87 (network meta-analysis) | 205,867 | NA | | | OR 0.76 (0.56-0.98) | Weak | Moderate |
| Harding et al 2017 | | Iodine | PTB | 2 | 376 | 32% | | | RR 0.71 (0.30-1.66) | NA – not significant effect | Moderate |
| Cantor et al 2015 | Iron | | PTB | 2 | 1,010 | 0% | | | RR 0.88 (0.55-1.42) | NA – not significant effect | Low |
| Peña-Rosas et al 2015 | Iron | | PTB <37 weeks | 6 | 1713 | 0% | | | RR 0.82 (0.58-1.14) | NA – not significant effect | High |
| Park et al 2019 | Iron | | PTB <37 weeks | 87 (network meta-analysis) | 205,867 | NA | | | OR 0.55 (0.31-0.90) | Moderate | Moderate |
| Haider et al 2013 | Iron (with or without folic acid) | | PTB <37 weeks | 12 | 10,636 | 0% | | | RR 0.84 (0.68-1.03) | NA – not significant effect | Moderate |
| Peña-Rosas et al 2015 | Iron with or without folic acid) | | PTB <37 weeks | 13 | 19,286 | 0% | | | RR 0.93 (0.84-1.03) | NA – not significant effect | High |
| Peña-Rosas et al 2015 | Iron and folic acid | | PTB <37 weeks | 3 | 1497 | 34% | | | RR 1.55 (0.40-6.00) | NA – not significant effect | High |
| Park et al 2019 | Iron and folic acid | | PTB <37 weeks | 87 (network meta-analysis) | 205,867 | NA | | | OR 0.59 (0.30- 1.07) | NA – not significant effect | Moderate |
| De-Regil et al 2015 | Folic acid (pre/early pregnancy) | | PTB <37 weeks | 1 | 4862 | NA | | | RR 1.14 (0.93-1.41) | NA – not significant effect | High |
| Lassi et al 2013 | Folic acid | | PTB <37 weeks | 1 | 2797 | NA | | | RR 1.09 (0.77-1.54) | NA – not significant effect | High |
| Saccone and Berghella 2016 | Folic acid | | PTB <37 weeks | 1 | 1654 | NA | | | RR 0.99 (0.82-1.18) | NA – not significant effect | Moderate |
| Park et al 2019 | Folic acid | | PTB <37 weeks | 87 (network meta-analysis) | 205,867 | NA | | | OR 0.61 (0.30 -1.13) | Not significant | Moderate |
| Makrides et al 2014 | | Magnesium | PTB <37 weeks | 7 | 5,981 | 37% | | | RR 0.89 (0.69-1.14) | NA – not significant effect | Moderate |
| Chaffee and King 2012 | | Zinc | PTB | 16 | 7,819 | 26% | | | RR 0.86 (0.75-0.99) | Weak | Moderate |
| Ota et al 2015a | | Zinc | PTB <37 weeks | 16 | 7637 | 17% | | | RR 0.86  (0.76-0.97) | Weak | High |
| Soltani et al 2015 | | Zinc (adolescent pregnancies) | PTB <37 weeks | 2 | 1063 | NA | | | RR 0.66 (0.42-1.05) | NA – not significant effect | Low |
| Park et al 2019 | | Zinc | PTB <37 weeks | 87 (network meta-analysis) | 205,867 | NA | | | OR 0.53 (0.28- 0.93) | Moderate | Moderate |
| Rumbold et al 2008 | | Antioxidants (vitamin C and E, lycopene, red palm oil, selenium) | PTB <37 weeks | 5 | 5,198 | 0% | | | RR 1.10 (0.99-1.22) | NA – not significant effect | High |
| Salles et al 2012 | | Antioxidants | PTB <37 weeks | 14 | 21,166 | 44% | | | RR 1.03 (0.94-1.14) | NA – not significant effect | Moderate |
| Dorniak-Wall et al 2014 | | L-arginine | PTB <37 weeks | 1 | 672 | NA | | | RR 0.48 (0.28-0.81) | Moderate | Critically low |
| Fall et al 2009 | | Multiple micronutrient | PTB <37 weeks | 12 | 52,374 | | 1% | OR 1.00  (0.93-1.09) | | NA – not significant effect | Critically low |
| Shah et al 2009 | | Multiple micronutrient (vs iron-folic acid) | PTB <37 weeks | 9 | 45,192 | | 0% | RR 0.99 (0.96-1.03) | | NA – not significant effect | Moderate |
| Shah et al 2009 | | Multiple micronutrient (vs placebo) | PTB <37 weeks | 4 | 5191 | | 0% | RR 0.97 (0.82-1.13) | | NA – not significant effect | Moderate |
| Kawai et al 2011 | | Multiple micronutrient | PTB <37 weeks | 14 | NA | | 0% | RR 0.99 (0.95–1.03) | | NA – not significant effect | Critically low |
| Smith et al 2017 | | Multiple micronutrient (vs iron-folic acid alone in LMIC) | PTB <37 weeks | 16 | 112,953 | | 31% | RR 0.92 (0.88-0.95) | | Not discernible | Moderate |
| Keats et al 2019 | | Multiple micronutrient (vs iron or iron-folic acid) | PTB <37 weeks | 18 | 91,425 | | 49% | RR 0.95 (0.90-1.01) | | NA – not significant effect | High |
| Park et al 2019 | | Multiple micronutrient | PTB <37 weeks | 87 (network meta-analysis) | 205,867 | | NA | OR 0.54 (0.27- 0.97) | | Moderate | Moderate |
| Szajewska, Borvath and Kolezko 2006 | | LC-PUFA (Omega 3) | PTB <37 weeks | 3 | 861 | | NA | RR 0.67 (0.41-1.10) | | NA – not significant effect | Moderate |
| Salvig and Lamont 2011 | | LC-PUFA (Omega 3) | PTB <37 weeks | 4 | 921 | | NA | RR 0.61 (0.40-0.93) | | Moderate | Low |
| Kar et al 2015 | | LC-PUFA (Omega 3) | PTB <37 weeks | 9 | 5,980 | | 0% | RR 0.83 (0.70-0.98) | | Weak | Moderate |
| Saccone and Berghella 2015a | | LC-PUFA (Omega 3) | PTB <37 weeks | 9 | 3,493 | | 0% | RR 0.90 (0.72-1.11) | | NA – not significant effect | Moderate |
| Saccone and Berghella 2015b | | LC-PUFA (Omega 3, in women with previous preterm birth) | PTB <37 weeks | 2 | 1080 | | 53% | RR 0.81 (0.59-1.12 | | NA – not significant effect | Moderate |
| Chen et al 2016 | | LC-PUFA (Marine oils omega 3) | PTB <37 weeks | 14 | 6,980 | | 6% | RR 0.90 (0.81–1.00) | | NA – not significant effect | Low |
| Newberry et al 2016 | | LC-PUFA (Marine/ fish oil omega 3, low risk women) | PTB <37 weeks | 7 | NA | | 0% | OR 0.87 (0.66-1.15) | | NA – not significant effect | Moderate |
| Newberry et al 2016 | | LC-PUFA (Fish oil omega 3, high risk women) | PTB <37 weeks | 9 | NA | | 0% | OR 0.86 (0.65-1.15) | | NA – not significant effect | Moderate |
| Saccone et al 2016 | | LC-PUFA (Omega 3 in women without previous preterm birth) | PTB <37 weeks | 9 | 3854 | | 0% | RR 0.90 (0.72–1.11) | | NA – not significant effect | High |
| Middleton et al 2018 | | LC-PUFA (Omega 3) | PTB <37 weeks | 26 | 10,304 | | 8% | RR 0.89 (0.81-0.97) | | Weak | High |
| Ota et al 2015b | | Balanced protein/energy supplementation | PTB | 5 | 3384 | | 0% | RR 0.96 (0.80-1.16) | | NA – not significant effect | Moderate |
| Das et al 2018 | | Lipid-based supplementation (vs iron folic acid) | PTB <37 weeks | 3 | 4924 | | 0% | RR 0.94 (0.80-1.11) | | NA – not significant effect | High |
| Das et al 2018 | | Lipid-based supplementation (vs multiple micronutrients) | PTB <37 weeks | 3 | 2630 | | 0% | RR 1.15 (0.93-1.42) | | NA – not significant effect | High |
| Goto et al 2019 | | Lipid-based nutrient supplements (versus prenatal IFA, UNIMAP, other MMN, and CSB) | PTB <37 weeks | 5 | 7,458 | | 0% | RR 1.01 (0.88-1.16) | | NA – not significant effect | Moderate |
| Gresham et al 2016 | | Food and fortified food products | PTB | 7 | 2969 | | 0% | SMD −0.10 (−0.23 to 0.04) | | NA – not significant effect | Moderate |

## Dietary intervention

| **Reference** | **Nutritional supplement or dietary intervention** | **Outcome** | **No. of RCT studies** | **No. participants** | **Variation between studies (I^2^)** | **Review pooled results (95% confidence intervals)** | **Strength of association (Harvard Cancer Index)** | **Quality assessment (AMSTAR2)** |
| --- | --- | --- | --- | --- | --- | --- | --- | --- |
| Duley et al 2005 | Low salt intake (vs normal intake) | PTB <37 weeks | 1 | 242 | NA | RR 1.08 (0.46-2.56) | NA – not significant effect | Moderate |
| Jahanfar and Jaafar 2015 | Caffeinated (vs decaffeinated) | PTB <37 weeks | 1 | 1153 | NA | RR 0.81 (0.48-1.37) | NA – not significant effect | Moderate |
| Thangaratinam et al 2012 | Diet and nutritional counselling | PTB | 4 | 1474 | 35% | RR 0.68 (0.48- 0.96) | Moderate | Moderate |
| Ota et al 2015b | Nutritional counselling (to increase energy/ protein intake) | PTB | 2 | 449 | 0% | RR 0.46 (0.21- 0.98) | Moderate | Moderate |
| Gresham et al 2016 | Diet and nutritional counselling | PTB | 7 | 1759 | 19% | SMD −0.25 (−0.56 to 0.05) | NA – not significant effect | Moderate |
| Zhang et al 2018 | Diet and nutritional counselling | PTB | 4 | 995 | 30% | RR 0.70 (0.39-1.28) | NA – not significant effect | Low |

# Small for gestational age (SGA)

## Nutrient supplementation

| **Reference** | **Nutritional supplement or dietary intervention** | **Outcome** | **No. of RCT studies** | **No. participants** | **Variation between studies (I^2^)** | **Review pooled results (95% confidence intervals)** | **Strength of association (Harvard Cancer Index)** | **Quality assessment (AMSTAR2)** |
| --- | --- | --- | --- | --- | --- | --- | --- | --- |
| Thorne-Lyman and Fawzi 2012a | Vitamin A or beta-carotene | SGA | 2 | 1387 | 0% | RR 0.89 (0.68-1.17) | NA – not significant effect | Low |
| Rumbold et al 2015a | Vitamin C (alone or with other supplements) | SGA <10^th^ centile | 12 | 10,320 | 37% | RR 0.98 (0.91-1.06) | NA – not significant effect | Moderate |
| Rumbold et al 2015b | Vitamin E (alone or with other supplements) | SGA <10^th^ centile | 8 | 10,161 | 41% | RR 0.98 (0.90-1.06) | NA – not significant effect | Moderate |
| Polyzos et al 2007 | Vitamin C and E | SGA <10^th^ centile | 4 | 4,680 | 0% | RR 1.07 (0.96–1.20) | NA – not significant effect | Moderate |
| Rahimi et al 2009 | Vitamin C and E (high risk women) | SGA <10^th^ centile | 5 | 5,232 | NA | RR 1.04 (0.94–1.15) | NA – not significant effect | Low |
| Basaran et al 2010 | Vitamin C and E | SGA <10^th^ centile | 5 | 14,888 | 47% | RR: 0.99 (0.91–1.07) | NA – not significant effect | Moderate |
| Conde-Agudelo et al 2011 | Vitamin C and E | SGA | 9 | 20,533 | 27% | RR 0.99 (0.91–1.06) | NA – not significant effect | Moderate |
| Dror and Allen 2012 | Vitamins C and E | SGA <10^th^ centile | 8 | 10,164 | 35% | RR 0.94 (0.82-1.09) | NA – not significant effect | Critically low |
| Thorne-Lyman and Fawzi 2012b | Vitamin D | SGA | 2 | 305 | 0% | RR 0.67 (0.40- 1.11) | NA – not significant effect | Low |
| Perez-Lopez et al 2015 | Vitamin D | SGA | 3 | 456 | 15% | RR 0.78 (0.50-1.21) | NA – not significant effect | Moderate |
| Roth et al 2017 | Vitamin D | SGA <10^th^ centile | 5 | 741 | 0% | RR 0.60 (0.40-0.90) | Moderate | High |
| Bi et al 2018 | Vitamin D | SGA <10^th^ centile | 5 | 898 | 0% | RR 0.72 (0.52 -0.99) | Weak | Moderate |
| Maugeri et al 2019 | Vitamin D | SGA <10^th^ centile | 5 | 853 | 13% | RR 0.69 (0.51-0.92) | Moderate | Moderate |
| Carroli et al 1994 | Calcium | IUGR | 3 | 1,347 | NA | OR 0.78 (0.49-1.23) | NA – not significant effect | Critically low |
| Hofmeyr et al 2007 | Calcium (high dose, ≥ 1 g/day) | SGA | 3 | 13091 | NA | RR 1.10 (0.88-1.37) | NA – not significant effect | Moderate |
| Imdad and Bhutta 2012 | Calcium | SGA | 7 | 433 | 0% | RR 1.01 (0.84-1.21) | NA – not significant effect | Moderate |
| Hofmeyr et al 2013 | Calcium (low dose, <1g/day) | SGA | 3 | 194 | NA | RR 0.38 (0.10–1.38) | NA – not significant effect | Moderate |
| Buppasiri et al 2015 | Calcium | IUGR | 6 | 1,701 | 0% | RR 0.83 (0.61-1.13) | NA – not significant effect | High |
| Hofmeyr et al 2018 | Calcium (high dose, ≥ 1 g/day) | SGA | 4 | 13,615 | 0% | RR 1.05 (0.86-1.29) | NA – not significant effect | High |
| Harding et al 2017 | Iodine | SGA | 2 | 377 | 0% | RR 1.26 (0.77-2.05) | Not significant | Moderate |
| Haider et al 2013 | Iron (with or without folic acid) | SGA <10^th^ centile | 8 | 9,991 | 59% | RR 0.85 (0.67-1.08) | NA – not significant effect | Moderate |
| Makrides et al 2014 | Magnesium | SGA <10^th^ centile | 3 | 1,291 | 7% | RR 0.76 (0.54-1.07) | NA – not significant effect | Moderate |
| Chaffee and King 2012 | Zinc | SGA | 5 | 3,442 | 59% | RR 1.03 (0.91-1.17) | NA – not significant effect | Moderate |
| Ota et al 2015a | Zinc | SGA <10^th^ centile | 8 | 4252 | 28% | RR 1.02 (0.94-1.11) | NA – not significant effect | High |
| Rumbold et al 2008 | Antioxidants (vitamin C and E, lycopene, red palm oil, selenium) | SGA <10^th^ centile | 5 | 5271 | 68% | RR 0.83 (0.62-1.11) | NA – not significant effect | High |
| Salles et al 2012 | Antioxidants | SGA <10^th^ centile | 8 | 9,672 | 49% | RR 0.92 (0.80-1.05) | NA – not significant effect | Moderate |
| Tentorio et al 2018 | Antioxidants (vitamins C and E, selenium,  L-arginine, allicin, lycopene and coenzyme Q10) | IUGR | 2 | 251 | 0% | RR: 0.46 (0.26-0.81) | Moderate | Moderate |
| Fall et al 2009 | Multiple micronutrient | SGA <10th centile | 12 | 52,374 | 0% | OR 0.90  (0.82-0.99) | Not discernible | Critically low |
| Shah et al 2009 | Multiple micronutrient (vs placebo) | SGA <10th centile or > 2 SD below mean for GA) | 3 | 5140 | NA | RR 0.85 (0.71-1.02) | NA – not significant effect | Moderate |
| Shah et al 2009 | Multiple micronutrient (vs iron-folic acid) | SGA <10th entile or > 2 SD below mean for GA) | 5 | 13039 | NA | RR 0.89 (0.77-1.01) | NA – not significant effect | Moderate |
| Haider et al 2011 | Multiple micronutrient (vs iron-folic acid) | SGA <10th centile | 14 | 4,783 | 39% | RR 0.91 (0.86-0.96) | Not discernible | Moderate |
| Kawai et al 2011 | Multiple micronutrient (vs iron-folic acid) | SGA <10th centile | 15 | 64,244 | 47% | RR 0.85 (0.78–0.93) | Weak | Critically low |
| Smith et al 2017 | Multiple micronutrient (vs iron-folic acid alone in LMIC) | SGA <10th centile | 16 | 112,953 | 51% | RR 0.97 (0.96-0.99) | Not discernible | Moderate |
| Keats et al 2019 | Multiple micronutrient (vs iron or iron-folic acid) | SGA | 17 | 57,348 | 39% | RR 0.92 (0.88-0.97) | Not discernible | High |
| Kar et al 2015 | LC-PUFA (Omega 3) | SGA | 8 | 5,469 | 41% | RR 0.82 (0.66-1.03) | NA – not significant effect | Moderate |
| Saccone et al 2015 | LC-PUFA (Omega 3) | IUGR (in women with previous IUGR) | 3 | 575 | 0% | RR 1.13 (0.83-1.54) | NA – not significant effect | Moderate |
| Chen et al 2016 | LC-PUFA (Marine oils omega 3) | IUGR | 7 | 3,705 | 0% | RR 1.03 (0.91–1.08) | NA – not significant effect | Low |
| Newberry et al 2016 | LC-PUFA (Fish oil omega 3, high risk women) | SGA/IUGR <10^th^ centile | 4 | NA | 0% | OR 1.00 (0.70-1.43) | NA – not significant effect | Moderate |
| Saccone et al 2016 | LC-PUFA (Omega 3) | SGA <10^th^ centile | 3 | 558 | 0% | RR 0.86 (0.59–1.27) | NA – not significant effect | High |
| Middleton et al 2018 | LC-PUFA (Omega 3) | SGA/IUGR | 8 | 6,907 | 0% | RR 1.01  (0.9-1.13) | NA – not significant effect | High |
| Ota et al 2015b | Balanced protein/energy supplementation | SGA | 7 | 4408 | 16% | RR 0.79 (0.69-0.90) | Weak | Moderate |
| Ota et al 2015b | High protein supplementation | SGA | 1 | 505 | NA | RR 1.58 (1.03-2.41) | Moderate effect | Moderate |
| Say et al 2003 | Calf blood extract supplementation (vs placebo) | SGA <5^th^ centile | 1 | 31 | NA | RR 0.54 (0.20-1.47) | NA – not significant effect | Moderate |
| Say et al 2003 | Glucose supplementation (vs bed rest) | SGA <10^th^ centile | 1 | 30 | NA | RR 1.11 (0.64-1.92) | NA – not significant effect | Moderate |
| Say et al 2003 | Galactose supplementation (vs bed rest) | SGA <10^th^ centile | 1 | 30 | NA | RR 0.78 (0.39-1.54) | NA – not significant effect | Moderate |
| Das et al 2018 | Lipid-based supplementation (vs multiple micronutrients) | SGA | 3 | 2393 | 0% | RR 0.95 (0.84-1.07) | NA – not significant effect | High |
| Goto et al 2019 | Lipid-based nutrient supplements (versus prenatal IFA, UNIMAP, other MMN, and CSB) | SGA <10th centile | 5 | 7,151 | 0% | RR 0.94 (0.90-0.99) | Not discernible | Moderate |
| Gresham et al 2014 | Food and fortified food products | SGA | 1 | 1150 | NA | SMD 0.02 (-0.29 to 0.32) | NA – not significant effect | Moderate |

## Dietary intervention

| **Reference** | **Nutritional supplement or dietary intervention** | **Outcome** | **No. of RCT studies** | **No. participants** | **Variation between studies (I^2^)** | **Review pooled results (95% confidence intervals)** | **Strength of association (Harvard Cancer Index)** | **Quality assessment (AMSTAR2)** |
| --- | --- | --- | --- | --- | --- | --- | --- | --- |
| Duley et al 2005 | Low salt intake (vs normal intake) | SGA < 3^rd^ centile or lowest centile reported | 1 | 242 | NA | RR 1.50 (0.73-3.07) | NA – not significant effect | Moderate |
| Jahanfar and Jaafar 2015 | Caffeinated (vs decaffeinated) | SGA < 10^th^ centile | 1 | 1150 | NA | RR 0.97 (0.57-1.64) | NA – not significant effect | Moderate |
| Thangaratinam et al 2012 | Diet and nutritional counselling | SGA < 10th centile or 2500 g | 3 | 2252 | 0% | RR 1.02 (0.75-1.37) | NA – not significant effect | Moderate |
| Gresham et al 2014 | Nutritional counselling | SGA | 4 | 1225 | 0% | SMD 0.18 (-0.05 to 0.41) | NA – not significant effect | Moderate |
| Ota et al 2015b | Nutritional counselling (to increase energy/protein intake) | SGA | 1 | 404 | NA | RR 0.97 (0.45-2.11) | NA – not significant effect | Moderate |
| Zhang et al 2018 | Diet and nutritional counselling | SGA <10^th^ centile | 6 | 463 | 0% | RR 1.33 (0.71-2.50) | NA – not significant effect | Low |

# Low birth weight (LBW)

## Nutrient supplementation

| **Reference** | **Nutritional supplement or dietary intervention** | | **Outcome** | **No. of RCT studies** | **No. participants** | **Variation between studies (I^2^)** | | **Review pooled results (95% confidence intervals)** | | **Strength of association (Harvard Cancer Index)** | **Quality assessment (AMSTAR2)** |
| --- | --- | --- | --- | --- | --- | --- | --- | --- | --- | --- | --- |
| Kongnyuy et al 2009 | Vitamin A (HIV+ women) | | LBW <2500g | 3 | 2,606 | 0% | | RR 0.83 (0.68–1.01) | | NA – not significant effect | Critically low |
| McCauley et al 2015 | Vitamin A | | LBW <2500g | 4 | 14,599 | 10% | | RR 1.02 (0.89-1.16) | | NA – not significant effect | Moderate |
| Thorne-Lyman and Fawzi 2012a | Vitamin A or beta-carotene (HIV+ women) | | LBW <2500g | 3 | 2053 | 0% | | RR 0.79 (0.64-0.99) | | NA – not significant effect | Low |
| Thorne-Lyman and Fawzi 2012a | Vitamin A or beta-carotene (HIV- women) | | LBW <2500g | 2 | 201 | 20% | | RR 0.83 (0.41-1.29) | | Weak | Low |
| Rahimi et al 2009 | Vitamin C and E (high risk women) | | LBW <2500g | 3 | 3,582 | NA | | RR 1.13 (1.004 −1.27) | | Weak | Low |
| Basaran et al 2010 | Vitamin C and E | | LBW <2500g | 5 | 8,666 | 15% | | RR: 1.00 (0.89–1.12) | | NA – not significant effect | Moderate |
| Conde-Agudelo et al 2011 | Vitamin C and E | | LBW | 6 | 15,837 | 41% | | RR 0.99 (0.92–1.07) | | NA – not significant effect | Moderate |
| Dror and Allen 2012 | Vitamins C and E | | LBW <2500g | 3 | 14,878 | 52% | | RR 0.99 (0.83-1.19) | | NA – not significant effect | Critically low |
| Thorne-Lyman and Fawzi 2012b | Vitamin D | | LBW <2500g | 3 | 507 | 5% | | RR 0.40 (0.23-0.71) | | Moderate | Low |
| Perez-Lopez et al 2015 | Vitamin D | | LBW <2500g | 4 | 496 | 0% | | RR 0.72 (0.44-1.16) | | NA – not significant effect | Moderate |
| Roth et al 2017 | Vitamin D | | LBW <2500g | 7 | 1156 | 47% | | RR 0.74 (0.47-1.16) | | NA – not significant effect | High |
| Bi et al 2018 | Vitamin D | | LBW | 4 | 775 | 65% | | RR 0.52 (0.20 -1.37) | | NA – not significant effect | Moderate |
| Maugeri et al 2019 | Vitamin D | | LBW <2500g | 3 | 513 | 0% | | RR 0.40 (0.22-0.74) | | Moderate | Moderate |
| Palacios et al 2019 | Vitamin D | | LBW <2500g | 5 | 679 | 36% | | RR 0.55 (0.35-0.87) | | Moderate | High |
| Palacios et al 2019 | Vitamin D and calcium | | LBW <2500g | 2 | 110 | 6% | | RR 0.68 (0.10-4.55) | | NA – not significant effect | High |
| Hofymeyr et al 2003 | Calcium | | LBW <2500g | 7 | 6491 | NA | | RR 0.83 (0.71-0.98) | | Weak | Critically low |
| Hofmeyr et al 2007 | Calcium (at least 1 g/day) | | LBW <2500g | 8 | 14359 | NA | | RR 0.84 (0.68-1.03) | | NA – not significant effect | Moderate |
| Imdad and Bhutta 2011 | Calcium (developing countries) | | LBW <2500g | 3 | 9498 | 61% | | RR 0.81 (0.58-1.12) | | NA – not significant effect | Low |
| Imdad and Bhutta 2012 | Calcium | | LBW <2500g | 6 | 1688 | 50% | | RR 0.85 (0.72-1.0)] | | NA – not significant effect | Moderate |
| Hofmeyr et al 2013 | Calcium (low dose, <1g/day) | | LBW <2500g | 2 | 134 | NA | | RR 0.20 (0.05–0.88) | | Strong | Moderate |
| An et al 2015 | Calcium | | LBW | 3 | 13,125 | 64% | | RR 0.91 (0.72–1.16) | | NA – not significant effect | Moderate |
| Buppasiri et al 2015 | Calcium | | LBW <2500g | 6 | 14162 | 62% | | RR 0.93 (0.81-1.07) | | NA – not significant effect | High |
| Hofmeyr et al 2018 | Calcium (high dose, ≥ 1 g/day) | | LBW <2500g | 9 | 14,883 | 50% | | RR 0.85 (0.72-1.01) | | Weak | High |
| Hofmeyr et al 2019 | Calcium (pre/early pregnancy) | | LBW <2500g | 1 | 507 | NA | | RR 1.00 (0.76 to 1.30) | | NA – not significant effect | High |
| Harding et al 2017 | Iodine | | LBW <2500g | 2 | 377 | 0% | | RR 0.56 (0.26-1.23) | | Not significant | Moderate |
| Cantor et al 2015 | Iron | | LBW | 4 | 688 | 0% | | RR 1.10 (0.54-2.25) | | NA – not significant effect | Low |
| Peña-Rosas et al 2015 | Iron | | LBW <2500g | 6 | 1136 | 45% | | RR 0.63 (0.30-1.32) | | NA – not significant effect | High |
| Haider et al 2013 | Iron (with or without folic acid) | | LBW <2500g | 13 | 9,860 | 1% | | RR 0.81 (0.71-0.93) | | Weak | Moderate |
| Peña-Rosas et al 2015 | Iron (with or without folic acid) | | LBW <2500g | 11 | 17,613 | 33% | | RR 0.84 (0.69-  1.03) | | NA – not significant effect | High |
| Peña-Rosas et al 2015 | Iron and folic acid | | LBW <2500g | 2 | 1311 | 29% | | RR 1.07 (0.31-3.74) | | NA – not significant effect | High |
| De-Regil et al 2015 | Folic acid (pre/early pregnancy) | | LBW <2500g | 2 | 5048 | 7% | | RR 1.13 (0.84-1.52) | | NA – not significant effect | High |
| Lassi et al 2013 | Folic acid | | LBW <2500g | 3 | 3089 | 0% | | RR 0.80 (0.63-1.02) | | NA – not significant effect | High |
| Saccone and Berghella 2016 | Folic acid | | LBW | 5 | 5,332 | NA | | RR 0.79 (0.49-1.28) | | NA – not significant effect | Moderate |
| Makrides et al 2014 | Magnesium | | LBW <2500g | 5 | 5,577 | 22% | | RR 0.95 (0.83-1.09) | | NA – not significant effect | Moderate |
| Chaffee and King 2012 | Zinc | | LBW <2500g | 11 | 5,614 | 38% | | RR 1.06 (0.91-1.23) | | NA – not significant effect | Moderate |
| Ota et al 2015a | Zinc | | LBW <2500g | 14 | 5643 | 38% | | RR 0.93  (0.78-1.12) | | NA – not significant effect | High |
| Soltani et al 2015 | Zinc (adolescent pregnancies) | | LBW <2500g | 1 | 507 | NA | | RR 0.39 (0.15-0.89) | | Strong | Low |
| Fall et al 2009 | Multiple micronutrient supplementation | LBW <2500g | | 12 | 52,374 | | 0% | | OR 0.89 (0.81-0.97) | Weak | Critically low |
| Shah et al 2009 | Multiple micronutrient (vs placebo) | LBW <2500g | | 4 | 6097 | | 55-64% | | RR 0.81 (0.73-0.91) | Weak | Moderate |
| Shah et al 2009 | Multiple micronutrient (vs iron-folic acid) | LBW <2500g | | 10 | 29,889 | | 55-64% | | RR 0.83 (0.74-0.93) | Weak | Moderate |
| Kawai et al 2011 | Multiple micronutrient (vs iron-folic acid) | LBW <2500g | | 15 | 64,244 | | 41% | | RR 0.86 (0.79–0.93) | Weak | Critically low |
| Smith et al 2017 | Multiple micronutrient (vs iron-folic acid alone in LMIC) | LBW <2500g | | 16 | 112,953 | | 42% | | RR 0.88 (0.85-0.90) | Weak | Moderate |
| Keats et al 2019 | Multiple micronutrient (vs iron or iron-folic acid) | LBW <2500g | | 18 | 68,801 | | 0% | | RR 0.88 (0.85-0.91) | Weak | High |
| Szajewska, Borvath and Kolezko 2006 | LC-PUFA (Omega 3) | LBW <2500g | | 2 | 328 | | NA | | RR 0.66 (0.34-1.26) | NA – not significant effect | Moderate |
| Chen et al 2016 | LC-PUFA (Marine oils omega 3) | LBW <2500g | | 7 | 4,901 | | 0% | | RR 0.77 (0.65–0.92) | Weak | Low |
| Newberry et al 2016 | LC-PUFA (Marine/fish oil omega 3, low risk women) | LBW <2500g | | 4 | NA | | 7% | | OR 0.72  (0.43 -1.11) | NA – not significant effect | Moderate |
| Middleton et al 2018 | LC-PUFA (Omega 3) | LBW <2500g | | 15 | 8,449 | | 29% | | RR 0.90 (0.82-0.99) | Not discernible | High |
| Das et al 2018 | Lipid-based supplementation (vs multiple micronutrients) | LBW <2500g | | 3 | 2404 | | 0% | | RR 0.92 (0.74-1.14) | NA – not significant effect | High |
| Das et al 2018 | Lipid-based supplementation (vs iron folic acid) | LBW <2500g | | 3 | 4826 | | 33% | | RR 0.87 (0.72-1.05) | NA – not significant effect | High |
| Goto et al 2019 | Lipid-based nutrient supplements (versus prenatal IFA, UNIMAP, other MMN, and CSB) | LBW <2500g | | 5 | 7,163 | | 0% | | RR 0.90 (0.83-0.98) | Not discernible | Moderate |
| Gresham et al 2014 | Food and fortified food products | LBW | | 8 | 4530 | | 24% | | SMD -0.22 (-0.37 to -0.06) | Significant – but not RR/OR | Moderate |

## Dietary intervention

| **Reference** | **Nutritional supplement or dietary intervention** | **Outcome** | **No. of RCT studies** | **No. participants** | **Variation between studies (I^2^)** | **Review pooled results (95% confidence intervals)** | **Strength of association (Harvard Cancer Index)** | **Quality assessment (AMSTAR2)** |
| --- | --- | --- | --- | --- | --- | --- | --- | --- |
| Gresham et al 2014 | Nutritional counselling | LBW | 2 | 908 | 0% | SMD -0.02 (-0.33 to 0.28) | NA – not significant effect | Moderate |
| Ota et al 2015b | Nutritional counselling (to increase energy/protein intake) | LBW | 1 | 399 | NA | RR 0.04 (0.01-0.14) | Very strong | Moderate |

# Stillbirth

## Nutrient supplementation

| **Reference** | **Nutritional supplement or dietary intervention** | **Outcome** | **No. of RCT studies** | **No. participants** | **Variation between studies (I^2^)** | **Review pooled results (95% confidence intervals)** | **Strength of association (Harvard Cancer Index)** | **Quality assessment (AMSTAR2)** |
| --- | --- | --- | --- | --- | --- | --- | --- | --- |
| Kongnyuy et al 2009 | Vitamin A (HIV+ women) | Stillbirth | 4 | 2,855 | 0% | RR 0.99 (0.68-1.43) | NA – not significant effect | Critically low |
| McCauley et al 2015 | Vitamin A | Stillbirth | 2 | 122,850 | 0% | RR 1.04 (0.98-1.1) | NA – not significant effect | Moderate |
| Rumbold et al 2015 (vit C) | Vitamin C (alone or with other supplements) | Stillbirth | 11 | 20,038 | 0% | RR 1.15 (0.89-1.49) | NA – not significant effect | Moderate |
| Rumbold et al 2015 (vit E) | Vitamin E (alone or with other supplements) | Stillbirth | 9 | 19,023 | 0% | RR 1.17 (0.88-1.56) | NA – not significant effect | Moderate |
| Basaran et al 2010 | Vitamin C and E | Stillbirth | 5 | 8,666 | 15% | RR: 1.44 (0.95–2.19) | NA – not significant effect | Moderate |
| Conde-Agudelo et al 2011 | Vitamin C and E | Stillbirth | 6 | 18,635 | 10% | RR 1.27 (0.93–1.72) | NA – not significant effect | Moderate |
| Dror and Allen 2012 | Vitamins C and E | Stillbirth | 7 | 8,912 | 0% | RR 1.23 (0.85-1.94) | NA – not significant effect | Critically low |
| Roth et al 2017 | Vitamin D | Stillbirth | 16 | 4606 | 0% | RR 0.75 (0.51-1.13) | NA – not significant effect | High |
| Palacios et al 2019 | Vitamin D | Stillbirth | 3 | 584 | 0% | RR 0.35 (0.06-1.98) | NA – not significant effect | High |
| Jabeen et al 2011 | Calcium | Stillbirth | 3 | 10027 | 0% | RR 0.81 (0.63-1.03) | NA – not significant effect | Low |
| De-Regil et al 2015 | Folic acid (early/ pre pregnancy) | Stillbirth | 4 | 6597 | 0% | RR 1.05 (0.54-2.05) | NA – not significant effect | High |
| Makrides et al 2014 | Magnesium | Stillbirth | 4 | 5,526 | 0% | RR 0.73 (0.43-1.25) | NA – not significant effect | Moderate |
| Ronsmans et al 2009 / Margetts et al 2009 / Shrimpton et al 2009 | Multiple micronutrient (vs iron-folic acid) | Stillbirth | 12 | 46,763 | 3% | OR 1.01 (0.88-1.16) | NA – not significant effect | Critically low |
| Smith et al 2017 | Multiple micronutrient (vs iron-folic acid alone in LMIC) | Stillbirth | 16 | 112,953 | 42% | RR 0.92 (0.86-0.99) | Not discernible | Moderate |
| Keats et al 2019 | Multiple micronutrient (vs iron or iron-folic acid) | Stillbirths | 17 | 97,927 | 12% | RR 0.95 (0.86-1.04) | NA – not significant effect | High |
| Chen et al 2016 | LC-PUFA (Marine oils omega 3) | Stillbirth | 9 | 7,669 | 0% | RR 0.73 (0.49–1.08) | NA – not significant effect | Low |
| Middleton et al 2018 | LC-PUFA (Omega 3) | Stillbirth | 16 | 7,880 | 0% | RR 0.94  (0.62-1.42) | NA – not significant effect | High |
| Ota et al 2015b | Balanced protein/energy supplementation | Stillbirth | 5 | 3408 | 10% | RR 0.60 (0.39-0.94) | Moderate | Moderate |
| Ota et al 2015b | High protein supplementation | Stillbirth | 1 | 529 | NA | RR 0.81 (0.31-2.15) | NA – not significant effect | Moderate |
| Das et al 2018 | Lipid-based supplementation (vs iron folic acid) | Stillbirth | 3 | 5575 | 63% | RR 1.14 (0.52-2.48) | NA – not significant effect | High |
| Goto et al 2019 | Lipid-based nutrient supplements (versus prenatal IFA, UNIMAP, other MMN, and CSB) | Stillbirth | 5 | 9,656 | 65% | RR 1.06 (0.60-1.88) | NA – not significant effect | Moderate |

## Dietary intervention

| **Reference** | **Nutritional supplement or dietary intervention** | **Outcome** | **No. of RCT studies** | **No. participants** | **Variation between studies (I^2^)** | **Review pooled results (95% confidence intervals)** | **Strength of association (Harvard Cancer Index)** | **Quality assessment (AMSTAR2)** |
| --- | --- | --- | --- | --- | --- | --- | --- | --- |
| Ota et al 2015b | Nutritional counselling (to increase energy/protein intake) | Stillbirth | 1 | 431 | NA | RR 0.37 (0.07-1.90) | NA – not significant effect | Moderate |

# Maternal mortality

## Nutrient supplementation

| **Reference** | **Nutritional supplement or dietary intervention** | **Outcome** | **No. of RCT studies** | **No. participants** | **Variation between studies (I^2^)** | **Review pooled results (95% confidence intervals)** | **Strength of association (Harvard Cancer Index)** | **Quality assessment (AMSTAR2)** |
| --- | --- | --- | --- | --- | --- | --- | --- | --- |
| McCauley et al 2015 | Vitamin A | Maternal death | 4 | 154,039 | 50% | RR 0.88 (0.65-1.20) | NA – not significant effect | Moderate |
| Thorne-Lyman and Fawzi 2012a | Vitamin A or beta-carotene | Maternal death | 3 | NA | 74% | RR 0.86 (0.60-1.24) | NA – not significant effect | Low |
| Rumbold et al 2015 (vit C) | Vitamin C (alone or with other supplements) | Maternal death | 7 | 17,120 | 0% | RR 0.60 (0.14-2.51) | NA – not significant effect | Moderate |
| Rumbold et al 2015 (vit E) | Vitamin E (alone or with other supplements) | Maternal death | 7 | 17,120 | 0% | RR 0.60 (0.14-2.51) | NA – not significant effect | Moderate |
| Basaran et al 2010 | Vitamin C and E | Maternal death | 7 | 19,427 | NA | RR: 0.72 (0.18–2.84) | NA – not significant effect | Moderate |
| Conde-Agudelo et al 2011 | Vitamin C and E | Maternal death | 6 | 17,550 | 0% | RR 0.60 (0.14–2.51) | NA – not significant effect | Moderate |
| Hofmeyr et al 2007 | Calcium (high dose, ≥ 1 g/day) | Maternal death | 1 | 8312 | NA | RR 0.17 (0.02-1.39) | NA – not significant effect | Moderate |
| Buppasiri et al 2015 | Calcium | Maternal death | 2 | 8974 | 4% | RR 0.29 (0.06-1.38) | NA – not significant effect | High |
| Hofmeyr et al 2018 | Calcium (high dose, ≥ 1 g/day) | Maternal death | 1 | 8,312 | NA | RR 0.84 (0.66-1.07) | NA – not significant effect | High |
| Peña-Rosas et al 2015 | Iron (with or without folic acid) | Maternal death | 2 | 12,560 | NA | RR 0.33 (0.01-8.19) | NA – not significant effect | High |
| Rumbold et al 2008 | Antioxidants (vitamin C and E, lycopene, red palm oil, selenium) | Maternal death | 2 | 4272 | NA | RR 1.00 (0.06-16.01) | NA – not significant effect | High |
| Middleton et al 2018 | LC-PUFA (Omega 3) | Maternal death | 4 | 4,830 | NA | RR 1.69 (0.07-39.3) | NA – not significant effect | High |
| Das et al 2018 | Lipid-based supplementation (vs iron folic acid) | Maternal death | 3 | 5628 | 0% | RR 0.53 (0.12-2.41) | NA – not significant effect | High |

## Dietary intervention

None reported
