# Supplementary material for: Effects of Maternal Nutritional Supplements and Dietary Interventions on Placental Complications: An Umbrella Review, Meta-Analysis and Evidence Map
Source: Nutrients. 2021 Jan 30;13(2):472. doi: 10.3390/nu13020472 (PMC7912620; doi:10.3390/nu13020472)
Supplement: Supplementary file 1 [file nutrients-13-00472-s001.zip › Supplementary files/Table S8 - Study characteristics of individual trials.docx]

**Table S8 – Study characteristics of individual trials included in reviews**

Contents

[Vitamin A 2](#_Toc55376958)

[Vitamin C and/or E 5](#_Toc55376959)

[Vitamin C 5](#_Toc55376960)

[Vitamin C and E 7](#_Toc55376961)

[Vitamin E 10](#_Toc55376962)

[Vitamin D and/or Calcium 11](#_Toc55376963)

[Vitamin D 11](#_Toc55376964)

[Vitamin D and calcium 17](#_Toc55376965)

[Calcium 20](#_Toc55376966)

[Calcium and linoleic acid (polyunsaturated omega-6 fatty acid) 25](#_Toc55376967)

[Iron and/or folic acid 26](#_Toc55376968)

[Iron 26](#_Toc55376969)

[Folic acid 29](#_Toc55376970)

[Iron-folic acid 32](#_Toc55376971)

[Zinc 37](#_Toc55376972)

[Multiple micronutrient (MMN) 43](#_Toc55376973)

[MMN 43](#_Toc55376974)

[Lipid-based nutrient supplement (LNS) 55](#_Toc55376975)

[Polyunsaturated omega-3 fatty acid 58](#_Toc55376976)

[Omega 3 58](#_Toc55376977)

[Omega 3 and omega 6 66](#_Toc55376978)

[Omega 3 and vitamin E 67](#_Toc55376979)

[Diet and nutritional counselling 67](#_Toc55376980)

[Antenatal dietary counselling 67](#_Toc55376981)

[Antenatal diet and physical activity counselling 70](#_Toc55376982)

# Vitamin A

| **Reference** | **Study design** | **Country** | **Participants** | **Intervention** | **Comparison** | **Co-interventions** | **Risk of bias** |
| --- | --- | --- | --- | --- | --- | --- | --- |
| Coutsoudis et al 1999 | Double-blind, placebo-controlled, parallel group RCT | South Africa | 728 HIV positive Black pregnant women at 28-32 weeks gestation | 5,000 IU retinyl palmitate and 30 mg beta-carotene daily during 3^rd^ trimester and 200,000 IU retinyl palmitate at delivery (n=368) | Placebo (n=360) | None reported | Unclear risk |
| Cox et al 2005 | Double-blind, placebo-controlled, parallel group RCT | Ghana | 98 healthy HIV negative primigravid pregnant women <24 weeks gestation | 10,000 IU retinyl palmitate in groundnut oil plus tocopherol as preservative weekly until 6 weeks postpartum (n=48) | Placebo (groundnut oil and tocopherol) until 6 weeks postpartum (n=50) | Iron and folic acid and antimalarial prophylaxis | Unclear risk |
| Dijkhuizen et al 2004 | Double-blind, placebo-controlled parallel group RCT, 2x2 factorial design | Indonesia | 170 pregnant women <20 weeks gestation, singleton pregnancy | Group 1: 4.5 mg beta-carotene (n=43); Group 2: 30 mg zinc sulphate (n=44); Group 3: 4.5 mg beta carotene and 30 mg zinc sulphate (n=42) all daily until delivery  **comparison in current review between beta-carotene (group 1) and placebo* | Placebo until delivery (n=41) | Iron and folic acid | Unclear risk |
| Fawzi et al 1998/ Merchant et al 2005 | Double-blind, placebo-controlled parallel group RCT, 2x2 factorial design | Tanzania | 1,075 HIV positive pregnant women between 12-27 weeks gestation | Group 1: 30 mg beta-carotene plus 5,000 IU preformed vitamin A daily, 200,000 IU vitamin A at delivery (n=269); Group 2: Daily multivitamin without vitamin A, with 20 mg B1, 20 mg B2, 25 mg B6, 100 mg niacin, 50 mcg B12, 500 mg C, 30 mg E, and 0.8 mg folic acid (n=269), Group 3: multivitamins including vitamin A, 200,000 IU vitamin A at delivery (n=270)  **comparison in current review between beta-carotene (group 1) and placebo* | Placebo (n=267) | 400 mg iron and 5mg folate daily and 500 mg prophylactic chloroquine phosphate per week | Unclear risk |
| Kirkwood et al 2010 | Cluster double-blind, placebo-controlled parallel group RCT | Ghana | Women aged 15-45 years in 1,086 study clusters | 25,000 IU vitamin A weekly (n= 104,484) | Placebo (soybean oil) (n= 103,297) | None reported | Unclear risk |
| Kumwenda et al 2002 | Double-blind, placebo-controlled parallel group RCT | Malawi | 697 HIV positive pregnant women at 18-28 weeks gestation | 10,000 IU of vitamin A daily until delivery (n=340) | Placebo until delivery (n=357) | 30 mg iron and 400 mcg folate daily until delivery; all women received vitamin A at 6 weeks' postpartum | Unclear risk |
| Radhika et al 2003 | Double-blind, placebo-controlled parallel group RCT | India | 170 healthy pregnant women at 16-24 weeks gestation, normotensive at entry and without diabetes, recurrent pregnancy loss or prior preterm delivery | 2,173–2,307 mcg beta-carotene (~3,621–3,845 IU) red palm oil daily from 26-28 weeks gestation for 8 weeks (n=85) | Placebo (groundnut oil) (n=85) | Iron-folate for 100 days | Unclear risk |
| Van den Broek et al 2006 | Double-blind, placebo-controlled parallel group RCT, three arms | Malawi | 700 pregnant rural women, anaemic (<11.0g/dl), singleton pregnancies at 12-24 weeks gestation | Group 1: 5,000 IU vitamin A (n=234); Group 2: 10,000 IU vitamin A (n=234), daily until delivery **combined in current review* | Placebo until delivery (n=232) | Daily 60 mg iron and 250mcg folic acid and anti-malarial prophylaxis at 20 and 34 weeks gestation | Unclear risk |
| West et al 1999 | Cluster, double-blind, placebo-controlled parallel group RCT, three arms | Nepal | Married women of reproductive age in 270 rural study clusters | Group 1: 7,000 mcg retinyl palmitate (23300 IU) (n=15,305, 90 clusters, of which 7,045 pregnant); Group 2: 42 mg all trans beta-carotene (n=14,536, 90 clusters, of which 6494 pregnant), weekly until 12 weeks postpartum  **combined in current review* | Placebo (n=14,805, 90 clusters, 6,580 pregnant), until 12 weeks postpartum | All capsules contained about 5 mg dl  alpha­tocopherol as preservative | Unclear risk |
| West et al 2011/ Christian et al 2013 (secondary analysis for SGA, PTB and LBW) | Cluster, double-blind, placebo-controlled parallel group RCT, three arms | Bangladesh | Rural pregnant women in their first trimester between 13-45 years old in 594 community clusters | Group 1: 7,000 mcg retinyl palmitate (23,300 IU) (n= 20012, 198 clusters); Group 2: 42 mg all trans beta-carotene (n=20,222, 198 clusters), weekly until 12 weeks postpartum  **combined in current review* | Placebo (n= 20,060, 198 cluster), until 12 weeks postpartum | All capsules also contained about 5 mg dl  alpha­tocopherol as preservative | Low risk |

# Vitamin C and/or E

## Vitamin C

| **Reference** | **Study design** | **Country** | **Participants** | **Intervention** | **Comparison** | **Co-interventions** | **Risk of bias** |
| --- | --- | --- | --- | --- | --- | --- | --- |
| Casanueva et al 2005 | Double-blind, placebo-controlled parallel group RCT | Mexico | 120 healthy pregnant women with no acute or chronic disease, singleton pregnancy, no vitamin supplements, before 20 weeks gestation | 100mg vitamin C daily (n=58) | Placebo (n=62) | None reported | Unclear risk |
| Kiondo et al 2014 | Double-blind, placebo-controlled parallel group RCT | Uganda | 932 healthy pregnant women age 15-42 years old, at 12-22 weeks gestation | 1,000mg ascorbic acid daily until delivery (n=466) | Placebo (micro-crystalline cellulose) until delivery (n=466) | None reported | Low risk |
| McEvoy et al 2014 | Multicentre, double-blind, placebo-controlled parallel group RCT | USA | 179 pregnant women, current smoker at least 1 cigarette a day, singleton pregnancy, 22 weeks gestation or earlier who passed placebo adherence test, 15 years or older | 500mg ascorbic acid daily until delivery (n=89) | Placebo (cornstarch) until delivery (n=90) | Standard prenatal vitamin containing 60mg of vitamin C | Unclear risk |
| Steyn et al 2003 | Double-blind, placebo-controlled parallel group RCT | South Africa | 203 pregnant women with history of previous mid-trimester abortion or previous preterm labour, singleton pregnancies before 26 weeks gestation | 250mg ascorbic acid daily until 34 weeks gestation (n=100) | Placebo until 34 weeks gestation (n=100) | None reported | Unclear risk |

## Vitamin C and E

| **Reference** | **Study design** | **Country** | **Participants** | **Intervention** | **Comparison** | **Co-interventions** | **Risk of bias** |
| --- | --- | --- | --- | --- | --- | --- | --- |
| Abramovici 2015 (secondary analysis of Roberts et al 2010 for smokers) | Multicentre, double-blind, placebo-controlled parallel group RCT | USA | 1,551 pregnant women currently smoking or quit for pregnancy, nulliparous, singleton pregnancy, normotensive on entry, at less than 16 weeks gestation | 1,000 mg ascorbic acid and 400 IU RRR alpha-tocopherol acetate daily until delivery (n=788) | Placebo (mineral oil) until delivery (n= 763) | None reported | Low risk |
| Beazley et al 2005 | Double-blind, placebo-controlled parallel group RCT | USA | 109 pregnant women at risk for pre-eclampsia, at 14-20 weeks gestation | 1,000mg vitamin C and 400 IU vitamin E daily (n=54) | Placebo (n=55) | Regular prenatal vitamins (no other details given) | Unclear risk |
| Chappell et al 1999 | Double-blind, placebo-controlled parallel group RCT | UK | 283 pregnant women at high risk of pre-eclampsia (abnormal two stage uterine-artery Doppler analysis or history of pre-eclampsia) at 16-22 weeks gestation | 1,000mg vitamin C and 400 IU vitamin E daily until delivery (n=141) | Placebo until delivery (n=142) | None reported | Unclear risk |
| Huria et al 2010 | Double-blind, placebo-controlled parallel group RCT | India | 285 pregnant women at risk of pre-eclampsia, primigravida, singleton pregnancy, normotensive on entry | 1,000mg vitamin C and 200 IU vitamin E daily from 12 weeks until delivery (n=145) | Placebo from 12 weeks until delivery (n=140) | Iron-folic acid | Unclear risk |
| Kalpdev et al 2011 | Parallel group RCT | India | 50 pregnant women at risk of pre-eclampsia (essential hypertension), enrolled between 13-19 weeks gestation | 1,000mg vitamin C and 400 IU vitamin E daily from 2^nd^ trimester until delivery (n=22) | No supplement (n=22) | Iron, folic acid and calcium as per clinic protocol | High risk |
| McCance et al 2011 | Multicentre, double-blind, placebo-controlled parallel group RCT | UK | 762 pregnant women with Type 1 diabetes preceding pregnancy, singleton pregnancy, between 8-22 weeks gestation, 16 years or older | 1,000mg vitamin C and 400 IU vitamin E (alpha-tocopherol) daily until delivery (n=379) | Placebo (calcium carbonate, microcrystalline cellulose, maltodextrin and stearic acid/ olive oil) until delivery (n=383) | None reported | Low risk |
| Nasrolahi et al 2006 | Parallel group RCT | Iran | 580 healthy pregnant women, primiparious with singleton pregnancy | 1,000mg vitamin C and 400 IU vitamin E daily until delivery (n=290) | No supplement (n=290) | Iron | High risk |
| Poston et al 2006 | Multicentre, double-blind, placebo-controlled parallel group RCT | UK and Netherlands | 2410 pregnant women at risk of pre-eclampsia, at gestational age 14-21 weeks | 1,000 mg vitamin C and 400 IU vitamin E (RRR alpha-tocopherol) daily from second trimester until delivery (n=1,199) | Placebo (sunflower seed oil) from second trimester until delivery (n= 1205) | None reported | Low risk |
| Roberts et al 2010 | Multicentre, double-blind, placebo-controlled parallel group RCT | USA | 10,154 healthy pregnant women, nulliparous, singleton pregnancy, normotensive on entry, at less than 16 weeks gestation | 1,000 mg/d ascorbic acid and 400 IU/d vitamin E (RRR alpha-tocopherol acetate) until delivery (n=5,088) | Placebo (mineral oil) until delivery (n= 5,066) | None reported | Low risk |
| Rumbold et al 2006 | Multicentre, double-blind, placebo-controlled parallel group RCT | Australia | 1,877 healthy pregnant women with singleton pregnancy, normotensive on entry, at 14-22 weeks gestation | 1,000 mg ascorbic acid and 400 IU vitamin E (*d* alpha-tocopherol succinate) daily until delivery (n=935) | Placebo until delivery (microcrystalline cellulose) until delivery (n=942) | Routine prenatal multivitamin with no more than 200mg vitamin C and 50 IU vitamin E daily | Low risk |
| Spinnato et al 2007 | Multicentre, double-blind, placebo-controlled parallel group RCT | Brazil | 739 pregnant women at risk of pre-eclampsia (chronic hypertension or pre-eclampsia in most recent pregnancy), at 12-19 weeks gestation | 1,000 mg ascorbic acid and 400 IU vitamin E (*d* alpha-tocopherol acetate) daily until delivery or development of pre-eclampsia (n=371) | Placebo (gelatin, soybean oil, glycerin, water lecithin and caramel colour) until delivery or development of pre-eclampsia (n=368) | None reported | Low risk |
| Taghriri and Danesh 2007 | Parallel group RCT | Iran | 150 healthy pregnant women, primigravid, age 20-35, singleton pregnancy at 20 weeks gestation | 500 mg vitamin C and 400 IU vitamin E daily (n=75) | No supplement (n=75) | None reported | High risk |
| Villar et al 2009 | Multicentre, double-blind, placebo-controlled parallel group RCT | India, Peru, South Africa, Vietnam | 1365 pregnant women at risk of pre-eclampsia, at gestational age 14-21 weeks | 1,000 mg vitamin C and 400 IU vitamin E (RRR alpha-tocopherol) daily from second trimester until delivery (n=687) | Placebo (microsrystalline cellulose tablets, sunflower seed oil) from second trimester until delivery (n= 678) | None reported | Low risk |
| Xu et al 2010 | Multicentre, double-blind, placebo-controlled parallel group RCT | Canada, Mexico | 2,640 healthy pregnant women at 12-18 weeks gestation | 1,000 mg ascorbic acid and 400 IU vitamin E (200 IU d alpha-tocopherol, 200 IU alpha-tocopherol-acetate) daily from second trimester until delivery (n=1315) | Placebo (n=1325) | None reported | Unclear risk |

## Vitamin E

| **Reference** | **Study design** | **Country** | **Participants** | **Intervention** | **Comparison** | **Co-interventions** | **Risk of bias** |
| --- | --- | --- | --- | --- | --- | --- | --- |
| Bastani et al 2011 | Parallel group RCT | Iran | 272 healthy pregnant women, singleton pregnancy, normotensive on entry, with less than 14 weeks gestation | 400 IU vitamin E (RRR alpha-tocopherol-acetate) daily from week 14 until delivery (n=104) | No supplement (n=168) | None reported | High risk |
| Mahdy et al 2013 | Double-blind, placebo-controlled, parallel group RCT | Malaysia | 299 healthy, pregnant women, primigravidae with singleton pregnancies, normotensive on entry, between 12-16 weeks gestation | 100mg tocotrienol-rich fraction (TRF) red palm oil daily until delivery (n=151) | Placebo until delivery (n=148) | None reported | High risk |

# Vitamin D and/or Calcium

## Vitamin D

| **Reference** | **Study design** | **Country** | **Participants** | **Intervention** | **Comparison** | **Co-interventions** | **Risk of bias** |
| --- | --- | --- | --- | --- | --- | --- | --- |
| Asemi et al 2013 | Double-blind, placebo-controlled, parallel group RCT | Iran | 48 healthy pregnant women, primigravida, 18–40 years old, singleton  pregnancy at 25 weeks of gestation | 400 IU cholecalciferol (D_3_) daily for 9 weeks (n=24) | Placebo (microcrystalline cellulose) for 9 weeks (n=24) | 400 mcg folic acid daily from start of pregnancy and 60 mg iron from 2^nd^ trimester | Unclear risk |
| Brooke et al 1980 | Double-blind, placebo-controlled, parallel group RCT | UK | 126 Asian (first generation immigrants) healthy pregnant women at 28-32 weeks gestation | 1,000 IU ergocalciferol (D_2_) daily until delivery (n=59) | Placebo until delivery (n=67) | None reported | High risk |
| Chawes et al 2016 | Double-blind, placebo-controlled, parallel group RCT | Denmark | 623 healthy pregnant women at 24 weeks gestation, vitamin D intake ≤600 IU/d | 2,400 IU cholecalciferol (D_3_) daily until 1 week postpartum (n=315) | Placebo until 1 week postpartum (n=308) | Routine care included daily 400 IU cholecalciferol (D_3_) and 2.4 g/d long chain n-3 polyunsaturated fatty acids | Unclear risk |
| Cooper et al 2016 | Multi-centre, double-blind, placebo-controlled, parallel group RCT | UK | 1,134 healthy pregnant women older than 18 years old, singleton  pregnancy at 10-17 weeks of gestation,  serum 25 (OHD) 25–100 nmol/L | 1,000 IU cholecalciferol (D_3_) daily from 14-17 weeks until delivery (n=569) | Placebo 14-17 weeks until delivery (n=565) | Routine care included daily antenatal multivitamin, containing 400 IU vitamin D | High risk |
| Dawodu et al 2013 | Double-blind, placebo-controlled, parallel group RCT, three arm | USA | 192 Arab healthy pregnant women, singleton  pregnancy at 12–16 weeks of gestation | Group 1: 2,000 IU cholecalciferol (D_3_) (n=65); Group 2 4,000 IU cholecalciferol (D_3_) (n=63), all daily until delivery  **combined in current review* | Placebo until delivery (n=64) | Routine care included daily antenatal multivitamin, containing 400 IU vitamin D | Unclear risk |
| Delvin et al 1986 | Parallel group RCT | France | 40 healthy pregnant women, singleton pregnancy at 3^rd^ trimester | 1,000 IU cholecalciferol (D_3_) daily until delivery (n=20) | No supplement (n=20) | None reported | High risk |
| Grant et al 2013 | Double-blind, placebo-controlled, parallel group RCT | New Zealand | 260 healthy pregnant women, singleton pregnancy at 26-30 weeks of gestation, not taking vitamin D supplement >200 IU/d | 1,000 IU cholecalciferol (D_3_) daily until delivery (n=87); Group 2: 2,000 IU/d cholecalciferol (D_3_) until delivery (n=87)  **combined in current review* | Placebo until delivery (n=87) | None reported | Unclear risk |
| Hashemipour et al 2014 | Parallel group RCT | Iran | 130 Iranian healthy pregnant women at 24–26 weeks of gestation, serum 25 (OHD) <30 ng/ml | 50,000 IU cholecalciferol (D_3_) weekly for 8 weeks from enrollment until delivery (n=65) | No supplement (n=65) | Daily multivitamin with 400 IU vitamin D3 plus 200 mg elemental calcium  until delivery | High risk |
| Hossain et al 2014 | Parallel group RCT | Pakistan | 200 healthy pregnant women, singleton pregnancies at ≤20 weeks gestation | 4000 UI cholecalciferol (D_3_) daily from 20 weeks until delivery (n=100) | No supplement (n=100) | Routine care includes 400 mg iron and 600 mg calcium lactate daily | High risk |
| Jamilian et al 2018 | Double-blind, placebo-controlled, parallel group RCT, | Iran | 90 pregnant women with gestational diabetes  mellitus, primigravida, 18–40 years old at 24–28 weeks gestation | 50,000 IU cholecalciferol (D_3_) and probiotic every two weeks from enrollment until delivery (n=30) | Probiotic only (n=30)  **3^rd^ arm with no supplement not included in current review, n=30* | Routine care 1,000 IU vitamin D_3_ and daily 400 mcg folic acid from beginning of pregnancy and 60 mg iron from 2^nd^ trimester | Low risk |
| Karamali et al 2015 | Double-blind, placebo-controlled, parallel group RCT, | Iran | 60 pregnant woman at risk for pre-eclampsia, primigravida, 18–40 years old | 50,000 IU cholecalciferol (D_3_) every two weeks for 12 weeks from 20 to 32 weeks of gestation (n=30) | Placebo (edible paraffin) for 12 weeks from 20 to 32 weeks of gestation (n=30) | Routine care 400 mcg folic acid daily from beginning of pregnancy and daily 60 mg iron and multivitamin with 400 I vitamin D_3_ from 2^nd^ trimester | Unclear risk |
| Khan et al 2016 | Double-blind, placebo-controlled, parallel group RCT | Pakistan | 115 healthy pregnant women, from 12-16 weeks gestation | 4,000 IU vitamin D (type not reported) daily until delivery (n=36) | Placebo until delivery (n=49) | None reported | Unclear risk |
| Litonjua et al 2016 | Multi-centre, double-blind, placebo-controlled, parallel group RCT | USA | 835 pregnant women between 18-39 years old at 10-18 weeks gestation with a history of asthma, eczema, or allergic rhinitis, non-smoker | 4,000 UI cholecalciferol (D_3_) daily until delivery (n=440) | Placebo until delivery (n=436) | Daily multivitamin with 400 IU vitamin D_3_ | Unclear risk |
| Marya et al 1988 | Parallel group RCT | India | 200 healthy pregnant women with uncomplicated singleton pregnancies between 22-35 years old | 2 doses of  600,000 IU cholecalciferol (D_3_) at 7^th^ and 8^th^ month pregnancy (n=100) | No supplement (n=100) | None reported | High risk |
| Mohammad-Alizadeh-Charandabi et al 2015 | Triple-blind, placebo-controlled, parallel group RCT, three arms | Iran | 126 healthy pregnant women, 18-39 years old at 25 -30 weeks gestation | Group 1: Daily 1,000 IU vitamin D (type not specified) (n=42); Group 2: Daily 1,000 IU vitamin D and 300 mg calcium carbonate for 60 days  **comparison between vitamin D (group 1) and placebo)* | Placebo for 60 days | Majority had multivitamin and iron supplement during pregnancy (81-97%, not significant difference between groups, 43-52% had calcium supplementation in pregnancy | Unclear risk |
| Naghshineh and Sheikhaliyan 2016 | Double-blind, placebo-controlled, parallel group RCT | Iran | 140 healthy pregnant women, nulliparous, less than 16 weeks gestation without sign of vitamin D deficiency | 600 IU vitamin D (type not specified) daily until delivery (n=70) | Placebo until delivery (n=70) | None reported | Unclear risk |
| Razavi et al 2017 | Double-blind, placebo-controlled, parallel group RCT, four arms | Iran | 120 pregnant women with gestational diabetes at 24-28 weeks gestation, age 18-40 years | Group 1: Daily 240 mg DHA + 360 mg EPA (n=30); Group 2: Daily 240 mg DHA + 360 mg EPA and 50 000 IU every two weeks (n=30); Group 3: 50 000 IU vitamin D every two weeks – all for six weeks from enrollment  **comparison between vitamin D (group 3) and placebo* | Group 4: Placebo (liquid paraffin) (n=30) | None reported | Unclear risk |
| Roth et al 2013 | Double-blind, placebo-controlled, parallel group RCT | Bangladesh | 160 healthy pregnant women, 18-35 years old at 26-30 weeks gestation | 35,000 IU cholecalciferol (D_3_) weekly from 26-29 weeks until delivery (n=80) | Placebo, daily from 26-29 weeks until delivery (n=80) | Daily 66 mg iron and 350 mcg folic acid | Unclear risk |
| Sablok et al 2015 | Parallel group RCT | India | 180 healthy pregnant women, primigravidae with singleton  pregnancy at 14–20 weeks gestation | One dose of  60, 000 IU cholecalciferol (D_3_) at 20 weeks if sufficient at entry (>50 nmol/l), two doses of 120,000 IU cholecalciferol (D_3_) at 20 and 24 weeks for insufficient (25–50 nmol/l) and four doses of 120,000 IU cholecalciferol (D_3_) at 20, 24, 28 and 32 weeks for deficient (<25 nmol/l) (n=120) | No supplement (n=60) | None reported | High risk |
| Sasan et al 2017 | Double-blind, placebo-controlled, parallel group RCT | Iran | 142 pregnant women at risk of pre-eclampsia (history of PE in previous pregnancy), not vitamin D deficient (<25 nmol/l) | 50,000 IU cholecalciferol (D_3_) every two weeks from enrollment until 36 weeks gestation (n=70) | Placebo until 36 weeks gestation (n=72) | None reported | Unclear risk |
| Singh et al 2015 | Parallel group RCT | India | 100 healthy pregnant women, primigravida, singleton pregnancy at 12-16 weeks gestation | 2,000 IU cholecalciferol (D_3_) daily until delivery (n=50) | No supplement (n=50) | None reported | High risk |
| Valizadeh et al 2016 | Parallel group RCT | Iran | 96 pregnant women with gestational diabetes  mellitus, singleton pregnancy, older than 16 years old, at 12-32 weeks gestation | A total of 700,000 IU cholecalciferol (D_3_) during pregnancy (n=48) | No supplement (n=48) | Routine care included multivitamins  with calcium and 400 IU vitamin D_3_ | High risk |
| Yap et al 2014 | Double-blind, placebo-controlled, parallel group RCT | Australia | 179 healthy pregnant women, 18 years and older, at gestational age <20 weeks, singleton pregnancy with vitamin D 25 OHD <32 ng/mL no vitamin D  supplements ≥1000 IU/d | 5,000 IU cholecalciferol (D_3_) daily until delivery (n=89) | 400 IU cholecalciferol (D_3_) daily until delivery (n=90) | None reported | Unclear risk |
| Yu et al 2009 | Parallel group RCT, randomized in four blocks by ethnicity | UK | 180 healthy pregnant women (45 Indian Asians, 45 Middle Eastern, 45 Black and 45 Caucasian) at 27 weeks of gestation | Group 1: 800 IU ergocalciferol (D_2_) daily until delivery (n=60); Group 2: single oral dose of 200,000 IU calciferol (n=60)  **combined in current review* | No supplement (n=60) | None reported | High risk |
| Zerofsky et al 2014 | Double-blind, placebo-controlled, parallel group RCT | USA | 57 healthy pregnant women older than 18 years with singleton pregnancy <20 weeks gestation | 2,000 IU cholecalciferol (D_3_) daily until delivery (n=28) | Placebo (rice flour) (n=29) | Daily prenatal multi-micronutrient with 400 IU vitamin D_3_ | Unclear risk |

## Vitamin D and calcium

| **Reference** | **Study design** | **Country** | **Participants** | **Intervention** | **Comparison** | **Co-interventions** | **Risk of bias** |
| --- | --- | --- | --- | --- | --- | --- | --- |
| Asemi et al 2012 | Single-blind, placebo-controlled, parallel group RCT | Iran | 54 pregnant women at risk for pre-eclampsia, primigravida, 18–35 years old, singleton  pregnancy at 25 weeks of gestation | Daily 500mg calcium carbonate and 200 IU cholecalciferol for 9 weeks (n=27) | Placebo (lactose) for 9 weeks (n=27) | None reported | High risk |
| Asemi et al 2016 | Double-blind, placebo-controlled, parallel group RCT | Iran | 46 healthy pregnant women, singleton pregnancies, 18-40 years old at 25 week gestation | Daily 500 mg calcium carbonate and 200 IU cholecalciferol (D_3_) for 9 weeks (n=23) | Placebo (microcystallline cellulose) for 9 weeks (n=23) | Daily 400 mcg folic acid from beginning of pregnancy and daily 50 mg iron from 2^nd^ trimester | Low risk |
| Diogenes et al 2013 | Single-blind, placebo-controlled, parallel group RCT | Brazil | 84 adolescent (13-19 years old) primigravida, singleton  pregnancy at 23-29 weeks of gestation | Daily 600 mg calcium carbonate and 200 IU cholecalciferol (D_3_) from 26 weeks gestation until delivery (n=43) | Placebo (microcrystalline cellulose and corn starch) from 26 weeks gestation until delivery (n=41) | None reported | High risk |
| Li et al 2000 | Parallel group RCT, three arm | China | 88 pregnant women at risk for pre-eclampsia, BMI <24, at 20-24 weeks gestation | Group 1: 600 mg calcium carbonate and 800 IU cholecalciferol (D_3_) (n=29); Group 2: 1,200g calcium carbonate and  1,600 IU cholecalciferol (D_3_) (n=29); both daily until delivery  **combined in current review* | No supplement | None reported | High risk |
| Marya et al 1987 | Parallel group RCT | India | 400 pregnant women between 22-35 years old at 20-24 weeks gestation | Daily 1,200 IU cholecalciferol (D_3_) and 375mg calcium until delivery | No supplement | None reported | High risk |
| Mohammad-Alizadeh-Charandabi et al 2015 | Triple-blind, placebo-controlled, parallel group RCT with three arms | Iran | 126 healthy pregnant women, 18-39 years old at 25 -30 weeks gestation | Group 1: Daily 1,000 IU vitamin D (type and unit not specified) (n=42); Group 2: Daily 1,000 IU vitamin D and 300 mg calcium carbonate for 60 days **comparison between vitamin D and calcium (group 2) and placebo* | Placebo for 60 days | Majority had multivitamin and iron supplement during pregnancy (81-97%, not significant difference between groups, 43-52% had calcium supplementation in pregnancy | Unclear risk |
| Samimi et al 2016 | Double-blind, placebo-controlled, parallel group RCT | Iran | 60 pregnant women at risk for pre-eclampsia, primigravida, between 18-35 years old at 16-20 weeks gestation | 50,000 IU cholecalciferol (D_3_) every two weeks and 1,000 mg calcium carbonate daily from 20 to 32 weeks gestation (n=30) | Placebo from 20 to 32 weeks gestation (n=30) | Daily 400 mcg folic acid from beginning of pregnancy and 60 mg iron and multivitamin with 400 IU vitamin D and 500 mg calcium daily from 2^nd^ trimester | Unclear risk |
| Taherian et al 2002 | Parallel group RCT, three arms | Iran | 990 healthy nulliparous women with singleton pregnancy before 20 weeks gestation | Daily 200 IU cholecalciferol (D_3_) and 500 mg calcium carbonate from 20 weeks until delivery (n=330)  **2^rd^ arm with 75mg daily aspirin not included in current review (n=330)* | No supplement (n=330) | None reported | High risk |

## Calcium

| **Reference** | **Study design** | **Country** | **Participants** | **Intervention** | **Comparison** | **Co-interventions** | **Risk of bias** |
| --- | --- | --- | --- | --- | --- | --- | --- |
| Aghamohammadi and Zafari 2015 | Double-blind, placebo-controlled, parallel group RCT | Iran | 89 pregnant women at risk of pre-eclampsia over the age of 35, singleton pregnancy, ≤ 18 weeks gestation | 1,000 mg elemental calcium daily from 18-20 weeks gestation until delivery (n=44) | Placebo from 18-20 weeks gestation until delivery (n=44) (n=45) | None reported | High risk |
| Almirante 1998 | Parallel group RCT | Philippines | 430 pregnant women at high risk of pre-eclampsia, nulliparas, adolescents and elderly in their first trimester | 500 mg calcium carbonate daily from 16-20 weeks until delivery (n=212) | No supplement (n=210) | None reported | High risk |
| Bassaw et al 1998 | Parallel group RCT, four arms | Trinidad | 510 pregnant women at high risk of pre-eclampsia, primigravidae or multigravidae with history of pre-eclampsia before 20 weeks gestation | 1,200 mg elemental calcium daily until delivery (n=81)  **2^nd^ arm with aspirin (n=87) and 3^rd^ arm with aspirin and calcium (n=87) not included in current review* | No supplement (n=250) | 200 mg iron and 5 mg folic acid daily | High risk |
| Belizan et al 1991 | Multicentre, double blind, placebo-controlled, parallel group RCT | Argentina | 1195 pregnant women at risk of pre-eclampsia, nullipara, singleton pregnancies, before 20 weeks gestation | 2,000 mg calcium carbonate daily until delivery (n=593) | Placebo (lactose and granulated starch) until delivery (n=601) | None reported | Low risk |
| Boggess et al 1997 | Double-blind, placebo-controlled, parallel group RCT | USA | 23 healthy pregnant women between ages of 18-35 years old, normotensive on entry | 1,500mg daily from 28-31 weeks until delivery (n=12) | Placebo (starch) (n=11) | None reported | Unclear risk |
| Cong et al 1995 | RCT conducted in two phases (low dose then high dose) | China | 212 healthy pregnant women, primipara | Group 1: 120mg daily calcium (n=56); Group 2: 240mg daily calcium (n=56) **following Hofymeyr et al 2018, these groups were combined and high dose excluded due to concerns with data* | No supplement (n=56) | None reported | High risk |
| Crowther et al 1999 | Double-blind, placebo-controlled, parallel group RCT | Australia | 456 healthy pregnant women with a singleton pregnancy, nulliparas with less than 24 weeks gestation and normal blood pressure | 1,800 mg daily calcium carbonate until delivery (n=227) | Placebo (lactose) until delivery (n=229) | None reported | Unclear risk |
| Goldberg et al 2013 | Multicentre, double-blind, placebo-controlled, parallel group RCT | The Gambia | 662 healthy pregnant women, singleton pregnancy, at 18-22 weeks gestation | 1,500 mg calcium carbonate daily from 20 weeks until delivery (n=330) | Placebo (microcrystalline cellulose and lactose) until delivery (n=332) | None reported | High risk |
| Hofymeyr et al 2019 | Multicentre, double-blind, placebo-controlled, parallel group RCT | Argentina, South Africa, Zimbabwe | 1,355 pregnant women at risk of pre-eclampsia (previous pre-eclampsia or eclampsia) intending to become pregnant; 651 conceived during study | 500 mg daily elemental calcium from preconception to 20 weeks gestation, then 1,500 mg daily as recommended by WHO (n=678 randomized, of which 331 became pregnant) | Placebo from preconception to 20 weeks gestation, then 1,500 mg daily as recommended by WHO (n=677 randomized, of which 320 became pregnant) | None reported | Unclear risk |
| Kumar et al 2009 | Double-blind, placebo-controlled, parallel group RCT | India | 524 healthy pregnant women, primigravida, singleton pregnancy, normotensive at entry at 12-25 weeks gestation | 2,000 mg calcium carbonate daily until delivery (n=290) | Placebo (n=262) | None reported | High risk |
| Levine et al 1997 | Multi-centre double-blind, placebo-controlled, parallel group RCT | USA | 4589 healthy pregnant women, nulliparas, normotensive at entry and passed compliance test, at 13-21 weeks gestation | 2,000 mg calcium carbonate daily until delivery or development of pre-eclampsia or suspicion of urolithiasis (n=2,295) | Placebo until delivery, development of pre-eclampsia or suspicion of urolithiasis (n=2,294) | Daily routine supplements with 50 mg calcium, 30 mg iron, and 400IU vitamin D_2_ | Unclear risk |
| Lopez Jarmillo et al 1989 | Double-blind, placebo-controlled, parallel group RCT | Ecuador | 106 healthy pregnant women, nulliparas, normotensive at entry, before 24 weeks gestation, 25 years or younger | 2,000 mg elemental calcium daily from 23 weeks until delivery (n=55) | Placebo from 23 weeks until delivery (n=51) | None reported | High risk |
| Lopez Jarmillo et al 1990 | Double-blind, placebo-controlled, parallel group RCT | Ecuador | 54 pregnant women at risk of pre-eclampsia, nulliparous with positive roll over test at 28-30 weeks gestation | 2,000 mg elemental calcium daily from 28-32 weeks until delivery (n=22) | Placebo (starch) from 28-32 weeks until delivery (n=34) | None reported | Unclear risk |
| Lopez Jarmillo et al 1997 | Double-blind, placebo-controlled, parallel group RCT | Ecuador | 274 adolescent pregnancies under 17.5 years old, before 20 weeks gestation, normotensive on entry | 2,000 mg elemental calcium daily from 20 weeks until delivery (n=134) | Placebo from 20 weeks until delivery (n=140) | None reported | Unclear risk |
| Nenad et al 2011 | Placebo-controlled, parallel group RCT | Serbia | 9178 healthy pregnant women, nulliparous, at 14-23 weeks gestation | 2,000 mg elemental calcium daily until delivery (n=4590) | Placebo until delivery (n=4588) | None reported | High risk |
| Niromanesh et al 2001 | Double-blind, placebo-controlled, parallel group RCT | Iran | 30 pregnant women at high risk of pre-eclampsia, at 28-32 weeks gestation, normotensive on entry | 2,000 mg elemental calcium daily until delivery (n=15) | Placebo until delivery (n=15) | None reported | Unclear risk |
| Purwar et al 1996 | Double-blind, placebo-controlled, parallel group RCT | India | 201 healthy pregnant women, nulliparous, singleton pregnancy, normotensive on entry | 2,000 mg elemental calcium daily from 20 weeks until delivery (n=103) | Placebo from 20 weeks until delivery (n=98) | None reported | Unclear risk |
| Roger et al 1999 | Parallel group RCT, three arms | China (Hong Kong) | 369 Chinese pregnant women at risk of pre-eclampsia with MAP >60 mmHg, between 20-24 weeks gestation | 600 mg calcium daily from 22 to 32 weeks, then 1,000 mg daily until delivery (n= 154) | No supplement (n= 83)  **3^rd^ arm with aspirin group not included in current review (n=132)* | None reported | High risk |
| Sanchez-Ramos et al 1994 | Double-blind, placebo-controlled, parallel group RCT | USA | 67 pregnant women at risk of pre-eclampsia, nulliparas, positive roll-over test and positive angiotensin II infusion test at 20-24 weeks gestation, normotensive on entry | 2,000 mg calcium carbonate daily until delivery (n=33) | Placebo until delivery (n-34) | None reported | Low risk |
| Villar et al 1987 | Multi-centered, double-blind, placebo-controlled, parallel group RCT | USA, Argentina | 52 healthy pregnant women: 34 Black Americans and 18 White Argentineans, singleton pregnancy, between 18-30 years, negative roll-over test, primiparous or nulliparous | 1,500 mg calcium carbonate dailyfrom 26 weeks until delivery (n=25) | Placebo from 26 weeks until delivery (n=27) | Women at the American site also received multivitamin including 200 mg calcium and 100 mg magnesium  per day | Unclear risk |
| Villar and Repke 1990 | Double-blind, placebo-controlled, parallel group RCT | USA | 189 adolescent pregnancies (17 years old or younger), singleton pregnancies | 2,000 mg calcium carbonate daily until delivery (n=94) | Placebo until delivery (n=95) | Multivitamin including 200 mg calcium and 100 mg magnesium  per day | Unclear risk |
| Villar et al 2006 | Multi-centre, double-blind, placebo-controlled, parallel group RCT | Argentina, Egypt, Inida, Peru, South Africa, Vietnam | 8325 healthy pregnant women, primiparous women less than 20 weeks gestation | 1,500 mg calcium carbonate daily until delivery (n=4157) | Placebo (n=4168) | None reported | Low risk |
| Wanchu et al 2001 | Parallel group RCT | India | 100 healthy pregnant women, normotensive, primigravidae, less than 20 weeks gestation | 2,000 mg elemental calcium daily from 20 weeks until delivery (n=50) | No supplement (n=50) | None reported | High risk |

## Calcium and linoleic acid (polyunsaturated omega-6 fatty acid)

| **Reference** | **Study design** | **Country** | **Participants** | **Intervention** | **Comparison** | **Co-interventions** | **Risk of bias** |
| --- | --- | --- | --- | --- | --- | --- | --- |
| Hererra et al 1998 | Double-blind, placebo-controlled, parallel group RCT | Colombia | Pregnant women at risk of pre-eclampsia, primiparous | 600mg calcium and 450 mg linoleic acid daily in third trimester (n=44) | Placebo (starch and lactose) (n=45) | None reported | Low risk |
| Hererra et al 2006 | Multi-centred, placebo-controlled, parallel RCT | Bangledesh, Colombia | 220 pregnant women at risk of pre-eclampsia, primigravida, 19-35 years, 18-22 weeks gestation | 600mg calcium and 450mg linoleic acid daily from 18-22 weeks to delivery (n=25) | Placebo (n=25) | None reported | Unclear risk |

# Iron and/or folic acid

## Iron

| **Reference** | **Study design** | **Country** | **Participants** | **Intervention** | **Comparison** | **Co-interventions** | **Risk of bias** |
| --- | --- | --- | --- | --- | --- | --- | --- |
| Chan et al 2009 | Double-blind, placebo-controlled, parallel group RCT | Hong Kong (China) | 1,164 healthy pregnant women with singleton pregnancy at less than 16 weeks gestation and hemoglobin between 8-14 g/dl | 300 mg ferrous sulphate (60mg elemental iron) daily until delivery (n= 565) | Placebo (starch and lactose) until delivery (n= 599) | None reported | Low risk |
| Cogswell et al 2003 | Double-blind, placebo-controlled, parallel group RCT | USA | 275 healthy pregnant women, low income, before 20 weeks gestation, hemoglobin ≥110 g/L and a ferritin concentration ≥ 20 μg/L | 30 mg ferrous sulfate daily until 28 days and then supplemented according to Institute of Medicine (IOM) guidelines until delivery (n = 146) | Placebo until 28 days and then supplemented according to Institute of Medicine (IOM) guidelines until delivery (n=128) | “Almost all the women received some supplemental iron during pregnancy” | High risk |
| Eskeland et al 1997 | Double-blind, placebo-controlled, parallel group RCT, 3 arm | Norway | 90 healthy pregnant women with singleton pregnancy, at less than 13 weeks gestation, hemoglobin ≥110 g/L | Group 1: Daily 3.6mg heme iron (porcine blood) and 24mg iron fumarate (n=31); Group 2: Daily 27 mg iron fumerate and 100mg vitamin C (n=30) from 20 week until delivery **comparison between iron only (group 1) and placebo in current review* | Placebo until delivery (n=29) | None reported during pregnancy | High risk |
| Falahi et al 2011 | Triple-blinded, placebo-controlled, parallel group RCT | Iran | 148 healthy pregnant women, primigravidae, between 20-35 years, before 20 weeks gestation, hemoglobin > 110 g/L, and serum ferritin > 20 μg/L | 60 mg elemental iron (as ferrous sulphate) daily until delivery (n=70) | Placebo until delivery (n=78) | None reported | Unclear risk |
| Harvey et al 2007 | Single-blind, placebo-controlled, parallel group RCT | UK | 13 healthy pregnant women, non-anaemic with singleton pregnancy aged 18-40 years, less than 14 weeks gestation | 100mg elemental iron (as ferrous gluconate) daily from 16 weeks gestation until delivery (n=6) | Placebo from 16 weeks until delivery (n=7) | None reported | Unclear risk |
| Liu et al 2000 | Parallel group RCT, three arms | China | 300 healthy pregnant women at 24- 28 weeks gestation, hemoglobin higher than 100 g/L | Group 1: 100mg elemental iron (ferrous sulphate) with 500 mg vitamin C and B-complex vitamins daily; Group 2: 900mg ferrous sulphate daily, both for four consecutive weeks  **comparison between iron only (group 2) and placebo in current review* | No supplement | None reported | Unclear risk |
| Makrides et al 2003 | Double-blind, placebo-controlled, parallel group RCT | Australia | 430 healthy pregnant women with singleton or twin pregnancies, non-anaemic | 20 mg elemental iron (ferrous sulfate) daily from 20 weeks until delivery (n=216) | Placebo from 20 weeks until delivery (n=214) | None reported | Unclear risk |
| Ouladsahebmadarek et al 2011 | Double blind, placebo controlled, parallel group RCT, three arm | Iran | 960 healthy pregnant women in first trimester, hemoglobin >120 g/L and normotensive at entry | 30mg elemental iron daily from 13 weeks gestation until delivery (n=480) | Placebo until delivery (n=480) | Multiple micronutrient | Unclear risk |
| Siega-Riz et al 2006 | Double blind, placebo controlled, parallel group RCT | USA | 429 healthy pregnant women, less than 20 weeks gestation, singleton pregnancy, non-anaemic | 30mg elemental iron (ferrous sulphate) daily until 26-29 weeks gestation (n=218) | Placebo until 26-29 weeks gestation (n=211) | Multiple micronutrient (no iron) | High risk |

## Folic acid

| **Reference** | **Study design** | **Country** | **Participants** | **Intervention** | **Comparison** | **Co-interventions** | **Risk of bias** |
| --- | --- | --- | --- | --- | --- | --- | --- |
| Charles 2005 | Double blind, placebo controlled, parallel group RCT, three arm | UK | 2928 pregnant women under 30 weeks gestation | Group 1: 200 mcg folic acid daily (n=466); Group 2: 5mg folic acid daily (n=485)  **combined in current review* | Placebo (n=1,977) | None reported | High risk |
| Christian et al 2003/ Christian et al 2009 | Cluster, double blind, placebo controlled, parallel group RCT, five arm | Nepal | 4998 married pregnant women from 426 rural communities | Group 1: 400 mcg folic acid daily (n=941 ); Group 2: 400 mcg folic acid and 60 mg/d ferrous fumarate daily (n=957 ); Group 3: 400 mcg folic acid, 60 mg ferrous fumarate and 30 mg zinc sulphate daily (n= 999); Group 4: daily multiple folic acid-iron-zinc plus vitamin D 10 mcg, vitamin E 10 mg, vitamin B-1 1.6 mg, vitamin B-2 1.8 mg, niacin 20 mg, vitamin B-6 2.2 mg, vitamin B-12 2.6 mcg, vitamin C 100 mg, vitamin K 65 mcg, copper 2.0 mg, magnesium 100 mg (n=1050), all until 12 weeks postpartum for live births or ≥ 5 weeks after a miscarriage or stillbirth, all women also received vitamin A  **comparison between folic acid (group 1) and control in current review* | 1000 mcg vitamin A daily 12 weeks postpartum (n= 1,051) | Deworming albendazole 400 mg single dose in the second and third trimester | Unclear risk |
| Fleming et al 1968 | Double blind, placebo controlled, parallel group RCT | Nigeria | 53 pregnant women less than 26 weeks gestation, primigravida, PVC 27% of more | 5 mg folic acid every two weeks in second trimester and weekly in third trimester (n=27) | Placebo (lactose) (n=26) | Antimalarial (pyrimethamine tablet) | High risk |
| Kirke et al 1992 | Multi-centre double blind, placebo controlled, parallel group RCT | Ireland | 281 pregnant women with previous child with neural tube defects | Group 1: 360mcg folic acid (n=84); Group 2: 4,000 IU vitamin A, 400 IU vitamin D (calciferol); 1.5 mg vitamin B1 (thiamine hydrochloride), 1.5 mg vitamin B2 (riboflavin); 1 mg vitamin B6 (pyridoxine hydrochloride); 15 mg niacin (nicotinamide); 40 mg vitamin C; 480 mg calcium phosphate, and iron (252 mg  ferrous sulphate) daily (n=88); Group 3: multivitamin with folic acid (n=85), all daily for at least 2 months before conception until date of the third missed period  **comparison between folic acid (group 1) and no folic acid (group 2) in current review* | Nonrandomized control **following De-Regil et al 2015, this was excluded from current review* | None reported | Unclear risk |
| MRC 1991 | Multi-centre double-blind, placebo-controlled, parallel group RCT | UK, Hungary, Israel, Australia, Canada,  Russia, France | 1817 women with a history of giving birth to a child with NTD | Group 1: 4mg folic acid, 120mg ferrous sulphate, 240mg di-calcium phosphate (n=298); Group 2: 4mg folic acid, 120mg ferrous sulphate, 240mg di-calcium, 4,000 IU vitamin A; 400 IU vitamin D; 1.5 mg vitamin B1;1.5 mg vitamin B2; 10 mg vitamin B6; 40 mg vitamin C and 15 mg niacin  (nicotinamide) (n=295), Group 3: multiple micronutrient without folic acid (n=302), all daily until 12 weeks of pregnancy  **comparison between folic acid (group 1) and control in current review* | 120mg ferrous sulphate, 240mg di-calcium phosphate daily until 12 weeks of pregnancy (n=300) | None reported | Unclear risk |

## Iron-folic acid

| **Reference** | **Study design** | **Country** | **Participants** | **Intervention** | **Comparison** | **Co-interventions** | **Risk of bias** |
| --- | --- | --- | --- | --- | --- | --- | --- |
| Christian et al 2003/ Christian et al 2009 | Cluster, double blind, placebo controlled, parallel group RCT, five arm | Nepal | 4998 married pregnant women from 426 rural communities | Group 1: 400 mcg folic acid daily (n=941 ); Group 2: 400 mcg folic acid and 60 mg/d ferrous fumarate daily (n=957 ); Group 3: 400 mcg folic acid, 60 mg ferrous fumarate and 30 mg zinc sulphate daily (n= 999); Group 4: daily multiple folic acid-iron-zinc plus vitamin D 10 mcg, vitamin E 10 mg, vitamin B-1 1.6 mg, vitamin B-2 1.8 mg, niacin 20 mg, vitamin B-6 2.2 mg, vitamin B-12 2.6 mcg, vitamin C 100 mg, vitamin K 65 mcg, copper 2.0 mg, magnesium 100 mg (n=1,050), all until 12 weeks postpartum for live births or ≥ 5 weeks after a miscarriage or stillbirth, all women also received vitamin A  **comparison between iron-folic acid (group 2) and folic acid only (group 1) in current review* | 1000 mcg vitamin A daily until 12 weeks postpartum (n= 1051) | Deworming albendazole 400 mg single dose in the second and third trimester | Unclear risk |
| MRC 1991 | Multi-centre double-blind, placebo-controlled, parallel group RCT | UK, Hungary, Israel, Australia, Canada,  Russia, France | 1817 women with a history of giving birth to a child with NTD | Group 1: 4mg folic acid, 120mg ferrous sulphate, 240mg di-calcium phosphate (n=298); Group 2: 4mg folic acid, 120mg ferrous sulphate, 240mg di-calcium, 4,000 IU vitamin A; 400 IU vitamin D; 1.5 mg vitamin B1;1.5 mg vitamin B2; 10 mg vitamin B6; 40 mg vitamin C and 15 mg niacin  (nicotinamide) (n=295), Group 3: multiple micronutrient without folic acid (n=302), all daily until 12 weeks of pregnancy  **comparison between multiple micronutrient (group 2) and folic acid (group 1)* | 120mg ferrous sulphate, 240mg di-calcium phosphate daily until 12 weeks of pregnancy (n=300) | None reported | Unclear risk |
| Lee et al 2005 | Parallel group RCT, 5 arms | Korea | 154 healthy pregnant women in the first trimester | Group 1: 30 mg elemental iron (ferrous sulphate) and 175 mcg folic acid daily from first trimester until delivery (n=30); Group 2: 60 mg/d elemental iron (ferrous sulphate) and 350 mcg folic acid daily from first trimester until delivery (n=31), Group 3: 30 mg elemental iron (ferrous sulphate) and 175 mcg folic acid daily from 20 weeks until delivery (n=27); Group 4: 60 mg elemental iron (ferrous sulphate) and 350 mcg folic acid daily from 20 weeks until delivery (n=29)  **combined in current review* | No supplement (n=20) | None reported | High risk |
| Liu et al 2013 | Double blind, placebo controlled, parallel group RCT, three arms | China | 18,775 health pregnant women, nulliparous, at least 20 years old, hemoglobin greater than 10.0 g/dL at least than 20 weeks gestation | Group 1: 400mcg folic acid and 30mg elemental iron (as ferrous fumarate) (n=6,252); Group 2: 400mcg folic acid and 30mg elemental iron, 800 mcg vitamin A, 10 mg vitamin E, 5 mcg vitamin D, 70 mg vitamin C, 1.4 mg thiamine, 1.4 mg riboflavin, 1.9 mg vitamin B6, 2.6 mg vitamin B12, 18 mg niacin, 15 mg zinc, 2 mg copper, 150 mcg iodine and 65 mcg selenium (n= 6,262), all daily until delivery  **comparison between iron-folic acid (group 1) and folic acid only (control) in current review* | Control: folic acid only 400mcg daily until delivery (n=6,261) | None reported | Low risk |
| Meier et al 2003 | Multi-centre double-blind, placebo-controlled, parallel group RCT | USA | 144 adolescents 15-18 years old in their first pregnancy and adult women 19 or older  in their first or greater pregnancy, non-iron deficient | 60mg elemental iron (ferrous sulphate) daily until delivery (n=58) | Placebo until delivery (n=53) | Daily 1mg folic acid | High risk |
| Mendendez et al 1994 | Cluster, double-blind, placebo controlled, parallel group RCT | The Gambia | 550 pregnant women, multigravidae, less than 34 weeks gestation, | 60mg elemental iron (ferrous sulphate) daily from 23-24 weeks until delivery (n=273) | Placebo until delivery (n=277) | 5mg folic acid per week | High risk |
| Ndyomugyenyi and Magnussen 2000 | Double blind, placebo controlled, parallel group RCT, three arm | Uganda | 860 pregnant women in high risk malaria area, primigravidae, without severe anaemia | Group 1: Daily 120 mg elemental iron (iron extran) and weekly 5 mg folic acid (n=294)  **2^nd^ arm 300 mg daily chloroquine (n=284) not used in current review* | Placebo (no chloroquine or iron and folic acid) (n=282)  *3^rd^ arm | None reported | Unclear risk |
| Taylor et al 1982 | Parallel group RCT | UK | 48 healthy pregnant women | 65mg elemental iron (as ferrous sulphate) and 350 mcg folic acid daily from 12 weeks until delivery (n=21) | No treatment (n=24) | None reported | High risk |
| Zeng et al 2008 | Cluster, double blind, placebo controlled, parallel group RCT, three arm | China | 5828 healthy pregnant women less than 28 weeks from two poor rural counties | Group 1: daily UNIMAPP (n=1,912, 183 clusters); Group 2: daily 60 mg elemental iron and 400 mcg of folic acid (n=1899, 170 clusters)  **comparison between iron-folic acid and folic acid in current review* | 400 mcg of folic acid (n=2017, 178 clusters) | None reported | Unclear risk |
| Ziaei et al 2007 | Double blind, placebo controlled, parallel group RCT | Iran | 750 healthy pregnant women in early second trimester, with hemoglobin ≥132 g/L, age 17-35 with singleton pregnancy | 50mg elemental iron (as ferrous sulphate) daily until delivery (n=375) | Placebo until delivery (n=375) | 1mg daily folic acid | Unclear risk |

# Zinc

| **Reference** | **Study design** | **Country** | **Participants** | **Intervention** | **Comparison** | **Co-interventions** | **Risk of bias** |
| --- | --- | --- | --- | --- | --- | --- | --- |
| Castillo-Duran et al 2001 | Multi-centre, double blind, placebo controlled, parallel group RCT | Chile | 804 healthy pregnant adolescents before 20 weeks gestation, under 19 years age at estimated time of delivery | 20mg zinc daily until delivery (n=401) | Placebo until delivery (n=403) | 40mg iron daily | High risk |
| Caulfield et al 1999 | Double blind, placebo controlled, parallel group RCT | Peru | 1,295 healthy pregnant women with uncomplicated pregnancies, singleton pregnancy, low zinc intake, at 10-24 weeks gestation | 15mg zinc (zinc sulphate) daily until delivery (n=521) | Placebo without zinc until delivery (n=495) | 60mg iron and 250mcg folate, vitamin C daily | Unclear risk |
| Cherry et al 1989 | Double blind, placebo controlled, parallel group RCT | USA | 652 pregnant adolescent women less than 25 weeks gestation, low income | 30mg zinc (zinc gluconate) daily until delivery (n=268) | Placebo (cellulose) until delivery (n=288) | None reported | Unclear risk |
| Christian et al 2003/ Christian et al 2009 | Cluster, double blind, placebo controlled, parallel group RCT, five arm | Nepal | 4,998 married pregnant women from 426 rural communities | Group 1: 400 mcg folic acid daily (n=941 ); Group 2: 400 mcg folic acid and 60 mg ferrous fumarate daily (n=957 ); Group 3: 400 mcg folic acid, 60 mg ferrous fumarate and 30 mg zinc sulphate daily (n= 999); Group 4: daily multiple folic acid-iron-zinc plus vitamin D 10 mcg, vitamin E 10 mg, vitamin B-1 1.6 mg, vitamin B-2 1.8 mg, niacin 20 mg, vitamin B-6 2.2 mg, vitamin B-12 2.6 mcg, vitamin C 100 mg, vitamin K 65 mcg, copper 2.0 mg, magnesium 100 mg (n=1,050), all until 12 weeks postpartum for live births or ≥ 5 weeks after a miscarriage or stillbirth, all women also received vitamin A  **comparison between zinc-iron-folic acid (group 3) and iron-folic acid (group 2)* | 1000 mcg vitamin A daily until 12 weeks postpartum (n= 1051) | Deworming albendazole 400 mg single dose in the second and third trimester | Unclear risk |
| Danesh et al 2010 | Double blind, placebo controlled, parallel group RCT | Iran | 110 healthy pregnant women with previous preterm delivery, age 18-35 years, at 12-16 weeks gestation | 50mg zinc (zinc sulphate) daily until delivery (n=42) | Placebo until delivery (n=42) | None reported | High risk |
| Dijkhuizen et al 2004 | Double-blind, placebo-controlled parallel group RCT, 2x2 factorial design | Indonesia | 170 pregnant women <20 weeks gestation, singleton pregnancy | Group 1: 4.5 mg beta-carotene (n=43); Group 2: 30 mg zinc sulphate (n=44); Group 3: 4.5 mg beta carotene and 30 mg zinc sulphate (n=42), daily all until delivery  **comparison between zinc only (group 2) and placebo) in current review* | Placebo until delivery (n=41) | Iron and folic acid | Unclear risk |
| Goldenberg et al 1995 | Multi-centre, double blind, placebo controlled, parallel group RCT | USA | 589 medically indigent but otherwise healthy African-American pregnant women between 13-44 years old at 14-23 weeks gestation | 25mg zinc (zinc sulphate) daily from 19 weeks gestation until delivery (n=286) | Placebo until delivery(n=294) | Multivitamin with iron folic acid (but no zinc) | Unclear risk |
| Hafeez et al 2005 | Multi-centre, double blind, placebo controlled, parallel group RCT | Pakistan | 250 healthy pregnant women at 10-16 weeks gestation | 20mg zinc (zinc sulphate) daily until delivery (n=121) | Placebo until delivery (n=121) | Routine supplements of iron folic acid | Unclear risk |
| Hunt et al 1984 | Double blind, placebo controlled, parallel group RCT | USA | 213 healthy Hispanic pregnant women, 17 years or older, under 27 weeks gestation | 20mgzinc (zinc acetate) daily until delivery (n=107) | Placebo without zinc (n=106) | Daily multivitamin (8000 IU vitamin A, 400 IU vitamin D, 30 IU vitamin E, 2 mg thiamin  mononitrate, 2 mg riboflavin, 20 mg niacinamide, 5 mg pyridoxine HCI, 1 mg folic acid, 10 Zg vitamin  B12 (cyanocobalamin), 10 mg D-calcium pantothenate, 60 mg vitamin C, 100 mg calcium (as carbon  ate), 20 mg iron (as ferrous fumarate), 50 mg of magnesium (as oxide), 1 mg of manganese (as sulphate),  and 150 Zg iodine (as potassium iodide) | Unclear risk |
| Jonsson et al 1996 | Multi-centre, double blind, placebo controlled, parallel group RCT | Denmark | 2,000 healthy pregnant women less than 20 weeks gestation | 44mg elemental zinc daily until delivery (n=1,000) | Placebo until delivery (n=1,000) | None reported | High risk |
| Merialdi et al 2004 | Double-blind, placebo controlled, parallel group RCT | Peru | 242 pregnant women at 10-16 weeks gestation with singleton pregnancy | 25mg zinc (zinc sulphate) daily until delivery (n=109) | Placebo without zinc (n=113) | 60 mg iron (ferrous sulphate)  and 250 mg folic acid daily | High risk |
| Mahomed et al 1989 | Double blind, placebo controlled, parallel group RCT | UK | 500 pregnant women less than 20 weeks gestation | 20mg zinc (zinc sulphate) daily until delivery (n=246) | Placebo until delivery (n=248) | None reported | Low risk |
| Osendarp et al 2000 | Double blind, placebo controlled, parallel group RCT | Bangladesh | 559 pregnant women between 12-16 weeks gestation | 30mgelemental zinc daily in last two trimesters of pregnancy (n=269) | Placebo in the last two trimesters of pregnancy (n=290) | None reported | Unclear risk |
| Robertson 1991 | Double blind, placebo controlled, parallel group RCT | UK | 152 pregnant women before 18 weeks gestation | 62mg zinc (zinc sulphide) daily until pregnancy (n=62) | Placebo without zinc until delivery (n=62) | 150mg iron and 500 mcg folic acid daily | High risk |
| Saaka et al 2009 | Double blind, placebo controlled, parallel group RCT | Ghana | 400 pregnant women less than 16 weeks gestation | 40mg zinc (zinc gluconate) every other day until delivery (n=299) | Placebo without zinc (n=301) | 40mg iron (ferrous sulphate), 400mcg folic acid and antimalarial | Unclear risk |
| Simmer et al 1991 | Double blind, placebo controlled, parallel group RCT | UK | 56 Asian pregnant women between 15-25 weeks gestation, smoker, low pre-pregnancy weight with previous small for gestational age baby | 22.5mg elemental zinc daily until delivery (n=30) | Placebo until delivery (n=26) | None reported | Unclear risk |
| Xie et al 2001 | Double blind, placebo controlled, parallel group RCT, four arm | China | 146 pregnant women less than 12 weeks gestation | Group 1: 5mg zinc (zinc lactate) (n=27); Group 2: 10mg zinc (n=40); Group 3: 30mg zinc (n=39), all daily until delivery  **combined in current review* | Placebo (maize starch) until delivery (n=40) | None reported | Unclear risk |

# Multiple micronutrient (MMN)

## MMN

UNIMMAP consists of: 30 mg iron (ferrous fumarate) and 400 mcg folic acid along with 800 mcg retinol (retinyl acetate), 200 IU vitamin D (ergocalciferol), 10 mg vitamin E (α-tocopherol acetate), 70 mg ascorbic acid, 1.4 mg vitamin B1 (thiamine mononitrate), 18 mg niacin (niacinanide), 1.4 mg vitamin B2 , 1.9 mg vitamin B6 (pyridoxine), 2.6 mcg vitamin B12 (cyanocobalamin), 15 mg zinc (zinc gluconate), 2 mg copper, 65 mcg selenium, and 150 mcg iodine

| **Reference** | **Study design** | **Country** | **Participants** | **Intervention** | **Comparison** | **Co-interventions** | **Risk of bias** |
| --- | --- | --- | --- | --- | --- | --- | --- |
| Adu-Afarwuah et al 2015 | Double blind, placebo controlled, parallel group RCT, three arm | Ghana | 1,320 pregnant women at least 18 years old, 20 weeks gestation or earlier | Group 1: MMN 800mcg vitamin A, 2.8mg B1, 2.8mg B2, 3.8mg B6, 5.2 mcg B12, 100 mg C, 400 IU D, 20 mg E, 45 mcg K, 36 mg niacin, 400mcg folic acid, 7mg pantothenic acid, 20 mg iron, 30 mg zinc, 4mg copper, 130mcg selenium, 250 mcg iodine, 2.6mg manganese (n=439); Group 2: 20g LNS with MMN plus 2.6 protein, 10g fat, 4.59g linoleic acid,0.59 a-linolenic acid, 280mg calcium, 190mg phosphorous, 200 mg potassium, 65mg magnesium (n=440), both daily from enrollment until delivery  **comparison between MMN (group 1) and iron-folic acid (control)* | 60mg iron, 400mcg folic acid daily until delivery (n=441) | None reported | Low risk |
| Ashorn et al 2015 | Double blind, placebo controlled, parallel group RCT, three arm | Malawi | 1391 healthy pregnant women at least 15 years old, before 20 weeks gestation | Group 1: MMN 800mcg vitamin A, 2.8mg B1, 2.8mg B2, 3.8mg B6, 5.2 mcg B12, 100 mg C, 400 IU D, 20 mg E, 45 mcg K, 36 mg niacin, 400mcg folic acid, 7mg pantothenic acid, 20 mg iron, 30 mg zinc, 4mg copper, 130mcg selenium, 250 mcg iodine, 2.6mg manganese (n=466); Group 2: 20g LNS with MMN plus 2.6 protein, 10g fat, 4.59g linoleic acid,0.59 a-linolenic acid, 280mg calcium, 190mg phosphorous, 200 mg potassium, 65mg magnesium, all daily from enrollment until delivery (n=462)  **comparison between LNS with MMN (group 2) and iron-folic acid (control)* | 60mg iron, 400mcg folic acid daily until delivery n=463) | None reported | Low risk |
| Bhutta et al 2009 | Cluster, double blind, placebo controlled, parallel, RCT | Pakistan | 2,378 pregnant women less than 16 weeks gestation in 28 clusters (16 rural and 12 urban) | UNIMMAP daily until delivery (n=1,148) | 60 mg iron and 400 mcg folic acid daily until delivery (n=1,230) | Diet and nutritional counselling in half MMN and control groups, these are reported with their supplement groupings | Low risk |
| Brough et al 2019 | Multicentre double blind, placebo controlled, parallel, RCT | UK | 402 pregnant women 16 years or older with singleton pregnancy, less than 13 weeks gestation, socially deprived, multi-ethic population | 3mg beta-carotene, 3.6 mg thiamin, 3 mg, 2mg riboflavin, 20mg niacin, 10mg B6, 6mcg B12, 400 mcg folic acid,  70 mg C, 5mcg D, 20mg E, 70 mcg K, 20 mg Fe, 15mg zinc, 150mg Mg, 140 mcg iodine, 1mg copper daily until delivery (n=207) | Placebo (starch with iron oxide coating) daily until delivery (n=195) | Women not using folic acid were also given 400 mcg folic acid to take daily until 12 weeks of gestation. | Unclear risk |
| Christian et al 2003/ Christian et al 2009 | Cluster, double blind, placebo controlled, parallel group RCT, five arm | Nepal | 4998 married pregnant women from 426 rural communities | Group 1: 400 mcg folic acid daily (n=941 ); Group 2: 400 mcg folic acid and 60 mg/d ferrous fumarate daily (n=957 ); Group 3: 400 mcg folic acid, 60 mg ferrous fumarate and 30 mg zinc sulphate daily (n= 999); Group 4: daily multiple folic acid-iron-zinc plus vitamin D 10 mcg, vitamin E 10 mg, vitamin B-1 1.6 mg, vitamin B-2 1.8 mg, niacin 20 mg, vitamin B-6 2.2 mg, vitamin B-12 2.6 mcg, vitamin C 100 mg, vitamin K 65 mcg, copper 2.0 mg, magnesium 100 mg (n=1050), all until 12 weeks postpartum for live births or ≥ 5 weeks after a miscarriage or stillbirth, all women also received vitamin A  **comparison between multiple micronutrients (group 4) and control in current review* | 1,000 mcg/d vitamin A (n= 1051) | Deworming albendazole 400 mg single dose in the second and third trimester | Unclear risk |
| Fawzi et al 1998/ Merchant et al 2005 | Double-blind, placebo-controlled parallel group RCT, 2x2 factorial design | Tanzania | 1,075 HIV positive pregnant women between 12-27 weeks gestation | Group 1: Daily 30 mg beta-carotene, 5000 IU preformed vitamin A, 200,000 IU vitamin A at delivery (n=269); Group 2: Multivitamin excluding vitamin A, 20 mg B1, 20 mg B2, 25 mg  B6, 100 mg niacin, 50 μg B12, 500 mg C, 30 mg E, and 0·8 mg  folic acid (n=269), Group 3: multivitamins including vitamin A, 200,000 IU vitamin A at delivery (n=270)  **comparison between MMN (group 3) and placebo in current review* | Placebo (n=267) | Daily 400 mg ferrous sulphate and 5mg folate as well as 500 mg prophylactic chloroquine  Phosphate weekly | Unclear risk |
| Fawzi et al 2007 | Multicentre double blind, placebo controlled, parallel, RCT | Tanzania | 8,468 pregnant women between 12-27 weeks gestation, HIV negative | 20mg B1, 20mg B2, 25mg B6, 50 mcg B12, 500mg C, 30mg E, niacin 100mg, 0.8mg folic acid, 60mg iron, 0.25mg folic acid daily from enrollment until 6 weeks postpartum (n=4,214) | 60mg iron, 0.25mg folic acid from enrollment until 6 weeks after delivery (n=4,214) | Malaria prophylaxis (sulphadoxine-pyrimethamine tablets) at 20 and 30 weeks of gestation was | Low risk |
| Friis et al 2004 | Double blind, placebo controlled, parallel group RCT | Zimbabwe | 1,669 pregnant women between 22-36 weeks gestation | 3,000mcg vitamin A, 3.5mg beta carotene, 1.5 mg thiamine, 1.6 mg riboflavin, 2.2mg B6, 4 mcg B12, 17mcg niacin, 80mg C, 10mcg D, 10mg E, 15mg zinc, 1.2mcg copper, 65mcg selenium daily from enrollment until delivery (n=837) | Placebo until delivery (n=832) | Iron-folic acid tablet as part of routine antenatal care | Low risk |
| Gupta et al 2007 | Double blind, placebo controlled, parallel group RCT | India | 200 pregnant women with BMI <18.5 and/or hemoglobin 7-9g/dL at 24-32 weeks gestation, 18-44 years with singleton pregnancy | 2500 IU beta carotene, 1mg thiamine mononitrate (B1) 1.5mg, riboflavin (B2), 1mg pyridoxine hydrochloride (B6), 1 mcg cyanocobalamin (B12), 50mg ascorbic acid  (C), 200 IU cholecalciferol (D_3_),7.5mg tocopherol acetate ( E), 5 mg; calcium pantothenate,  150 mcg; folic acid, 20 mg nicotinamide (niacinamide), 30 mcg biotin, 15mg zinc, 150 mcg potassium iodide, 10 mg ferrous  fumarate, 100 mg magnesium oxide (light), 2.5 mg manganese sulfate, 2 mg copper, 162 mg calcium, 125 mg phosphorus, 40 mg potassium, 36.3 mg chloride, 25mcg chromium,  25 mcg molybdenum,30 mcg sodium selenate, 5 mcg nickel, 2mg  silicon dioxide, 10 mcg vanadium, 150 mcg boron daily until delivery (n=99) | Placebo (calcium with chocolate flavouring) (n=101) | 60mg iron and 500mcg folic acid as part of routine care | Unclear risk |
| ICMR 2000 | Multicentre double blind, placebo controlled, parallel group RCT | India | 466 women with a history of giving birth to a child with open NTD | Daily 120mg ferrous sulphate, 240mg calcium phosphate, 4000 IU vitamin A, 400 IU vitamin D, 2.5mg vitamin B1, 2.5mg vitamin B2, 2mg vitamin B6, 15mg nicotinamide, 40mg vitamin C, 4mg folic acid, 10mg zinc from at least 1 month before conception up to 12 weeks of pregnancy (n=127) | Daily 120mg ferrous sulphate and 240mg calcium phosphate from at least 1 month before conception up to 12 weeks of pregnancy (n=142) | None reported | Unclear |
| Johnson et al 2017 | Partially blinded, parallel group RCT with four arms | The Gambia | 620 pregnant women at 10-20 weeks gestation | Group 1: MMN UNIMMAP tablets doubled (n=146); Group 2: LNS only group: 746 kcal/day of energy from protein and lipid plus 60 mg iron and 400 mcg folic acid (n=151); Group 3: LNS with MMN UNIMMAP tablets doubled (n=159), all daily from 12 weeks until delivery  **comparison between MMN (group 1) and iron-folic acid (control)* | 400 mcg folic acid and 60 mg iron (n=146), daily from 12 weeks until delivery | None reported | High risk |
| Kaestel et al 2005 | Double blind, placebo controlled, parallel group RCT, three arm | Guinea-Bissau | 2100 pregnant women with less than 37 weeks gestation | Group 1: UNIMMAP single dose daily until delivery (n=695); Group 2: UNIMMAP – double dose (n=697) – **comparison between group 1 and control* | Folic acid 400 mcg and iron 60 mg daily until delivery (n = 708) | None reported | High risk |
| Kirke et al 1992 | Multi-centre double blind, placebo controlled, parallel group RCT | Ireland | 281 pregnant women with previous child with neural tube defects | Group 1: 360mcg folic acid (n=84); Group 2: 4000 IU vitamin A, 400 IU vitamin D (calciferol); 1.5 mg vitamin B1 (thiamine hydrochloride), 1.5 mg vitamin B2 (riboflavin); 1 mg vitamin B6 (pyridoxine hydrochloride); 15 mg niacin (nicotinamide); 40 mg vitamin C; 480 mg calcium phosphate, and iron (252 mg  ferrous sulphate) daily (n=88); Group 3: multivitamin with folic acid (n=85), all daily for at least 2 months before conception until date of the third missed period  **comparison between multiple micronutrient with folic acid (group 3) and folic acid only (group 1)* | Nonrandomized control **following De-Regil et al 2015, this was excluded from current review* | None reported | Unclear risk |
| Liu et al 2013 | Double blind, placebo controlled, parallel group RCT, three arms | China | 18,775 health pregnant women, nulliparous, at least 20 years old, hemoglobin greater than 10.0 g/dL at least than 20 weeks gestation | Group 1: 400mcg folic acid and 30mg elemental iron (as ferrous fumarate) (n=6,252); Group 2: 400mcg folic acid and 30mg elemental iron, 800 mcg vitamin A, 10 mg vitamin E, 5 mcg vitamin D, 70 mg vitamin C, 1.4 mg thiamine, 1.4 mg riboflavin, 1.9 mg vitamin B6, 2.6 mg vitamin B12, 18 mg niacin, 15 mg zinc, 2 mg copper, 150 mcg iodine and 65 mcg selenium (n= 6,262), all daily until delivery  **comparison between multiple micronutrient (group 2) and iron-folic acid (group 1) in current review* | Folic acid 400 mcg daily until delivery (n=6,261) | None reported | Low risk |
| Osrin et al 2005 | Double blind, placebo controlled, parallel group RCT | Nepal | 1,200 healthy pregnant women with singleton pregnancy between 12-20 weeks gestation | UNIMMAP daily until delivery (n=600) | Iron 60 mg and folic acid 400 mcg daily until delivery (n=600) | None reported | High risk |
| Ramakrishan et al 2003 | Double blind, placebo controlled, parallel group RCT | Mexico | 873 pregnant women before 13 weeks gestation | 60mg iron,  215 mcg folic acid, 2150 IU vitamin A, 209 IU D3, 5.73 IU E, 0.93 mg thiamin, 1.87 riboflavin, 15.5mg niacin, 1.94 B6, 2.04 mcg B12, 66.5mg C, 12.9mg zinc, 252 mg magnesium from enrollment 6 days a week until delivery (n=435) | Iron 60 mg 6 days a week until delivery (n=438) | None reported | High risk |
| Roberfroid et al 2008 | Double blind, placebo controlled, parallel group RCT | Burkina  Faso | 1426 pregnant women | UNIMMAP daily until delivery (n=714) | Iron 60 mg and folic acid 400 mcg daily until delivery (n=712) | 300 mg chloroquine per week or 1500 mg sulfadoxine and 75 mg pyrimethamine once in the second and  third trimester | Low risk |
| Rumiris et al 2006 | Double blind, placebo controlled, parallel group RCT | Indonesia | 60 healthy pregnant women between 8-12 weeks gestation with low antioxidant status (SOD > 1,102 U/gHb) | Daily 1000IU vitamin A, 2.2 mg B6, 2.2 mcg B12, 200 mg vitamin C, 400IU vitamin E, 200 mg *N-*acetylcysteine, 2mg Cu, 15 mg zinc, 0.5mg magnesium, 800mg calcium, 100mcg selenium until delivery  (n=29) | Placebo until delivery (n=31) | 400 mg folic acid and 30mg iron | Unclear risk |
| Shankar et al 2008 | Cluster double blind, placebo controlled, parallel group RCT | Indonesia | 31,290 pregnant women of any gestational age on Lombok Island | UNIMMAP daily until delivery (n = 15,804) | 30 mg iron and 400 mcg folic acid daily until delivery (n = 15,486) | None reported | Low risk |
| Sunawang et al 2009 | Cluster single blind, placebo controlled, parallel group RCT | Indonesia | 843 healthy pregnant women at 12-20 weeks gestation in 80 clusters | UNIMMAP daily until 30 days after delivery (n=432) | 60 mg iron and 400 mcg folic acid daily until 30 days after delivery (n=1,480) | None reported | Unclear risk |
| Tofail et al 2008 / Persson et al 2012 | Double blind, placebo controlled, parallel group RCT, six arm | Bangladesh | 4,436 healthy pregnant women between 6-8 weeks gestation | UNIMMAP daily until delivery (n=1,480) | 30 mg iron and 400 mcg folic acid (n=1,480), 60 mg iron and 400 mcg folic acid (n=1,476), daily until delivery **combined in current review* | Food supplementation (608 kcal  6 days per week) at 9 or 20 weeks gestation | High risk |
| Vadillo-Ortega et al 2011 | Double blind, placebo controlled, parallel group RCT, three arm | Mexico | 444 pregnant women at risk of pre-eclampsia at 14-32 weeks gestation | Daily 4mg vitamin B6, 400 mcg folate, 9.6 mcg B12, 500 mg vitamin C, 400 UI E, 50mg niacin, 6.6 g L-arginine, 200mg potassium until delivery (n=228)  **2^nd^ arm of vitamins alone – not included in current review (n=222)* | Placebo until delivery (n=222) | None reported | High risk |
| West et al 2014 | Cluster double blind, placebo controlled, parallel group RCT | Bangladesh | 44,567 pregnant women age 12-45 years in 165 clusters | UNIMMAP daily until delivery (n=22,405) | 27 mg iron and 600 mcg folic acid daily until delivery (n= 22,162) | None reported | Low risk |
| Zagre et al 2007 | Cluster double blind, placebo controlled, parallel group RCT | Niger | 3,670 women experiencing amenorrhoea for < 12 weeks, without severe anaemia, in 78 rural clusters | UNIMMAP daily until delivery (n=1,893) | Iron-folic acid (amounts not reported but likely 30mg iron and 400mcg folic acid) until delivery (n=1,777) | None reported | Low risk |
| Zeng et al 2008 | Cluster, double blind, placebo controlled, parallel group RCT, three arm | China | 5828 healthy pregnant women less than 28 weeks from two poor rural counties | Group 1: daily UNIMAPP until delivery (n=1912); Group 2: daily 60 mg elemental iron and 400 mcg of folic acid until delivery (n=1,899)  **comparison between MMN (group 1) and folic acid in current review* | 400 mcg of folic acid (n=2,017) | None reported | Unclear risk |

## Lipid-based nutrient supplement (LNS)

| **Reference** | **Study design** | **Country** | **Participants** | **Intervention** | **Comparison** | **Co-interventions** | **Risk of bias** |
| --- | --- | --- | --- | --- | --- | --- | --- |
| Adu-Afarwuah et al 2015 | Double blind, placebo controlled, parallel group RCT, three arm | Ghana | 1,320 pregnant women at least 18 years old, 20 weeks gestation or earlier | Group 1: MMN 800mcg vitamin A, 2.8mg B1, 2.8mg B2, 3.8mg B6, 5.2 mcg B12, 100 mg C, 400 IU D, 20 mg E, 45 mcg K, 36 mg niacin, 400mcg folic acid, 7mg pantothenic acid, 20 mg iron, 30 mg zinc, 4mg copper, 130mcg selenium, 250 mcg iodine, 2.6mg manganese (n=439); Group 2: 20g LNS with MMN plus 2.6 protein, 10g fat, 4.59g linoleic acid,0.59 a-linolenic acid, 280mg calcium, 190mg phosphorous, 200 mg potassium, 65mg magnesium (n=440), both daily from enrollment until delivery  **comparison between LNS with MMN (group 2) and iron-folic acid (control)* | 60mg iron, 400mcg folic acid daily until delivery (n=441) | None reported | Low risk |
| Ashorn et al 2015 | Double blind, placebo controlled, parallel group RCT, three arm | Malawi | 1391 healthy pregnant women at least 15 years old, before 20 weeks gestation | Group 1: MMN 800mcg vitamin A, 2.8mg B1, 2.8mg B2, 3.8mg B6, 5.2 mcg B12, 100 mg C, 400 IU D, 20 mg E, 45 mcg K, 36 mg niacin, 400mcg folic acid, 7mg pantothenic acid, 20 mg iron, 30 mg zinc, 4mg copper, 130mcg selenium, 250 mcg iodine, 2.6mg manganese (n=466); Group 2: 20g LNS with MMN plus 2.6 protein, 10g fat, 4.59g linoleic acid,0.59 a-linolenic acid, 280mg calcium, 190mg phosphorous, 200 mg potassium, 65mg magnesium, all daily from enrollment until delivery (n=462)  **comparison between LNS with MMN (group 2) and iron-folic acid (control)* | 60mg iron, 400mcg folic acid daily until delivery (n=463) | None reported | Low risk |
| Huybregts et al 2009 | Open-label, parallel group RCT | Burkina Faso | 1296 pregnant women in two rural communities | Daily LNS consisting of 33% peanut butter, 32% soy flour, 15% vegetable oil, 22% sugar (14.7g protein, 27.6g fat, 26.9 carbohydrates) plus MMN cocktail equal to UNIMMAP (n=655) | Daily UNIMMAP (n=641) | None reported | High risk |
| Johnson et al 2017 | Partially blinded, parallel group RCT with four arms | The Gambia | 620 pregnant women at 10-20 weeks gestation | Group 1: MMN UNIMMAP tablets doubled (n=146); Group 2: LNS only group: 746 kcal/day of energy from protein and lipid plus 60 mg iron and 400 mcg folic acid (n=151); Group 3: LNS with MMN UNIMMAP tablets doubled (n=159), all daily from 12 weeks until delivery  **comparison between LNS with MMN (group3) and iron-folic acid (control)* | Daily 400 mcg folic acid and 60 mg iron (n=146), from 12 weeks until delivery | None reported | High risk |
| Mridha et al 2016 | Partially blinded, parallel group cluster RCT | Bangladesh | 4,011 pregnant women 20 weeks gestation or less in 11 rural clusters | Daily 20g day LNS made from peanut paste, soybean oil, powdered milk and sugar (2.6g protein, 10g fat) plus MMN modeled after UNIMMAP (twice daily amount of thiamin, riboflavin, niacin, vitamin  B-6, vitamin B-12, vitamin D, vitamin E, zinc, copper, and selenium, 20mg iron) (n=1,047) | Daily 60mg iron and 400mcg folic acid (n=2,964) | None reported | Unclear risk |

# Polyunsaturated omega-3 fatty acid

## Omega 3

| **Reference** | **Study design** | **Country** | **Participants** | **Intervention** | **Comparison** | **Co-interventions** | **Risk of bias** |
| --- | --- | --- | --- | --- | --- | --- | --- |
| Berghmann et al 2007 | Double blind, placebo controlled, parallel group RCT, three arm | Germany | 144 healthy pregnant Caucasian women, at least 18 years old | 600mg fish oil (200 mg DHA) plus prebiotic from 22 to 27 weeks gestation, resuming postpartum 2 weeks to 3 months (n=48) | Prebiotic only from 22 to 27 weeks gestation, resuming postpartum 2 weeks to 3 months (n=48)  **3^rd^ arm of basic supplement only not used in current review* | Basic supplement with vitamins and minerals (not further described) | Unclear risk |
| Bisgaard et al 2016 | Double blind, placebo controlled, parallel group RCT | Denmark | 736 pregnant women between 22-26 weeks gestation | 2,400 mg fish oil (890 mg DHA + 1,320 mg EPA) daily from 24 weeks until delivery (n=365) | Placebo (olive oil) until delivery (n=371) | None reported | Low risk |
| Bulstra-Ramakers et al 1994 | Double blind, placebo controlled, parallel group RCT | Netherlands | 63 women with a history of intrauterine growth retardation (<10^th^ centile) with or without pregnancy induced hypertension in the previous pregnancy between 12-14 weeks gestation | 3,000 mg EPA daily from enrollment to delivery (n=34) | Placebo until delivery (n=34) | None reported | Unclear risk |
| Carlson et al 2013 | Double blind, placebo controlled, parallel group RCT | USA | 350 healthy pregnant women between 8-20 weeks gestation, between 16-35.99 years old, singleton pregnancies | 600 mg DHA daily until delivery (n=178) | Placebo (half soybean, half corn oil, contained a-linoleic acid) until delivery (n=172) | None reported | Unclear risk |
| Dilli et al 2018 | Double blind, placebo controlled, parallel group RCT | Turkey | 140 pregnant women between 18-40 years old, 24-28 weeks gestation, with gestational diabetes mellitus | 252 mg DHA + 384 mg EPA daily from 26-27 weeks until delivery (n=70) | Placebo (sunflower oil) until delivery (n=70) | None reported | High risk |
| Dunstan et al 2003 (Dunstan et al 2008) | Double blind, placebo controlled, parallel group RCT | Australia | 98 healthy pregnant women with a history of allergic rhinitis and/or asthma less than 20 weeks gestation | 2,070mg DHA and 1,030mg EPA daily until delivery (n=52) | Placebo (olive oil) until delivery (n=46) | None reported | Unclear risk |
| Haghiac et al 2015 | Double blind, placebo controlled, parallel group RCT | USA | 72 pregnant women BMI ≥25 with singleton pregnancy and between 8-16 weeks gestation | 800 mg DHA + 1,200 mg EPA daily from 10-16 weeks until delivery (n=36) | Wheat germ oil until delivery (n=36) | None reported | High risk |
| Harper et al 2010 | Multicentre, double blind, placebo controlled, parallel group RCT | USA | 852 pregnant women with singleton pregnancies and between 16-22 gestational age with at least one prior spontaneous preterm birth | Fish oil (800 mg DHA + 1,200 mg EPA) daily from enrollment to 36 weeks (n=434) | Mineral oil from enrollment to 36 weeks (n=418) | 250mg 17q-hydroxyprogesterone caproate | Low risk |
| Harris et al 2015 | Multicentre, partially blind, placebo controlled, parallel group RCT, four arms | USA | 871 pregnant women age 18-40 years between 16-20 weeks gestation | Group 1: 300mg DHA (n=200); Group 2: 600mg DHA (n=200); Group 3: nutritional education for increasing omega-3 intake from foods (n=209)  **DHA groups are combined in comparison with* *control in current review* | Placebo (olive oil) (n=213) | None reported | High risk |
| Hauner 2012 | Parallel group RCT | Germany | 208 healthy pregnant women age 18-43 years at less than 15 weeks gestation | Fish oil (1,020 mg DHA + 180 mg EPA) and 9mg vitamin E from 15 weeks gestation to 4 month postpartum (n=104) | Diet counselling (n=104) | None reported | High risk |
| Helland et al 2001 | Multicentre, double blind, placebo controlled, parallel group RCT | Norway | 590 healthy pregnant women age 19-35 with singleton pregnancies, nulliparous or primiparous, at 17-19 weeks gestation | Cod-liver oil (1,183 mg DHA + 803 mg DHA) daily until delivery (n=301) | Corn oil until delivery (n=289) | None reported | High risk |
| Horvaticek et al 2017 | Placebo controlled, parallel group RCT | Croatia | 109 pregnant women with Type-1 diabetes mellitus with less than 9 weeks gestation | 616 mg DHA + 120 mg EPA daily until delivery (n=56) | Corn oil until delivery (n=53) | None reported | High risk |
| Jamilian et al 2016a | Double blind, placebo controlled, parallel group RCT | Iran | 54 pregnant women with singleton pregnancy and gestational diabetes at 24-28 weeks gestation | 120 mg DHA + 180 mg DHA from enrollment for 6 weeks (n=27) | Placebo for 6 weeks (n=27) | 400 mcg folic acid from beginning of pregnancy and 60 mg iron from second semester | Unclear risk |
| Lalooha et al 2012 | Single-blinded, placebo controlled, parallel group RC | Iran | 100 pregnant women at risk of pre-eclampsia, at 14-18 weeks gestation | 1,000 mg “omega 3 supplement” (DHA and EPA composition not reported) daily until delivery (n=50) | Placebo (starch) daily until delivery (n=50) | None reported | Unclear risk |
| Makrides et al 2010 / Zhao et al 2012 | Multicentre, double blind, placebo controlled, parallel group RCT | Australia | 2,399 pregnant women with singleton pregnancy less than 21 weeks gestation | Fish oil (800 mg DHA + 100 mg EPA) daily from enrollment to delivery (n=1,197) | Placebo (vegetable oil blend – rapeseed, sunflower and palm oils) daily until delivery (n=1,202) | None reported | Low risk |
| Malcolm 2003 | Double blind, placebo controlled, parallel group RCT | UK | 100 healthy pregnant women at 15 weeks gestation | Fish oil (200mg DHA) daily from enrollment to delivery (n=50) | Placebo (high-oleic sunflower oil) to delivery (n=50) | None reported | High risk |
| Mardones 2008 | Open label, placebo controlled, parallel group RCT | Chile | 1,173 pregnant women, with singleton pregnancies, age 18 years old or older, parity from 0 to 5, up to 20 weeks gestation and underweight | Milk product fortified with omega 3 (600 mg DHA) from enrollment to delivery (n=589) | Regular powered milk to delivery (n=552) | Milk product for both intervention and control included protein-energy and multiple micronutrients | High risk |
| Miller 2016 | Multicentre, double blind, placebo controlled, parallel group RCT | USA | 115 pregnant women age 18-42 years with singleton pregnancies | Fish oil (300 mg DHA + 67 mg EPA) from the last trimester to three months postpartum (n=60) | Placebo (high oleic acid sunflower oil) to three months postpartum (n=55) | None reported | Unclear risk |
| Min 2014 | Double blind, placebo controlled, parallel group RCT | UK | 173 pregnant women between 17-45 years with singleton pregnancy either pre-existing type 2 diabetes or without any known medical condition between 10-12 weeks gestation | Fish oil (600mg DHA) daily from enrollment to delivery (n=41 with type 2 diabetes, 45 healthy women) | Placebo (high oleic acid sunflower oil) daily until delivery (n=47 with type 2 diabetes, 40 healthy women) | Vitamin E (amount not given) | High risk |
| Min 2016 | Double blind, placebo controlled, parallel group RCT | UK | 138 pregnant women with singleton pregnancies diagnosed to gestational diabetes | Fish oil (600 mg DHA + 84 mg EPA) daily until delivery (n=67) | Placebo (high oleic acid sunflower oil) daily until delivery (n=71) | Vitamin E (amount not given) | Unclear risk |
| Mozurkewich 2013 | Multicentre, double blind, placebo controlled, parallel group RCT, three arm | USA | 126 pregnant women with a past history of depression, singleton pregnancy, 18 years or older at 12-20 weeks gestation | Group 1: Fish oil (274 mg DHA + 1060 mg EPA) (n=42); Group 2: Fish oil (900 mg DHA + 80 mg EPA) (n=42) daily from enrollment to 6-8 weeks postpartum, **combined in current review* | Placebo (soybean oil) daily until delivery (n=42 | None reported | Unclear risk |
| Olsen 1992 | Partially blinded, placebo controlled, parallel group RCT | Denmark | 533 healthy pregnant women with singleton pregnancy at approximately 30 weeks gestation, 18-44 years old | Fish oil (864 mg DHA + 621 mg EPA) daily from enrollment to delivery (n=266) | Placebo (olive oil) until delivery (n=136)  **No supplement group (n=131) not included in current review* | None reported | Unclear risk |
| Olsen 2000 | Multicentre, double blind, placebo controlled, parallel group RCT | Denmark, UK, Sweden, Italy, the Netherlands, Norway, Belgium, Russia | 1,647 high risk pregnant women with 1) history of preterm birth, IUGR, PIH or current twin pregnancies healthy after 16 weeks gestation, or 2) signs or symptoms of PE or IUGR in current pregnancy | Fish oil (900 mg DHA + 1,300 mg EPA in the prophylactic groups, n= 724; 2,100 mg DHA + 2,900 mg EPA in therapeutic groups, n= 80) from enrollment to delivery  **current review uses the prophylactic groups* | Placebo (olive oil, prophylactic groups n= 753; therapeutic groups n=62) from enrollment to delivery  **current review uses the prophylactic groups* | None reported | Unclear risk |
| Onwude 1995 | Double blind, placebo controlled, parallel group RCT | UK | 232 women with high risk pregnancy (history of LBW, pregnancy hypertension, unexplained stillbirth or primigravida with abnormal uterine Doppler at 24 weeks gestation) | Fish oil (1,080 mg DHA + 1,620 mg EPA) daily from enrollment to 28 weeks gestation (n=113) | Placebo (air-filled capsules) (n=119) | None reported | High risk |
| Ostadrahimi et al 2017 | Multicentre, double blind, placebo controlled, parallel group RCT | Iran | 150 pregnant women with singleton pregnancy, 18-35 years old, less than 6^th^ pregnancy | Fish oil (120 mg DHA + 180 mg EPA) daily from 20 weeks to 30 days postpartum (n=75) | Placebo (liquid paraffin) daily until 30 days postpartum (n=75) | None reported | Unclear risk |
| Ramakrishnan 2010 | Multicentre, double blind, placebo controlled, parallel group RCT | Mexico | 1,094 healthy pregnant women between 18-35 years old at 18-22 weeks gestation | Algal supplement (400 mg DHA) from enrolment until delivery (n=547) | Placebo (olive oil or corn/soy oil mix) daily until delivery (n=547) | None reported | Unclear risk |
| Razavi et al 2017 | Double-blind, placebo-controlled, parallel group RCT, four arms | Iran | 120 pregnant women with gestational diabetes at 24-28 weeks gestation, age 18-40 years | Group 1: Daily 240 mg DHA + 360 mg EPA (n=30); Group 2: Daily 240 mg DHA + 360 mg EPA and 50,000 IU every two weeks (n=30); Group 3: 50 000 IU every two weeks – all for 6 weeks from enrollment **comparison between omega 3 (group 1) and placebo in current review* | Placebo (liquid paraffin) for 6 weeks (n=30) | None reported | Unclear risk |
| Smuts 2003a | Double blind, placebo controlled, parallel group RCT | USA | 350 healthy pregnant women with singleton pregnancies, age 16-36 years, at 24-28 weeks gestation | DHA-enriched eggs (100 mg DHA) from enrollment until delivery (n=176) | Regular eggs until delivery (n=174) | None reported | Unclear risk |
| Smuts 2003b | Double blind, placebo controlled, parallel group RCT, three arm | USA | 72 healthy pregnant women age 16-35 years, at 24-28 weeks gestation | DHA enriched eggs (up to 135 mg DHA from enrollment until delivery (n=27) | Regular eggs until delivery (n=25)  **3^rd^ arm of not given eggs (n=21) was not included in current review* | None reported | High risk |
| Tofail et al 2006/ Tofail et al 2012 | Double blind, placebo controlled, parallel group RCT | Bangladesh | 400 pregnant women at 25 weeks gestation from low-income households | Fish oil (1.2 g DHA + 1.8 g EPA) daily from enrollment until delivery (n=200) | Placebo (soybean oil) until delivery (n=200) | None reported | High risk |
| Van Goor et al 2010 | Double blind, placebo controlled, parallel group RCT, three arm | Netherlands | 183 healthy pregnant women on first or secondary pregnancy between 14-20 weeks gestation | Group 1: Fish oil (220 mg DHA) and 220mg capsule arachidonic acid (n=58); Group 2: Fish oil (220mg DHA) and placebo (soy bean oil) (n=63) – from enrollment to 12 weeks postpartum **comparison between fish oil (group 2) and placebo* | Placebo (soy bean oil) (n=62) | None reported | High risk |

## Omega 3 and omega 6

| **Reference** | **Study design** | **Country** | **Participants** | **Intervention** | **Comparison** | **Co-interventions** | **Risk of bias** |
| --- | --- | --- | --- | --- | --- | --- | --- |
| D'Almedia et al 1992 | Double blind, placebo controlled, parallel group RCT, three arms | Angola | 150 pregnant women age 14-40 years old at 16 weeks gestation or less | Evening primrose oil and fish oil (80 mg DHA + 144 mg EPA + 296 GLA) daily for six months (n=50) | Placebo (olive oil) for six months (n=50)  **3^rd^ arm magnesium oxide (n=50) not used in current review* | None reported | Unclear risk |
| De Groot et al 2004 | Double blind, placebo controlled, parallel group RCT | Netherlands | 79 healthy pregnant, white women less than 14 weeks gestation | Enriched margarine 2.8g ALA + 9.0g LA from 14 weeks to delivery (n=40) | Enriched margarine 10.9g LA from 14 weeks to delivery (n=39) | None reported | High risk |
| Van Goor et al 2010 | Double blind, placebo controlled, parallel group RCT, three arm | Netherlands | 183 healthy pregnant women on first or secondary pregnancy between 14-20 weeks gestation | Group 1: Fish oil (220 mg DHA) and 220mg capsule arachidonic acid (n=58); Group 2: Fish oil (220mg DHA) and placebo (soy bean oil) (n=63) – from enrollment to 12 weeks postpartum **comparison between fish oil and arachidonic acid (group 1) and placebo* | Placebo (soy bean oil) (n=62) | None reported | High risk |

## Omega 3 and vitamin E

| **Reference** | **Study design** | **Country** | **Participants** | **Intervention** | **Comparison** | **Co-interventions** | **Risk of bias** |
| --- | --- | --- | --- | --- | --- | --- | --- |
| Jamilian et al 2016b | Double blind, placebo controlled, parallel group RCT | Iran | 60 pregnant women with gestational diabetes, age 18-40 years | Flaxseed oil (400mg a-linoleic acid and 400 IU vitamin E) daily for six weeks (n=30) | Placebo daily until delivery (n=30) | 400 mcg folic acid from beginning of pregnancy and 60mg iron | Unclear risk |

# Diet and nutritional counselling

## Antenatal dietary counselling

| **Reference** | **Study design** | **Country** | **Participants** | **Intervention** | **Comparison** | **Co-interventions** | **Risk of bias** |
| --- | --- | --- | --- | --- | --- | --- | --- |
| Bonomo et al 2005 | Parallel group RCT | Italy | 300 Caucasian pregnant women with mild gestational glucose intolerance, singleton pregnancies | Dietary counselling with biweekly follow-up (n=150) | No treatment (n=150)  **there was a third group of 150 normal gestational glucose tolerance that is not included in current review* | None reported | High risk |
| Briley et al 2002 | Parallel group RCT | USA | 27 healthy African American pregnant women at 24 weeks gestation | 6 antenatal individualized in-home nutrition assessment and counselling visits (n=15) | No counselling (n=12) | None reported | High risk |
| Crowther et al 2005 | Multicentre, parallel group RCT | Australia, UK | 1,000 pregnant women with gestational diabetes, 16-30 weeks gestation | Individualized dietary advice from dietitian and instruction on how to self-monitor glucose levels (n=490) | Standard care at each facility (n=510) | None reported | Unclear risk |
| Harris et al 2015 | Multicentre, partially blind, placebo controlled, parallel group RCT, four arms | USA | 871 pregnant women age 18-40 years between 16-20 weeks gestation | Group 1: 300mg DHA (n=200); Group 2: 600mg DHA (n=200); Group 3: nutritional education for increasing omega-3 intake from foods (n=209)  **comparison between nutritional education (group 3) and control in current review* | Placebo (olive oil) (n=213) | None reported | High risk |
| Jahan et al 2013 | Parallel group RCT | Bangledesh | 384 healthy pregnant women at 24 weeks gestation | Monthly 1-hour education session at the clinic for 3 months (n=192) | Routine care at health facility (n=192) | None reported | High risk |
| Kafatos et al 1989 | Parallel group RCT | Greece | 568 pregnant women with less than 27 weeks gestation | Nutritional counselling to promote foods with high nutrient value (n randomized unclear) | No counselling (n randomized unclear) | None reported | High risk |
| Khoury et al 2005 | Parallel group RCT | Norway | 290 white healthy pregnant women age 21-38 with singleton pregnancies at 17-20 weeks gestation with BMI 19-32 | Four dietitian counselling sessions to promote a cholesterol-lowering diet (fish, low fat meats and dairy products, oils, whole grains, fruits, vegetables and legumes) (n=141) | Usual diet (n=149) | Routine multivitamin | Unclear risk |
| Landon et al 2009 | Multicentre, parallel group RCT | USA | 958 pregnant women with mild gestational diabetes at 24-31 weeks gestation | Formal nutritional counselling and diet therapy, insulin provided if required (n=482) | Routine care at health facility (n=473) | None reported | Unclear risk |
| Peccei et al 2017 | Parallel group RCT | USA | 300 overweight or obese (BMI ≥25) pregnant women with singleton pregnancy at less than 16 weeks gestation | Dietary consultations with dietitian every 2 weeks from enrollment to 6 months postpartum (n=200) | Brochure and initial counselling on healthy eating guidelines (n=100) | None reported | Unclear risk |
| Thornton et al 2009 | Multicentre, parallel group RCT | USA | 257 obese pregnant women (BMI ≥ 30) with singleton pregnancy between 12-28 weeks gestation | Prescription of balanced nutritional regimen and food diary of daily consumption (n=124) | Routine care at health facility (n=133) | None reported | Unclear risk |
| Walsh et al 2012 | Parallel group RCT | Ireland | 800 women without diabetes, in their second pregnancy having previously delivered a macrosomic infant (>4kg), 18 years or older | One group dietary education session on low glycaemic diet at around 16 weeks gestation with two follow up meetings with dietitian (n=394) | Routine care at health facility (n=406) | None reported | Unclear risk |
| Wolff et al 2008 | Parallel group RCT | Denmark | 66 pregnant women without diabetes, non-smoking, Caucasian | 10 consultations with dietitian on eating a healthy diet according to official Danish dietary recommendations (n=23) | No dietary intervention (n=27) | None reported | Unclear risk |

## Antenatal diet and physical activity counselling

| **Reference** | **Study design** | **Country** | **Participants** | **Intervention** | **Comparison** | **Co-interventions** | **Risk of bias** |
| --- | --- | --- | --- | --- | --- | --- | --- |
| Bogaerts et al 2012 | Multi-centered, parallel group RCT, three arm | Netherlands | 205 obese (BMI >29) pregnant women 15 weeks gestation or less with singleton pregnancy | Group 1: four antenatal lifestyle intervention sessions to raise awareness on healthy eating and physical activity (n=78); Group 2: brochure about nutritional advise and physical activity (n=64)  **comparison between group 1 prenatal sessions with routine ANC in current review* | Routine antenatal care (n=63) | None reported | Unclear risk |
| Bruno et al 2016 | Parallel group RCT | Italy | 191 obese (BMI ≥25) pregnant women with singleton pregnancy older than 18 years old between 9-12 weeks gestation | 1 personalized dietary session with dietitian with prescription of a low-gylcaemic, low-saturated rat diet with total intake of 1,500kcal/day and physical exercise program (n=96) | Standard care (nutritional booklet with standard healthy nutrition and exercise recommendations (n=95) | None reported | High risk |
| Dodd et al 2016 | Multicentre, parallel group RCT | Australia | 2,212 overweight or obese (BMI ≥25) pregnant women between 10-20 weeks gestation without pre-existing diabetes | 6 sessions of individualized dietary advice from dietitian and physical activity goal setting (n=1,018) | Standard care at each facility (n=1,104) | None reported | Unclear risk |
| Guelinckx et al 2010 | Multicentre, parallel group RCT, three arms | Belgium | 195 white, obese (BMI > 29.0) pregnant women without diabetes before 15 weeks gestation | Group 1: brochure providing nutritional and physical activity advice (n=65); Group 2: 3 group sessions with nutritionist on balanced healthy diet and how to increase physical activity (n=65)  **comparison between counselling (group 2) and routine prenatal care in current review* | Routine prenatal care (n=65) | None reported | High risk |
| Luoto et al 2011 | Multicentre, parallel group, cluster RCT | Finland | 399 women at risk of gestational diabetes, between 18-40 years at 8-12 weeks gestation in 14 municipalities | 2 counselling sessions on dietary and physical activity counselling (n=246) | Routine care at health facility (n=196) | None reported | Unclear risk |
| Phelan et al 2011 | Multicentered, single blinded, parallel group RCT | USA | 401 pregnant women between 10-16 weeks gestation, older than 18 years old and BMI between 19.8-40 | One face to face visit to counsel on appropriate weight gain during pregnancy, physical activity and healthy diet and three brief supportive phone calls from the dietitian (n=200) | Routine care at health facility (n=201) | None reported | Unclear risk |
| Polley et al 2002 | Parallel group RCT | USA | 120 healthy pregnant women with singleton pregnancy less than 20 weeks gestation, BMI <19.8, at least 18 years of age | Written and oral dietary counselling on appropriate weight gain during pregnancy, exercise and healthful eating during pregnancy (n=61) | Routine care at health facility (n=59) | None reported | Unclear risk |
| Poston et al 2015 | Multicentre, parallel group RCT | UK | 1,555 obese pregnant women (BMI ≥ 30) with singleton pregnancy between 15-18 weeks gestation, older than 16 years old | Eight health trainer-led sessions to promote a healthy dietary pattern of eating and physical activity goals (n= 783 ) | Routine care at health facility (n= 772) | None reported | High risk |
| Renault et al 2014 | Parallel group RCT, three arm | Denmark | 425 obese pregnant women (BMI ≥ 30) with singleton pregnancy at less than 16 weeks gestation, older than 18 years | Group 1: 11-13 consultations via outpatient visits and phone contacts with dietitian for dietary advice and encouragement to increase physical activity as assessed by pedometer (n=142); Group 2: physical activity intervention only (n=142) - *current review compares group 1 with routine care* | Routine care at health facility (n=141) | None reported | Unclear risks |
