# Supplementary material for: Effects of Maternal Nutritional Supplements and Dietary Interventions on Placental Complications: An Umbrella Review, Meta-Analysis and Evidence Map
Source: Nutrients. 2021 Jan 30;13(2):472. doi: 10.3390/nu13020472 (PMC7912620; doi:10.3390/nu13020472)
Supplement: Supplementary file 1 [file nutrients-13-00472-s001.zip › Supplementary files/Table S9 - Individual trials outcomes.docx]

**Table S9: individual trials’ outcomes**

Contents

[Vitamin A 1](#_Toc55377289)

[Vitamin C and/or E 4](#_Toc55377290)

[Vitamin D and/or calcium 12](#_Toc55377291)

[Iron and/or folic acid 24](#_Toc55377292)

[Zinc 28](#_Toc55377293)

[Multiple micronutrients 30](#_Toc55377294)

[Lipid-based nutrients 39](#_Toc55377295)

[Polyunsaturated omega-3 fatty acid 41](#_Toc55377296)

[Antenatal dietary counselling with or without physical activity promotion 46](#_Toc55377297)

# Vitamin A

| **Reference** | **Nutrient** | **Outcome** | **Intervention** | | **Control** | | **Comparison** |
| --- | --- | --- | --- | --- | --- | --- | --- |
|  |  |  | **Cases** | **n** | **Cases** | **n** |  |
| Fawzi et al 1998/ Merchant et al 2005 | Vitamin A (vs placebo) | GH (≥110/90 mmHg anytime during pregnancy - included as in Rumbold et al 2008) | 45 | 480 | 44 | 475 | 9.4% vs 9.2% |
| West et al 1999 | Vitamin A | Eclampsia | 6 | 14 948 | 6 | 7241 | 0.0% (4 of 7747) vitamin A vs 0.0% (2 of 7201) beta carotene vs 0.0% (6 of 7241) placebo |
| Coutsoudis et al 1999 | Vitamin A | SGA | 43 | 346 | 43 | 343 | 12.4% vs 12.5% |
| Fawzi et al 1998 | Vitamin A (compared to no vitamin A) | SGA (< 10^th^ centile) | 48 | 521 | 57 | 500 | 12.4% vs 15.0% |
| West et al 2011, Christian et al 2013 | Vitamin A | SGA (< 10^th^ centile) | 6105 | 8636 | 3052 | 4348 | 70.0% (3050 of 4357) vitamin A vs 71.4% (3055 of 4279) beta-carotene vs of 70.2% (3052 of 4348) placebo |
| Coutsoudis et al 1999 | Vitamin A | LBW (<2500g) | 42 | 346 | 48 | 343 | 12.2% vs 14.1% |
| Cox et al 2005 | Vitamin A | LBW (<2500g) | 11 | 41 | 5 | 38 | 27% vs 13% |
| Fawzi et al 1998 | Vitamin A (compared to no vitamin A) | LBW (<2500g) | 47 | 521 | 51 | 500 | 11.6% vs 13.0% |
| Kumwenda et al 2002 | Vitamin A | LBW (<2500g) | 40 | 285 | 65 | 309 | 14.0% vs 21.1% |
| Radhika et al 2003 | Vitamin A | LBW (<2500g) | 10 | 64 | 12 | 58 | 15.6% vs 20.7%, |
| West et al 2011, Christian et al 2013 | Vitamin A | LBW (<2500g) | 5016 | 9129 | 2407 | 4580 | 54.8% (2521 of 4600) vitamin A vs 55.1% (2495 of 4529) vs 53.4% (2407 of 4508) placebo |
| Coutsoudis et al 1999 | Vitamin A | PTB (< 37 weeks) | 38 | 335 | 57 | 326 | 11.4% vs 17.4% |
| Dijkhuizen 2004 | Vitamin A | PTB | 1 | 43 | 3 | 41 | 2.3% vs 7.3% |
| Fawzi et al 1998 | Vitamin A (compared to no vitamin A) | PTB (< 37 weeks) | 105 | 521 | 97 | 500 | 23.4% vs 22.1% |
| Radhika et al 2003 | Vitamin A | PTB (< 37 weeks) | 9 | 64 | 11 | 58 | 13.6% vs 18.6% |
| van den Broek et al 2006 | Vitamin A | PTB | 29 | 468 | 13 | 232 | 7.2% (17 of 234) 5000 IU vs 5.2% (12 of 234) 10,000 IU vs 5.6% (13 of 232) placebo |
| West et al 2011, Christian et al 2013 | Vitamin A | PTB (< 37 weeks) | 6474 | 25487 | 3293 | 12713 | 25.2% (3201 of 12,702) vitamin A vs 25.6% (3273 of 12,785) beta carotene vs 25.9% (3293 of 12,713) placebo |
| Fawzi et al 1998 | Vitamin A (compared to no vitamin A) | Stillbirths | 25 | 521 | 24 | 500 | 4.8% vs 4.8% |
| Kirkwood et al 2010 | Vitamin A | Stillbirths (born dead at 6 months of gestation or later) | 1183 | 39601 | 1241 | 39234 | 3.0% vs 3.2% |
| Kumwenda et al 2002 | Vitamin A | Stillbirths | 6 | 317 | 8 | 306 | 1.9% vs 2.6% |
| West et al 2011 | Vitamin A | Stillbirths (born ≥28  weeks' gestation without moving or crying) | 1431 | 39804 | 703 | 19862 | 3.5% (703 of 19, 806) vitamin A vs 3.8% (766 of 19,998) beta carotene vs 3.5% (703 of 19,862) placebo |
| Dijkhuizen et al 2004 | Vitamin A | Maternal mortality | 0 | 43 | 1 | 41 | 0.0% vs 2.4% |
| Kirkwood et al 2010 | Vitamin A | Maternal mortality (All deaths occurring during pregnancy, at delivery, or up  to 42 days after delivery) | 138 | 39 601 | 148 | 39 234 | 0.0% vs 0.0% |
| Van den Broek et al 2006 | Vitamin A | Maternal mortality | 0 | 468 | 2 | 232 | 0% (0 of 234) 5000 IU vs 0% (0 of 234) 10,000 IU vs 0.9% (2 of 232) placebo |
| West et al 1999 | Vitamin A | Maternal mortality | 59 | 14948 | 51 | 7241 | 0.4% (33 of 7747) vitamin A vs 0.4% (26 of 7201) beta carotene vs 0.7% (51 of 7241) |
| West et al 2011 | Vitamin A | Maternal mortality (deaths during  gestation through the 12th week (84th  day) postpartum) | 97 | 39,804 | 41 | 19,862 | 0.2% (47 of 19, 806) vitamin A vs 0.3% (50 of 19,998) beta carotene vs 0.2% (41 of 19,862) placebo |

# Vitamin C and/or E

| **Reference** | **Nutrient** | **Outcome** | **Intervention** | | **Control** | | **Comparison** |
| --- | --- | --- | --- | --- | --- | --- | --- |
|  |  |  | **Cases** | **n** | **Cases** | **n** |  |
| Kiondo et al 2014 | Vitamin C | PE (≥ 140/ 90 mm Hg and 2+ proteinuria ≥ 20 weeks gestation) | 13 | 415 | 17 | 418 | 3.1% vs 4.1% |
| McEvoy et al 2014 | Vitamin C | PE | 2 | 83 | 3 | 75 | 2.4% vs 3.9% |
| Steyn et al 2003 | Vitamin C | PE | 3 | 100 | 3 | 100 | 3% vs 3%, |
| Abramovici et al 2015 | Vitamin C and E | PE (≥ 140/ 90 mm Hg and proteinuria >300 mg/24 hours or 2+ ≥ 20 weeks gestation) | 52 | 763 | 62 | 788 | 6.8% vs 7.9% |
| Beazley et al 2005 | Vitamin C and E | PE | 9 | 52 | 9 | 48 | 17.3% vs 18.8% |
| Chappell et al 1999 | Vitamin C and E | PE (≥ 90 mm Hg diastolic and 300mg/24 hr or 2+ proteinuria ≥ 20 weeks gestation) | 11 | 141 | 24 | 142 | 7.8% vs 16.9% |
| Huria et al 2009 | Vitamin C and E | PE | 5 | 107 | 11 | 109 | 4.7% vs 10.1% |
| Kalpdev et al 2011 | Vitamin C and E | Superimposed pre-eclampsia: onset proteinuria ≥300 mg/24 h in hypertensive women with no proteinuria  before 20 weeks’ gestation | 2 | 22 | 3 | 22 | 9.0% vs 13.6% vs |
| McCance et al 2010 | Vitamin C and E | PE (Two readings of ≥ 90 mm Hg diastolic or one of ≥110 mm Hg diastolic and with at least 300mg/24 hr or 1+ proteinuria ≥ 20 weeks gestation) | 57 | 375 | 70 | 374 | 15.2% vs 18.7% |
| Nasrolahi et al 2006 | Vitamin C and E | PE | 5 | 290 | 18 | 290 | 1.7% vs 6.2% |
| Poston et al 2006 | Vitamin C and E | PE (Two readings of ≥ 90 mm Hg diastolic with at least 300mg/24 hr or 2+ proteinuria ≥ 20 weeks gestation) | 181 | 1196 | 187 | 1199 | 15.1% vs 15.6% |
| Roberts et al 2010 | Vitamin C and E | PE (≥ 140/ 90 mm Hg and proteinuria >300 mg/24 hours or 2+ ≥ 20 weeks gestation) | 358 | 4993 | 332 | 4976 | 7.2% vs 6.7% |
| Rumbold et al 2006 | Vitamin C and E | PE (≥ 140/ 90 mm Hg and proteinuria, renal insufficiency, liver disease, neurologic problems, hematologic disturbances or fetal growth restriction ≥ 20 weeks gestation) | 56 | 935 | 47 | 942 | 6.0% vs 5.0% |
| Spinnato II et al 2007 | Vitamin C and E | PE (≥ 140/ 90 mm Hg and proteinuria >300 mg/24 hours or 2+ ≥ 20 weeks gestation) | 49 | 355 | 55 | 352 | 13.8% vs 15.6% |
| Taghriri and Danesh 2007 | Vitamin C and E | PE | 2 | 75 | 11 | 75 | 2.7% vs 14.7% |
| Villar et al 2009 | Vitamin C and E | PE (Two readings of ≥ 90 mm Hg diastolic with at least 300mg/24 hr or 2+ proteinuria ≥ 20 weeks gestation) | 164 | 681 | 157 | 674 | 24.1% vs 23.3% |
| Xu et al 2010 | Vitamin C and E | PE (Two readings of ≥ 90 mm Hg diastolic with at least 300mg/24 hr or 2+ proteinuria ≥ 20 weeks gestation) | 69 | 1167 | 68 | 1196 | 6.0% vs 5.7% |
| Bastani et al 2011 | Vitamin E | PE (≥ 140/ 90 mm Hg and proteinuria >300 mg/24 hours or 1+ ≥ 20 weeks gestation) | 1 | 104 | 3 | 168 | 1.0% vs 1.8% |
| Mahdy et al 2013 | Vitamin E | PE (diastolic blood pressure >90mmHg and proteinuria >300 mg/24 hours≥ 20 weeks gestation) | 1 | 151 | 5 | 148 | 0.7% vs 3.4% |
| Kiondo et al 2014 | Vitamin C | Severe PE (≥ 160 / 110mm Hg and 3+ proteinuria or visual disturbances, headache, epigastric or right upper quadrant pain, pulmonary oedema or cyanosis, abnormal liver function, low platelets, oliguria, fetal growth restriction) | 5 | 415 | 4 | 418 | 1.2% vs 1.0% |
| Roberts et al 2010 | Vitamin C and E | Severe PE (≥ 160/110 mm Hg and proteinuria >500 mg/24 hours or oliguria, pulmonary edema, or thrombocytopenia) | 28 | 763 | 27 | 788 | 3.7% vs 3.4% |
| Beazley et al 2005 | Vitamin C and E | Severe PE | 3 | 52 | 3 | 48 | 5.8% vs 6.3% |
| Chappell et al 1999 | Vitamin C and E | Severe PE (≥ 110 mm Hg diastolic and 300mg/24 hr or 2+ proteinuria ≥ 20 weeks gestation) | 3 | 141 | 5 | 142 | 2.1% vs 3.5% |
| Huria et al 2009 | Vitamin C and E | Severe PE | 2 | 107 | 5 | 109 | 1.9% vs 4.6% |
| Poston et al 2006 | Vitamin C and E | Severe PE (Two readings of ≥ 110 mm Hg diastolic or single reading ≥ 120 mm Hg diastolic with at least 300mg/24 hr or 2+ proteinuria ≥ 20 weeks gestation) | 62 | 1196 | 53 | 1199 | 5.2% vs 4.4% |
| Roberts et al 2010 | Vitamin C and E | Severe PE (≥ 160/110 mm Hg and proteinuria >500 mg/24 hours or oliguria, pulmonary edema, or thrombocytopenia) | 134 | 4993 | 129 | 4976 | 2.7% vs 2.6% |
| Spinnato II et al 2007 | Vitamin C and E | Severe PE (≥ 160/ 110 mm Hg and proteinuria or proteinuria >5g/24 hours with any hypertension, hypertension complicated by pulmonary edema, low platelet count or hemolysis, reported among patients without chronic hypertension) | 11 | 170 | 4 | 168 | 6.5% vs 2.4% |
| Villar et al 2009 | Vitamin C and E | Severe PE (≥ 160/ 110 mm Hg and proteinuria) | 22 | 681 | 29 | 674 | 3.2% vs 4.3% |
| Xu et al 2010 | Vitamin C and E | Severe PE (PE and the presence of at least 1 of the following adverse conditions: (1) ≥ 160/ 110 mm Hg; (2) proteinuria ≥ 5  g/24-hour urine collection or dipstick ≥ 3; (3) convulsion; (4) thrombocytopenia; (5) elevated liver enzyme levels;  (6) hematocrit <24% or blood transfusion; (7) IUGR; (8) perinatal death; or  (9) preterm delivery (<34 weeks of gestational age) | 33 | 1167 | 39 | 1196 | 2.8% vs 3.3% |
| Kiondo et al 2014 | Vitamin C | GH (≥140/90 mm Hg without proteinuria) | 32 | 415 | 48 | 418 | 7.7% vs 11.5% |
| Chappell et al 1999 | Vitamin C and E | GH (≥ 90 mm Hg diastolic and without significant proteinuria) | 16 | 141 | 13 | 142 | 11.3% vs 9.2% |
| McCance et al 2010 | Vitamin C and E | GH (Two readings of ≥ 90 mm Hg diastolic or one of ≥110 mm Hg diastolic) | 42 | 375 | 41 | 374 | 11.2% vs 11.0% |
| Poston et al 2006 | Vitamin C and E | GH (Two readings of ≥ 90 mm Hg diastolic without significant proteinuria) | 84 | 1196 | 55 | 1199 | 7.0% vs 4.6% |
| Rumbold et al 2006 | Vitamin C and E | GH (≥ 140/ 90 mm Hg) | 124 | 935 | 109 | 942 | 13.3% vs 11.6% |
| Villar et al 2009 | Vitamin C and E | GH (Two readings of ≥ 90 mm Hg diastolic without significant proteinuria) | 64 | 681 | 52 | 674 | 9.4% vs 7.7% |
| Xu et al 2010 | Vitamin C and E | GH (Two readings of ≥ 90 mm Hg diastolic ≥ 20 weeks gestation) | 253 | 1167 | 249 | 1196 | 21.7% vs 20.8% |
| Kiondo et al 2014 | Vitamin C | Eclampsia | 0 | 415 | 2 | 418 | 0.0% vs 0.5% |
| Kalpdev et al 2011 | Vitamin C and E | Eclampsia | 0 | 22 | 0 | 22 | NA |
| McCance et al 2010 | Vitamin C and E | Eclampsia | 1 | 375 | 2 | 374 | 0.3% vs 0.5% |
| Poston et al 2006 | Vitamin C and E | Eclampsia | 3 | 1196 | 1 | 1199 | 0.5% vs 0.1% |
| Roberts et al 2010 | Vitamin C and E | Eclampsia | 10 | 4993 | 4 | 4976 | 0.2% vs 0.1% |
| Spinnato II et al 2007 | Vitamin C and E | Eclampsia (Pregnancy induced hypertension and convulsions) | 0 | 355 | 1 | 342 | 0.0% vs 0.3% |
| Villar et al 2009 | Vitamin C and E | Eclampsia (a seizure in a woman with  pre-eclampsia in the absence of a known or subsequently  diagnosed convulsive disorder) | 3 | 681 | 2 | 674 | 0.4% vs 0.3% |
| Xu et al 2010 | Vitamin C and E | Eclampsia | 1 | 1167 | 0 | 1196 | 0.1% vs 0.0% |
| Kalpdev et al 2011 | Vitamin C and E | HELLP syndrome | 0 | 22 | 0 | 22 | NA |
| McCance et al 2010 | Vitamin C and E | HELLP syndrome | 3 | 375 | 2 | 374 | 0.8% vs 0.7% |
| Poston et al 2006 | Vitamin C and E | HELLP syndrome | 6 | 1196 | 1 | 1199 | 0.5% vs 0.1% |
| Roberts et al 2010 | Vitamin C and E | HELLP syndrome (pregnancy-associated hypertension with all of the following: a platelet count of less than 100,000 per  cubic millimeter, an aspartate aminotransferase  level of 100 U per liter or more, and evidence of  hemolysis) | 2 | 4993 | 8 | 4976 | <0.1% vs 0.2% |
| Spinnato II et al 2007 | Vitamin C and E | HELLP syndrome | 2 | 355 | 2 | 352 | 0.6% vs 0.6% |
| Villar et al 2009 | Vitamin C and E | HELLP syndrome | 10 | 681 | 8 | 674 | 1.5% vs 1.2% |
| McEvoy et al 2014 | Vitamin C | SGA (< 10^th^ centile) | 7 | 83 | 10 | 76 | 8.4% vs 13.2% |
| Abramovici et al 2014 | Vitamin C and E | SGA (< 10^th^ centile) | 88 | 734 | 116 | 766 | 12.0% vs 15.1% |
| Beazley et al 2005 | Vitamin C and E | SGA (< 10^th^ centile) | 2 | 52 | 4 | 48 | 3.8% vs 8.3% |
| Chappell et al 1999 | Vitamin C and E | SGA (< 10^th^ centile) | 33 | 141 | 45 | 142 | 23.4% vs 31.7% |
| Huria et al 2009 | Vitamin C and E | SGA (< 10^th^ centile) | 12 | 107 | 13 | 109 | 11.2% vs 11.9% |
| Kalpdev et al 2011 | Vitamin C and E | SGA (birthweight < 1SD for GA) | 3 | 22 | 2 | 22 | 13.6% vs 9.0% |
| McCance et al 2010 | Vitamin C and E | SGA (<10^th^ centile) | 23 | 373 | 36 | 372 | 6.2% vs 9.7% |
| Poston et al 2006 | Vitamin C and E | SGA (< 10^th^ centile) | 403 | 1393 | 360 | 1391 | 28.9% vs 25.9% |
| Roberts et al 2010 | Vitamin C and E | IUGR (< 3^rd^ percentile) | 133 | 4990 | 132 | 4881 | 2.7% vs 2.7% |
| Rumbold et al 2006 | Vitamin C and E | IUGR (< 3^rd^ percentile) | 80 | 924 | 92 | 929 | 8.7% vs 9.9% |
| Spinnato II et al 2007 | Vitamin C and E | SGA (<10^th^ centile) | 49 | 356 | 49 | 352 | 14.0% vs 14.0% |
| Villar et al 2009 | Vitamin C and E | SGA (< 10^th^ centile) | 141 | 592 | 149 | 573 | 23.8% vs 26.0% |
| Xu et al 2010 | Vitamin C and E | SGA (< 10^th^ centile) | 173 | 1243 | 194 | 1293 | 14.0% vs 15.1% |
| Kiondo et al 2014 | Vitamin C | LBW (<2500g) | 46 | 415 | 43 | 418 | 11.1% vs 10.3% |
| Beazley et al 2005 | Vitamin C and E | LBW (<2500g) | 13 | 52 | 12 | 48 | 25.0% vs 25.0% |
| Poston et al 2006 | Vitamin C and E | LBW (<2500g) | 387 | 1393 | 335 | 1391 | 27.8% vs 24.1% |
| Roberts et al 2010 | Vitamin C and E | LBW (<2500g) | 345 | 4900 | 369 | 3244 | 7.0% vs 7.6% |
| Spinnato II et al 2007 | Vitamin C and E | LBW (<2500g) | 61 | 356 | 62 | 352 | 17.4% vs 17.8% |
| Villar et al 2009 | Vitamin C and E | LBW (<2500g) | 250 | 753 | 277 | 762 | 33.2% vs 36.4% |
| Bastani et al 2011 | Vitamin E | LBW (<2500g) | 8 | 104 | 11 | 168 | 7.6% vs 6.5% |
| Casanueva et al 2005 | Vitamin C | PTB | 7 | 52 | 14 | 57 | 13.4% vs 24.0% |
| Kiondo et al 2014 | Vitamin C | PTB (< 37 weeks ) | 47 | 415 | 51 | 418 | 11.3% vs 12.2% |
| McEvoy et al 2014 | Vitamin C | PTB (< 37 weeks ) | 6 | 83 | 10 | 76 | 7.0% vs 13.0% |
| Steyn et al 2003 | Vitamin C | PTB (< 37 weeks) | 50 | 100 | 35 | 100 | 50.0% vs 35.0% |
| Abramovici et al 2015 | Vitamin C and E | PTB (< 37 weeks) | 106 | 763 | 83 | 788 | 13.9% vs 10.5% |
| Beazley et al 2005 | Vitamin C and E | PTB (< 37 weeks) | 20 | 52 | 14 | 48 | 38.4% vs 30.0% |
| Chappell et al 1999 | Vitamin C and E | PTB (< 37 weeks) | 6 | 141 | 5 | 142 | 4.3% vs 3.5% |
| Huria et al 2009 | Vitamin C and E | PTB (< 37 weeks) | 5 | 107 | 17 | 109 | 4.7% vs 15.6% |
| Kalpdev et al 2011 | Vitamin C and E | PTB (< 37 weeks) | 3 | 22 | 10 | 22 | 13.6% vs 45.5% |
| McCance et al 2010 | Vitamin C and E | PTB (< 37 weeks) | 126 | 375 | 152 | 374 | 33.6% vs 40.6% |
| Poston et al 2006 | Vitamin C and E | PTB (≤ 37 weeks) | 400 | 1372 | 373 | 1376 | 29.2% vs 27.1% |
| Roberts et al 2010 | Vitamin C and E | PTB (< 37 weeks) | 513 | 4993 | 526 | 4976 | 10.3% vs 10.6% |
| Rumbold et al 2006 | Vitamin C and E | PTB (< 37 weeks) | 64 | 932 | 63 | 935 | 6.9% vs 6.7% |
| Spinnato II et al 2007 | Vitamin C and E | PTB (< 37 weeks) | 96 | 356 | 82 | 352 | 27.4% vs 23.5% |
| Villar et al 2009 | Vitamin C and E | PTB (< 37 weeks) | 188 | 674 | 213 | 669 | 27.9% vs 31.8% |
| Xu et al 2010 | Vitamin C and E | PTB (< 37 weeks) | 193 | 1243 | 184 | 1293 | 16.6% vs 15.5% |
| Bastani et al 2011 | Vitamin E | PTB | 7 | 104 | 16 | 168 | 6.7% vs 9.5% |
| Kiondo et al 2014 | Vitamin C | Stillbirth (died in uterus after 24 weeks of pregnancy) | 19 | 415 | 19 | 418 | 4.6% vs 4.5% |
| Steyn et al 2003 | Vitamin C | Stillbirth (intrauterine death) | 1 | 100 | 0 | 100 | 1.0% vs 0.0% |
| Chappell et al 1999 | Vitamin C and E | Stillbirth | 1 | 141 | 2 | 142 | 0.7% vs 1.4% |
| McCance et al 2010 | Vitamin C and E | Stillbirth | 9 | 379 | 8 | 382 | 2.4% vs 2.1%, p=1.0 |
| Poston et al 2006 | Vitamin C and E | Stillbirth (intrauterine death >24 weeks’ gestation) | 19 | 1369 | 7 | 1372 | 1.4% vs 0.5% |
| Roberts et al 2010 | Vitamin C and E | Stillbirth (fetal death at ≥20 week) | 38 | 4993 | 36 | 4976 | 0.8% vs 0.7% |
| Rumbold et 2006 | Vitamin C and E | Stillbirth (fetal death ≥20 weeks) | 8 | 935 | 5 | 942 | 0.9% vs 0.6% |
| Spinnato II et al 2007 | Vitamin C and E | Stillbirth | 7 | 356 | 10 | 352 | 2.0% vs 2.8% |
| Xu et al 2010 | Vitamin C and E | Stillbirth | 10 | 1243 | 6 | 1293 | 0.8% vs 0.5% |
| McCance et al 2010 | Vitamin C and E | Maternal mortality | 0 | 379 | 1 | 382 | 0.0% vs 0.3% |
| Poston et al 2006 | Vitamin C and E | Maternal mortality | 1 | 1196 | 1 | 1199 | 0.1% vs 0.1% |
| Roberts et al 2010 | Vitamin C and E | Maternal mortality | 1 | 4993 | 1 | 4993 | <0.1% vs <0.1% |
| Spinnato II et al 2007 | Vitamin C and E | Maternal mortality | 0 | 355 | 0 | 352 | NA |
| Villar et al 2009 | Vitamin C and E | Maternal mortality | 0 | 681 | 1 | 681 | 0.0% vs 0.1% |
| Xu et al 2010 | Vitamin C and E | Maternal mortality | 0 | 1167 | 0 | 1196 | NA |

# Vitamin D and/or calcium

| **Reference** | **Nutrient** | **Outcome** | **Intervention** | | **Control** | | **Comparison** |
| --- | --- | --- | --- | --- | --- | --- | --- |
|  |  |  | **Cases** | **n** | **Cases** | **n** |  |
| Asemi et al 2013 | Vitamin D | PE | 0 | 27 | 1 | 27 | 0% vs 3.7% |
| Hossain et al 2014 | Vitamin D | PE (de novo ≥ 140/ 90 mm Hg and 1+ proteinuria ≥ 20 weeks gestation) | 10 | 100 | 6 | 100 | 10.0% vs 6.0% |
| Jamilian et al 2018 | Vitamin D | PE | 2 | 30 | 3 | 30 | 6.7% vs 10.0% |
| Karamali et al 2015 | Vitamin D | PE (Hypertension and proteinuria ≥ 20 weeks gestation) | 1 | 30 | 3 | 30 | 3.3% vs 10.0% |
| Naghshineh et al 2016 | Vitamin D | PE | 2 | 68 | 7 | 70 | 2.9% vs 10.0% |
| Razavi et al 2017 | Vitamin D (vs placebo) | PE | 3 | 30 | 3 | 30 | 10.0% vs 10.0% |
| Roth et al 2013 | Vitamin D | PE | 0 | 80 | 1 | 80 | 0.0% vs 1.3% |
| Sablok et al 2015 | Vitamin D | PE | 8 | 108 | 8 | 57 | 7.4% vs 14.0% |
| Sasan et al 2017 | Vitamin D | PE (≥ 140/ 90 mm Hg and 1+ proteinuria) | 11 | 70 | 22 | 72 | 15.7% vs 30.6% |
| Valizadeh et al 2016 | Vitamin D | PE | 1 | 42 | 3 | 42 | 2.4% vs 7.1% |
| Yap et al 2014 | Vitamin D | PE | 2 | 89 | 4 | 90 | 2.2% vs 4.4% |
| Zerofsky et al 2014 | Vitamin D | PE | 1 | 25 | 0 | 26 | 4.0% vs 0.0% |
| Marya et al 1987 | Vitamin D and calcium | PE (≥ 140/ 90 mm Hg and proteinuria >300 mg/24 hours) | 12 | 200 | 18 | 200 | 6.0% vs 9.0% |
| Samimi et al 2016 | Vitamin D and calcium | PE | 1 | 30 | 3 | 30 | 3.3% vs 10.0% |
| Taherian et al 2002 | Vitamin D and calcium | PE (increase of 30 mmHg systolic or 15 mmHg diastolic with ≥ 300mg/24 hours proteinuria) | 13 | 330 | 33 | 330 | 4% vs 10.1% |
| Aghamohammadi and Zafari 2015 | Calcium | PE (≥ 140/ 90 mm Hg or increase of 30 mmHg systolic or 15 mmHg diastolic and proteinuria of 30 mg/dl) | 6 | 40 | 14 | 40 | 15% vs 35% |
| Almirante 1998 | Calcium | EPH gestosis | 14 | 212 | 41 | 210 | 6.6% vs 19.5% |
| Bassaw et al 1998 | Calcium | PE (≥ 140/ 90 mm Hg with >1+ proteinuria) | 1 | 81 | 37 | 250 | 1.2% vs 14.8% |
| Belizan et al 1991 | Calcium | PE (≥ 140/ 90 mm Hg and proteinuria 300mg/ltr) | 15 | 579 | 23 | 588 | 2.6% vs 3.9% |
| Cong et al 1995 | Calcium | PE | 0 | 112 | 2 | 56 | 0.0% vs 3.6% |
| Crowther et al 1999 | Calcium | PE ( ≥120 mmHg diastolic on 1 occasion or of ≥110mmHg on 2 occasions 4 hours or more apart with proteinuria ≥300mg/24 hours or 2+) | 10 | 227 | 23 | 229 | 4.4% vs 10.0% |
| Hofymeyr et al 2019 | Calcium | PE ≥ 140/ 90 mm Hg and 2 or more on urine dipstick, or >300 mg/24 h, or >500 mg/L or urinary protein) | 69 | 296 | 82 | 283 | 23% vs 29% |
| Khan et al 2013 | Calcium | PE (≥ 140/ 90 mm Hg and proteinuria 300mg/24 hr) | 7 | 123 | 19 | 139 | 5.7% vs 13.7% |
| Kumar et al 2009 | Calcium | PE (≥ 140/ 90 mm Hg and 2 or more on urine dipstick, or >300 mg/24 h, or 1+ proteinuria) | 11 | 273 | 30 | 251 | 4.0% vs 12.0% |
| Levine et al 1997 | Calcium | PE (pregnancy-associated hypertension ≥90 mm Hg diastolic  and proteinuria >300 mg/24 h, or 1+ proteinuria occurring within seven days) | 158 | 2295 | 168 | 2294 | 6.9% vs 7.3% |
| Lopez-Jaramillo et al 1989 | Calcium | PE (≥ 140/ 90 mm Hg or increase of 30 mmHg systolic or 15 mmHg diastolic (following Hofymeyr et al 2018 for inclusion)) | 2 | 49 | 12 | 43 | 4.1% vs 27.9% |
| Lopez-Jaramillo et al 1990 | Calcium | PE (≥ 140/ 90 mm Hg and proteinuria) | 0 | 22 | 8 | 34 | 0.0% vs 23.5% |
| Lopez-Jaramillo et al 1997 | Calcium | PE (≥ 140/ 90 mm Hg and proteinuria >30 mg/dL) | 4 | 125 | 21 | 135 | 3.2% vs 15.5% |
| Nenad et al 2011 | Calcium | PE | 316 | 4590 | 366 | 4588 | 6.9% vs 7.3% |
| Niromanesh et al 2001 | Calcium | PE (≥ 140/ 90 mm Hg and 1+ proteinuria) | 1 | 15 | 7 | 15 | 6.7% vs 46.7% |
| Purwar et al 1996 | Calcium | PE (≥ 140/ 90 mm Hg and proteinuria ≥ 300mg/24 hr) | 2 | 97 | 11 | 93 | 2.1% vs 11.8% |
| Rogers et al 1999 | Calcium | PE (≥ 140/ 90 mm Hg and 2+ proteinuria) | 8 | 144 | 7 | 75 | 5.6% vs 9.3% |
| Sanchez-Ramos et al 1994 | Calcium | PE (≥ 140/ 90 mm Hg and 2+ or ≥ 300mg/ 24 hr proteinuria) | 4 | 29 | 15 | 34 | 13.8% vs 44.1% |
| Villar and Repke 1990 | Calcium | PE (≥ 140/ 90 mm Hg and ≥ 300mg proteinuria) | 0 | 90 | 3 | 88 | 0.0% vs 3.4% |
| Villar et al 1987 | Calcium | PE (Diastolic BP ≥ 90mmHG (following Hofymeyr et al 2018 for inclusion) | 1 | 25 | 3 | 27 | 4% vs 10% |
| Villar et al 2006 | Calcium | PE (≥ 140/ 90 mm Hg and 2+ or ≥ 300mg/ 24 hr proteinuria) | 171 | 4151 | 186 | 4161 | 4.1% vs 4.5% |
| Wanchu et al 2001 | Calcium | PE (≥ 140/ 90 mm Hg or increase of 30 mmHg systolic or 15 mmHg diastolic over baseline) | 9 | 50 | 8 | 50 | 18.0% vs 16.0% |
| Herrera et al 1998 | Calcium (with linoleic acid) | PE (≥ 140/ 90 mm Hg and ≥ 300mg/24 hr proteinuria) | 4 | 43 | 16 | 43 | 9.3% vs 37.2% |
| Herrera et al 2006 | Calcium (with linoleic acid) | PE (≥ 140/ 90 mm Hg and ≥ 300mg/24 hr proteinuria) | 0 | 24 | 3 | 24 | 0.0% vs 12.5% |
| Naghshineh et al 2016 | Vitamin D | Severe PE | 0 | 68 | 3 | 70 | 0.0% vs 4.3% |
| Asemi et al 2012 | Vitamin D and calcium | Severe PE | 1 | 27 | 1 | 27 | 3.7% vs 3.7% |
| Asemi et al 2016 | Vitamin D and calcium | NA | 0 | 23 | 1 | 23 | 0.0% vs 4.3% |
| Taherian et al 2002 | Vitamin D and calcium | Severe PE (≥160/110 mmHg and 4+ protein by dipstick on two occasions 4 hours apart) | 3 | 330 | 6 | 330 | 0.9% vs 1.8% |
| Crowther et al 1999 | Calcium | Severe PE (severe hypertension  with proteinuria of one 24-hour urine collection of 3g  or more) | 4 | 227 | 6 | 229 | 1.8% vs 2.6% |
| Hofymeyr et al 2019 | Calcium | Severe PE (proteinuria plus severe diastolic  [>110 mm Hg] or systolic [>160 mm Hg] hypertension) | 52 | 296 | 60 | 283 | 18% vs 21% |
| Levine et al 1997 | Calcium | Severe PE (preeclampsia with either severe  pregnancy-associated hypertension or severe pregnancy-associated  proteinuria, or as eclampsia or the HELLP syndrome with or without  proteinuria) | 50 | 2295 | 59 | 2294 | 2.2% vs 2.6% |
| Sanchez-Ramos et al 1994 | Calcium | Severe PE (Pre-eclampsia and ≥ 160/110 mm Hg or ≥ 5g/24 hr proteinuria, oliguria, elevated liver enzymes, thrombocytopenia, pulmonary edema or severe epigastric pain) | 1 | 29 | 6 | 34 | 3.4% vs 17.6% |
| Villar et al 2006 | Calcium | Severe PE (≥ 160/110 mm Hg) | 35 | 4151 | 47 | 4161 | 0.8% vs 1.1% |
| Wanchu et al 2001 | Calcium | Severe PE (≥ 110 mm Hg diastolic and/or persistent proteinuria ≥ 2+) | 0 | 50 | 2 | 50 | 0.0% vs 4.0% |
| Cooper et al 2016 | Vitamin D | GH | 13 | 479 | 15 | 486 | 2.3% vs 2.6% |
| Hossain et al 2014 | Vitamin D | GH (≥140/90 mm Hg without proteinuria) | 11 | 100 | 7 | 100 | 11.0% vs 7.0% |
| Sablok et al 2015* | Vitamin D | GH | 4 | 108 | 4 | 57 | 3.7% vs 7.0% |
| Yap et al 2014 | Vitamin D | GH | 1 | 89 | 2 | 90 | 1.1% vs 2.2% |
| Zerofsky et al 2014 | Vitamin D | GH | 0 | 25 | 2 | 26 | 0% vs 7.7% |
| Li et al 2000 | Vitamin D and calcium | GH | 7 | 58 | 8 | 30 | 17.2% (5 of 29) 600mg Caltrate-D vs 6.9 (2 of 29) 1200 Caltrate-D vs 26.7% (8 of 30) no supplement |
| Bassaw et al 1998 | Calcium | GH (≥ 140/ 90 mm Hg without significant proteinuria) | 0 | 81 | 26 | 250 | 0.0% vs 10.4% |
| Belizan et al 1991 | Calcium | GH (≥ 140/ 90 mm Hg without proteinuria) | 41 | 579 | 63 | 588 | 7.2% vs 10.7% |
| Hofymeyr et al 2019 | Calcium | GH (diastolic blood pressure  >90 mm Hg on two occasions 4 h apart; or >110 mm Hg once, or systolic blood pressure >140 mm Hg on two occasions 4 h apart; or both, or >160 mm Hg once, after 20 weeks)) | 194 | 296 | 197 | 283 | 66% vs 70% |
| Lopez-Jaramillo et al 1990 | Calcium | GH (≥ 140/ 90 mm Hg without proteinuria) | 3 | 22 | 16 | 34 | 14% vs 47.1% |
| Purwar et al 1996 | Calcium | GH (≥ 140/ 90 mm Hg) | 6 | 97 | 16 | 93 | 6.2% vs 17.2% |
| Rogers et al 1999 | Calcium | GH (≥ 140/ 90 mm Hg and <2+ proteinuria) | 21 | 144 | 18 | 75 | 14.6% vs 24.0% |
| Sanchez-Ramos et al 1994 | Calcium | GH (≥ 140/ 90 mm Hg without significant proteinuria) | 5 | 29 | 7 | 34 | 17.2% vs 20.6% |
| Villar and Repke 1990 | Calcium | GH (≥ 140/ 90 mm Hg without proteinuria) | 3 | 90 | 5 | 88 | 3.3% vs 5.7% |
| Villar et al 2006 | Calcium | GH (≥ 140/ 90 mm Hg without proteinuria) | 442 | 4151 | 459 | 4161 | 10.6% vs 11.0% |
| Herrera et al 2006 | Calcium (with linoleic acid) | GH (≥ 140/ 90 mm Hg without proteinuria) | 2 | 24 | 7 | 24 | 8.3% vs 29.2% |
| Cong et al 1995 | Calcium | Eclampsia | 0 | 112 | 1 | 56 | 0.0% vs 1.8% |
| Hofymeyr et al 2019 | Calcium | Eclampsia | 4 | 296 | 5 | 283 | 1% vs 2% |
| Khan et al 2013 | Calcium | Eclampsia (≥ 140/ 90 mm Hg and NA 300mg/24 hr) | 2 | 123 | 7 | 139 | 1.6% vs 5.0% |
| Levine et al 1997 | Calcium | Eclampsia (A woman with pregnancy-associated  hypertension had a seizure without any other known cause) | 4 | 2295 | 4 | 2294 | 0.2% vs 0.2% |
| Purwar et al 1996 | Calcium | Eclampsia | 0 | 97 | 0 | 93 | NA |
| Villar et al 2006 | Calcium | Eclampsia (seizure in a woman with  preeclampsia in the absence of a known or subsequently  diagnosed convulsive disorder) | 17 | 4151 | 25 | 4161 | 0.4% vs 0.6% |
| Hofymeyr et al 2019 | Calcium | HELLP syndrome (haemolysis, elevated liver enzymes, and low platelet count) | 10 | 69 | 7 | 81 | 15% vs 9% |
| Levine et al 1997 | Calcium | HELLP syndrome (pregnancy-associated hypertension with two or more platelet counts of < 100,000 per cubic millimeter, a serum aspartate aminotransferase  concentration  ≥ 70 U per liter, and a serum lactate dehydrogenase  concentration  ≥ 600 U per liter, a serum total bilirubin  concentration  ≥ 1.2 mg per decilite, or a  peripheral-blood smear with nucleated red cells or schistocytes) | 7 | 2295 | 2 | 2294 | 0.3% vs 0.1% |
| Brooke et al 1980 | Vitamin D | SGA (<10^th^ centile) | 9 | 59 | 19 | 67 | 15.3% vs 28.6% |
| Dawodu et al 2013 | Vitamin D | SGA | 5 | 84 | 4 | 42 | 11.9% (5 of 42) in 4000 IU group vs 0% in 2000 IU group vs 9.5% (4 of 42) control |
| Hashemipour et al 2014 | Vitamin D | SGA | 0 | 65 | 1 | 65 | 0.0% vs 1.5% |
| Sablok et al 2015 | Vitamin D | SGA | 9 | 108 | 11 | 57 | 8.3% vs 19.4% |
| Yu et al 2009 | Vitamin D | SGA (<10^th^ centile) | 17 | 120 | 10 | 59 | 15% (9 of 60) vs daily dose vs 13% (8 of 60) single dose vs 17% (10 of 59) no treatment |
| Taherian et al 2002 | Vitamin D and calcium | SGA | 35 | 330 | 39 | 330 | 10.7% vs 11.9% |
| Crowther et al 1999 | Calcium | SGA | 32 | 227 | 27 | 229 | 14.1% vs 11.8% |
| Khan et al 2013 | Calcium | SGA | 4 | 123 | 13 | 139 | 3.3% vs 9.4% |
| Kumar et al 2009 | Calcium | SGA | 17 | 273 | 21 | 251 | 6.2% vs 8.4% |
| Levine et al 1997 | Calcium | SGA (< 10^th^ centile) | 124 | 2295 | 105 | 2294 | 5.8% vs 4.9% |
| Purwar et al 1996 | Calcium | SGA | 6 | 97 | 8 | 93 | 6.2% vs 8.6% |
| Sanchez-Ramos et al 1994 | Calcium | SGA | 2 | 29 | 4 | 34 | 6.9% vs 11.8% |
| Villar and Repke 1990 | Calcium | SGA | 3 | 94 | 3 | 95 | 3.2% vs 3.2% |
| Herrera et al 1998 | Calcium (with linoleic acid) | SGA (<10^th^ centile) | 1 | 43 | 3 | 43 | 2.3% vs 7.0% |
| Herrera et al 2006 | Calcium (with linoleic acid) | SGA (<10^th^ centile) | 1 | 24 | 4 | 24 | 4.1% vs 16.6% |
| Brooke et al 1980 | Vitamin D | LBW (<2500g) | 7 | 59 | 15 | 67 | 11.9% vs 22.4% |
| Hossain et al 2011 | Vitamin D | LBW (<2500g) | 19 | 100 | 18 | 100 | 19.0% vs 18.0% |
| Karamali et al 2015 | Vitamin D | LBW (<2500g) | 0 | 30 | 2 | 30 | 0.0% vs 6.7% |
| Khan et al 2016 | Vitamin D | LBW (<2500g) | 7 | 27 | 11 | 35 | 25.9% vs 31.4% |
| Marya 1988 | Vitamin D | LBW (<2500g) | 4 | 100 | 19 | 100 | 4 vs 19% |
| Roth et al 2013 | Vitamin D | LBW (<2500g) | 12 | 72 | 14 | 72 | 16.7% vs 19.4% |
| Diogenes et al 2013 | Vitamin D and calcium | LBW (<2500g) | 2 | 26 | 1 | 21 | 7.7% vs 4.8% |
| Samimi et al 2016 | Vitamin D and calcium | LBW (<2500g) | 0 | 30 | 2 | 30 | 0.0% vs 6.7% |
| Aghamohammadi and Zafari 2015 | Calcium | LBW | 8 | 40 | 12 | 40 | 20% vs 30% |
| Belizan et al 1991 | Calcium | LBW (<2500g) | 31 | 547 | 41 | 559 | 5.7% vs 7.3% |
| Crowther et al 1999 | Calcium | LBW (<2500g) | 6 | 227 | 17 | 229 | 2.6% vs 7.4% |
| Hofymeyr et al 2019 | Calcium | LBW (<2500g) | 79 | 264 | 73 | 243 | 30% vs 30% |
| Kumar et al 2009 | Calcium | LBW (<2500g) | 19 | 273 | 32 | 251 | 7.0% vs 12.7% |
| Levine et al 1997 | Calcium | LBW (<2500g) | 188 | 2295 | 205 | 2294 | 8.8% vs 9.6% |
| Lopez-Jaramillo et al 1989 | Calcium | LBW | 0 | 49 | 0 | 43 | NA |
| Lopez-Jaramillo et al 1990 | Calcium | LBW (<2500g) | 0 | 125 | 0 | 135 | NA |
| Villar et al 1987 | Calcium | LBW (<2500g) | 0 | 25 | 0 | 27 | NA |
| Herrera et al 1998 | Calcium (with linoleic acid) | LBW (<2500g) | 1 | 43 | 5 | 43 | 2.3% vs11.6% |
| Herrera et al 2006 | Calcium (with linoleic acid) | LBW (<2500g) | 1 | 24 | 5 | 24 | 4.1% vs 16.6% |
| Asemi et al 2013 | Vitamin D | PTB (<37 weeks) | 1 | 27 | 1 | 27 | 3.7% vs 3.7% |
| Chawes et al 2016 | Vitamin D | PTB (<37 weeks) | 11 | 315 | 9 | 308 | 3.5% vs 2.9% |
| Cooper et al 2016 | Vitamin D | PTB (<37 weeks) | 16 | 479 | 10 | 486 | 2.8% vs 1.8% |
| Delvin et al 1986 | Vitamin D | PTB (<37 weeks) | 0 | 15 | 0 | 19 | NA |
| Grant et al 2013 | Vitamin D | PTB (<37 weeks) | 0 | 171 | 4 | 87 | 0% (0 of 87) 1000 IU group vs 0% (0 of 84) 2000 IU group vs 4.6% (4 of 87) placebo |
| Hossain et al 2011 | Vitamin D | PTB (<37 weeks) | 12 | 100 | 10 | 100 | 12.0% vs 10.0 % |
| Jamilian et al 2018 | Vitamin D | PTB (<37 weeks | 0 | 30 | 1 | 30 | 0.0% vs 3.3% |
| Karamali et al 2015 | Vitamin D | PTB (<37 weeks | 0 | 30 | 1 | 30 | 0.0% vs 3.3% |
| Litonjua et al 2016 | Vitamin D | PTB (<37 weeks | 40 | 405 | 31 | 401 | 10% vs 8% |
| Mohammad-Alizadeh-Charandabi et al 2015 | Vitamin D | PTB (<37 weeks | 2 | 42 | 4 | 42 | 4.8% vs 9.5% |
| Naghshineh et al 2016 | Vitamin D | PTB | 4 | 68 | 17 | 70 | 6.0% vs 24.3% |
| Razavi et al 2017 | Vitamin D | PTB (<37 weeks | 0 | 30 | 1 | 30 | 0.0% vs 3.3% |
| Roth et al 2013 | Vitamin D | PTB (<37 weeks) | 9 | 74 | 13 | 75 | 12.2% vs 17.3% |
| Sablok et al 2015 | Vitamin D | PTB | 9 | 108 | 12 | 57 | 8.3% vs 21.1% |
| Singh et al 2015 | Vitamin D | PTB (<37 weeks) | 6 | 50 | 15 | 50 | 12.0% vs 30.0% |
| Valizadeh et al 2016 | Vitamin D | PTB | 3 | 42 | 5 | 42 | 7.1% vs 11.9% |
| Yap et al 2014 | Vitamin D | PTB | 4 | 89 | 7 | 90 | 4.5% vs 7.7% |
| Asemi et al 2012 | Vitamin D and calcium | PTB (<37 weeks) | 1 | 27 | 0 | 27 | 3.7% vs 0.0% |
| Asemi et al 2016 | Vitamin D and calcium | PTB | 1 | 23 | 0 | 23 | 4.3% vs 0.0% |
| Diogenes et al 2013 | Vitamin D and calcium | PTB (<37 weeks) | 0 | 43 | 0 | 41 | NA |
| Mohammad-Alizadeh-Charandabi et al 2015 (  Mirghafourvand et al 2013) | Vitamin D and calcium | PTB (<37 weeks) | 4 | 42 | 4 | 42 | 9.5% vs 9.5% |
| Samimi et al 2016 | Vitamin D and calcium | PTB (<37 weeks) | 2 | 30 | 1 | 30 | 6.7% vs 3.3% |
| Taherian et al 2002 | Vitamin D and calcium | PTB (<37 weeks) | 45 | 330 | 29 | 330 | 13.7% vs 8.9% |
| Aghamohammadi and Zafari 2015 | Calcium | PTB | 6 | 40 | 9 | 40 | 15% vs 22% |
| Almirante 1998 | Calcium | PTB | 12 | 212 | 30 | 210 | 5.6% vs 14.3% |
| Belizan et al 1991 | Calcium | PTB (<37 weeks) | 33 | 527 | 37 | 542 | 6.3% vs 6.8% |
| Boggess et al 1997 | Calcium | PTB (<37 weeks ) | 0 | 12 | 3 | 11 | 0.0% vs 27.3% |
| Crowther 1999 | Calcium | PTB (<37 weeks) | 10 | 227 | 23 | 229 | 4.4% vs 10.0% |
| Hofymeyr et al 2019 | Calcium | PTB (<37 weeks) | 112 | 296 | 119 | 283 | 38% vs 42% |
| Khan et al 2013 | Calcium | PTB | 3 | 123 | 11 | 139 | 2.4% vs 7.9% |
| Kumar et al 2009 | Calcium | PTB | 19 | 273 | 32 | 251 | 7.0% vs 12.7% |
| Levine et al 1997 | Calcium | PTB (<37 weeks) | 248 | 2295 | 229 | 2294 | 10.8% vs 10.0% |
| Lopez-Jaramillo et al 1989 | Calcium | PTB (<36 weeks) | 0 | 49 | 0 | 43 | NA |
| Lopez-Jaramillo et al 1990 | Calcium | PTB (<36 weeks) | 0 | 125 | 0 | 135 | NA |
| Purwar et al 1996 | Calcium | PTB | 2 | 97 | 6 | 93 | 2.1% vs 6.5% |
| Sanchez-Ramos et al 1994 | Calcium | PTB | 5 | 29 | 8 | 34 | 17.2% vs 23.5% |
| Villar and Repke 1990 | Calcium | PTB (<37 weeks) | 7 | 94 | 20 | 95 | 7.4% vs 21.1% |
| Villar et al 1987 | Calcium | PTB | 0 | 25 | 0 | 27 | NA |
| Villar et al 2006 | Calcium | PTB (<37 weeks) | 398 | 4038 | 436 | 1042 | 9.8% vs 10.8% |
| Wanchu et al 2001 | Calcium | PTB (<37 weeks) | 3 | 50 | 3 | 50 | 6.0% vs 6.0% |
| Herrera et al 2006 | Calcium (with linoleic acid) | PTB | 1 | 24 | 2 | 24 | 4.1% vs 8.3% |
| Grant et al 2013 | Vitamin D | Stillbirth | 0 | 171 | 1 | 87 | 0.0% vs 0.1% |
| Roth et al 2013 | Vitamin D | Stillbirth | 1 | 80 | 1 | 80 | 1.3% vs 1.3% |
| Valizadeh et al 2016 | Vitamin D | Stillbirth | 1 | 42 | 1 | 42 | 2.4% vs 2.4% |
| Yap et al 2014 | Vitamin D | Stillbirth | 2 | 89 | 2 | 90 | 2.2% vs 2.2% |
| Yu et al 2009 | Vitamin D | Stillbirth | 0 | 120 | 1 | 59 | 0% vs 1.7% |
| Goldberg et al 2013 | Calcium | Stillbirth | 9 | 330 | 11 | 332 | 2.7% vs 3.3% |
| Hofymeyr et al 2019 | Calcium | Stillbirth | 2 | 296 | 33 | 283 | 9% vs 12% |
| Khan et al 2013 | Calcium | Stillbirth | 3 | 123 | 7 | 139 | 2.4% vs 5.0% |
| Kumar et al 2009 | Calcium | Stillbirth | 6 | 273 | 5 | 251 | 2.2% vs 2.0% |
| Lopez-Jaramillo et al 1989* | Calcium | Stillbirth | 0 | 49 | 0 | 43 | NA |
| Purwar et al 1996 | Calcium | Stillbirth | 0 | 97 | 0 | 93 | NA |
| Villar et al 2006 | Calcium | Stillbirth | 105 | 4181 | 113 | 4197 | 2.5% vs 2.7% |
| Sablok et al 2015 | Vitamin D | Maternal mortality (death while pregnant or within 42 days of termination of pregnancy) | 0 | 120 | 0 | 60 | NA |
| Goldberg et al 2013 | Calcium | Maternal mortality | 1 | 330 | 1 | 332 | 0.3% vs 0.3% |
| Hofymeyr et al 2019 | Calcium | Maternal mortality | 2 | 323 | 0 | 310 | 1% vs 0% |
| Lopez-Jaramillo et al 1990 | Calcium | Maternal mortality | 0 | 125 | 0 | 135 | NA |
| Purwar et al 1996 | Calcium | Maternal mortality | 0 | 97 | 0 | 93 | NA |
| Villar et al 2006 | Calcium | Maternal mortality | 1 | 4151 | 6 | 4161 | 0.02% vs 0.14% |

# Iron and/or folic acid

| **Reference** | **Nutrient** | **Outcome** | **Intervention** | | **Control** | | **Comparison** |
| --- | --- | --- | --- | --- | --- | --- | --- |
|  |  |  | **Cases** | **n** | **Cases** | **n** |  |
| Eskeland et al 1997 | Iron (vs placebo) | PE | 1 | 24 | 1 | 23 | 4.2% vs 4.3% |
| Ouladsah-  ebmadarek et al 2011 | Iron (vs placebo) | PE | 16 | 410 | 10 | 372 | 3.9% vs 2.7%, p=0.42 |
| Charles et al 2005 | Folic acid (vs placebo) | PE (diastolic >90 mmHg on two separate occasions, with or without oedema, with albuminuria exceeding 0.25 g/L) | 15 | 907 | 51 | 1890 | 1.8% (8/448) 200 mcg folic acid vs 1.5% (7/459) 5 mg folic acid vs 2.7% (51/1890) placebo |
| Fleming et al 1968 | Folic acid (vs placebo) | PE (pre-eclamptic toxaemia | 2 | 27 | 2 | 26 | 7.4% vs 7.7% |
| Taylor et al 1982 | Iron-folic acid (vs no treatment) | PE | 1 | 24 | 0 | 24 | 4.2% vs 0.0% |
| Ziaei et al 2007 | Iron-folic acid (vs placebo and folic acid) | PE (hypertension disorder-included as Pena-Rosas et al 2015) | 10 | 370 | 3 | 357 | 2.7% vs 0.8% |
| Christian et al 2003 | Folic acid (vs control) | Eclampsia | 9 | 625 | 19 | 683 | 1.4% vs 2.8% |
| Christian et al 2003 | Iron-folic acid (vs folic acid) | Eclampsia | 11 | 639 | 9 | 625 | 1.7% vs 1.4% |
| Chan et al 2009 | Iron (vs placebo) | SGA (<10^th^ centile) | 15 | 419 | 33 | 443 | 3.6%, vs 7.5% |
| Cogswell et al 2003 | Iron (vs placebo) | SGA (<10^th^ centile) | 8 | 117 | 17 | 96 | 6.8% vs 17.7% |
| Ouladsahebmadarek et al 2011 | Iron (vs placebo) | SGA (<10^th^ centile) | 58 | 410 | 65 | 372 | 14.1% vs 17.5% |
| Siega-Riz et al 2001 | Iron (vs placebo) | SGA (<10^th^ centile) | 18 | 166 | 26 | 168 | 10.8% vs 15.5% |
| Christian et al 2003 | Folic acid (alone vs no folic acid) | SGA (<10^th^ centile) | 363 | 628 | 402 | 685 | 57.8% vs 8.7% |
| Christian et al 2003 | Iron-folic acid (vs folic acid) | SGA (<10^th^ centile) | 327 | 633 | 363 | 628 | 51.7% vs 7.8% |
| Zeng et al 2008 | Iron-folic acid (vs folic acid) | SGA (<10^th^ centile) | 278 | 1470 | 280 | 1545 | 18.9% vs 18.1% |
| Ziaei et al 2007 | Iron-folic acid | SGA | 57 | 370 | 36 | 357 | 15.4% vs 10.1% |
| Cogswell et al 2003 | Iron (vs placebo) | LBW (<2500g) | 5 | 117 | 16 | 96 | 4.3% vs 16.7% |
| Eskeland et al 1997 | Iron (vs placebo) | LBW (<2500g) | 0 | 28 | 0 | 26 | NA |
| Makrides et al 2003 | Iron (vs placebo) | LBW (<2500g) | 12 | 216 | 9 | 214 | 5.4% vs 4.2% |
| Siega-Riz et al 2001 | Iron (vs placebo) | LBW (<2500g) | 8 | 173 | 16 | 172 | 4.8% vs 9.5% |
| Charles et al 2005 | Folic acid (vs placebo) | LBW (<2500g) | 47 | 907 | 115 | 1890 | 6.1% (27/448) 200 mcg folic acid vs 4.3% (20 /459) vs 6.1% (115/ 1890) placebo |
| Christian et al 2003 | Folic acid (alone vs no folic acid) | LBW (<2500g) | 262 | 628 | 297 | 685 | 41.7% vs 3.4% |
| Fleming et al 1968 | Folic acid (vs placebo) | LBW (<2500g) | 3 | 27 | 3 | 26 | 11.1% vs 11.5% |
| Christian et al 2003 | Iron-folic acid (vs folic acid) | LBW (<2500g) | 218 | 635 | 262 | 628 | 34.3% vs 41.7% |
| Liu et al 2013 | Iron-folic acid (vs folic acid) | LBW (<2500g) | 129 | 6252 | 125 | 6261 | 2.2% vs 2.1% |
| Meier et al 2003 | Iron-folic acid (vs placebo and folic acid | LBW (<2500g) | 1 | 58 | 2 | 52 | 5.4 vs 2.9% |
| Mendendez et al 1994 | Iron-folic acid (vs placebo and folic acid | LBW (<2500g) | 7 | 231 | 12 | 219 | 3.0% vs 5.5% |
| Ndyomugyenyi and Magnussen 2000 | Iron-folic acid (vs placebo) | LBW (<2500g) | 11 | 174 | 15 | 168 | 6.3% vs 8.9% |
| Taylor et al 1982 | Iron-folic acid (vs no treatment) | LBW (<2500g) | 2 | 24 | 0 | 24 | 8.3% vs 0.0% |
| Zeng et al 2008 | Iron-folic acid (vs folic acid) | LBW (<2500g) | 66 | 1470 | 82 | 1545 | 4.5% vs 5.3% |
| Chan et al 2009 | Iron (vs placebo) | PTB (<37 weeks) | 27 | 419 | 30 | 443 | 6.4%, vs 6.8% |
| Cogswell et al 2003 | Iron (vs placebo) | PTB (<37 weeks) | 15 | 117 | 12 | 96 | 12.8% vs 12.5% |
| Eskeland et al 1997 | Iron (vs placebo) | PTB (<37 weeks) | 0 | 24 | 1 | 23 | 0.0% vs 4.3% |
| Harvey et al 2007 | Iron (vs placebo) | PTB (<37 weeks) | 0 | 6 | 0 | 7 | NA |
| Liu et al 2000 | Iron (vs no treatment) | PTB (<37 weeks) | 3 | 200 | 2 | 100 | 1.5% vs 2.0% |
| Ouladsahebmadarek et al 2011 | Iron (vs placebo) | PTB (delivery  between 20-38 w of gestation from the date of LMP) | 16 | 410 | 18 | 372 | 3.9% vs 4.8% |
| Siega-Riz et al 2001 | Iron (vs placebo) | PTB (<37 weeks) | 13 | 173 | 24 | 172 | 7.5% vs 13.9% |
| Charles et al 2005 | Folic acid (vs placebo) | PTB (<37 weeks) | 39 | 907 | 75 | 1890 | 4.9% (22/448) 200 mcg folic acid vs 3.7% (17459) vs 4.0% (75/1890) placebo |
| Christian et al 2003 | Folic acid (alone vs no folic acid) | PTB (<37 weeks) | 139 | 628 | 140 | 685 | 22.1% vs 20.4% |
| Fleming et al 1968 | Folic acid (vs placebo) | PTB (36-38 weeks gestation) | 7 | 27 | 9 | 26 | 25.9% vs 34.6% |
| Christian et al 2003 | Iron-folic acid (vs folic acid alone) | PTB (<37 weeks) | 146 | 633 | 139 | 628 | 23.1% vs 22.1% |
| Lee et al 2005 | Iron-folic acid (vs no supplement) | PTB (<37 weeks) | 0 | 111 | 0 | 20 | NA |
| Liu et al 2013 | Iron-folic acid (vs folic acid) | PTB (<37 weeks) | 340 | 6252 | 353 | 6261 | 5.7% vs 6.0% |
| Taylor et al 1982 | Iron-folic acid (vs no treatment) | PTB (<37 weeks) | 3 | 24 | 0 | 24 | 12.5% vs 0.0% |
| Zeng et al 2008 | Iron-folic acid (vs folic acid) | PTB (<37 weeks) | 76 | 1470 | 102 | 1545 | 4.9% vs 6.1% |
| Ziaei et al 2007 | Iron-folic acid (vs placebo and folic acid) | PTB | 17 | 370 | 13 | 357 | 4.6% vs 3.6% |
| Makrides et al 2003 | Iron (vs placebo) | Stillbirth | 1 | 216 | 1 | 214 | 0.5% vs 0.5% |
| Fleming et al 1968 | Folic acid (vs placebo) | Stillbirth | 0 | 27 | 1 | 26 | 0.0% vs 3.8% |
| Kirke et al 1992 | Folic acid (vs multiple micronutrient without folic acid) | Stillbirth | 0 | 115 | 2 | 119 | 0.0% vs 2.1% |
| MRC 1991 | Folic acid (vs iron-calcium) | Stillbirth | 2 | 298 | 3 | 300 | 0.7% vs 1.0% |
| Liu et al 2013 | Iron-folic acid (vs folic acid) | Stillbirth (death from 28 weeks of gestation to delivery) | 28 | 5954 | 28 | 5934 | 4.7% vs 4.7% |
| Mendendez et al 1994 | Iron-folic acid (vs placebo and folic acid) | Stillbirth | 8 | 273 | 12 | 277 | 2.9% vs 4.3% |
| Zeng et al 2008 | Iron-folic acid (vs folic acid) | Stillbirth (fetuses  delivered at 28 weeks’ gestation or later with no signs of life) | 47 | 1584 | 56 | 1722 | 3.0% vs 3.3% |
| Lee et al 2005 | Iron-folic acid (vs no supplement) | Maternal morality | 0 | 111 | 0 | 20 | NA |

# Zinc

| **Reference** | **Nutrient** | **Outcome** | **Intervention** | | **Control** | | **Comparison** |
| --- | --- | --- | --- | --- | --- | --- | --- |
|  |  |  | **Cases** | **n** | **Cases** | **n** |  |
| Castillo-Duran et al 2001 | Zinc | PE | 2 | 249 | 2 | 258 | 0.8% vs 0.8% |
| Danesh et al 2010 | Zinc | PE | 0 | 42 | 2 | 42 | 0.0% vs 4.8% |
| Jonsson et al 1996 | Zinc | PE | 20 | 585 | 22 | 621 | 3.4% vs 3.5% |
| Mahomed et al 1989 | Zinc | PE (≥90 mm Hg diastolic and more than trace proteinuria on two separate occasions) | 11 | 241 | 3 | 238 | 4.6% vs 0.9% |
| Merialdi et al 2004 | Zinc | PE | 5 | 121 | 1 | 121 | 4.1% vs 0.8% |
| Merialdi et al 2004 | Zinc | GH | 4 | 121 | 0 | 121 | 3.3% vs 0.0% |
| Christian et al 2003 | Zinc | SGA (<10^th^ centile) | 378 | 670 | 327 | 633 | 56.4% vs51.7% |
| Danesh et al 2010 | Zinc | IUGR (no further definition) | 0 | 42 | 1 | 42 | 0.0% vs 2.4% |
| Goldenberg et al 1995 | Zinc | SGA (<10^th^ centile) | 16 | 294 | 18 | 286 | 5.4% vs 6.3% |
| Jonsson et al 1996 | Zinc | SGA (<10^th^ centile) | 37 | 585 | 38 | 621 | 6.3% vs 6.1% |
| Osendarp et al 2000 | Zinc | SGA (<10^th^ centile) | 145 | 194 | 161 | 216 | 74.7% vs 74.5% |
| Saaka et al 2009 | Zinc | SGA (<10^th^ centile) | 14 | 272 | 18 | 271 | 5.1% vs 6.6% |
| Simmer et al 1991 | Zinc | SGA | 2 | 30 | 6 | 22 | 6.7% vs 27.3% |
| Xie et al 2001 | Zinc | SGA | 7 | 116 | 6 | 40 | 6.0% vs 15.0% |
| Castillo-Duran 2001 | Zinc | LBW (<2500g) | 6 | 249 | 16 | 258 | 2.4% vs 6.2% |
| Caulfield 1999 | Zinc | LBW (<2500g) | 18 | 521 | 18 | 495 | 3.5% vs 3.6% |
| Christian et al 2003 | Zinc | LBW (<2500g) | 265 | 672 | 218 | 635 | 39.4% vs 34.3% |
| Danesh 2010 | Zinc | LBW (<2500g) | 4 | 42 | 8 | 42 | 9.5% vs 19.0% |
| Goldenberg 1995 | Zinc | LBW (<2500g) | 23 | 294 | 36 | 286 | 7.8% vs 12.7% |
| Hafeez, Mehmood and Mazhar 2005 | Zinc | LBW (<2500g) | 15 | 121 | 11 | 121 | 12.0% vs 9.1% |
| Hunt 1984 | Zinc | LBW | 4 | 87 | 4 | 90 | 4.6% vs 4.4% |
| Mahomed et al 1989 | Zinc | LBW (<2500g) | 15 | 247 | 13 | 244 | 6.1% vs 5.3% |
| Osendarp 2000 | Zinc | LBW (<2500g) | 89 | 194 | 87 | 216 | 45.9% vs 40.3% |
| Robertson, Heywood and Atkinson 1991 | Zinc | LBW (<2500g) | 15 | 72 | 17 | 62 | 20.8% vs 27.4% |
| Saaka et al 2009 | Zinc | LBW (<2500g) | 26 | 272 | 21 | 271 | 9.6% vs 7.7% |
| Simmer et al 1991 | Zinc | LBW | 5 | 30 | 5 | 24 | 16.7% vs 20.8% |
| Xie et al 2001 | Zinc | LBW | 9 | 116 | 9 | 40 | 7.8% vs 22.5% |
| Castillo-Duran 2001 | Zinc | PTB (≤36 weeks) | 14 | 249 | 30 | 258 | 5.6% vs 11.6% |
| Caulfield 1999 | Zinc | PTB (<37 weeks) | 29 | 521 | 30 | 495 | 5.5% vs 6.1% |
| Cherry 1989 | Zinc | PTB | 50 | 268 | 68 | 288 | 18.7% vs 25.6% |
| Christian et al 2003 | Zinc | PTB (<37 weeks) | 127 | 628 | 137 | 593 | 20.2% vs 23.1% |
| Danesh 2010 | Zinc | PTB (<37 weeks) | 9 | 42 | 14 | 42 | 21.4% vs 33.3% |
| Dijkhuizen 2004 | Zinc | PTB | 2 | 44 | 3 | 41 | 4.5% vs 7.3% |
| Goldenberg 1995 | Zinc | PTB (<37 weeks) | 30 | 294 | 38 | 286 | 10.2% vs 13.3% |
| Hafeez 2005 | Zinc | PTB (<37 weeks) | 22 | 121 | 10 | 121 | 17.8% vs 8.1% |
| Hunt 1984 | Zinc | PTB | 5 | 87 | 4 | 90 | 5.7% vs 4.4% |
| Jonsson 1996 | Zinc | PTB | 33 | 585 | 49 | 621 | 5.6% vs 7.9% |
| Mahomed 1989 | Zinc | PTB (<37 weeks) | 10 | 243 | 17 | 243 | 4.1% vs 7.0% |
| Merialdi 2004 | Zinc | PTB | 7 | 121 | 5 | 121 | 5.8% vs 4.1% |
| Osendarp 2000 | Zinc | PTB (<37 weeks) | 34 | 194 | 34 | 216 | 17.5% vs 15.7% |
| Saaka 2009 | Zinc | PTB (<37 weeks) | 40 | 272 | 39 | 271 | 14.7% vs 14.4% |
| Simmer 1991 | Zinc | PTB | 2 | 30 | 1 | 22 | 6.7% vs 4.5% |
| Xie 2001 | Zinc | PTB | 10 | 116 | 7 | 40 | 8.6% vs 17.5% |
| Merialdi et al 2004 | Zinc | Stillbirths | 0 | 121 | 2 | 121 | 0.0% vs 1.7% |
| Robertson, Heywood and Atkinson 1991 | Zinc | Stillbirths | 0 | 72 | 0 | 62 | NA |
| Dijkhuizen et al 2004 | Zinc | Maternal mortality | 0 | 44 | 1 | 41 | 0.0% vs 2.4% |

# Multiple micronutrients

| **Reference** | **Nutrient** | **Outcome** | **Intervention** | | **Control** | | **Comparison** |
| --- | --- | --- | --- | --- | --- | --- | --- |
|  |  |  | **Cases** | **n** | **Cases** | **n** |  |
| Rumiris et al 2006 | Multiple micronutrients supplement (vs iron-folic acid) | PE (≥ 140/ 90 mm Hg and 2+ or ≥ 300mg/24 hr proteinuria) | 2 | 29 | 9 | 31 | 6.9% vs 29.0% |
| Vadillo-Ortega et al 2011 | Multiple micronutrients supplement (vs no multiple micronutrients) | PE (≥ 140/ 90 mm Hg and ≥ 300mg/24 hr proteinuria, with or without eclampsia) | 29 | 228 | 67 | 222 | 12.7% vs 30.1% |
| Rumiris et al 2006 | Multiple micronutrient supplement (vs iron-folic acid) | Severe PE | 1 | 29 | 3 | 31 | 3.4% vs 9.7% |
| Fawzi et al 1998/ Merchant et al 2005 | Multiple micronutrient supplement (vs placebo) | GH (≥110/90 mmHg anytime during pregnancy-included as in Rumbold et al 2008) | 35 | 485 | 54 | 470 | 7.2% vs 11.5% |
| Gupta et al 2007 | Multiple micronutrient supplement (vs iron-folic acid) | GH (Pregnancy-induced hypertension after 20 weeks) | 2 | 88 | 3 | 82 | 2.3% vs 3.7% |
| Christian et al 2003/ Christian et al 2009 | Multiple micronutrients (vs iron-folic acid) | Eclampsia | 18 | 717 | 11 | 639 | 1.8% vs 1.7% |
| Adu-Afarwuah et al 2015 (Dewey et al 2009) | Multiple micronutrients supplement (vs iron-folic acid) | SGA (<10^th^ centile) | 99 | 317 | 109 | 304 | 31.2% vs 35.9% |
| Ashorn et al 2015 (Ashorn et al 2010) | Multiple micronutrients supplement (vs iron-folic acid) | SGA (<10^th^ centile) | 109 | 379 | 117 | 385 | 28.8% vs 30.4% |
| Brough et al 2010 | Multiple micronutrient supplement (vs placebo) | SGA (<10^th^ centile) | 30 | 179 | 31 | 174 | 16.8% vs 17.8% |
| Christian et al 2003 | Multiple micronutrient (vs iron-folic acid) | SGA (<10^th^ centile) | 379 | 704 | 327 | 633 | 53.8% vs 51.7% |
| Fawzi et al 1998 | Multiple micronutrient (compared to no multiple micronutrient) | SGA (<10^th^ centile) | 39 | 512 | 66 | 509 | 10.0% vs 17.6% |
| Fawzi et al 2007 | Multiple micronutrient supplement (vs iron-folic acid) | SGA (<10^th^ centile) | 407 | 3803 | 523 | 3845 | 10.7% vs 13.6% |
| Friis et al 2004 | Multiple micronutrient supplement (vs iron-folic acid) | IUGR-LBW: gestational age > 37 weeks and birth weight <2500g) | 28 | 564 | 38 | 542 | 5.0% vs 7.0% |
| Gupta et al 2007 | Multiple micronutrient supplement (vs iron-folic acid) | SGA | 23 | 74 | 37 | 72 | 31.1% vs 51.4% |
| Johnson et al 2017 (Moore et al 2009) | Multiple micronutrient supplement (vs iron-folic acid) | SGA (<10^th^ centile) | 52 | 164 | 52 | 146 | 31.7% vs 35.6% |
| Kæstel et al 2005 | Multiple micronutrient supplement (vs iron-folic acid) | SGA (<10^th^ centile) | 24 | 279 | 33 | 291 | 8.6% vs 11.3% |
| Osrin et al 2005 | Multiple micronutrient supplement (vs iron-folic acid) | SGA (<10^th^ centile) | 42 | 480 | 55 | 483 | 8.8% vs 11.4% |
| Ramakrishan et al 2003 | Multiple micronutrient supplement (vs iron) | SGA (<10^th^ centile) | 32 | 317 | 37 | 314 | 10.1% vs 11.8% |
| Roberfroid et al 2008 | Multiple micronutrient supplement (vs iron-folic acid) | SGA (<10^th^ centile) | 194 | 518 | 213 | 512 | 37.4% vs 41.6% |
| Rumiris et al 2006 | Multiple micronutrient (vs iron-folic acid) | SGA | 1 | 29 | 1 | 31 | 3.4% vs 3.2% |
| West et al 2014 | Multiple micronutrient supplement (vs iron-folic acid) | SGA (<10^th^ centile) | 6405 | 10 161 | 6479 | 10 099 | 63.0% vs 64.2% |
| Zeng et al 2008 | Multiple micronutrient (vs iron-folic acid) | SGA (<10^th^ centile) | 238 | 1406 | 278 | 1470 | 16.9% vs 18.9% |
| Adu-Afarwuah et al 2015 | Multiple micronutrients supplement (vs iron-folic acid) | LBW (<2500g) | 32 | 318 | 44 | 305 | 10.1% vs 14.4% |
| Ashorn et al 2015 | Multiple micronutrients supplement (vs iron-folic acid) | LBW (<2500g) | 51 | 379 | 49 | 385 | 13.5% vs 12.7% |
| Bhutta et al 2009 | Multiple micronutrient supplement (vs iron-folic acid) | LBW | 203 | 1148 | 241 | 1230 | 17.7% vs 19.6% |
| Brough et al 2010 | Multiple micronutrient supplement (vs placebo) | LBW (<2500g) | 13 | 179 | 8 | 174 | 7.3% vs 4.6% |
| Christian et al 2003 | Multiple micronutrient (vs iron-folic acid) | LBW (<2500g) | 249 | 705 | 218 | 635 | 35.3% vs 34.3% |
| Fawzi et al 1998 | Multiple micronutrient (compared to no multiple micronutrient) | LBW (<2500g) | 36 | 512 | 62 | 509 | 8.8% vs 15.8% |
| Fawzi et al 2007 | Multiple micronutrient supplement (vs iron-folic acid) | LBW (<2500g) | 306 | 3923 | 368 | 3914 | 7.8% vs 9.4% |
| Friis et al 2004 | Multiple micronutrient supplement (vs iron-folic acid) | LBW (<2500g) | 54 | 564 | 62 | 542 | 9.6% vs 11.4% |
| Gupta et al 2007 | Multiple micronutrient supplement (vs iron-folic acid) | LBW (<2500g) | 12 | 74 | 31 | 72 | 16.2% vs 43.1% |
| Johnson et al 2017 | Multiple micronutrient supplement (vs iron-folic acid) | LBW (<2500g) | 16 | 164 | 18 | 146 | 9.8% vs 12.3% |
| Kæstel et al 2005 | Multiple micronutrient supplement (vs iron-folic acid) | LBW (<2500g) | 44 | 367 | 51 | 373 | 12.0% vs 13.6% |
| Liu et al 2013 | Multiple micronutrient (vs iron-folic acid) | LBW (<2500g) | 116 | 6262 | 129 | 6252 | 2.0% vs 2.2% |
| Osrin et al 2005 | Multiple micronutrient supplement (vs iron-folic acid) | LBW (<2500g) | 101 | 529 | 133 | 523 | 19.1% vs 25.4% |
| Ramakrishan et al 2003 | Multiple micronutrient supplement (vs iron-folic acid) | LBW (<2500g) | 27 | 318 | 28 | 315 | 8.5%vs 8.9% |
| Roberfroid et al 2008 | Multiple micronutrient supplement (vs iron-folic acid) | LBW (<2500g) | 77 | 526 | 82 | 526 | 14.6% vs 15.6% |
| Shankar et al 2008 | Multiple micronutrient supplement (vs iron-folic acid) | LBW (<2500g) | 510 | 5695 | 567 | 5406 | 9.0% vs 10.5% |
| Sunawang et al 2009 | Multiple micronutrient supplement (vs iron-folic acid) | LBW (<2500g) | 24 | 384 | 25 | 341 | 6.3% vs 7.3% |
| West et al 2014 | Multiple micronutrient supplement (vs iron-folic acid) | LBW (<2500g) | 4275 | 10 642 | 4809 | 10 530 | 40.2% vs 45.7% |
| Zagre et al 2007 | Multiple micronutrient supplement (vs iron-folic acid) | LBW (<2500g) | 96 | 1328 | 103 | 1222 | 7.2% vs 8.4% |
| Zeng et al 2008 | Multiple micronutrient (vs iron-folic acid) | LBW (<2500g) | 57 | 1406 | 66 | 1470 | 4.1% vs 4.5% |
| Adu-Afarwuah et al 2015 | Multiple micronutrients supplement (vs iron-folic acid) | PTB (<37 weeks) | 19 | 318 | 28 | 305 | 6.0% vs 9.2% |
| Ashorn et al 2015 | Multiple micronutrients supplement (vs iron-folic acid) | PTB (<37 weeks) | 41 | 433 | 4 | 434 | 9.5% vs 11.3% |
| Brough et al 2010 | Multiple micronutrient supplement (vs placebo) | PTB (<37 weeks) | 9 | 179 | 8 | 174 | 5.0% vs 4.6% |
| Christian et al 2003 | Multiple micronutrient (vs iron-folic acid) | PTB (<37 weeks) | 145 | 704 | 146 | 633 | 20.6% vs 23.1% |
| Fawzi et al 1998 | Multiple micronutrient (vs no multiple micronutrient) | PTB (<37 weeks) | 96 | 512 | 106 | 509 | 21.1% vs 24.5% |
| Fawzi et al 2007 | Multiple micronutrient supplement (vs iron-folic acid) | PTB (<37 weeks) | 676 | 4000 | 666 | 3988 | 16.9% vs 16.7% |
| Friis et al 2004 | Multiple micronutrient supplement (vs iron-folic acid) | PTB (<37 weeks) | 85 | 564 | 99 | 542 | 15.1% vs 18.3% |
| Johnson et al 2017 | Multiple micronutrient supplement (vs iron-folic acid) | PTB (<37 weeks) | 3 | 164 | 4 | 146 | 1.8% vs 2.7% |
| Liu et al 2013 | Multiple micronutrient (vs iron-folic acid) | PTB (<37 weeks) | 308 | 6262 | 340 | 6252 | 5.2% vs 5.7% |
| Osrin et al 2005 | Multiple micronutrient supplement (vs iron-folic acid) | PTB (<37 weeks) | 47 | 571 | 54 | 568 | 8.2% vs 9.5% |
| Ramakrishan et al 2003 | Multiple micronutrient supplement (vs iron) | PTB (<37 weeks) | 24 | 321 | 21 | 321 | 7.5% vs 6.5% |
| Roberfroid et al 2008 | Multiple micronutrient supplement (vs iron-folic acid) | PTB (<37 weeks) | 86 | 607 | 81 | 604 | 14.2% vs 13.4% |
| Rumiris et al 2006 | Multiple micronutrient (vs iron-folic acid) | PTB (<37 weeks) | 1 | 29 | 3 | 31 | 3.4% vs 9.7% |
| Shankar et al 2008 | Multiple micronutrient supplement (vs iron-folic acid) | PTB (<37 weeks) | 3736 | 14373 | 3654 | 14053 | 26.0% vs 26.0% |
| Vadillo-Ortega et al 2011 | Multiple micronutrients supplement (vs no multiple micronutrients) | PTB (<37 weeks) | 24 | 228 | 44 | 222 | 10.5% vs 19.8% |
| West et al 2014 | Multiple micronutrient supplement (vs iron-folic acid) | PTB (<37 weeks) | 2510 | 13 475 | 2912 | 13 333 | 18.6% vs 21.8% |
| Zeng et al 2008 | Multiple micronutrient (vs iron-folic acid) | PTB (<37 weeks) | 78 | 1406 | 76 | 1470 | 5.2% vs 4.9% |
| Adu-Afarwuah et al 2015 | Multiple micronutrients supplement (vs iron-folic acid) | Stillbirth | 7 | 354 | 9 | 349 | 0.2% vs 2.6% |
| Ashorn et al 2015 | Multiple micronutrients supplement (vs iron-folic acid) | Stillbirth | 2 | 433 | 7 | 434 | 0.5% vs 1.6% |
| Bhutta et al 2009 | Multiple micronutrient supplement (vs iron-folic acid) | Stillbirth (death ≥28 weeks) | 50 | 743 | 48 | 832 | 6.7% vs 5.8% |
| Fawzi et al 1998 | Multiple micronutrient (vs no multiple micronutrient) | Stillbirths | 18 | 512 | 31 | 509 | 3.5% vs 6.1% |
| Fawzi et al 2007 | Multiple micronutrient supplement (vs iron-folic acid) | Stillbirth (death between 28 weeks and delivery) | 134 | 4187 | 163 | 4179 | 3.2% vs 3.9% |
| Friis et al 2004 | Multiple micronutrient supplement (vs iron-folic acid) | Stillbirth | 4 | 571 | 7 | 538 | 0.7% vs 1.3%, |
| ICMR 2000 | Multiple micronutrient (vs iron-calcium) | Stillbirth | 3 | 137 | 3 | 142 | 2.2% vs 2.1% |
| Kæstel et al 2005 | Multiple micronutrient supplement (vs iron-folic acid) | Stillbirth | 30 | 547 | 19 | 544 | 5.5% vs 3.5% |
| Kirke et al 1992 | Multiple micronutrients (vs folic acid alone) | Stillbirth | 0 | 120 | 0 | 115 | NA |
| Liu et al 2013 | Multiple micronutrient (vs iron-folic acid) | Stillbirth (death from 28 weeks of gestation to delivery) | 26 | 5942 | 28 | 5954 | 4.3% vs 4.7%, |
| MRC 1991 | Multiple micronutrient (vs folic acid) | Stillbirth | 2 | 295 | 2 | 298 | 0.7% vs 0.7% |
| Osrin et al 2005 | Multiple micronutrient supplement (vs iron-folic acid) | Stillbirth | 15 | 571 | 18 | 568 | 26.6% vs 31.7% |
| Ramakrishan et al 2003 | Multiple micronutrient supplement (vs iron) | Stillbirth | 5 | 309 | 4 | 302 | 1.6% vs 1.3% |
| Roberfroid et al 2008 | Multiple micronutrient supplement (vs iron-folic acid) | Stillbirth (delivery of an infant showing no sign of life after a gestational age of 28 w | 18 | 632 | 8 | 628 | 2.8% vs 1.3% |
| Shankar et al 2008 | Multiple micronutrient supplement (vs iron-folic acid) | Stillbirth (spontaneous death of a fetus after 28 weeks of gestational age and  occurring in utero before labour or during labour itself and before complete expulsion  or extraction) | 245 | 14618 | 268 | 14321 | 1.7% vs 1.9% |
| Sunawang et al 2009 | Multiple micronutrient supplement (vs iron-folic acid) | Stillbirth (fetal loss from 28  weeks or later up to birth without sign of life) | 8 | 432 | 8 | 411 | 1.9% vs 2.0% |
| Tofail et al 2008 / Persson et al 2012 | Multiple micronutrient supplement (vs iron-folic acid) | Stillbirth (birth of a dead fetus after 28 weeks gestation) | 27 | 1480 | 62 | 2965 | 1.8% vs 2.1% |
| West et al 2014 | Multiple micronutrient supplement (vs iron-folic acid) | Stillbirth | 620 | 22405 | 693 | 22162 | 2.8% vs 3.1% |
| Zagre et al 2007 | Multiple micronutrient supplement (vs iron-folic acid) | Stillbirth | 57 | 1521 | 44 | 1381 | 3.7% vs 3.2% |
| Zeng et al 2008 | Multiple micronutrient (vs iron-folic acid) | Stillbirth (fetuses  delivered at 28 weeks’ gestation or later with no signs of life) | 64 | 1545 | 47 | 1584 | 4.1% vs 3.0% |
| Adu-Afarwuah et al 2015 | Multiple micronutrients supplement (vs iron-folic acid) | Maternal mortality | 1 | 354 | 1 | 349 | 0.3% vs 0.3% |
| Ashorn et al 2015 | Multiple micronutrients supplement (vs iron-folic acid) | Maternal mortality | 4 | 465 | 3 | 460 | 0.9% vs 0.7% |
| Kæstel et al 2005 | Multiple micronutrient supplement (vs iron-folic acid) | Maternal mortality | 7 | 520 | 4 | 526 | 1.3% vs 0.8% |
| Shankar et al 2008 | Multiple micronutrient supplement (vs iron-folic acid) | Maternal mortality (deaths related to pregnancy up to 12 weeks post partum) | 41 | 14229 | 39 | 14046 | 0.3% vs 0.3% |
| Tofail et al 2008 / Persson et al 2012 | Multiple micronutrient supplement (vs iron-folic acid) | Maternal mortality | 0 | 1480 | 1 | 2965 | 0.0% vs 0.03% |
| Vadillo-Ortega et al 2011 | Multiple micronutrients supplement (vs no multiple micronutrients) | Maternal mortality | 0 | 228 | 0 | 222 | NA |
| West et al 2014 | Multiple micronutrient supplement (vs iron-folic acid) | Maternal mortality (died during pregnancy) | 10 | 22405 | 6 | 22162 | 0.04% vs 0.03% |
| Zagre et al 2007 | Multiple micronutrient supplement (vs iron-folic acid) | Maternal mortality | 4 | 1893 | 3 | 1777 | 0.2% vs 0.2% |

# Lipid-based nutrients

| **Reference** | **Nutrient** | **Outcome** | **Intervention** | | **Control** | | **Comparison** |
| --- | --- | --- | --- | --- | --- | --- | --- |
|  |  |  | **Cases** | **n** | **Cases** | **n** |  |
| Adu-Afarwuah et al 2015 | Lipid-based nutrient supplement (vs iron-folic acid) | SGA (<10^th^ centile) | 92 | 305 | 109 | 304 | 30.2% vs 35.9% |
| Ashorn et al 2015 | Lipid-based nutrient supplement (vs iron-folic acid) | SGA (<10^th^ centile) | 112 | 380 | 117 | 385 | 29.5% vs 30.4% |
| Johnson et al 2017 | Lipid-based nutrient supplement (vs iron-folic acid) | SGA (<10^th^ centile) | 51 | 159 | 52 | 146 | 32.1% vs 35.6% |
| Mridha et al 2016 | Lipid-based nutrient supplement (vs iron-folic acid) | SGA (<10^th^ centile) | 568 | 898 | 1515 | 2251 | 63.3% vs 67.3% |
| Adu-Afarwuah et al 2015 | Lipid-based nutrient supplement (vs iron-folic acid) | LBW (<2500g) | 27 | 307 | 44 | 305 | 8.8% vs 14.4% |
| Ashorn et al 2015 | Lipid-based nutrient supplement (vs iron-folic acid) | LBW (<2500g) | 46 | 380 | 49 | 385 | 12.1% vs 12.7% |
| Huybregts et al 2009 | Lipid-based nutrient supplement (vs MMN tablet) | LBW (<2500g) | 67 | 523 | 67 | 497 | 12.8% vs 13.5% |
| Johnson et al 2017 | Lipid-based nutrient supplement (vs iron-folic acid) | LBW (<2500g) | 15 | 159 | 18 | 146 | 9.4% vs 12.3% |
| Mridha et al 2016 | Lipid-based nutrient supplement (vs iron-folic acid) | LBW (<2500g) | 323 | 898 | 889 | 2251 | 36.0% vs 39.5% |
| Adu-Afarwuah et al 2015 | Lipid-based nutrient supplement (vs iron-folic acid) | PTB (<37 weeks) | 29 | 308 | 28 | 305 | 9.4% vs 9.2% |
| Ashorn et al 2015 | Lipid-based nutrient supplement (vs iron-folic acid) | PTB (<37 weeks) | 39 | 428 | 49 | 434 | 9.1% vs 11.3% |
| Huybregts et al 2009 | Lipid-based nutrient supplement (vs MMN tablet) | PTB (<37 weeks) | 92 | 576 | 79 | 567 | 16.0% vs 13.9% |
| Johnson et al 2017 | Lipid-based nutrient supplement (vs iron-folic acid) | PTB (<37 weeks) | 4 | 159 | 4 | 146 | 2.5% vs 2.7% |
| Mridha et al 2016 | Lipid-based nutrient supplement (vs iron-folic acid) | PTB (<37 weeks) | 118 | 898 | 308 | 2251 | 13.1% vs 13.7% |
| Adu-Afarwuah et al 2015 | Lipid-based nutrient supplement (vs iron-folic acid) | Stillbirth | 3 | 354 | 9 | 349 | 0.8% vs 2.6% |
| Ashorn et al 2015 | Lipid-based nutrient supplement (vs iron-folic acid) | Stillbirth | 14 | 428 | 7 | 434 | 3.3% vs 1.6%, |
| Huybregts et al 2009 | Lipid-based nutrient supplement (vs MMN tablet) | Stillbirth (delivery of an infant showing no  sign of life after 28 wks gestation) | 12 | 606 | 15 | 596 | 2.0% vs 2.5% |
| Mridha et al 2016 | Lipid-based nutrient supplement (vs iron-folic acid) | Stillbirth | 34 | 1047 | 71 | 2964 | 3.2% vs 2.4% |
| Adu-Afarwuah et al 2015 | Lipid-based nutrient supplement (vs iron-folic acid) | Maternal mortality | 1 | 354 | 1 | 349 | 0.3% vs 0.3% |
| Ashorn et al 2015 | Lipid-based nutrient supplement (vs iron-folic acid) | Maternal mortality | 1 | 454 | 3 | 460 | 0.2% vs 0.7%, |
| Mridha et al 2016 | Lipid-based nutrient supplement (vs iron-folic acid) | Maternal mortality | 0 | 1047 | 2 | 2964 | 0.0% vs 0.07% |

# Polyunsaturated omega-3 fatty acid

| **Reference** | **Nutrient** | **Outcome** | **Intervention** | | **Control** | | **Comparison** |
| --- | --- | --- | --- | --- | --- | --- | --- |
|  |  |  | **Cases** | **n** | **Cases** | **n** |  |
| Bisgaard et al 2016 | Omega 3 | PE | 16 | 365 | 15 | 371 | 4.4% vs 4.3% |
| Bulstra-Ramakers et al 1994 | Omega 3 | PE (increase in diastolic blood pressure of at least 25 mmHg, with a final diastolic pressure >90mmHg with proteinuria >0.5g/24 hr) | 5 | 32 | 3 | 31 | 15.6% vs 9.7% |
| Carlson et al 2013 | Omega 3 | PE | 2 | 154 | 2 | 147 | 1.3% vs 1.4%, |
| Harris et al 2015 | Omega 3 | PE | 2 | 224 | 1 | 121 | 0.9% vs 0.8% |
| Horvaticek et al 2017 | Omega 3 | PE | 4 | 43 | 5 | 38 | 9.3% vs 13.2% |
| Jamilian et al 2016a | Omega 3 | PE | 0 | 27 | 1 | 27 | 0.0% vs 3.7% |
| Lalooha et al 2012 | Omega 3 | PE | 2 | 50 | 10 | 50 | 4.0% vs 20.0% |
| Makrides et al 2010 / Zhao et al 2012 | Omega 3 | PE (presence of pregnancy induced hypertension and proteinuria) | 60 | 1197 | 58 | 1202 | 5.0% vs 4.8% |
| Mardones et al 2008 | Omega 3 | PE | 8 | 493 | 16 | 477 | 1.6% vs 3.4% |
| Mozurkewich et al 2013 | Omega 3 | PE (reported as pregnancy hypertension with proteinuria in Middleton et al 2018) | 10 | 77 | 5 | 41 | 13.0% vs 12.2% |
| Olsen et al 1992/ Salvig et al 1996 | Omega 3 | PE (>140/90 mmHg on two occasions with proteinuria in late pregnancy) | 0 | 266 | 4 | 136 | 0.0% vs 2.9% |
| Olsen et al 2000 (previous PIH) | Omega 3 | PE (>90mmHg diastolic and proteinuria > 1+ or 0.3g/24 hours) | 11 | 152 | 17 | 169 | 7.2% vs 10.1% |
| Olsen et al 2000 (twins) | Omega 3 | PE (>90mmHg diastolic and proteinuria > 1+ or 0.3g/24 hour) | 14 | 246 | 6 | 251 | 5.7% vs 2.4% |
| Onwude et al 1995 | Omega 3 | PE (>90mmHg diastolic and proteinuria > 2+) | 15 | 113 | 18 | 119 | 13.3% vs 15.1% |
| Razavi et al 2017 | Omega 3 (vs placebo) | PE | 2 | 30 | 3 | 30 | 6.7% vs 10.0% |
| Smuts et al 2003a | Omega 3 | PE | 5 | 142 | 10 | 149 | 3.5% vs 6.7% |
| Smuts et al 2003b | Omega 3 | PE | 1 | 18 | 0 | 19 | 5.6% vs 0.0% |
| D’Almedia et al 1992 | Omega 3 and omega 6 | PE (> 30 /15mmHg rise with oedema and proteinuria) | 2 | 50 | 5 | 50 | 4.0% vs 10.0% |
| Jamilian et al 2016b | Omega 3 and vitamin E | PE | 2 | 29 | 1 | 30 | 6.9% vs 3.3% |
| Lalooha et al 2012 | Omega 3 | Severe PE | 0 | 50 | 6 | 50 | 0.0% vs 12.0% |
| Olsen 1992 | Omega 3 (vs placebo) | GH (>140/90 mmHg on two occasions in late pregnancy) | 8 | 266 | 5 | 136 | 3.0% vs 3.7% |
| Onwude et al 1995 | Omega 3 | GH (>90mmHg diastolic without proteinuria -chronic hypertension excluded) | 38 | 113 | 35 | 119 | 33.6% vs 29.4% |
| D’Almedia et al 1992 | Omega 3 and omega 6 | Eclampsia | 0 | 50 | 3 | 50 | 0.0% vs 6.0% |
| Bulstra-Ramakers et al 1994 | Omega 3 | SGA (<10^th^ centile) | 12 | 32 | 9 | 31 | 37.5% vs 29.0% |
| Harper et al 2010 | Omega 3 | SGA (<10^th^ centile) | 35 | 427 | 41 | 410 | 8.2% vs 10.0% |
| Makrides et al 2010 / Zhao et al 2012 | Omega 3 | SGA (<10^th^ centile) | 73 | 1197 | 82 | 1202 | 6.1% vs 6.8% |
| Mardones et al 2008 | Omega 3 | SGA | 30 | 493 | 39 | 477 | 6.1% vs 8.2% |
| Olsen et al 2000 (previous IUGR) | Omega 3 | SGA (<10^th^ centile) | 43 | 131 | 37 | 132 | 32.8% vs 28.0% |
| Olsen et al 2000 (twins) | Omega 3 | SGA (<10^th^ centile) | 165 | 554 | 148 | 557 | 29.8% vs 26.6% |
| Onwude et al 1995 | Omega 3 | SGA (<10^th^ centile) | 33 | 113 | 35 | 119 | 29.2% vs 29.4% |
| Ramakrishnam et al 2010 | Omega 3 | SGA (<10^th^ centile) | 55 | 487 | 53 | 486 | 11.3% vs 10.9% |
| Jamilian et al 2016b | Omega 3 and vitamin E | SGA (<10^th^ centile) | 2 | 29 | 1 | 30 | 6.9% vs 3.3% |
| Bulstra-Ramakers et al 1994 | Omega 3 | LBW (<2500g) | 11 | 32 | 9 | 31 | 34.4% vs 29.0% |
| Carlson et al 2013 | Omega 3 | LBW (<2500g) | 6 | 154 | 13 | 147 | 3.9% vs 8.8% |
| Harper et al 2010 | Omega 3 | LBW (<2500g) | 94 | 427 | 112 | 410 | 22.0% vs 27.3% |
| Makrides et al 2010 / Zhao et al 2012 | Omega 3 | LBW (<2500g) | 41 | 1197 | 63 | 1202 | 3.4% vs 5.2% |
| Mardones et al 2008 | Omega 3 | LBW (<2500g) | 27 | 493 | 37 | 477 | 5.5% vs 7.8% |
| Min et al 2014 (uncomplicated pregnancies) | Omega 3 | LBW (<2500g) | 4 | 32 | 3 | 27 | 12.5% vs 11.1% |
| Min et al 2014 (type 2 diabetes) | Omega 3 | LBW (<2500g) | 4 | 28 | 5 | 30 | 14.3% vs 16.7% |
| Min et al 2016 | Omega 3 | LBW (<2500g) | 8 | 58 | 4 | 56 | 13.8% vs 7.1% |
| Olsen et al 2000 (previous LBW) | Omega 3 | LBW (<2500g) | 15 | 108 | 26 | 118 | 13.9% vs 22.0% |
| Olsen et al 2000 (twins) | Omega 3 | LBW (<2500g) | 238 | 556 | 242 | 556 | 42.8% vs 42.8% |
| Ostadrahimi et al 2017 | Omega 3 | LBW (<2500g) | 0 | 75 | 5 | 75 | 0.0% vs 6.7% |
| Ramakrishnan et al 2010 | Omega 3 | LBW (<2500g) | 27 | 487 | 27 | 486 | 5.5% vs 5.6% |
| Smuts et al 2003a | Omega 3 | LBW (<2500g) | 13 | 142 | 16 | 149 | 9.2% vs 10.7% |
| Smuts et al 2003b | Omega 3 | LBW (<2500g) | 0 | 18 | 5 | 19 | 0.0% vs 26.3% |
| Tofail et al 2006/ Tofail et al 2012 | Omega 3 | LBW (<2500g) | 36 | 125 | 30 | 125 | 28.8% vs 24.0% |
| D’Almedia et al 1992 | Omega 3 and omega 6 | LBW (<2500g) | 2 | 50 | 5 | 50 | 4.0% vs 10.0% |
| Bergmann et al 2007 | Omega 3 | PTB (<37 weeks) | 3 | 48 | 2 | 96 | 6.3% vs 2.1% |
| Bisgaard et al 2016 | Omega 3 | PTB (<37 weeks) | 12 | 346 | 16 | 349 | 3.5% vs 4.6% |
| Bulstra-Ramakers et al 1994 | Omega 3 | PTB (<37 weeks) | 8 | 32 | 10 | 31 | 25.0% vs 32.3% |
| Carlson et al 2013 | Omega 3 | PTB (<37 weeks) | 12 | 154 | 13 | 147 | 7.8% vs 8.8% |
| Dilli et al 2018 | Omega 3 | PTB (<37 weeks) | 6 | 52 | 9 | 68 | 11.5% vs 13.2% |
| Dunstan et al 2003 | Omega 3 | PTB (<36 weeks) | 3 | 40 | 1 | 43 | 7.5% vs 2.3% |
| Harper et al 2010 | Omega 3 | PTB (<37 weeks) | 164 | 434 | 174 | 418 | 37.8% vs 41.6% |
| Harris et al 2015 | Omega 3 | PTB (<34 weeks) | 4 | 224 | 7 | 121 | 1.8% vs 5.8% |
| Hauner et al 2012 | Omega 3 | PTB (<37 weeks) | 3 | 92 | 4 | 96 | 3.3% vs 4.2% |
| Helland et al 2001 | Omega 3 | PTB (<37 weeks) | 1 | 301 | 2 | 289 | 0.3% vs 0.7% |
| Horvaticek et al 2017 | Omega 3 | PTB (<37 weeks) | 4 | 51 | 4 | 47 | 7.8% vs 8.5% |
| Lalooha et al 2012 | Omega 3 | PTB (<37 weeks) | 0 | 60 | 1 | 30 | 0.0% vs 3.3% |
| Makrides et al 2010 / Zhao et al 2012 | Omega 3 | PTB (<37 weeks) | 67 | 1197 | 88 | 1202 | 5.6% vs 7.3% |
| Malcolm et al 2003 | Omega 3 | PTB (<36 weeks) | 0 | 31 | 1 | 32 | 0.0% vs 3.1% |
| Mardones et al 2008 | Omega 3 | PTB (<37 weeks) | 22 | 493 | 32 | 477 | 4.5% vs 6.7% |
| Miller et al 2016 | Omega 3 | PTB (<37 weeks) | 3 | 60 | 10 | 55 | 5.0% vs 18.2% |
| Min et al 2014 (uncomplicated pregnancies) | Omega 3 | PTB (<37 weeks) | 3 | 32 | 3 | 27 | 9.4% vs 11.1% |
| Min et al 2014 (type-2 diabetes) | Omega 3 | PTB (<37 weeks) | 5 | 28 | 12 | 30 | 17.9% vs 40.0% |
| Min et al 2016 | Omega 3 | PTB (<37 weeks) | 12 | 58 | 5 | 56 | 20.7% vs 8.9% |
| Olsen et al 1992 | Omega 3 | PTB (<37 weeks) | 9 | 266 | 9 | 136 | 3.4% vs 6.6% |
| Olsen et al 2000 (previous PTB) | Omega 3 | PTB (<37 weeks) | 5 | 108 | 16 | 120 | 4.6% vs 13.3% |
| Olsen et al 2000 (twins) | Omega 3 | PTB (<37 weeks) | 37 | 286 | 44 | 283 | 12.9% vs 15.5% |
| Onwude et al 1995 | Omega 3 | PTB (<37 weeks) | 22 | 113 | 19 | 119 | 19.5% vs 16.0% |
| Ramakrishnam et al 2010 | Omega 3 | PTB (<37 weeks) | 49 | 487 | 40 | 486 | 10.1% vs 8.2% |
| Razavi et al 2017 | Omega 3 | PTB (<37 weeks) | 0 | 30 | 1 | 30 | 0.0% vs 3.3% |
| Smuts et al 2003a | Omega 3 | PTB (<37 weeks) | 14 | 142 | 17 | 149 | 9.9% vs 11.4% |
| Smuts et al 2003b | Omega 3 | PTB (<37 weeks) | 1 | 18 | 5 | 19 | 5.6% vs 26.3% |
| Tofail et al 2006/ Tofail et al 2012 | Omega 3 | PTB (<37 weeks) | 30 | 125 | 27 | 124 | 24.0% vs 21.8% |
| Van Goor et al 2010 | Omega 3 and omega 6 | PTB (<37 weeks) | 0 | 38 | 3 | 39 | 0.0% vs 7.7% |
| Jamilian et al 2016b | Omega 3 and vitamin E | PTB (<37 weeks) | 3 | 29 | 2 | 30 | 10.3% vs 6.7% |
| Bulstra-Ramakers et al 1994 | Omega 3 | Stillbirth | 1 | 32 | 0 | 31 | 3.1% vs 0.0% |
| Haghiac et al 2015 | Omega 3 | Stillbirth | 1 | 25 | 0 | 25 | 4.0% vs 0.0% |
| Helland et al 2001 | Omega 3 | Stillbirth | 1 | 301 | 0 | 289 | 0.3% vs 0.0% |
| Horvaticek et al 2017 | Omega 3 | Stillbirth (2^nd^ trimester fetal demise) | 1 | 48 | 0 | 50 | 2.1% vs 0.0% |
| Makrides et al 2010 / Zhao et al 2012 | Omega 3 | Stillbirth | 1 | 1197 | 7 | 1202 | 0.1% vs 0.6% |
| Min et al 2014 (uncomplicated pregnancies) | Omega 3 | Stillbirth | 0 | 32 | 0 | 27 | NA |
| Min et al 2014 (type-2 diabetes) | Omega 3 | Stillbirth | 2 | 28 | 0 | 30 | 7.1% vs 0.0% |
| Min et al 2016 | Omega 3 | Stillbirth | 0 | 58 | 1 | 57 | 0.0% vs 1.8% |
| Olsen et al 1992 | Omega 3 | Stillbirth | 1 | 226 | 0 | 136 | 0.4% vs 0.0% |
| Olsen et al 2000 (all) | Omega 3 | Stillbirth | 16 | 1056 | 19 | 1085 | 1.5% vs 1.8% |
| Onwude et al 1995 | Omega 3 | Stillbirth | 0 | 113 | 2 | 119 | 0.0% vs 1.7% |
| Ramakrishnan et al 2010 | Omega 3 | Stillbirth (fetuses delivered at 28 weeks or later with  no signs of life and recorded as occurring before or during the onset of labor) | 2 | 489 | 3 | 488 | 0.4% vs 0.6% |
| Tofail et al 2006/ Tofail et al 2012 | Omega 3 | Stillbirth | 8 | 159 | 6 | 165 | 5.0% vs 3.6% |
| De Groot et al 2003 | Omega 3 and omega 6 | Stillbirth | 0 | 40 | 1 | 39 | 0.0% vs 2.6% |
| Bisgaard et al 2016 | Omega 3 | Maternal mortality | 0 | 365 | 0 | 371 | NA |
| Makrides et al 2010 | Omega 3 | Maternal mortality | 0 | 1197 | 0 | 1202 | NA |
| Olsen et al 2000 (all) | Omega 3 | Maternal mortality | 0 | 818 | 0 | 829 | NA |

# Antenatal dietary counselling with or without physical activity promotion

| **Reference** | **Nutrient** | | **Outcome** | | **Intervention** | | | **Control** | | | **Comparison** | |
| --- | --- | --- | --- | --- | --- | --- | --- | --- | --- | --- | --- | --- |
|  |  |  |  |  | **Cases** | **n** | | **Cases** | | **n** |  |  |
| Bogaerts et al 2012 | Diet and nutrition counselling | | PE (≥140/90 mm hg after 20 weeks with proteinuria ≥300mg/24hr) | | 2 | 76 | | 4 | | 63 | 2.7% vs 6.3% | |
| Crowther et al 2005 | Diet and nutrition counselling | | PE (a blood pressure of at least 140/90 mm Hg on two occasions) | | 141 | 490 | | 139 | | 510 | 28.8% vs 27.3% | |
| Harris et al 2015 | Diet and nutritional education | | PE | | 0 | 191 | | 1 | | 121 | 0.0% vs 0.8% | |
| Khoury et al 2005 | Diet and nutrition counselling | | PE (≥140/90 mm hg with proteinuria after 20 weeks gestation -included as in Allen et al 2014) | | 13 | 141 | | 14 | | 149 | 9.2% vs 9.4% | |
| Landon et al 2009 | Diet and nutrition counselling | | PE (≥140/90 mm hg with proteinuria, elevated liver enzyme levels, and/or thrombocytopenia) | | 12 | 476 | | 25 | | 455 | 2.5% vs 5.5% | |
| Peccei et al 2017 | Diet and nutrition counselling | | PE | | 5 | 180 | | 0 | | 92 | 2.8% vs 0.0% | |
| Thornton et al 2009 | Diet and nutrition counselling | | PE | | 7 | 116 | | 11 | | 116 | 6.0% vs 9.5% | |
| Wolff et al 2008 | Diet and nutrition counselling | | PE | | 0 | 23 | | 1 | | 27 | 0.0% vs 3.7% | |
| Dodd et al 2014 | | Diet and nutrition counselling with physical exercise | | PE | 56 | 1080 | 53 | | 1073 | | | 5.2% vs 4.9% |
| Guelinckx et al 2010 | | Diet and nutrition counselling with physical exercise | | PE (≥140/90 mm hg with proteinuria) | 2 | 42 | 1 | | 43 | | | 4.8% vs 2.3% |
| Luoto et al 2011 | Diet and nutrition counselling with physical exercise | | PE (As reported on medical birth registry) | | 14 | 216 | | 10 | | 179 | 6.5% vs 5.6% | |
| Phelan et al 2011 (normal weight) | Diet and nutrition counselling with physical exercise | | PE | | 3 | 90 | | 9 | | 92 | 3.3% vs 9.8% | |
| Phelan et al 2011 (overweight) | Diet and nutrition counselling with physical exercise | | PE | | 17 | 81 | | 11 | | 86 | 21.0% vs 12.8% | |
| Polley et al 2002 (normal weight) | Diet and nutrition counselling with physical exercise | | PE | | 0 | 30 | | 0 | | 31 | NA | |
| Polley et al 2002 (overweight) | Diet and nutrition counselling with physical exercise | | PE | | 2 | 27 | | 3 | | 22 | 7.4% vs 13.6% | |
| Poston et al 2015 | Diet and nutrition counselling with physical exercise | | PE (≥140/90 mmHg with proteinuria ≥2+ or ≥300mg/24hr) | | 27 | 753 | | 27 | | 752 | 3.6% vs 3.6% | |
| Renault et al 2014 | Diet and nutrition counselling with physical exercise | | PE (>140/90 mmHg on >1 occasion >20 weeks’ gestation plus proteinuria) | | 2 | 130 | | 3 | | 134 | 1.5% vs 2.2% | |
| Poston et al 2015 | Diet and nutrition counselling with physical exercise | | Severe PE (≥170/100 mmHg with proteinuria ≥3+ or ≥500mg/ 24hr) | | 6 | 753 | | 10 | | 752 | 0.8% vs 1.3% | |
| Bogaerts et al 2012 | Diet and nutrition counselling | | GH (≥140/90 mm hg after 20 weeks gestation in an otherwise normotensive woman) | | 8 | 76 | | 6 | | 63 | 10.8% vs 9.5% | |
| Landon et al 2009 | Diet and nutrition counselling | | GH (≥140/90 mm hg) | | 29 | 476 | | 37 | | 455 | 6.1% vs 8.1% | |
| Peccei et al 2017 | Diet and nutrition counselling | | GH | | 3 | 180 | | 2 | | 92 | 1.7% vs 2.2% | |
| Thornton et al 2009 | Diet and nutrition counselling | | GH | | 3 | 116 | | 10 | | 116 | 2.6% vs 8.6% | |
| Wolff et al 2008 | Diet and nutrition counselling | | GH | | 1 | 23 | | 4 | | 27 | 4.3% vs 14.8% | |
| Bruno et al 2016 | Diet and nutrition counselling with physical exercise | | GH | | 2 | 69 | | 13 | | 62 | 2.9% vs 21.0% | |
| Dodd et al 2014 | Diet and nutrition counselling with physical exercise | | GH (≥140/90 mm hg) | | 101 | 1080 | | 94 | | 1073 | 9.4% vs 8.8% | |
| Guelinckx et al 2010 | Diet and nutrition counselling with physical exercise | | GH (≥140/90 mm hg after 20 weeks) | | 18 | 42 | | 14 | | 43 | 43.9% vs 34.1% | |
| Phelan et al 2011 (normal weight) | Diet and nutrition counselling with physical exercise | | GH | | 3 | 90 | | 11 | | 92 | 3.3% vs 12.0% | |
| Phelan et al 2011 (overweight) | Diet and nutrition counselling with physical exercise | | GH | | 17 | 81 | | 11 | | 86 | 21.0% vs 12.8% | |
| Polley et al 2002 (normal weight) | Diet and nutrition counselling with physical exercise | | GH | | 2 | 30 | | 4 | | 31 | 6.7% vs 12.9% | |
| Polley et al 2002 (overweight) | Diet and nutrition counselling with physical exercise | | GH | | 1 | 27 | | 2 | | 22 | 3.7% vs 9.1% | |
| Renault et al 2014 | Diet and nutrition counselling with physical exercise | | GH (BP >140/90 mmHg on >1 occasion >20 weeks’ gestation without proteinuria) | | 5 | 130 | | 9 | | 134 | 3.8% vs 6.7% | |
| Bonomo et al 2005 | Diet and nutrition counselling | | SGA (<10^th^ centile) | | 9 | 150 | | 13 | | 150 | 8.7% vs 6.0% | |
| Crowther et al 2005 | Diet and nutrition counselling | | SGA (<10^th^ centile) | | 33 | 506 | | 38 | | 524 | 6.5% vs 7.3% | |
| Kafatos et al 1989 | Diet and nutrition counselling | | SGA | | 12 | 205 | | 12 | | 199 | 5.9% vs 6.0% | |
| Khoury et al 2005 | Diet and nutrition counselling | | SGA (<10^th^ centile) | | 11 | 141 | | 12 | | 149 | 7.8% vs 8.1% | |
| Landon et al 2009 | Diet and nutrition counselling | | SGA (<10^th^ centile) | | 36 | 477 | | 29 | | 455 | 7.5% vs 6.4% | |
| Peccei et al 2017 | Diet and nutrition counselling | | SGA (<10^th^ centile) | | 11 | 180 | | 3 | | 92 | 6.1% vs 3.3% | |
| Bruno et al 2016 | Diet and nutrition counselling with physical exercise | | SGA (<10^th^ centile) | | 6 | 69 | | 5 | | 62 | 8.7% vs 8.1% | |
| Luoto et al 2011 | Diet and nutrition counselling with physical exercise | | SGA (<10^th^ centile) | | 10 | 216 | | 5 | | 179 | 4.7% vs 2.9% | |
| Poston et al 2015 | Diet and nutrition counselling with physical exercise | | SGA (<10^th^ centile) | | 53 | 761 | | 38 | | 750 | 7.0% vs 5.1% | |
| Renault et al 2014 | Diet and nutrition counselling with physical exercise | | SGA (a relative birthweight of 76% or less) | | 7 | 130 | | 2 | | 134 | 5.4% vs 1.5% | |
| Jahan et al 2013 | Diet and nutrition counselling | | LBW (<2500g) | | 3 | 150 | | 67 | | 150 | 2.7% vs 44.7% | |
| Phelan et al 2011 (normal weight) | Diet and nutrition counselling with physical exercise | | LBW (<2500g) | | 4 | 90 | | 5 | | 92 | 4.4% vs 5.4% | |
| Phelan et al 2011 (overweight) | Diet and nutrition counselling with physical exercise | | LBW (<2500g) | | 5 | 81 | | 4 | | 86 | 6.2% vs 4.7% | |
| Polley et al 2002 (normal weight) | Diet and nutrition counselling with physical exercise | | LBW (<2500g) | | 4 | 30 | | 3 | | 31 | 13.3% vs 9.7% | |
| Polley et al 2002 (overweight) | Diet and nutrition counselling with physical exercise | | LBW (<2500g) | | 1 | 27 | | 2 | | 22 | 3.7% vs 9.1% | |
| Poston et al 2015 | Diet and nutrition counselling with physical exercise | | LBW (<2500g) | | 31 | 761 | | 36 | | 751 | 4.1% vs 4.8% | |
| Briley et al 2002 | Diet and nutrition counselling | | PTB (<37 weeks) | | 0 | 10 | | 1 | | 10 | 0.0% vs 10.0% | |
| Harris et al 2015 | Diet and nutrition counselling | | PTB (<34 weeks) | | 3 | 191 | | 7 | | 121 | 1.6% vs 5.8% | |
| Kafatos et al 1989 | Diet and nutrition counselling | | PTB | | 9 | 228 | | 17 | | 201 | 3.9% vs 8.5% | |
| Khoury et al 2005 | Diet and nutrition counselling | | PTB (<37 weeks) | | 1 | 141 | | 11 | | 149 | 0.7% vs 7.4% | |
| Landon et al 2009 | Diet and nutrition counselling | | PTB (<37 weeks) | | 45 | 477 | | 53 | | 455 | 9.4% vs 11.6% | |
| Thornton et al 2009 | Diet and nutrition counselling | | PTB (<37 weeks) | | 3 | 116 | | 5 | | 116 | 2.6% vs 4.3% | |
| Walsh et al 2012 | Diet and nutrition counselling | | PTB (<37 weeks) | | 3 | 372 | | 8 | | 387 | 0.8% vs 2.1% | |
| Bruno et al 2016 | Diet and nutrition counselling with physical exercise | | PTB | | 0 | 69 | | 5 | | 62 | 0.0% vs 8.1% | |
| Dodd et al 2014 | Diet and nutrition counselling with physical exercise | | PTB (<37 weeks) | | 62 | 1075 | | 83 | | 1067 | 5.8% vs 7.8% | |
| Guelinckx et al 2010 | Diet and nutrition counselling with physical exercise | | PTB (<37 weeks) | | 2 | 65 | | 1 | | 65 | 3.1% vs 1.5% | |
| Phelan et al 2011 (normal weight) | Diet and nutrition counselling with physical exercise | | PTB (<36 weeks) | | 6 | 90 | | 13 | | 92 | 6.7% vs 14.1% | |
| Phelan et al 2011 (overweight) | Diet and nutrition counselling with physical exercise | | PTB (<36 weeks) | | 10 | 81 | | 7 | | 86 | 12.3% vs 8.1% | |
| Polley et al 2002 (normal weight) | Diet and nutrition counselling with physical exercise | | PTB (<36 weeks) | | 5 | 30 | | 2 | | 31 | 16.7% vs 6.5% | |
| Polley et al 2002 (overweight) | Diet and nutrition counselling with physical exercise | | PTB (<36 weeks) | | 2 | 27 | | 3 | | 22 | 7.4% vs 13.6% | |
| Poston et al 2015 | Diet and nutrition counselling with physical exercise | | PTB (<37 weeks) | | 45 | 761 | | 48 | | 751 | 5.9% vs 6.4% | |
| Renault et al 2014 | Diet and nutrition counselling with physical exercise | | PTB (<37 weeks) | | 4 | 130 | | 6 | | 134 | 3.1% vs 4.5% | |
| Crowther et al 2005 | Diet and nutrition counselling | | Stillbirth | | 0 | 506 | | 3 | | 524 | 0.0% vs 0.6% | |
| Kafatos et al 1989 | Diet and nutrition counselling | | Stillbirth | | 2 | 223 | | 5 | | 208 | 0.9% vs 2.4% | |
| Khoury et al 2005 | Diet and nutrition counselling | | Stillbirth | | 0 | 141 | | 1 | | 149 | 0.0% vs 0.7% | |
| Landon et al 2009 | Diet and nutrition counselling | | Stillbirth | | 0 | 485 | | 0 | | 473 | NA | |
| Walsh et al 2012 | Diet and nutrition counselling | | Stillbirth (fetal demise at >24 weeks’ gestation) | | 1 | 394 | | 0 | | 406 | 0.3% vs 0.0% | |
| Dodd et al 2014 | Diet and nutrition counselling with physical exercise | | Stillbirth (intrauterine fetal death  after 20 weeks’ gestation and before birth) | | 5 | 1018 | | 5 | | 1104 | 0.5% vs 0.5% | |
| Dodd et al 2014 | Diet and nutrition counselling with physical exercise | | Maternal death | | 1 | 1018 | | 1 | | 1104 | 0.1% vs 0.1% | |
